# Photomediated C–H trifluoromethoxylations enabled by bis(trifluoromethyl)peroxide

**DOI:** 10.1039/d5sc04945h

**Published:** 2025-08-25

**Authors:** Kamar Shakeri, Merlin Kleoff, Paul Golz, Thomas Drews, Manuela Weber, Sebastian Riedel, Mathias Christmann

**Affiliations:** a Freie Universität Berlin, Institute of Chemistry and Biochemistry, Organic Chemistry Takustr. 3 14195 Berlin Germany mathias.christmann@fu-berlin.de; b Freie Universität Berlin, Institute of Chemistry and Biochemistry, Inorganic Chemistry Fabeckstr. 34/36 Berlin 14195 Germany s.riedel@fu-berlin.de

## Abstract

We describe a photomediated protocol for the trifluoromethoxylation of benzylic, aldehydic, and non-activated C–H bonds, using bis(trifluoromethyl)peroxide (BTMP, (F_3_CO)_2_) as the key reagent. Under catalyst-free conditions in acetone, this reaction proceeds with selective functionalization of benzylic methylene groups. Furthermore, by using tetrabutylammonium decatungstate as a photocatalyst, the scope extends to include both non-activated methylene C(sp^3^)–H and formyl C(sp^2^)–H bonds. The methodology was successfully applied to 24 examples including odorants, pharmaceuticals, and natural products and was demonstrated on gram scale. Finally, by using [^13^C]-BTMP, the corresponding trifluoromethoxy groups can be site-specifically labeled with ^13^C.

## Abstract 2

Bis(trifluoromethyl)peroxide (BTMP) enables atom-economic photomediated C–H trifluoromethoxylation of benzylic, non-activated, and aldehydic positions. Additionally, [^13^C]-BTMP can be used for isotopic labeling and trifluoromethoxylation in one step.

## Graphical abstract

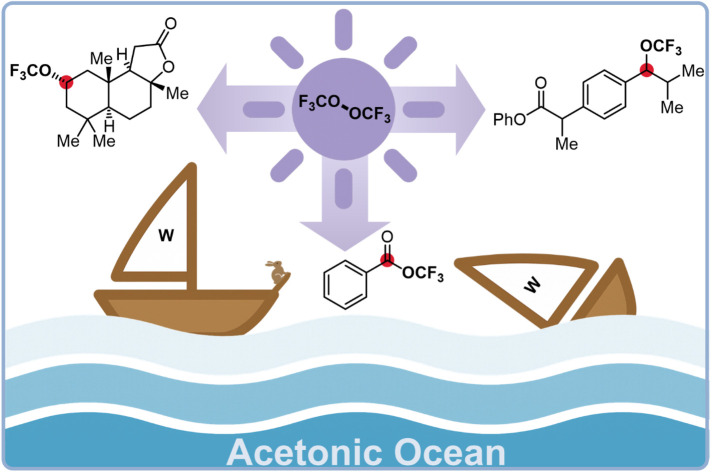

## Introduction

The strategic incorporation of fluorine into agrochemicals and pharmaceuticals has become critical for optimizing their metabolic stability, lipophilicity, and conformational properties.^1–8^ However, increasing concerns over the persistence of many fluorinated compounds have led the EU to initiate a ban on per- and polyfluoroalkyl substances (PFAS).^9^ Notably, compounds bearing trifluoromethoxy (–OCF_3_) groups are exempt from this regulation, as they degrade under biological conditions into non-persistent molecules.^10^

**Scheme 1:**
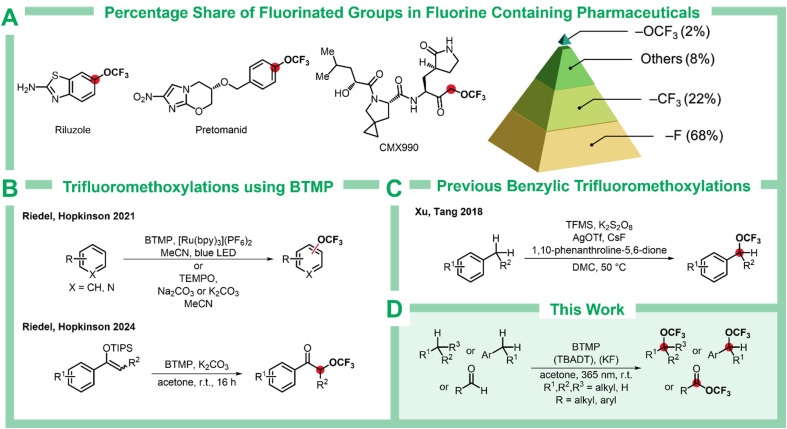
(A) Percentage share of fluorinated groups in fluorine containing substrates in pharmaceutical industry and some examples. (B) Previous trifluoromethoxylations using BTMP. (C) Benzylic C–H trifluoromethoxylation by Xu, Tang, and coworkers. (D) C–H trifluoromethoxylation of benzylic, non-activated, and aldehydic C–H bonds. Bpy = 2,2′-bipyridine, TEMPO =(2,2,6,6-tetramethyllpiperidin-1-yl)oxyl, TFMS = trifluoromethyl 4-fluorobenzenesulfonate, Tf = trifluoromethanesulfonyl, DMC = dimethyl carbonate, TBADT = tetrabutylammonium decatungstate.

Despite the well-established benefits of incorporating fluorine into drug design, trifluoromethoxy (–OCF_3_) groups remain significantly underrepresented in approved pharmaceuticals. While 68% of fluoro-pharmaceuticals contain a C–F bond and 22% feature a C–CF_3_ moiety, only 2% bear a C–OCF_3_ group (Scheme 1).^11^ This scarcity is particularly pronounced for C(sp^3^)–OCF_3_ containing compounds. The limited utilization of this valuable functional group can be largely attributed to the lack of efficient and broadly applicable synthetic methods for accessing trifluoromethoxylated molecules. Direct C–H functionalization strategies for the installation of trifluoromethoxy groups offer a particularly promising route to streamline access to OCF_3_-containing targets in drug discovery.^12–22^

Although numerous methods have been reported for the C–H trifluoromethoxylation of aromatic compounds,^9–27^ the corresponding transformations on aliphatic C–H bonds remain largely unexplored.^28^ Only one method has been reported for the benzylic C–H trifluoromethoxylation, developed by Xu, Tang, and coworkers, which requires the use of silver salts in catalytic to stoichiometric amounts depending on the substrate.^29^ Moreover, no general procedure exists for the trifluoromethoxylation of non-activated alkanes, significantly restricting access to trifluoromethoxylated alkyl derivatives.

Hopkinson and Riedel recently demonstrated the use of bis(trifluoromethyl)peroxide (BTMP, (F_3_CO)_2_) for trifluoromethoxylation of arenes, silyl enol ethers, and allyl silanes.^30–35^ Despite being a peroxide, BTMP remains remarkably stable up to 200 °C, is an easy to handle gas, and can be prepared in large quantities from abundant starting materials.^36,37^ Its two –OCF_3_ units provide superior atom economy compared to other trifluoromethoxylation reagents.^30,31^ Here, we report that BTMP is a versatile reagent for the direct C–H trifluoromethoxylation of benzylic positions, non-activated alkanes, and aldehydes.

## Results and discussion

To investigate the C–H trifluoromethoxylation of benzylic positions using BTMP, we selected ethylbenzene (1a) as a model substrate for screening. Given the established versatility of decatungstate catalysis in photochemical C–H functionalizations,^37–42^ tetrabutylammonium decatungstate (TBADT) was selected as the catalyst. Subsequent reaction with BTMP was expected to yield the corresponding trifluoromethyl ether 2a. Initial experiments involved irradiating a solution of ethylbenzene (1a) and TBADT in acetonitrile under UV light for 18 h. This reaction provided 2a in a yield of 18% (Table 1, entry 1). To optimize the reaction conditions, acetone was investigated as an alternative solvent, which significantly improved the yield of 2a to 55% (Table 1, entry 2).

**Table 1:** Optimization and Control Experiments

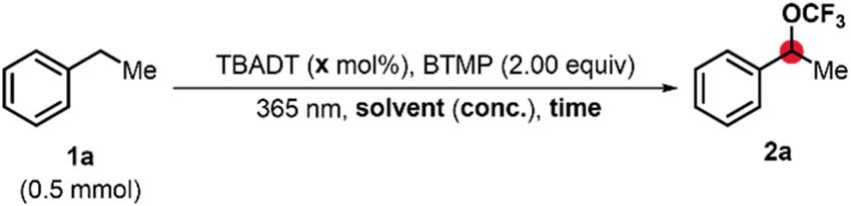					
Entry	*X* (mol%)	Solvent	Conc. (m)	Time [h]	Yield^a^ (%)
1	5	MeCN	0.24	18	18
2	5	Acetone	0.24	18	55
3	5	Acetone/HFIP (10 : 1)	0.24	18	34
4^b^	5	Acetone	0.24	18	29
5	5	Acetone	0.48	18	46
6	10	Acetone	0.24	18	31
7^c^	5	Acetone	0.24	18	38
8	5	Acetone	0.24	2	60
9	5^d^	Acetone	0.24	2	27
10^e^	5	Acetone	0.24	4	Traces
11	—	**Acetone**	**0.24**	**4**	**66**

a Determined by ^19^F NMR spectroscopy using α,α,α-trifluorotoluene (PhCF_3_) as internal standard.

b Performed with 1.00 equivalent KF as additive.

c Using 4 equivalents BTMP.

d Using NaDT instead of TBADT.

e Reaction conducted under exclusion of light.

Addition of hexafluoroisopropanol (HFIP) resulted in a reduced yield of 34% (Table 1, entry 3). Similarly, KF as an additive showed no improvement (Table 1, entry 4).

Changing the reaction molarity or catalyst loading also diminished yields (Table 1, entries 5 and 6). Also, increasing the amount of BTMP to 4 equivalents lead to a decreased yield of 38% (Table 1, entry 7). Shortening the reaction time to 2 h significantly increased the yield to 60% (Table 1, entry 8). Longer reaction times resulted in further decomposition of the substrate. The substitution of TBADT with sodium decatungstate (NaDT) resulted in a lower yield (Table 1, entry 9). Control experiments conducted under exclusion of light yielded only trace amounts of 2a (Table 1, entry 10). Surprisingly, performing the reaction in the absence of a catalyst gave the highest yield of 66% (Table 1, entry 11).

**Scheme 2:**
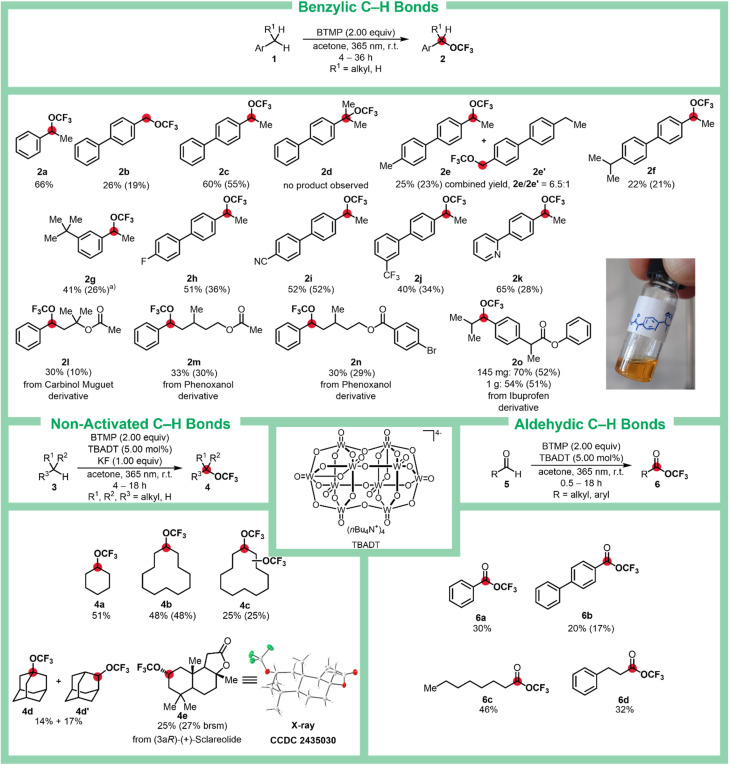
Scope of trifluoromethyl ethers 2, 4, and 6, by C–H trifluoromethoxylation using BTMP. All yields were determined by ^19^F NMR spectroscopy using α,α,α-trifluorotoluene (PhCF_3_) as internal standard. Isolated yields are given in parenthesis. (a) Obtained as a 2 : 1 mixture with the starting material determined by ^1^H NMR spectroscopy.

With the optimized conditions established, the trifluoromethoxylation protocol was explored over a range of benzylic substrates (Scheme 2). First, the effect of primary, secondary, and tertiary benzylic positions on the reaction outcome was investigated. For a substrate with a benzylic methyl group, the corresponding trifluoromethoxy derivative 2b was obtained in 19% yield. In contrast, a substrate with a benzylic methylene group showed a significant improvement, yielding 2c in 55%. However, when a tertiary benzylic substrate was employed, no formation of 2d was observed.

The selectivity for benzylic methylene groups was demonstrated through competition experiments using biphenyl derivatives with ethyl/methyl or ethyl/isopropyl substituents, as exemplified by 2e and 2f, respectively. Starting from 4-ethyl-4′-methyl-1,1′-biphenyl, trifluoromethoxylation yielded 2e and its constitutional isomer 2e′ in a 6.5 : 1 ratio, with a combined yield of 23%, demonstrating a strong preference for the methylene group over the methyl group. In the presence of a tertiary benzylic C–H bond, functionalization occurred exclusively in the secondary benzylic position, yielding 2f with an isolated yield of 21%.

The protocol was shown to exhibit tolerance for functional groups such as *tert*-butyl (2g, 26% yield), fluorine (2h, 36% yield), cyano (2i, 52% yield), and trifluoromethyl (2j, 34% yield) substituents. A pyridinyl group was also tolerated, affording 2k in 28% yield. Furthermore, the stability of aliphatic and benzylic esters under the reaction conditions was demonstrated by the synthesis of trifluoromethoxylated derivatives of the odorants Carbinol Muguet (2l, 10% yield), phenoxanol (2m, 30% yield), and 2n (29% yield).

Notably, the Ibuprofen derivative 1o was selectively functionalized at the secondary benzylic position, affording trifluoromethyl ether 2o in 52% yield. To showcase the scalability of the trifluoromethoxylation protocol, 2o was also prepared on a gram scale, achieving a comparable yield of 51%.

The benzylic trifluoromethoxylation using BTMP offers a significant improvement in the atom economy.^43^ For instance, the synthesis of compound 2c requires a total reagent-to-substrate mass ratio of 2 : 1 with BTMP (55% yield), compared to the 15 : 1 ratio reported in the method by Xu, Tang, and coworkers (58% yield) (see SI, chapter 3).^29^

Next, we focused on the functionalization of non-activated and aldehydic C–H bonds (see SI, chapter 7, for screening details). Using TBADT and KF in acetone, we obtained trifluoromethylcyclohexyl ether (4a) in 51% yield, as determined by ^19^F NMR. With cyclododecane as the substrate, 4b was isolated as a colorless liquid in 48% yield. Notably, 4b could undergo a second functionalization under the same reaction conditions, affording the double-functionalized product 4c in 25% yield. Additionally, adamantane was C–H trifluoromethoxylated at both the secondary and tertiary positions, yielding 4d and 4d′ in 14% and 17% ^19^F NMR yield, respectively. The protocol was also successfully applied to the natural product (3aR)-(+)-sclareolide, providing the corresponding trifluoromethylether 4e as a single diastereomer in 25% yield. The structure of 4e was unambiguously confirmed by single-crystal X-ray diffraction, with crystals of 4e obtained through slow evaporation of a solution in a dichloromethane–cyclohexane mixture.

Expanding the scope of C–H activated groups to aldehydes enabled the preparation of trifluoromethyl esters 6a–6d, providing a novel approach to access these compounds.^44^ For benzaldehyde, the corresponding ester 6a was obtained in 30% ^19^F NMR yield. Using the heavier analog 5b, trifluoromethyl ester 6b was isolated in 17% yield. Aliphatic aldehydes were also successfully transformed, as evidenced by the formation of 6c (46% ^19^F NMR yield) and 6d (32% ^19^F NMR yield).

**Scheme 3:**
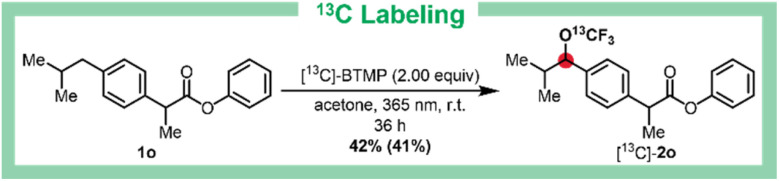
One step trifluoromethoxylation and ^13^C labeling of Ibuprofen derivative 1o. Yield was determined by ^19^F NMR spectroscopy using α,α,α-trifluorotoluene (PhCF_3_) as internal standard. Isolated yield is given in parenthesis.

**Scheme 4:**
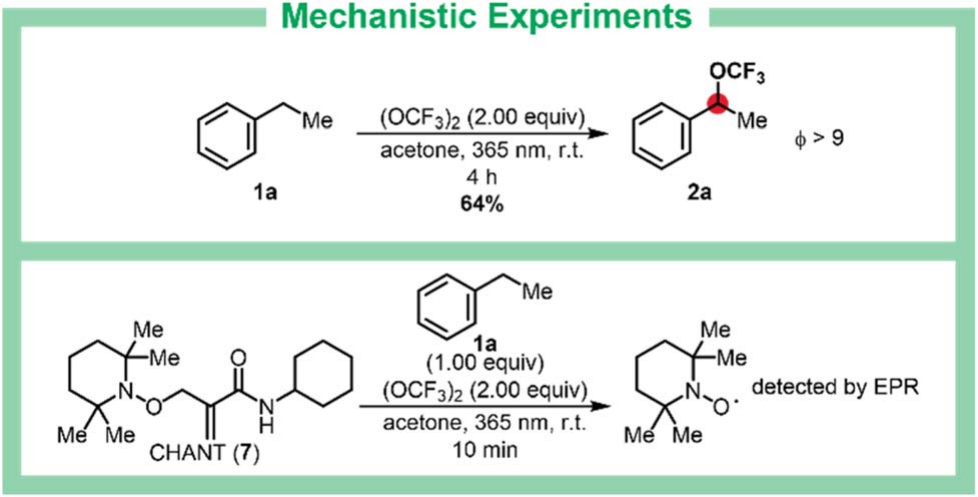
Mechanistic Experiments. Yield was determined by ^19^F NMR spectroscopy using α,α,α-trifluorotoluene (PhCF_3_) as internal standard. CHANT = *N*-cyclohexyl-2-(((22,66-tetramethylpiperidin-1-yl)oxy)methyl)acrylamide, TEMPO = (2,2,6,6-tetramethylpiperidin-1-yl)oxyl.

Stable-isotope-labeled substrates are crucial in chemical, biomedical, and environmental research, offering powerful tools for monitoring drugs in biological systems, tracking metabolic processes, and accelerating drug discovery.^45–49^ To this end, we developed a synthesis of ^13^C-labeled bis(trifluoromethyl)peroxide (^13^[C]-BTMP) from commercially available ^13^CO and F_2_ (see SI, chapter 2). This reagent enables simultaneous C–H trifluoromethoxylation and ^13^C-labeling of various compounds in a single step using our C–H trifluoromethoxylation protocol. As an example, the ^13^C-labeled ibuprofen derivative [^13^C]-2o was prepared in 41% isolated yield using [^13^C]-BTMP (Scheme 3). For a deeper mechanistically insight into the catalyst-free benzylic trifluoromethoxylation, quantum yield experiment and EPR spectroscopy were conducted (Scheme 4).^50^ The quantum yield of *Φ* > 9 indicates a chain reaction process.^51^ Notably, neither UV/Vis spectra of the reaction mixture nor of acetone mixed with BTMP shows significant difference to the UV/Vis spectra of pure acetone. But when mixing acetone with BTMP and irradiating the mixture for 4 h, mesityl oxide as well as phorone could be detected by HRMS. During the irradiation process, there must be the formation of these two species from acetone. We cannot exclude the existence of traces of mesityl oxide or phorone at the beginning of the reaction mixture even though we could not detect any in HRMS or UV/Vis before starting irradiation. This leak of absorption hints quite efficient radical chain process. The release of TEMPO from *N*-cyclohexyl-2-(((2,2,6,6-tetramethylpiperidin-1-yl)oxy)methyl)acrylamide (7) (CHANT)^52^ (proven by EPR) under standard reaction conditions shows formation of a radical species (see SI, chapter 8).

**Scheme 5:**
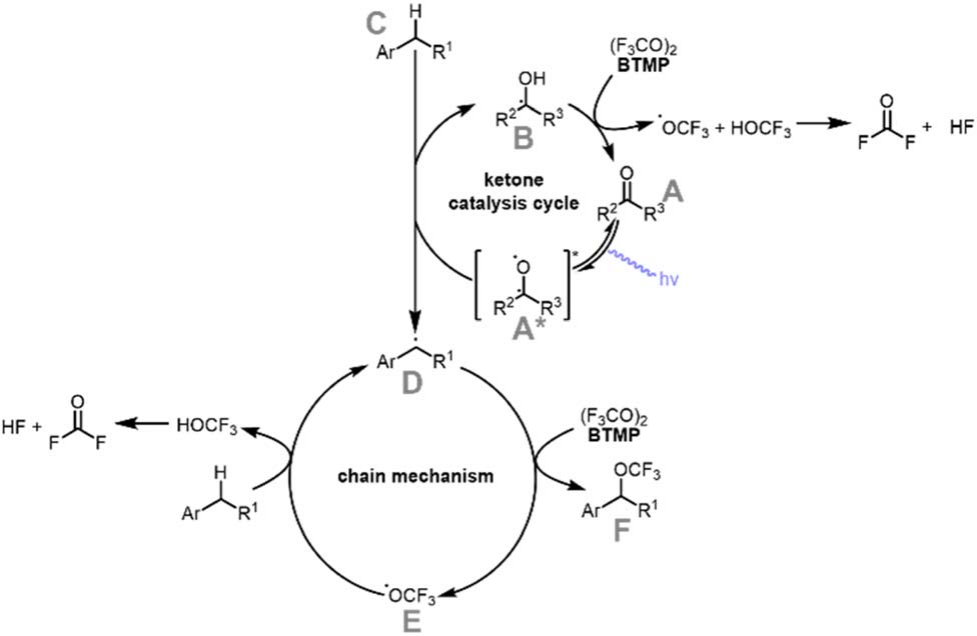
Plausible reaction mechanism.

On the base of these observations, a plausible reaction mechanism suggests a radical chain propagation pathway for the trifluoromethoxylation of benzylic C–H bonds (Scheme 5).

The proposed reaction mechanism begins with the excitation of acetone or traces of mesityl oxide or phorone (A) by UV light irradiation, potentially forming the excited state A*. A subsequent hydrogen atom transfer (HAT) step may lead to the formation of the intermediate B, which is hypothesized to reduce BTMP. This reduction could trigger mesolytic cleavage, forming an OCF_3_ radical and HOCF_3_. The HOCF_3_ is proposed to further decompose to fluorophosgene and HF. Acetone appears to be regenerated in the process, possibly completing the catalytic cycle. In parallel, a benzylic radical (D) could be generated, which is proposed to react with BTMP to yield the functionalized product F and another OCF_3_ radical (E). This radical could propagate the chain reaction by abstracting a hydrogen atom from another benzylic C–H bond, regenerating radical D and maintaining the reaction cycle.

## Conclusion

In conclusion, we have developed a C–H trifluoromethoxylation protocol utilizing BTMP as an OCF_3_ source, enabling the functionalization of benzylic, non-activated, and aldehydic positions. Notably, trifluoromethoxylation of benzylic positions was achieved even in the absence of a catalyst. For non-activated and aldehydic C–H bonds, the use of TBADT as a catalyst expanded the scope of the methodology. The protocol was successfully applied to 24 examples, delivering products in moderate to good yields, and its scalability was demonstrated by performing the reaction on a gram scale.

Additionally, we synthesized a ^13^C-labeled peroxide ([^13^C]-BTMP), which facilitates simultaneous C–H trifluoromethoxylation and ^13^C labeling in a single step, as exemplified by the preparation of the Ibuprofen derivative [^13^C]-2o. Finally, mechanistic studies suggest the possibility of a radical chain mechanism.

## Author contributions

K. S., M. K., S. R., and M. C. conceived the methodology. K. S. carried out the synthetic experiments. P. G. and T. D. prepared BTMP. M. W. performed XRD measurment. K. S., M. K., S. R., and M. C. wrote the manuscript.

## Conflicts of interest

K. S., M. K., P. G., S. R., and M. C. are inventors of a pending patent related to this work, submitted by Freie Universität Berlin (25174771.3). The authors declare that they have no other competing interests.

## Supplementary Material

SC-OLF-D5SC04945H-s001

SC-OLF-D5SC04945H-s002

## Data Availability

CCDC 2435030 (4e) contains the supplementary crystallographic data for this paper.^53^ Supplementary information: The data supporting this article have been included as part of the SI. See DOI: https://doi.org/10.1039/d5sc04945h.
