# Supplementary material for: Photomediated C–H trifluoromethoxylations enabled by bis(trifluoromethyl)peroxide
Source: Chem Sci. 2025 Aug 25;16(38):17921–6. doi: 10.1039/d5sc04945h (PMC12415769; doi:10.1039/d5sc04945h)
Supplement: SC-016-D5SC04945H-s001 [file SC-016-D5SC04945H-s001.pdf]

Supplementary Information (SI) for Chemical Science.
This journal is © The Royal Society of Chemistry 2025

Photomediated C–H Trifluoromethoxylations Enabled by Bis(trifluoromethyl)peroxide

Kamar Shakeri,[a] Merlin Kleoff,[b] Paul Golz,[b] Thomas Drews,[b] Manuela Weber,[b] Sebastian Riedel,*[b] and Mathias Christmann*[a]

[a] Institute of Chemistry and Biochemistry, Organic Chemistry Freie Universität Berlin, 14195 Berlin, Germany

[b] Institute of Chemistry and Biochemistry, Inorganic Chemistry Freie Universität Berlin, 14195 Berlin, Germany

*E-Mail: m.christmann@fu-berlin.de

Supporting Information

Table of Contents

1. General Information....1
    1.1 Materials and Methods....1
    1.2 Analysis....1
2. Synthesis of [^{13}C]-BTMP....3
    2.1 Synthesis of (^{13}C)Carbonyl Difluoride....3
    2.2 Synthesis of (^{13}C)Bis(trifluoromethyl)peroxide....3
    2.3 Determination of Solubility of BTMP....4
3. Calculating the Atom Economics of Bis(trifluoromethyl)peroxide (BTMP) as Trifluoromethoxylation Agent....5
    3.1 Xu and Tang's Methodology....5
    3.2 This Work using BTMP....6
4. Calculating the Amount of Bis(trifluoromethyl)peroxide (BTMP)....7
5. Performing Trifluoromethoxylation – Handling the Gaseous Peroxide depicted using [^{13}C]-BTMP....8
6. Optimization of the Trifluoromethoxylation of Benzylic C–H Bonds....12
7. Optimization of the Trifluoromethoxylation of Non-Activated C–H Bonds....14
8. Reaction Mechanism....15
    8.1 Determination of the Photon Flux of the LED Chip....15
    8.2 Determination of the Reaction Quantum Yield (Φ)....16
    8.3 EPR Spectra, UV/Vis Spectra, HRMS Data after irradiation of Acetone mixed with BTMP....17
    8.4 Proposed Reaction Mechanism for Trilfuoromethoxylation in the absence of TBADT....20
    8.5 Proposed Reaction Mechanism for Trilfuoromethoxylation in the absence of TBADT....21
9. General Procedures....22
    9.1 General Procedure for Suzuki Coupling (GP1)....22
    9.2 General Procedure for the Acetylation of Alcohols 2j and 2k (GP2)....22
    9.3 General Procedure for Trifluoromethoxylation of Benzylic C–H Bonds (GP3)....22
    9.4 General Procedure for Trifluoromethoxylation of Non-activated C–H Bonds (GP4)....23
    9.5 General Procedure for Trifluoromethoxylation of Aldehydic C–H Bonds (GP5)....23
10. Synthesis of Starting Materials....25
    10.1 4-Methyl-1,1'-biphenyl (1b)....25
    10.2 4-Ethyl-1,1'-biphenyl (1c)....25
    10.3 4-Isopropyl-1,1'-biphenyl (1d)....26
    10.4 4-Ethyl-4'-methyl-1,1'-biphenyl (1e)....27
    10.5 4-Ethyl-4'-isopropyl-1,1'-biphenyl (1f)....27
    10.6 4-Ethyl-4'-fluoro-1,1'-biphenyl (1h)....28
    10.7 4'-Ethyl-[1,1'-biphenyl]-4-carbonitrile (1i)....29

10.8 4'-Ethyl-3-(trifluoromethyl)-1,1'-biphenyl (1j) 29
10.9 2-(4-Ethylphenyl)pyridine (1k) 30
10.10 2-Methyl-4-phenylbut-2-yl acetate (1l) 31
10.11 3-Methyl-5-phenylpentyl acetate (1m) 31
10.12 3-Methyl-5-phenylpentyl 4-bromobenzoate (1n) 32
10.13 Phenyl 2-(4-isobutylphenyl)propanoate (1o) 32
10.14 [1,1'-Biphenyl]-4-carbaldehyde (5b) 33
11. Synthesis of Trifluoromethoxylated Products 35
11.1 (1-(Trifluoromethoxy)ethyl)benzene (2a) 35
11.2 4-((Trifluoromethoxy)methyl)-1,1'-biphenyl (2b) 35
11.3 4-(1-(Trifluoromethoxy)ethyl)-1,1'-biphenyl (2c) 36
11.4 4-Methyl-4'-(1-(trifluoromethoxy)ethyl)-1,1'-biphenyl (2e) and 4-ethyl-4'-((trifluoromethoxy)methyl)-1,1'-biphenyl (2e') 36
11.5 4-Isopropyl-4'-(1-(trifluoromethoxy)ethyl)-1,1'-biphenyl (2f) 37
11.6 1-(Tert-butyl)-3-(1-(trifluoromethoxy)ethyl)benzene (2g) 38
11.7 4-Fluoro-4'-(1-(trifluoromethoxy)ethyl)-1,1'-biphenyl (2h) 38
11.8 4'-(1-(Trifluoromethoxy)ethyl)-[1,1'-biphenyl]-4-carbonitrile (2i) 39
11.9 4'-(1-(Trifluoromethoxy)ethyl)-3-(trifluoromethyl)-1,1'-biphenyl (2j) 39
11.10 2-(4-(1-(Trifluoromethoxy)ethyl)phenyl)pyridine (2k) 40
11.11 2-Methyl-4-phenyl-4-(trifluoromethoxy)butanyl acetate (2l) 40
11.12 3-Methyl-5-phenyl-5-(trifluoromethoxy)pentyl acetate (2m) 41
11.13 3-Methyl-5-phenyl-5-(trifluoromethoxy)pentyl 4-bromobenzoate (2n) 42
11.14 Phenyl 2-(4-(2-methyl-1-(trifluoromethoxy)propyl)phenyl)propanoate (2o) 42
11.14.1 Gram scale: Phenyl 2-(4-(2-methyl-1-(trifluoromethoxy)propyl)phenyl)propanoate (2o) .. 43
11.15 (Trifluoromethoxy)cyclohexane (4a) 43
11.16 (Trifluoromethoxy)cyclododecane (4b) 44
11.17 Bis(trifluoromethoxy)cyclododecane (4c) 44
11.18 (3*S*,5*S*,7*S*)-1-(Trifluoromethoxy)adamantane (4d) and (1*R*,3*R*,5*R*,7*R*)-2-(trifluoromethoxy)adamantane (4d') 45
11.19 (3a*R*,8*S*,9a*S*)-3a,6,6,9a-Tetramethyl-8-(trifluoromethoxy)decahydronaphtho[2,1-b]furan-2(1H)-one (4e) 46
11.20 Trifluoromethyl benzoate (6a) 46
11.21 Trifluoromethyl [1,1'-biphenyl]-4-carboxylate (6b) 47
11.22 Trifluoromethyl octanoate (6c) 47
11.23 Trifluoromethyl 3-phenylpropanoate (6d) 48
11.24 Phenyl 2-(4-(2-methyl-1-(trifluoromethoxy-^{13}C)propyl)phenyl)propanoate ([^{13}C]-2o) 48
12. Synthesis of S2, S4, S5, S6, and S8 49
12.1 2-(4-Ethylphenyl)-4,4,5,5-tetramethyl-1,3,2-dioxaborolane (S2) 49
12.2 *N*-(4'-Ethyl-[1,1'-biphenyl]-4-yl)acetamide (S4) 49

12.3 4-(4-Phenoxyphenyl)butan-2-one (S5) 50
12.4 (*E*)-4-(4-Phenoxyphenyl)but-3-en-2-one (S6) 51
12.5 4-(((8*R*,9*S*,13*S*,14*S*)-13-Methyl-17-oxo-7,8,9,11,12,13,14,15,16,17-decahydro-6*H*-cyclopenta[*a*]phenanthren-3-yl)oxy)benzaldehyde (S10) 51
13 Limitations 53
14. X-ray Data of (3a*R*,8*S*,9a*S*)-3a,6,6,9a-Tetramethyl-8-(trifluoromethoxy)decahydronaphtho[2,1-b]furan-2(1*H*)-one (4e) 54
.... 54
15. NMR and HRMS Spectra of Synthesized Substrates 55
16. References 123

1. General Information

1.1 Materials and Methods

Reactions with air or moisture sensitive substances were carried out under an argon atmosphere using standard Schlenk techniques. Room temperature (r.t.) refers to 18 – 23 °C. Heating of reactions was performed with an oil bath unless otherwise noted.

Unless otherwise noted, all starting materials and reagents were purchased from commercial distributors and used without further purification. Anhydrous dichloromethane and tetrahydrofuran were provided by purification with a MBraun SPS-800 solvent system (BRAUN) using solvents of HPLC grade purchased from FISHER Scientific and ROTH.
Solvents for extraction, crystallization, and flash column chromatography were purchased in technical grade and distilled under reduced pressure prior to use.
Tetra-*n*-butylammonium decatungstate (TBADT)[1] and sodium decatungstate[2] were synthesized using literature known procedures.

Column chromatography was performed on silica 60 M (0.040–0.063 mm, 230–400 mesh, MACHEREY-NAGEL).

Medium pressure liquid chromatography (MPLC) was performed with a TELEDYNE ISCO Combi-Flash Rf200 using prepacked silica columns and cartridges from TELDYNE. UV response was monitored at 254 nm and 280 nm. As eluents, cyclohexane (CyH, 99.5+% quality) and EtOAc (HPLC grade) were used.

1.2 Analysis

Reaction monitoring: Reactions were monitored by thin layer chromatography (TLC). TLC analysis was performed on silica gel coated aluminum plates ALUGRAM® Xtra SIL G/UV254 purchased from MACHEREY-NAGEL. Products were visualized by UV

light at 254 nm and by using staining reagents (based on $KMnO_4$, $Ce(SO_4)_2$, or anisaldehyde).

NMR spectroscopy: ^{1}H, ^{19}F, and ^{13}C NMR spectra were acquired on a JEOL (ECX 400, ECP 500), VARIAN (INOVA 600), and BRUKER (AVANCE III 500, AVANCE III 700) and analyzed on MestReNova 14.3.0. The chemical shifts (δ) are listed in parts per million (ppm) and are reported relative to the corresponding residual non-deuterated solvent signal ($CDCl_3$: δ_H = 7.26 ppm, δ_C = 77.2 ppm; CD_2Cl_2: δ_H = 5.32 ppm, δ_C = 53.8 ppm; DMSO-d_6: δ_H = 2.50 ppm, δ_C = 39.5 ppm). ^{19}F NMR spectra are not calibrated by an internal reference. ^{19}F NMR yields were measured using α,α,α-trifluorotoluene ($PhCF_3$) as an internal standard.
Integrals are in accordance with assignments; coupling constants (*J*) are given in Hz. Multiplicity is indicated as follows: singlet (s), doublet (d), triplet (t), quartet (q), quintet (quin), septet (sept), broad (br), and combinations thereof. In the case where no multiplicity could be identified, the chemical shift range of the signal is given as m (multiplet). ^{13}C NMR spectra are ^{1}H-broadband decoupled.

High resolution mass spectrometry: High resolution mass spectra (HRMS) were measured with an AGILENT 6210 ESI-TOF (10 µL/min, 1.0 bar, 4 kV) instrument.

X-ray: X-ray diffraction data was collected on a BRUKER D8 Venture CMOS area detector (Photon 100) diffractometer with Cu Kα radiation. Single crystals were coated with perfluoroether oil and mounted on a 0.2 mm Micromount. The structures were solved with the ShelXT1 structure solution program using intrinsic phasing and refined with the ShelXL2 refinement package using least squares on weighted F2 values for all reflections using OLEX2.

Light source:
A self-assembled and partially 3d-printed setup was used for all photoreactions unless otherwise stated. As a light source, CHANZON LED chips (365 nm, 30 W) were used and cooled from the bottom using a water cooler (40 × 40 mm, made from aluminum from the company KALOLARY).

2. Synthesis of [^{13}C]-BTMP

2.1 Synthesis of (^{13}C)Carbonyl Difluoride

(^{13}C)Carbonyl difluoride was synthesized from silver(II) fluoride and (^{13}C)carbon monoxide according to a literature procedure.[3] A stainless steel reactor with a total volume of 780 mL, filled with silver(II) fluoride (57.0 g, 391 mmol, 10.2 equiv) was pressurized with 1200 mbar (^{13}C)carbon monoxide (1.11 g, 38.4 mmol, 1.00 equiv). After 24 h the obtained gas was purified by trap-to-trap distillation (–160 °C, and –196 °C). The trap at –160 °C contained (^{13}C)carbonyl difluoride (2.49 g, 37.2 mmol, 97%).
IR (gas): $\tilde{\nu}$ [cm^{-1}] = 1882 (vs), 1207 (vs), 958 (s), 749 (m), 633 (w), 588 (w).

2.2 Synthesis of (^{13}C)Bis(trifluoromethyl)peroxide

(^{13}C)Bis(trifluoromethyl)peroxide was synthesized from elemental fluorine and (^{13}C)carbonyl difluoride according to a literature procedure.[4] A stainless steel reactor with a total volume of 350 mL filled with silver fluoride catalyst was pressurized with 1200 mbar (^{13}C)carbonyl difluoride (1.16 g, 17.3 mmol, 1.50 equiv) and 800 mbar fluorine (400 mg, 11.5 mmol, 1.00 equiv). The mixture was held at 90 °C for 24 h and then passed over soda lime to deactivate trifluoromethyl hypofluorite, residual fluorine, and carbonyl difluoride. The gas obtained was then purified by trap-to-trap distillation (–80 °C, –160 °C, and –196 °C). (^{13}C)bis(trifluoromethyl)peroxide (1.35 g, 7.80 mmol, 92%) was collected in the –160 °C trap and stored in a stainless steel vessel.
IR (gas): $\tilde{\nu}$ [cm^{-1}] = 1248 (vs), 1277 (vs), 1205 (s), 1136 (vs), 1063 (vw), 889 (vw), 707 (vw), 629 (w).

Safety note: Caution! Extreme caution should be exercised when working with elemental fluorine, carbonyl difluoride, and hypofluorites. Beyond their toxic nature, explosions have been reported in the literature during handling of these extremely hazardous compounds in conjunction with easily oxidizable organic matter or carbon monoxide.

2.3 Determination of Solubility of BTMP

The solubility of bis(trifluoromethyl)peroxide was determined using gravimetric measurements in a sealed, gas-tight flask with varying peroxide pressures. A specified volume of either acetonitrile (74.9 g, 96.1 mL) or acetone (70.6 g, 89.3 mL) was added to the flask, which was then degassed via a freeze-pump-thaw protocol to remove any dissolved gases. The flask was subsequently equilibrated at 20 °C and pressurized with bis(trifluoromethyl)peroxide. Once the pressure stabilized, the mass of the resulting diluted peroxide solution was measured. The determined masses were corrected by the calculated mass of gaseous peroxide.

Table S1. Solubility of BTMP in acetonitrile and acetone.

	$P_p(F_3CO)_2$ [mbar]	Solubility of $(F_3CO)_2$	
		[mg/g]	[mol/L]
Acetone	606	8.0	37.1
	707	9.5	44.3
	866	11.9	55.4
	963	13.5	62.5
	1068	15.1	70.1
Acetonitrile	603	3.6	16.6
	709	4.0	18.4
	804	4.4	20.1
	908	5.1	23.2
	1007	5.7	26.3

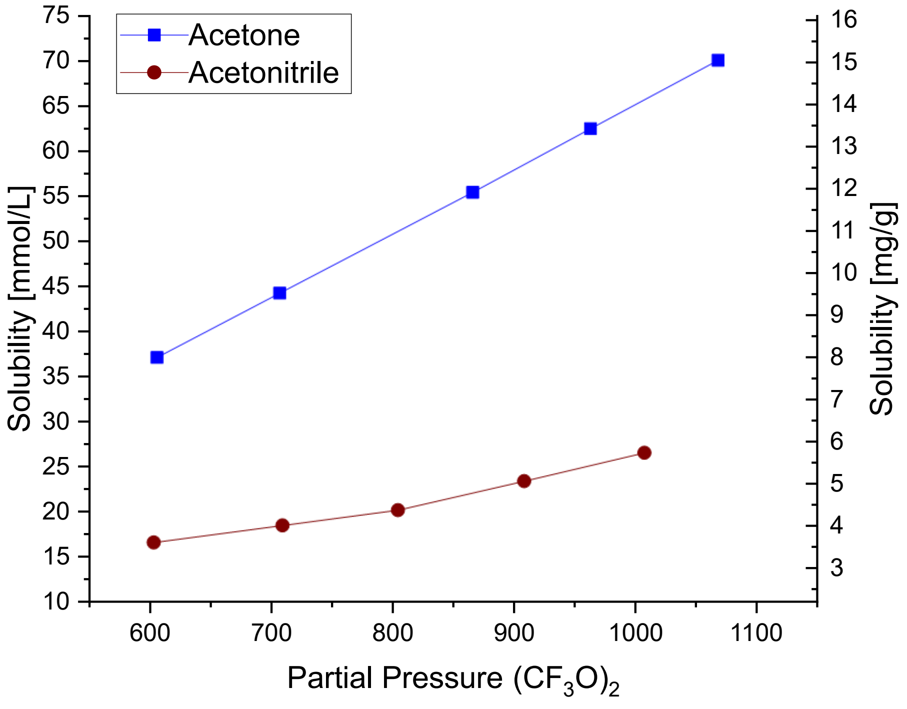

3. Calculating the Atom Economics of Bis(trifluoromethyl)peroxide (BTMP) as Trifluoromethoxylation Agent

3.1 Xu and Tang's Methodology

Table S2. Calculation of ratio of total mass of substrate to reagents using Xu and Tang's trifluoromethoxylation methodology.[5]

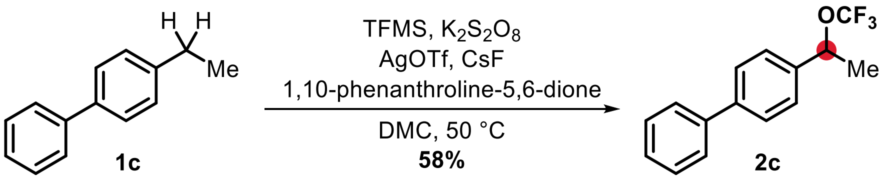

substrate	molar mass [g/mol]	equivalents	amount of substance [mmol]	mass [mg]
4-ethyl-1,1'-biphenyl	182.2660	1.00	0.500	91.1
**reagent**	**molar mass [g/mol]**	**equivalents**	**amount of substance [mmol]**	**mass [mg]**
TFMS	244.1596	5.00	2.50	610
AgOTf	256.9314	0.300	0.150	38.5
$K_2S_2O_8$	270.3086	3.00	1.50	405
1,10-phenanthroline-5,6-dione	210.1920	0.0500	0.0250	5.25
CsF	151.9039	4.00	2.00	304
Total				1362.75

ratio mass substrate/mass reagents = $\frac{\text{91.1 mg}}{\text{1362.75 mg}} \approx \frac{1}{15}$

TFMS = trifluoromethyl 4-fluorobenzenesulfonate

3.2 This Work using BTMP

Table S3. Calculation of ratio of total mass of substrate to reagents using BTMP as trifluoromethoxylation reagent.

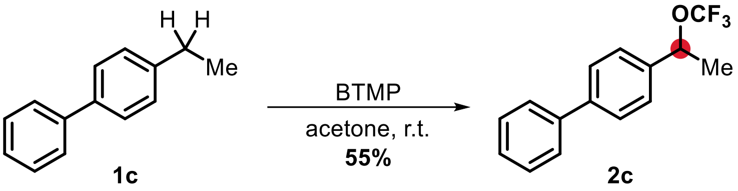

substrate	molar mass [g/mol]	equivalents	amount of substance [mmol]	mass [mg]
4-ethyl-1,1'-biphenyl	182.2660	1.00	0.500	91.1
**reagent**	**molar mass [g/mol]**	**equivalents**	**amount of substance [mmol]**	**mass [mg]**
BTMP	170.0104	2.00	1.00	170
Total				170

ratio mass substrate/mass reagent = $\frac{\mathbf{91.1\ mg}}{\mathbf{170\ mg}} \approx \frac{\mathbf{1}}{\mathbf{2}}$

BTMP = bis(trifluoromethyl)peroxide

4. Calculating the Amount of Bis(trifluoromethyl)peroxide (BTMP)

To control the amount of BTMP used in the reactions we used volume (V_L) and pressure (p_L) in the glass lines. First, it is necessary to determine the gas volume of the line using the following steps:

Evacuate the line ($p_L \approx 0$ bar).

Close all valves in order to stop evacuation but maintaining the vacuum.

Add a flask with a known volume (V_F) to the line. The flask contains air and thus has a known pressure of $p_F \approx 1$ bar = 100000 Pa.

Open the valve where the line and flask are connected.

Measure the pressure p_{L+F} in the line + flask (now having a bigger volume V_{L+F}).

To simplify the calculation, we assumed that BTMP behaves as an ideal gas.

Determining V_L:

$$p_F \cdot V_F = p_{L+F} \cdot V_{L+F}$$

$$V_L = V_{L+F} - V_F = \frac{p_F \cdot V_F}{p_{L+F}} - V_F = \frac{1.00\ \mathrm{bar}\ \cdot 0.310\ \mathrm{L}}{0.600\ \mathrm{bar}} - 0.310\ \mathrm{L} = 0.210\ \mathrm{L}$$

Pressure (p_L) needed for adding one equivalent of peroxide (n = 500 µmol):

$$p_L \cdot V_L = n \cdot R \cdot T$$

$$p_L = \frac{n \cdot R \cdot T}{V_L}$$

$$p_L = \frac{500\ \cdot 10^{-6}\ \mathrm{mol} \cdot 8.31\ \frac{\mathrm{J}}{\mathrm{mol} \cdot \mathrm{K}} \cdot 293\ \mathrm{K}}{0.210 \cdot 10^{-3}\ \mathrm{m^3}} = 5809\ \frac{\mathrm{J}}{\mathrm{m^3}} = 5809\ \mathrm{Pa} \approx 0.0600\ \mathrm{bar}$$

The amount of 500 µmol BTMP gas causes a pressure of 1.14 bar in the reaction flask ($V_{reaction\ flask}$ = 11 mL):

$$p_L \cdot V_L = p_{reaction\,flask} \cdot V_{reaction\,flask}$$

$$p_{reaction\,flask} = \frac{p_L \cdot V_L}{V_{reaction\,flask}}$$

$$p_{reaction\,flask} = \frac{0.0600\ \mathrm{bar} \cdot 0.210\ \mathrm{L}}{0.0110\ \mathrm{L}} = 1.14\ \mathrm{bar}$$

Thus, make sure to use flasks resisting overpressure.

5. Performing Trifluoromethoxylation – Handling the Gaseous Peroxide depicted using [^{13}C]-BTMP

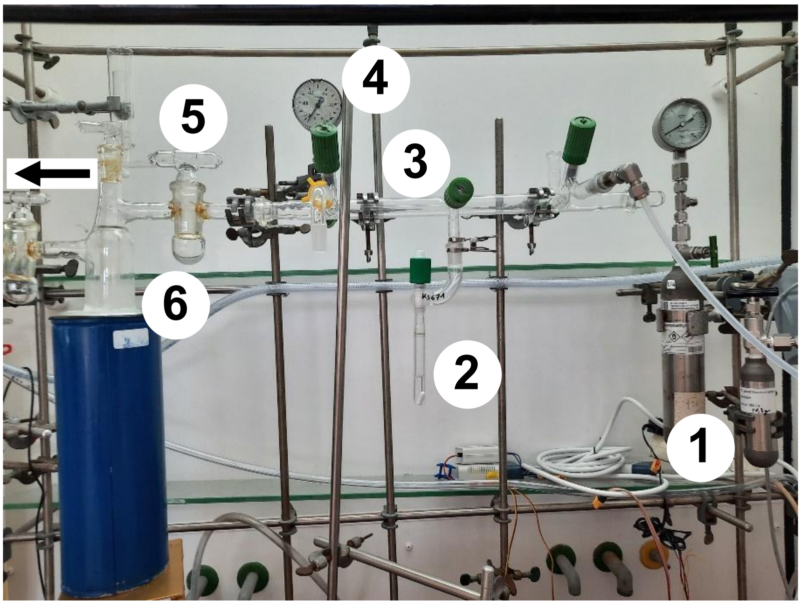

General setup for condensing ([^{13}C]-)BTMP into reaction flask.
1: Gas bottle containing ([^{13}C]-)BTMP
2: Reaction flask (Schlenk flask from RETTBERG); charged with a magnetic stir bar, substrate (and if necessary TBADT, and KF) dissolved in acetone
3: Gas line; all valves are closed
4: Barometer
5: Valve to open and close connection to vacuum pump
6: Cooling trap filled with liquid nitrogen
← connection to vacuum pump

First step: Freeze-Pump-Thaw

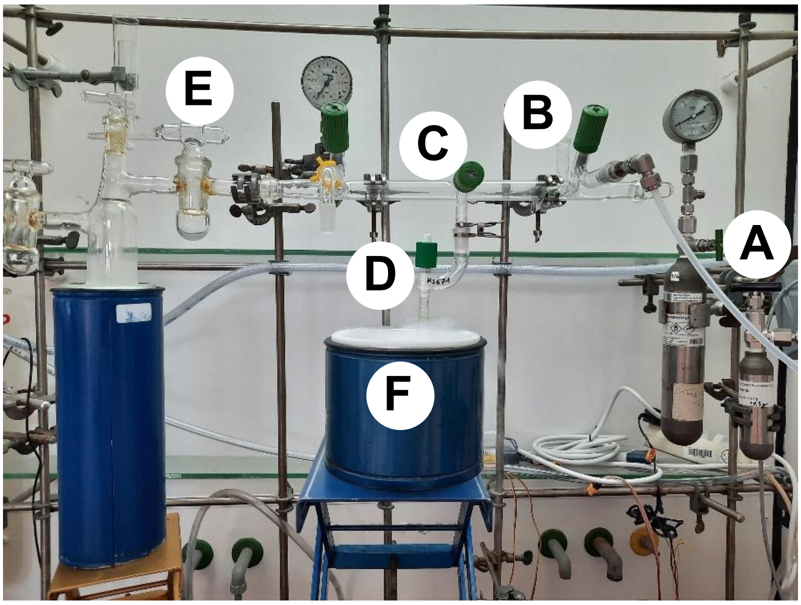

A: Valve closed
B: Valve opened
C: Valve opened
D: Valve closed
E: Valve opened
F: Dewar filled with liquid nitrogen

Freeze the solution until it is completely solid. Then, open valve **D**. Close it again after evacuating the RETTBERG tube, remove Dewar (**F**) and allow the solution to melt again. When the solution stops releasing gas and is liquid again, freeze it again. Repeat this process two times.

Second step: Condensing BTMP

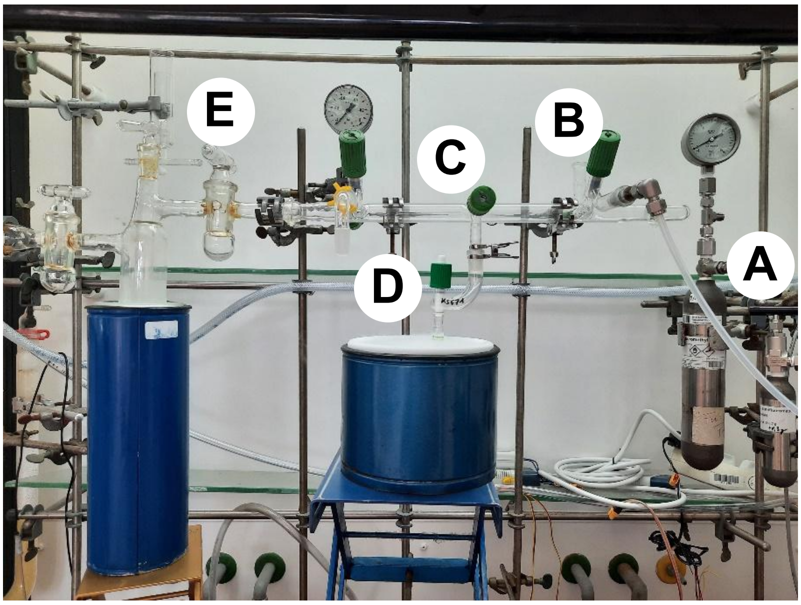

A: Valve closed

B: Valve opened

C: Valve opened

D: Valve closed

E: Valve closed

After two cycles of freeze-pump-thaw the solution stays frozen in liquid nitrogen. Now, valve **E** is closed. The line is evacuated. Valve **A** is slowly opened until the barometer shows a pressure difference Δp for the amount of BTMP needed (see calculations for determining Δp).

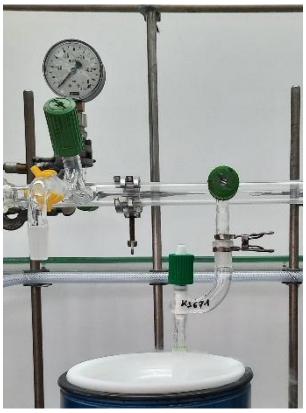

→

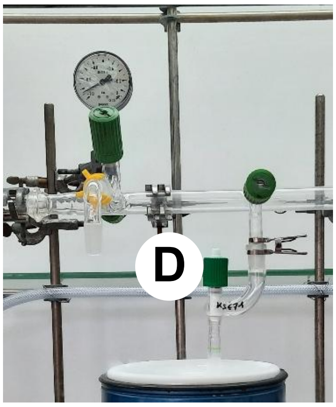

Δp ≈ 0.0600 bar; open valve **D**. BTMP is condensing into the Schlenk valve. Wait for around 30 s before closing valve **D** again (Liquid nitrogen stops boiling)

→

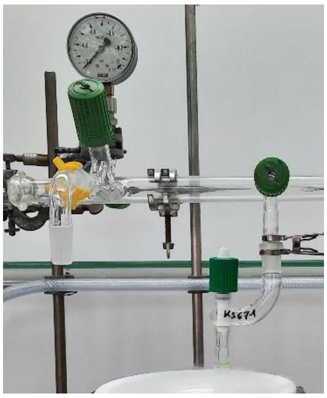

Close valve **D** again. Wait until the solution is completely melted.

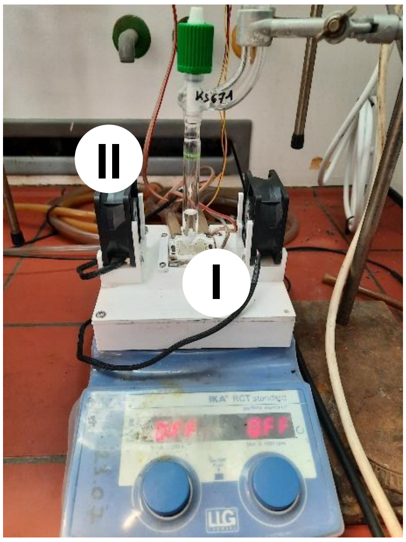

I: 365 nm LED Chip (30 W), cooled by water from the bottom.
II: Fans for keeping temperature constant
Place the reaction flask at a distance of 1–2 mm to the LED Chip.

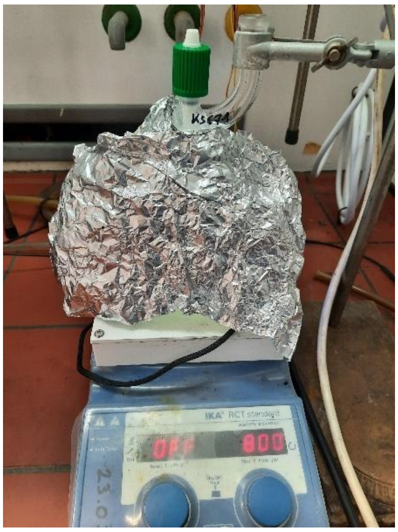

Wrap the apparatus in aluminum foil, switch on the light and stir the solution.

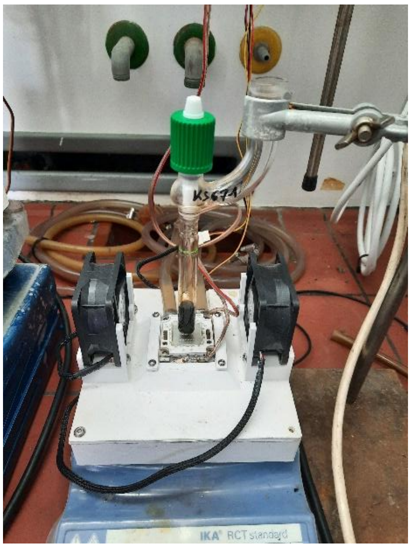

After stirring the clear reaction mixture turned dark brown.

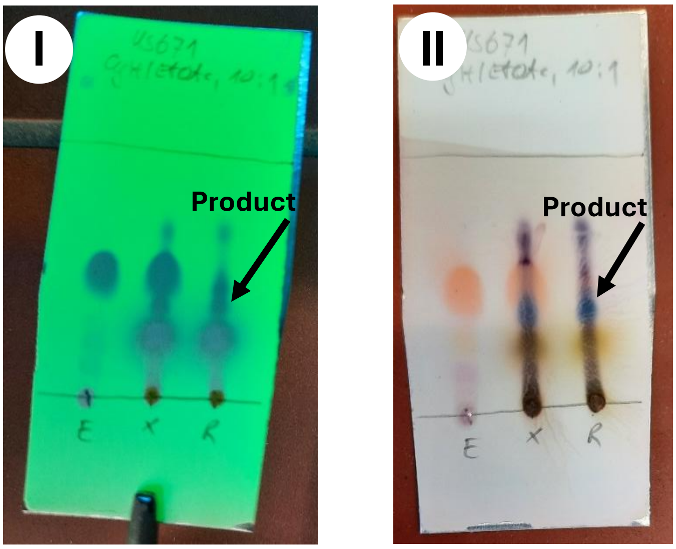

TLC of crude mixture was run with CyH/EtOAc = 10:1
I: TLC under UV light irradiation (254 nm)
II: TLC dyed with anisaldehyde stain
E = starting material
x = cross spot (staring material + reaction mixture)
R = reaction mixture

6. Optimization of the Trifluoromethoxylation of Benzylic C–H Bonds

Table S4. Optimization of trifluoromethoxylation of benzylic C–H bonds using ethylbenzene (1a) as substrate.

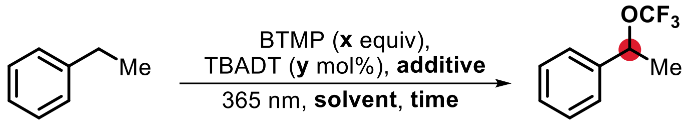

1a **2a**

entry	x	y (mol%)	additive	solvent	time [h]	result[a)]
**1**	2 equiv	5	CuCl (1.2 equiv)	MeCN (0.24 M)	18	5%
**2**	2 equiv	5	-	MeCN (0.24 M)	18	18%
**3**	2 equiv	10	-	MeCN (0.24 M)	18	18%
**4**	2 equiv	5	KF (1.0 equiv)	MeCN (0.24 M)	18	30%
**5**	2 equiv	5	-	MeCN/HFIP (10:1; 0.24 M)	18	23%
**6**	2 equiv	5	-	acetone (0.24 M)	18	55%
**7**	2 equiv	5	KF (1.0 equiv)	acetone (0.24 M)	18	29%
**8**	2 equiv	5	-	acetone/HFIP (10:1; 0.24 M)	18	34%
**9**	2 equiv	5	-	HFIP (0.24 M)	18	-
**10**	2 equiv	5	-	acetone/HFIP (10:1, 0.24 M)	18	41%
**11**	4 equiv	5	-	acetone (0.24 M)	18	38%
**12**	2 equiv	10	-	acetone (0.24 M)	18	31%
**13**	2 equiv	5	-	acetone/F_3CCH_2OH (10:1, 0.24 M)	18	38%
**14**	2 equiv	2	-	acetone (0.24 M)	18	33%
**15**	2 equiv	5	-	acetone/1 M HCl (2.5:1, 0.24 M)	18	23%
**16**	2 equiv	5	$NaHCO_3$ (0.1 equiv)	acetone (0.24 M)	18	34%
**17**	2 equiv	5	-	acetone (0.24 M)	18	26%
**18**	4 equiv	5	-	-	18	-
**19**	2 equiv	5	KF (1.0 equiv)	acetone/HFIP (10:1, 0.24 M)	18	32%
**20**	2 equiv	5	-	acetone (0.12 M)	18	28%
**21**	2 equiv	5	-	acetone (0.48 M)	18	46%
**22**	2 equiv	5	-	acetone (0.24 M)	18	23%
**23**	2 equiv	5	-	acetone (0.24 M)	18	28%
**24**	2 equiv	5	-	acetone (0.24 M)	18	40%

**25**	2 equiv	5	-	acetone/HFIP (0.24 M)	18	30%
**26**	2 equiv	5	-	acetone (0.24 M)	3	56%
**27**	2 equiv	5	-	acetone (0.24 M)	1	57%
**28**	2 equiv	5	-	acetone (0.24 M)	2	59%
**29**	2 equiv	5	-	acetone (0.24 M)	0.5	36%
**30**[b]	2 equiv	5	-	acetone (0.24 M)	2	27%
**31**[c]	2 equiv	5	-	acetone (0.24 M)	2	51%
**32**	2 equiv	5	-	MeCN (0.24 M)	2	12%
**33**	2 equiv	5	KF (1.0 equiv)	acetone (0.24 M)	2	57%
**34**	2 equiv	5	-	acetone/HFIP (10:1; 0.24 M)	2	28%
**35**	2 equiv	5	-	acetone (0.48 M)	2.5	38%
**36**	2 equiv	5	-	acetone (0.12 M)	2	27%
**37**	2 equiv	1	-	acetone (0.24 M)	2	30%
**38**	2 equiv	5	-	acetone (0.37 M)	2	-
**39**[d]	2 equiv	5	-	acetone (0.24 M)	2	32%
**40**	2 equiv	5	-	acetone (0.24 M)	2	60%
**41**	2 equiv	no	-	acetone (0.24 M)	4	66%
**42**	2 equiv	no	-	MeCN (0.24 M)	4	7%
**43**	2 equiv	no	-	acetone (0.24 M)	4	66%
**44**	2 equiv	5	no light	acetone (0.24 M)	4	traces
**45**	2 equiv	no	-	acetone (0.24 M)	2	53%

[a] ^{19}F NMR yield using α,α,α-trifluorotoluene as internal standard; [b] using sodium decatungstate (NaDT) instead of TBADT; [c] reaction was performed with 1.00 mmol ethylbenzene (1a) in a 35 mL RETTBERG Schlenk tube; [d] RETTBERG Schlenk tube had a distance of 2 cm from the LED Chip (instead of 1–2 mm)

Table S5. Trifluoromethoxylation of benzylic C–H bonds using 4-ethyl-1,1'-biphenyl (1c) as substrate and other photocatalysts than TBADT.

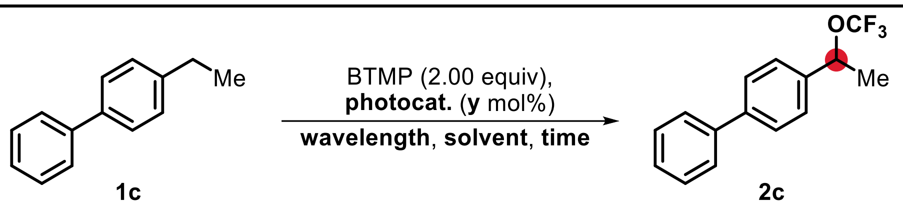

1c **2c**

entry	photocat.	y (mol%)	wavelength [nm]	solvent	time [h]	result[a]
**1**	benzophenone	5	365	MeCN (0.24 M)	2	10%
**2**	benzophenone	5	365	acetone (0.24 M)	2	38%
**3**	benzophenone	5	365	MeCN (0.24 M)	3	8%

**4**	benzophenone	5	365	acetone (0.24 M)	18	24%
**5**	benzophenone	100	365	acetone (0.24 M)	18	21%
**6**	9-mesityl-10-methylacridinium Perchlorate; Pyridine *N*-Oxide	5; 50	455	MeCN (0.10 M)	18	29%

a) ^{19}F NMR yield using α,α,α-trifluorotoluene as internal standard

7. Optimization of the Trifluoromethoxylation of Non-Activated C–H Bonds

Table S6. Optimization of trifluoromethoxylation of non-activated C–H bonds using (3a*R*)-(+)-sclareolide (3f) as substrate.

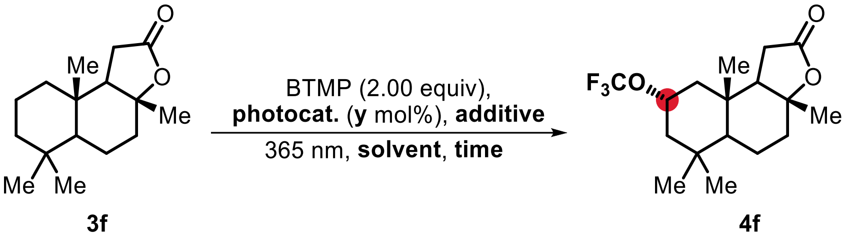

entry	photocat.	y (mol%)	additive	solvent	time [h]	result[a)]
**1**	TBADT	5	KF (1.0 equiv)	acetone (0.72 M)	2	10%
**2**	TBADT	5	KF (1.0 equiv)	acetone (0.48 M)	2	25%
**3**	TBADT	5	-	acetone (0.24 M)	3	18%
**4**	TBADT	5	-	acetone (0.24 M)	18	16%
**5**	TBADT	5	-	MeCN (0.24 M)	18	<5%
**6**	TBADT	5	-	acetone (0.48 M)	18	10%
**7**	benzophenone	5	-	MeCN (0.1 M)	19	17%
**8**	benzophenone	5	-	MeCN (0.1 M)	3	<5%
**9**	benzophenone	5	-	acetone (0.1 M)	3	<5%
**10**	benzophenone	5	-	acetone (0.1 M)	20	<5%
**11**	benzophenone	100	-	acetone (0.1 M)	2	<5%
**12**	benzophenone	100	-	MeCN (0.1 M)	18	16%
**13**	benzil	5	-	MeCN (0.1 M)	22	<5%
**14**	fluorenone	5	-	MeCN (0.1 M)	18	5%
**15**	acetophenone	5	-	MeCN (0.1 M)	18	<5%

a) ^{19}F NMR yield using α,α,α-trifluorotoluene as internal standard

8. Reaction Mechanism

8.1 Determination of the Photon Flux of the LED Chip

According to the procedure of Yoon,[6] the *photon flux* of the LED (λ_{max} = 365 nm) was determined by standard ferrioxalate actinometry. A 0.006 M solution of ferrioxalate was prepared by dissolving potassium ferrioxalate hydrate (29.5 mg) in H_2SO_4 (10 mL of a 0.05 M solution). A buffered solution of 1,10-phenanthroline was prepared by dissolving 1,10-phenanthroline (25.0 mg) and sodium acetate (5.63 g) in H_2SO_4 (25 mL of a 0.5 M solution). Both solutions were stored in the dark. To determine the *photon flux* of the LED, the ferrioxalate solution (1.0 mL) was placed in a Rettberg tube and irradiated for 90 seconds at λ_{max} = 365 nm using the LED chip. After irradiation, the phenanthroline solution (0.175 mL) was added to the Rettberg tube and the mixture was allowed to stir in the dark for 1 h to grant the ferrous ions to completely coordinate to the phenanthroline. The absorbance of the solution was measured at 510 nm. A non-irradiated sample was also prepared and the absorbance at 510 nm was measured. Conversion was calculated using eq. 1.

$$\text{mol}\, Fe^{2+} = \frac{V \cdot \Delta A(510\ \text{nm})}{l \cdot \varepsilon} \qquad \text{(eq. 1)}$$

where *V* is the total volume (0.001175 L) of the solution after addition of phenanthroline, *ΔA* is the difference in absorbance at 510 nm between the irradiated and non-irradiated solutions, *l* is the path length (1.00 cm), and ε is the molar absorptivity of the ferrioxalate actinometer at 510 nm ($11000\ \frac{\text{L}}{\text{mol·cm}}$). The photon flux can be calculated using eq. 2.

$$Photon\ Flux = \frac{\text{mol}\, Fe^{2+}}{\Phi \cdot t \cdot f} \qquad \text{(eq. 2)}$$

where *Φ* is the quantum yield for the ferrioxalate actinometer (1.25 at λ_{ex} = 358 nm), *t* is the irradiation time (90 s), and *f* is the fraction of light absorbed at λ_{ex} = 365 nm by the ferrioxalate actinometer. This value is calculated using eq. 3

$$f = 1 - 10^{-A(365\ \text{nm})} \qquad \text{(eq. 3)}$$

where A(365 nm) is the absorbance of the ferrioxalate solution at 365 nm.

The photon flux was thus calculated to be $2.38 \times 10^{-9}\ \frac{\text{Einstein}}{\text{s}}$.

8.2 Determination of the Reaction Quantum Yield (Φ)

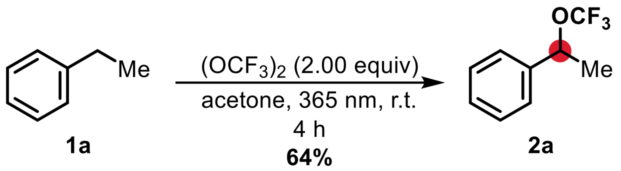

A RETTBERG Schlenk tube was charged with a stir bar and a solution of **1a** (53.0 mg, 61.2 µL, 500 µmol, 1.00 equiv) in acetone (2.1 mL). The suspension was degassed twice by Freeze-Pump-Thaw procedure. Then, BTMP (169 mg, 1.00 mmol, 2.00 equiv) was condensed into the cooled (liquid nitrogen) solution, the flask was closed, and the solution was allowed to melt and warm up to r.t. The reaction mixture was irradiated with UV-light (365 nm, 30 W LED Chip) for 4 h (14400 s). The flask was opened slowly to release the overpressure. The yield was determined by ^{19}F NMR analysis (64%, 3.2×10^{-4} mol of **2a**).

The reaction quantum yield (Φ) was determined using eq. 4 where the *photon flux* is $2.38 \times 10^{-9}\ \frac{\text{Einstein}}{\text{s}}$ (determined by actinometry as described above), *t* is the reaction time (14400 s) and *f* is the fraction of incident light absorbed by the reaction mixture, determined using eq. 3.

$$\Phi = \frac{\text{mol}\ of\ product\ formed}{Photon\ flux \cdot t \cdot f} \qquad \text{(eq. 4)}$$

$$\Phi = \frac{3.2 \cdot 10^{-4}\ \text{mol}}{2.38 \times 10^{-9}\ \frac{\text{Einstein}}{\text{s}} \cdot 14400\ \text{s} \cdot 0.999}$$

$$\Phi = 9.3$$

The reaction quantum yield (Φ) was thus determined to be 9.3.

^{19}F NMR yield after 2 h: 53% (≡ 0.265 mmol) → Quantum yield after 2 h (= 7200 s) Φ = 15.3.

This result indicates that one photon enables the formation of at least 9 molecules of (1-(trifluoromethoxy)ethyl)benzene (**2a**, neglecting quenching processes). As there is

no measurable absorption at the beginning (see UV/Vis data), the radical chain must even be longer.

8.3 EPR Spectra, UV/Vis Spectra, HRMS Data after irradiation of Acetone mixed with BTMP

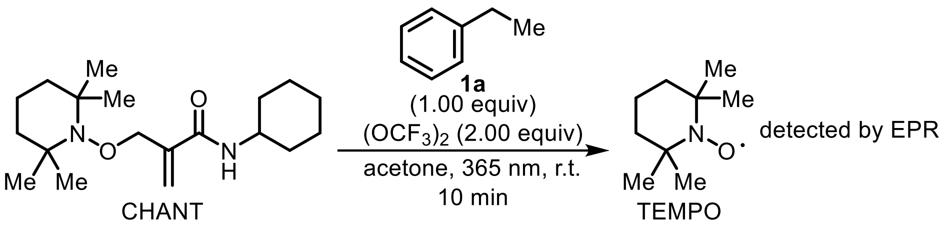

Ethylbenzene (0.24 M), and *N*-cyclohexyl-2-(((2,2,6,6-tetramethylpiperidin-1-yl)oxy)methyl)acrylamide (CHANT) (synthesized according to P. J. H. Williams, G. A. Boustead, D. E. Heard, P. W. Seakins, A. R. Rickard, V. Chechik, *J. Am. Chem. Soc.*, 2022, **144**, 15969–15976) were dissolved in acetone in a YOUNG EPR-tube. BTMP was condensed into the tube and the reaction mixture was irradiated with UV-light (365 nm). Then, the EPR spectra was measured and showed characteristic signals of (2,2,6,6-Tetramethylpiperidin-1-yl)oxyl (TEMPO) radicals (A. I. Smirnov, T. I. Smirnova, P. D. Morse, *Biophys. J.*, 1995, **68**, 2350–2360).

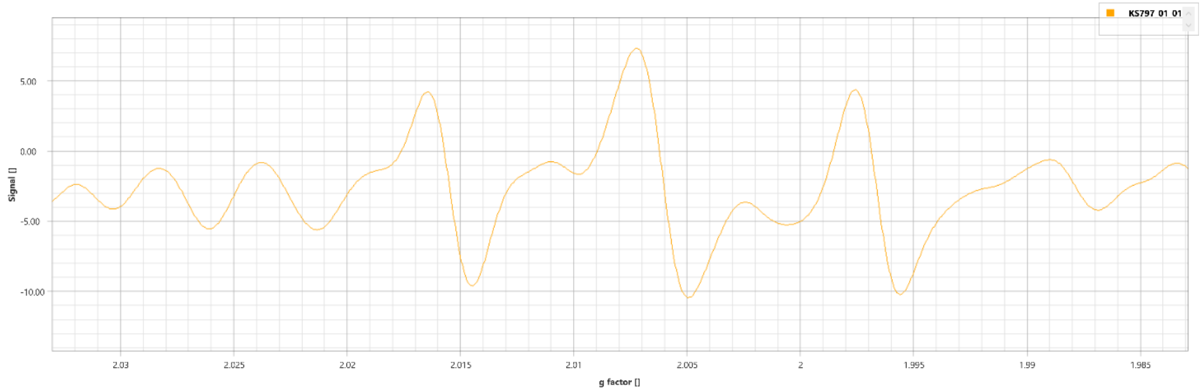

Figure S1. EPR Spectra of a solution of ethylbenzene (0.24 M), BTMP, *N*-cyclohexyl-2-(((2,2,6,6-tetramethylpiperidin-1-yl)oxy)methyl)acrylamide (CHANT) in acetone after irradiation with UV-light (365 nm).

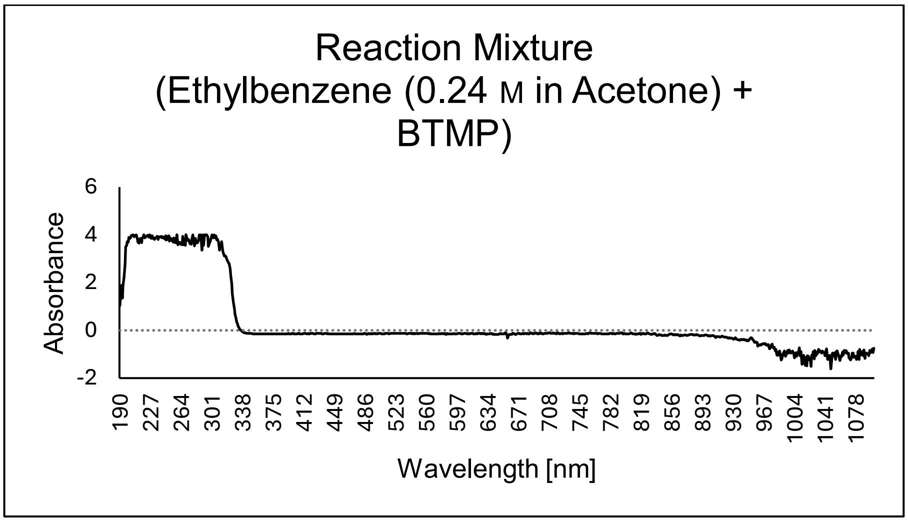

Figure S2. UV/Vis spectra of the reaction mixture.

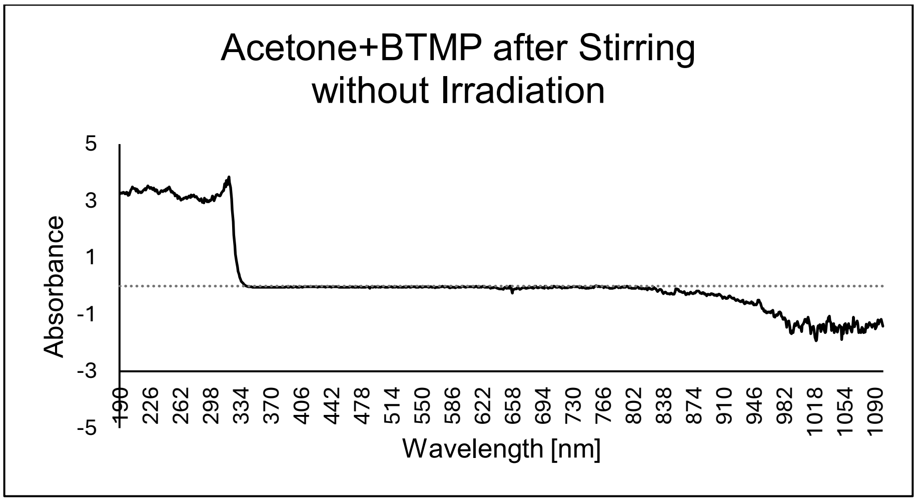

Figure S3. UV/Vis spectra of acetone and BTMP after stirring for 4 h without irradiation.

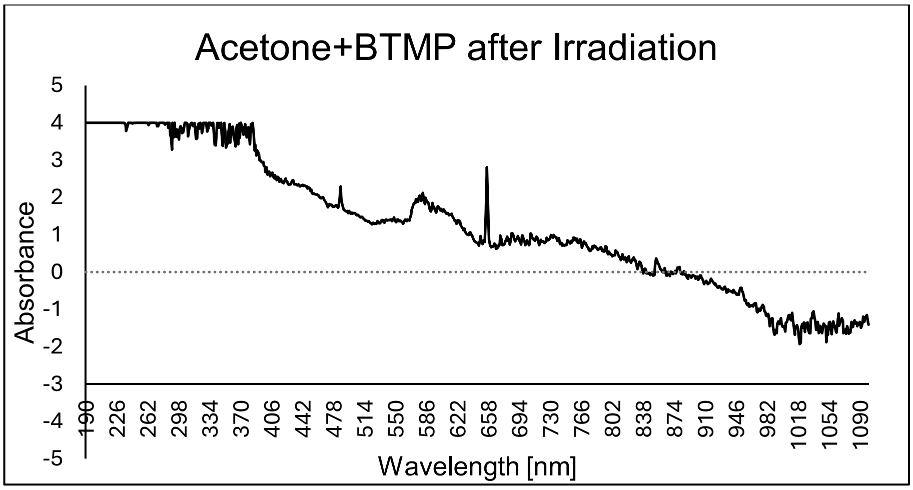

Figure S4. UV/Vis spectra of acetone and BTMP after irradiation with 365 nm wavelength for 4 h.

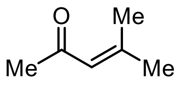

mesityl oxide

HRMS (ESI): *m/z* calculated for $C_6H_{10}ONa^+$ [M+Na]$^+$: 121.0624; found: 121.0629.

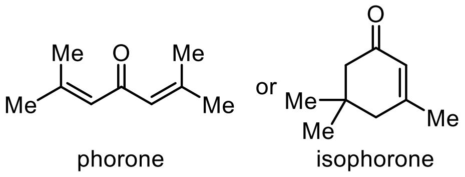

HRMS (ESI): *m/z* calculated for $C_9H_{14}ONa^+$ [M+Na]$^+$: 161.0937; found: 161.0942.

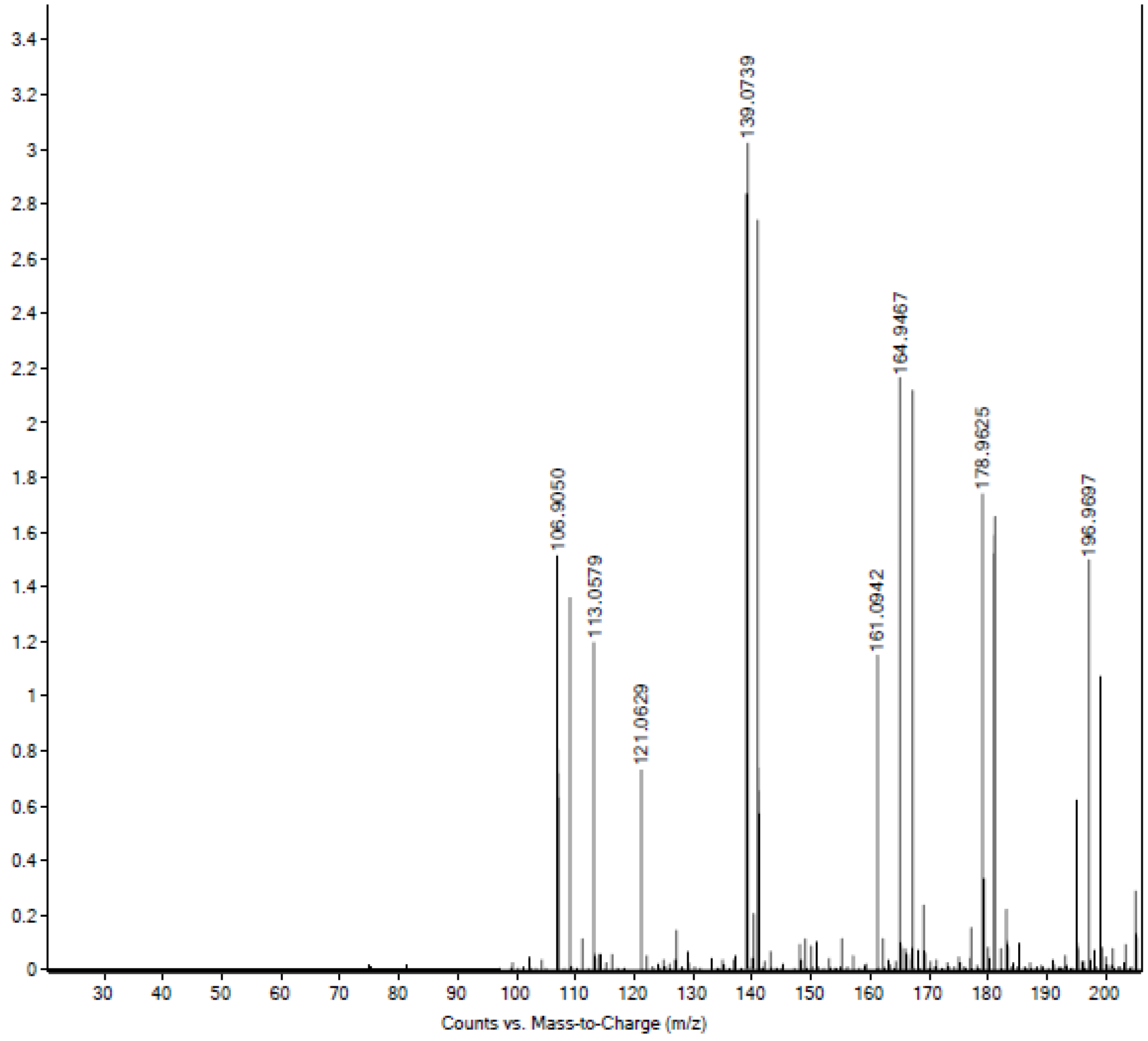

Figure S5. HRMS (ESI, pos.) of the acetone and BTMP after irradiation for 4 h.

When only irradiating acetone with 365 nm, no formation of those species was observed. Thus, BTMP seems to accelerate or enable the formation of those condensation products.

8.4 Proposed Reaction Mechanism for Trilfuoromethoxylation in the absence of TBADT

Trifluoromethoxylation of ethylbenzene (**1a**) was achieved in acetone without using any catalyst (66% yield). This transformation was only possible in 7% yield using MeCN as solvent (table S4, entry 42). This indicates an important role of acetone in the reaction mechanism. Reaction quantum yield experiment leads to a quantum yield of $\Phi = 9.3$. Thus, the mechanism proceeds probably via a chain reaction. Furthermore, radical formation is indicated by EPR experiment. A plausible mechanism for trifluoromethoxylation of benzylic C–H bonds is shown in scheme S1.

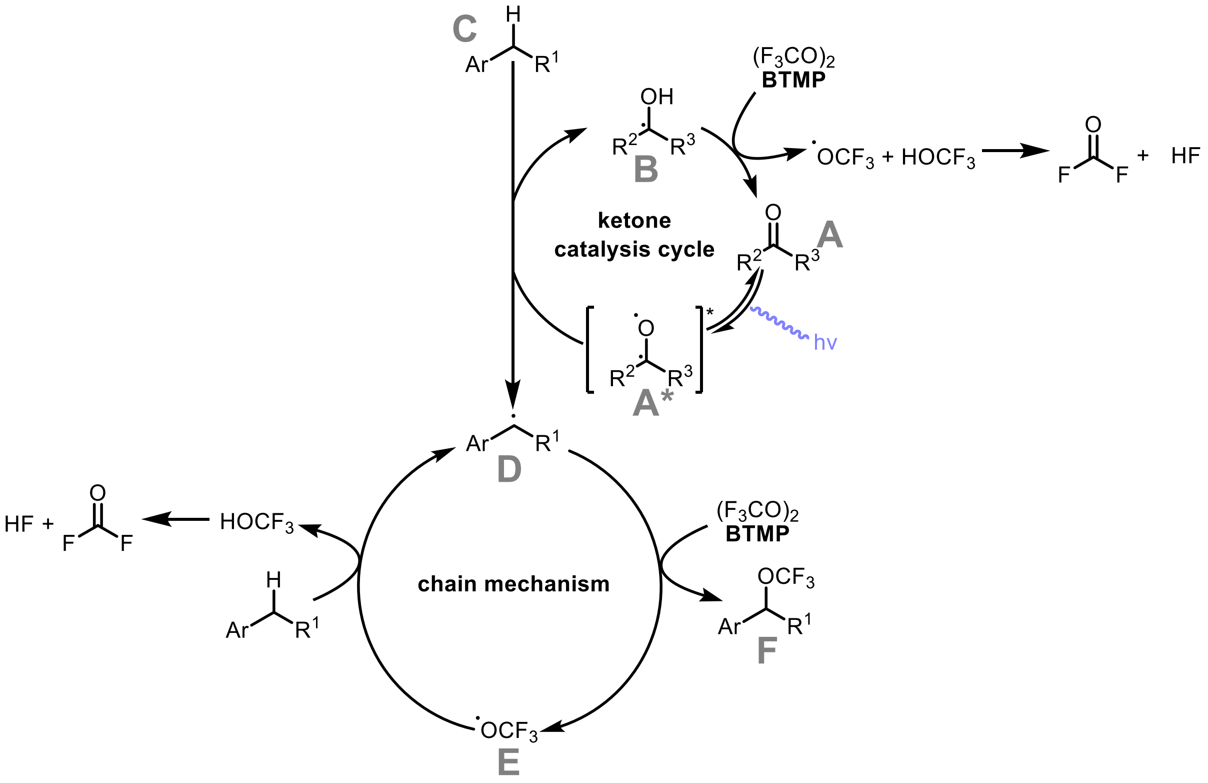

Scheme S1. Plausible reaction mechanism for trifluoromethoxylation in the absence of TBADT.

A → A*: *Photo excitation* of ketone. Either traces of mesityl oxide or phorone or acetone itself. Tetramethyldioxetane was found in HRMS. **HRMS (ESI):** *m/z* calculated for $C_6H_{12}O_2Na^+$ $[M+Na]^+$: 139.0729; found: 139.0733. Might be generated by [2+2]-cycloaddition of acetone.

See: L. Li, X. Mu, W. Liu, Y. Wang, Z. Mi, C.-J. Li, *J. Am. Chem. Soc.*, 2016, **138**, 5809–5812.

C → D: *HAT* by A* (H. Cao, X. Tang, H. Tang, Y. Yuan, J. Wu, *Chem. Catal.*, 2021, **1**, 523–598. L. Li, X. Mu, W. Liu, Y. Wang, Z. Mi, C.-J. Li, *J. Am. Chem. Soc.*, 2016, **138**, 5809–5812.).

B → A: *Oxidative quenching*. HAT from B to BTMP; BTMP undergoes mesolytic cleavage to $^{\cdot}OCF_3$ and $HOCF_3$. $HOCF_3$ decomposes to fluorophosgene and HF (S. Dix, P. Golz, J. R. Schmid, S. Riedel, M. N. Hopkinson, *Chem. Eur. J.*, 2021, **27**, 11554–11558).

D → F: *Chain elongation*. Benzylic radical attacks BTMP generating trifluoromethoxylated product and $^{\cdot}OCF_3$ (E), which reacts with R–H further to $HOCF_3$ via HAT. (Compare reaction quantum yield experiment indicating a chain mechanism.)

8.5 Proposed Reaction Mechanism for Trilfuoromethoxylation in the absence of TBADT

Based on the mechanism above and common TBADT-catalysis cycles (D. Ravelli, M. Fagnoni, T. Fukuyama, T. Nishikawa, I. Ryu, ACS Catal., 2018, 8, 701 – 713), the following mechanism is proposed as plausible for trifluoromethoxylation using TBADT.

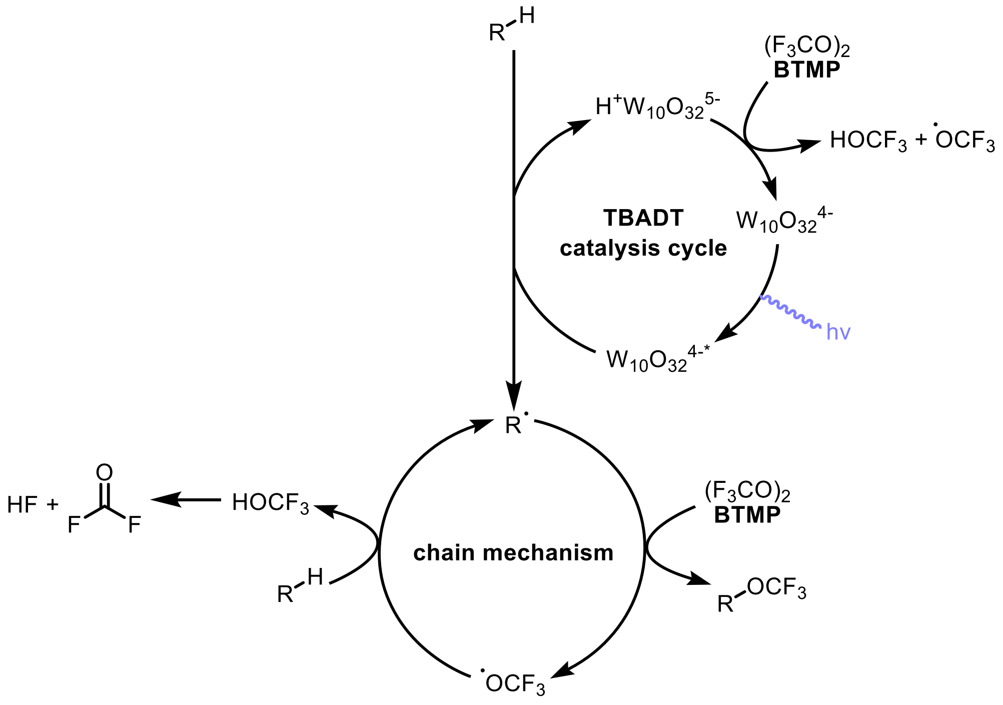

Scheme S2. Plausible reaction mechanism for trifluoromethoxylation using TBADT.

9. General Procedures

9.1 General Procedure for Suzuki Coupling (GP1)

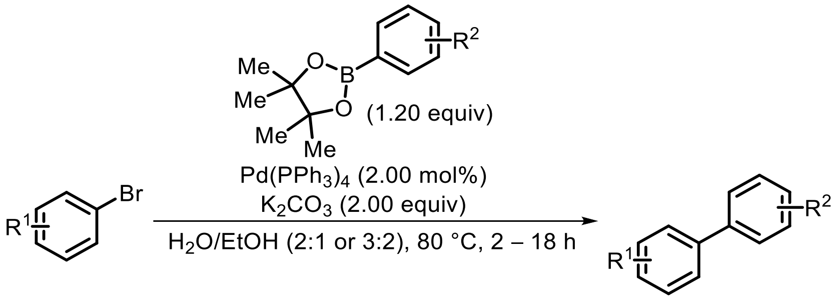

To a two-necked round-bottom flask with a cooler was added the bromobenzene derivative (1.00 equiv), phenylboronic acid (1.20 equiv), $Pd(PPh_3)_4$ (2.00 mol%), and K_2CO_3 (2.00 equiv) in a mixture of water and EtOH (2:1 or 3:2, argon was bubbled through these solvents). The mixture was heated to 80 °C. After 2–18 h the reaction mixture was cooled to r.t. and filtered through a plug of Celite®, and rinsed with EtOAc. The aqueous phase was separated and extracted four times with EtOAc. The combined organic phases were dried over $MgSO_4$, filtered, and the solvent was evaporated under reduced pressure. The product was purified by flash column chromatography.

9.2 General Procedure for the Acetylation of Alcohols 2j and 2k (GP2)

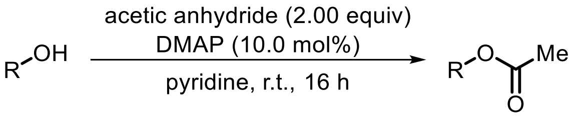

To a round-bottom flask was added the alcohol substrate (1.00 equiv), acetic anhydride (2.00 equiv), 4-dimethylaminopyridine (DMAP, 10.0 mol%), and pyridine. The reaction was stirred for 16 h at r.t., then slowly quenched with $NaHCO_3$ (aq., sat., 50 mL). The mixture was extracted with Et_2O (3 × 50 mL). The organic extracts were combined and washed with $CuSO_4$ (aq., sat., 50 mL), H_2O (50 mL), NaCl (aq., sat., 50 mL). The organic layer was dried over Na_2SO_4, filtered and concentrated. The residue was purified by column chromatography to afford the product.

9.3 General Procedure for Trifluoromethoxylation of Benzylic C–H Bonds (GP3)

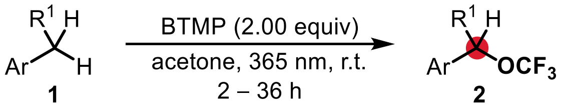

A RETTBERG Schlenk tube was charged with a stir bar and a solution of substrat **1** (1.00 equiv) in acetone (0.24 M). The suspension was degassed twice by Freeze-

Pump-Thaw procedure. Then, BTMP (2.00 equiv) was condensed into the cooled (liquid nitrogen) solution, the flask was closed, and the solution was allowed to melt and warm up to r.t. The reaction mixture was irradiated with UV-light (365 nm, 30 W LED Chip) for 2–36 h. The flask was opened slowly to release the overpressure. Then, the solvent was directly evaporated under reduced pressure and product **2** was purified by flash column chromatography.

(After irradiation, 0.1 mL of the reaction mixture were transferred into an NMR tube to prepare the sample for crude ^{19}F NMR yield calculation. Additionally, 0.1 mL of a solution of α,α,α-trifluorotoluene in $CDCl_3$ (0.24 M) was added to the NMR tube.)

9.4 General Procedure for Trifluoromethoxylation of Non-activated C–H Bonds (GP4)

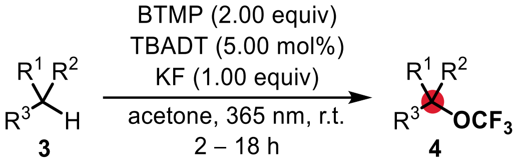

A RETTBERG Schlenk tube was charged with a stir bar, TBADT (5.00 mol%), KF (1.00 equiv), and a solution of substrate **3** (1.00 equiv) in acetone (0.48 M). The suspension was degassed twice by Freeze-Pump-Thaw procedure. Then, BTMP (2.00 equiv) was condensed into the cooled (liquid nitrogen) solution, the flask was closed, and the solution was allowed to melt and warm up to r.t. The reaction mixture was irradiated with UV-light (365 nm, 30 W LED Chip) for 2–18 h. The flask was opened slowly to release the overpressure. Then, the solvent was directly evaporated under reduced pressure and product **4** was purified by flash column chromatography.

(After irradiation, 0.1 mL of the reaction mixture were transferred into a small glass vial, $CDCl_3$ was added, and the suspension was filtered into an NMR tube to prepare the sample for crude ^{19}F NMR yield calculation. Additionally, 0.1 mL of a solution of α,α,α-trifluorotoluene in $CDCl_3$ (0.48 M) was added to the NMR tube.)

9.5 General Procedure for Trifluoromethoxylation of Aldehydic C–H Bonds (GP5)

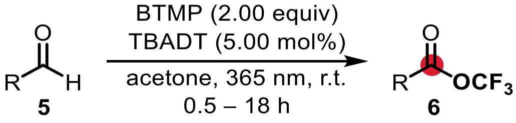

A RETTBERG Schlenk tube was charged with a stir bar, TBADT (5.00 mol%), and a solution of substrate **5** (1.00 equiv) in acetone (0.24 M). The suspension was degassed twice by Freeze-Pump-Thaw procedure. Then, BTMP (2.00 equiv) was condensed into the cooled (liquid nitrogen) solution, the flask was closed, and the solution was allowed to melt and warm up to r.t. The reaction mixture was irradiated with UV-light (365 nm, 30 W LED Chip) for 0.5–18 h. The flask was opened slowly to release the overpressure. Then, the solvent was directly evaporated and product **6** was purified by flash column chromatography.

(After irradiation, 0.1 mL of the reaction mixture were transferred into a small glass vial, $CDCl_3$ was added, and the suspension was filtered into an NMR tube to prepare the sample for crude ^{19}F NMR yield calculation. Additionally, 0.1 mL of a solution of α,α,α-trifluorotoluene in $CDCl_3$ (0.24 M) was added to the NMR tube.)

10. Synthesis of Starting Materials

10.1 4-Methyl-1,1'-biphenyl (1b)

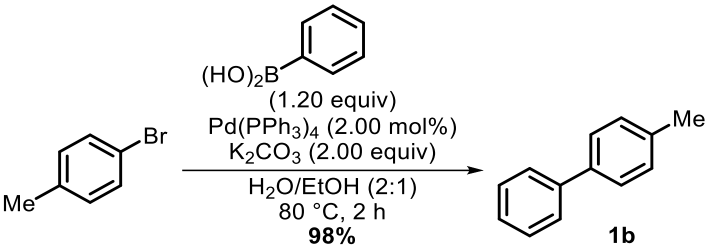

1b

4-Methyl-1,1’-biphenyl (**1b**) was prepared from 4-bromotoluene (500 mg, 2.97 mmol, 1.00 equiv), phenylboronic acid (434 mg, 3.57 mmol, 1.20 equiv), $Pd(PPh_3)_4$ (69.0 mg, 60.0 µmol, 2.00 mol%), K_2CO_3 (821 mg, 5.94 mmol, 2.00 equiv), H_2O (4.4 mL), and EtOH (2.2 mL) according to **GP1**. The reaction mixture was stirred at 80 °C for 2 h. Product **1b** (494 mg, 2.94 mmol, 98%) was purified by flash column chromatography (SiO_2, CyH) and isolated as a colorless solid.

^{1}H NMR (500 MHz, $CDCl_3$): δ = 7.62 – 7.59 (m, 2H), 7.53 – 7.50 (m, 2H), 7.47 – 7.43 (m, 2H), 7.40 – 7.29 (m, 1H), 7.27 (d, *J* = 7.9 Hz, 2H), 2.42 (s, 3H) ppm.
^{13}C{^{1}H} NMR (126 MHz, $CDCl_3$): δ = 141.5, 138.7, 137.4, 129.8, 129.1, 129.1, 127.3, 127.3, 21.4 ppm.

The spectroscopic data are consistent with those reported in literature.[7]

10.2 4-Ethyl-1,1'-biphenyl (1c)

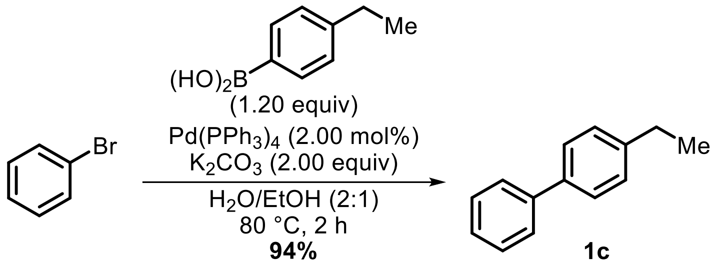

1c

4-Ethyl-1,1’-biphenyl (**1c**) was prepared from bromobenzene (2.00 g, 12.7 mmol, 1.00 equiv), (4-ethylphenyl)boronic acid (2.29 g, 15.3 mmol, 1.20 equiv), $Pd(PPh_3)_4$ (300 mg, 255 µmol, 2.00 mol%), K_2CO_3 (3.52 g, 25.5 mmol, 2.00 equiv), H_2O (17.6 mL), and EtOH (8.8 mL) according to **GP1**. The reaction mixture was stirred at 80 °C for 2 h. The product **1c** (2.18 g, 12.0 mmol, 94%) was purified by flash column chromatography (SiO_2, *n*-pentane) and isolated as a colorless solid.

^{1}H NMR (600 MHz, $CDCl_3$): δ = 7.59 (dt, *J* = 8.1, 1.7 Hz, 2H), 7.54 – 7.52 (m, 2H), 7.45 – 7.41 (m, 2H), 7.33 (tt, *J* = 7.0, 1.1 Hz, 1H), 7.30 – 7.27 (m, 2H), 2.71 (q, *J* = 7.6 Hz, 2H), 1.29 (t, *J* = 7.6 Hz, 3H) ppm.
^{13}C{^{1}H} NMR (151 MHz, $CDCl_3$): δ = 143.7, 141.5, 139.0, 129.0, 128.6, 127.4, 127.4, 127.3, 28.9, 15.9 ppm.

The spectroscopic data are consistent with those reported in literature.[8]

10.3 4-Isopropyl-1,1'-biphenyl (1d)

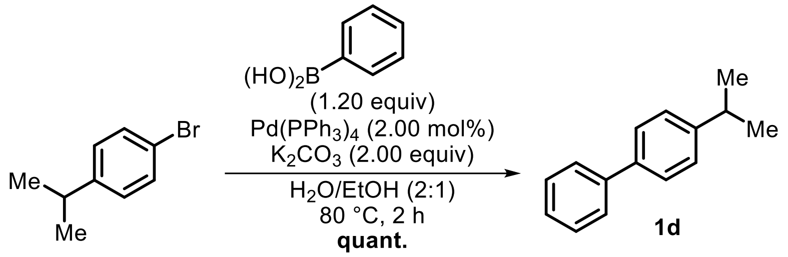

1d

4-Isopropyl-1,1'-biphenyl (**1d**) was prepared from 4-bromocumene (500 mg, 2.51 mmol, 1.00 equiv), phenylboronic acid (367 mg, 3.01 mmol, 1.20 equiv), $Pd(PPh_3)_4$ (58.0 mg, 50.0 µmol, 2.00 mol%), K_2CO_3 (694 mg, 5.02 mmol, 2.00 equiv), H_2O (4.40 mL), and EtOH (2.20 mL) according to **GP1**. The reaction mixture was stirred at 80 °C for 2 h. Product **1d** (492 mg, 2.51 mmol, quant.) was purified by flash column chromatography (SiO_2, CyH) and isolated as colorless liquid which solidifies in the fridge at 0 °C.

^{1}H NMR (400 MHz, $CDCl_3$): δ = 7.61 (ddd, *J* = 8.0, 4.0, 1.3 Hz, 2H), 7.57 – 7.53 (m, 2H), 7.48 – 7.43 (m, 2H), 7.37 – 7.31 (m, 3H), 2.98 (hept, *J* = 6.9 Hz, 1H), 1.32 (d, *J* = 6.9 Hz, 6H) ppm.
^{13}C{^{1}H} NMR (176 MHz, $CDCl_3$): δ = 148.3, 141.5, 139.1, 129.0, 127.4, 127.4, 127.3, 127.2, 34.1, 24.4 ppm.

The spectroscopic data are consistent with those reported in literature.[9]

10.4 4-Ethyl-4'-methyl-1,1'-biphenyl (1e)

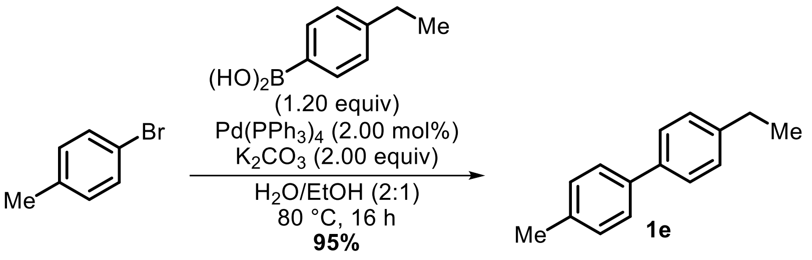

4-Ethyl-4'-methyl-1,1'-biphenyl (**1e**) was prepared from 1-bromo-4-methylbenzene (430 mg, 2.51 mmol, 1.00 equiv), (4-ethylphenyl)boronic acid (452 mg, 3.01 mmol, 1.20 equiv), $Pd(PPh_3)_4$ (58.0 mg, 50.0 µmol, 2.00 mol%), K_2CO_3 (694 mg, 5.02 mmol, 2.00 equiv), H_2O (4.40 mL), and EtOH (2.20 mL) according to **GP1**. The reaction mixture was stirred at 80 °C for 16 h. Product **1e** (468 mg, 2.38 mmol, 95%) was purified by flash column chromatography (SiO_2, CyH) and isolated as colorless solid.

^{1}H NMR (400 MHz, $CDCl_3$): δ = 7.52 (tt, *J* = 8.5, 2.0 Hz, 4H), 7.33 – 7.22 (m, 4H), 2.72 (q, *J* = 7.6 Hz, 2H), 2.42 (s, 3H), 1.30 (t, *J* = 7.6 Hz, 3H) ppm.
^{13}C NMR (176 MHz, $CDCl_3$): δ = 143.2, 138.7, 138.5, 136.8, 129.6, 128.4, 127.0, 127.0, 28.7, 21.2, 15.7 ppm.

The spectroscopic data are consistent with those reported in literature.[10]

10.5 4-Ethyl-4'-isopropyl-1,1'-biphenyl (1f)

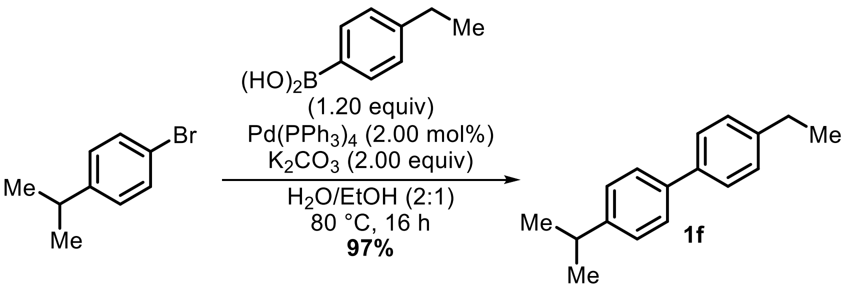

4-Ethyl-4'-isopropyl-1,1'-biphenyl (**1f**) was prepared from 1-bromocumene (500 mg, 2.51 mmol, 1.00 equiv), (4-ethylphenyl)boronic acid (452 mg, 3.01 mmol, 1.20 equiv), $Pd(PPh_3)_4$ (58.0 mg, 50.0 µmol, 2.00 mol%), K_2CO_3 (694 mg, 5.02 mmol, 2.00 equiv), H_2O (4.40 mL), and EtOH (2.20 mL) according to **GP1**. The reaction mixture was stirred at 80 °C for 16 h. Product **1f** (548 mg, 2.44 mmol, 97%) was purified by flash column chromatography (SiO_2, CyH) and isolated as colorless solid.

^{1}H NMR (400 MHz, $CDCl_3$): δ = 7.56 – 7.52 (m, 4H), 7.34 – 7.27 (m, 4H), 2.98 (sept, *J* = 7.0 Hz, 1H), 2.72 (q, *J* = 7.6 Hz, 2H), 1.32 (d, *J* = 6.9 Hz, 6H), 1.30 (t, *J* = 7.7 Hz, 3H) ppm.
^{13}C NMR (176 MHz, $CDCl_3$): δ = 147.8, 143.2, 138.9, 138.7, 128.3, 127.1, 126.9, 33.9, 28.7, 24.2, 15.7 ppm.

The spectroscopic data are consistent with those reported in literature.[11]

10.6 4-Ethyl-4'-fluoro-1,1'-biphenyl (1h)

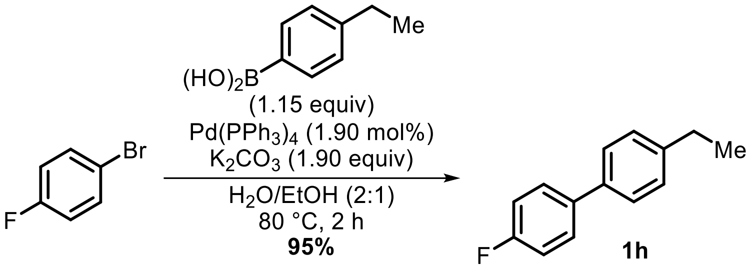

4-Ethyl-4'-fluoro-1,1'-biphenyl (**1h**) was prepared from 1-bromo-4-fluorobenzene (314 µL, 500 mg, 2.86 mmol, 1.00 equiv), (4-ethylphenyl)boronic acid (490 mg, 3.27 mmol, 1.15 equiv), $Pd(PPh_3)_4$ (63.0 mg, 55.0 µmol, 1.90 mol%), K_2CO_3 (753 mg, 5.46 mmol, 1.90 equiv), H_2O (4.40 mL), and EtOH (2.20 mL) according to **GP1**. The reaction mixture was stirred at 80 °C for 2 h. Product **1h** (546 mg, 2.73 mmol, 95%) was purified by flash column chromatography (SiO_2, *n*-pentane/Et_2O, 20:1) and isolated as colorless liquid which solidifies in the fridge at 0 °C.

^{1}H NMR (700 MHz, $CDCl_3$): δ = 7.56 – 7.52 (m, 2H), 7.48 (d, *J* = 8.2 Hz, 2H), 7.29 (d, *J* = 7.9 Hz, 2H), 7.15 – 7.11 (m, 2H), 2.71 (q, *J* = 7.6 Hz, 2H), 1.30 (t, *J* = 7.7 Hz, 3H) ppm.
^{13}C{^{1}H} NMR (176 MHz, $CDCl_3$): δ = 162.4 (d, *J* = 246 Hz), 143.5, 137.8, 137.4 (d, *J* = 3 Hz), 128.6 (d, *J* = 8 Hz), 128.5, 127.1, 115.67 (d, *J* = 21 Hz), 28.6, 15.7 ppm.
^{19}F NMR (565 MHz, $CDCl_3$): δ = –116.2 (tt, J = 9, 5 Hz) ppm.

The spectroscopic data are consistent with those reported in literature.[12]

10.7 4'-Ethyl-[1,1'-biphenyl]-4-carbonitrile (1i)

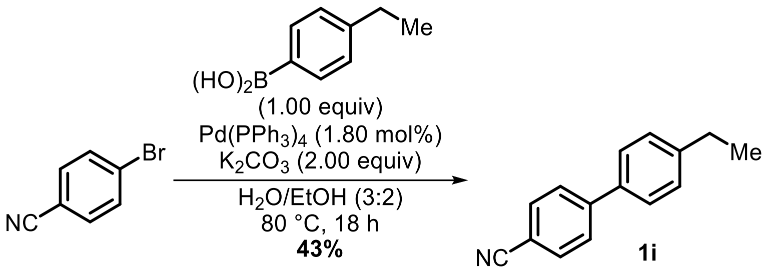

4'-Ethyl-[1,1'-biphenyl]-4-carbonitrile (**1i**) was prepared from 4-bromobenzonitrile (2.00 g, 11.0 mmol, 1.00 equiv), (4-ethylphenyl)boronic acid (1.61 g, 11.0 mmol, 1.00 equiv), $Pd(PPh_3)_4$ (231 mg, 200 µmol, 1.80 mol%), K_2CO_3 (3.00 g, 22.0 mmol, 2.00 equiv), H_2O (15 mL), and EtOH (10 mL) according to **GP1**. The reaction mixture was stirred at 80 °C for 18 h. **1i** (970 mg, 4.70 mmol, 43%) was purified by flash column chromatography (SiO_2, CyH/EtOAc, 9:1) and was isolated as colorless to yellowish solid.

^{1}H NMR (400 MHz, $CDCl_3$): δ = 7.74 – 7.65 (m, 4H), 7.55 – 7.50 (m, 2H), 7.35 – 7.30 (m, 2H), 2.72 (q, *J* = 7.6 Hz, 2H), 1.29 (t, *J* = 7.6 Hz, 3H) ppm.
^{13}C{^{1}H} NMR (151 MHz, $CDCl_3$): δ = 145.9, 145.4, 136.8, 132.9, 129.0, 127.8, 127.5, 119.4, 110.9, 28.9, 15.8 ppm.

The spectroscopic data are consistent with those reported in literature.[13]

10.8 4'-Ethyl-3-(trifluoromethyl)-1,1'-biphenyl (1j)

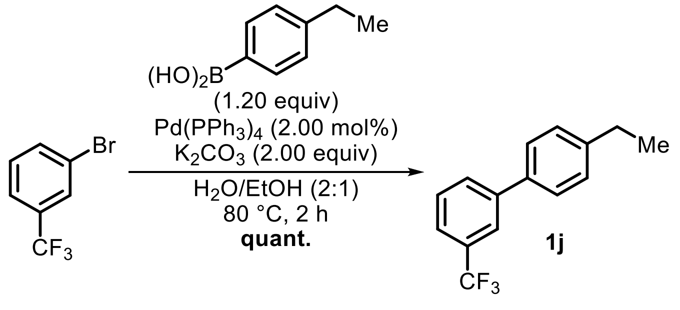

4'-Ethyl-3-(trifluoromethyl)-1,1'-biphenyl (**1j**) was prepared from 1-bromo-3-(trifluoromethyl)benzene (500 mg, 2.22 mmol, 1.00 equiv), (4-ethylphenyl)boronic acid (400 mg, 2.66 mmol, 1.20 equiv), $Pd(PPh_3)_4$ (51.0 mg, 44.0 µmol, 2.00 mol%), K_2CO_3 (614 mg, 4.44 mmol, 2.00 equiv), H_2O (3.3 mL), and EtOH (1.6 mL) according to **GP1**. The reaction mixture was stirred at 80 °C for 2 h. **1j** (555 mg, 2.22 mmol, quant.) was

purified by filtration through a short plug of silica (2 cm, rinsed with CyH) and was isolated as colorless to yellowish liquid.

^{1}H NMR (400 MHz, $CDCl_3$): δ = 7.83 (s, 1H), 7.76 (d, *J* = 7.5 Hz, 1H), 7.55 (m, 4H), 7.31 (d, *J* = 7.9 Hz, 2H), 2.72 (q, *J* = 7.6 Hz, 2H), 1.29 (t, *J* = 7.6 Hz, 3H) ppm.
$^{13}C\{^1H\}$ NMR (151 MHz, $CDCl_3$): δ = 144.6, 142.3, 137.5, 131.4 (q, *J* = 32.0 Hz), 130.6, 129.5, 128.9, 127.4, 124.6 (q, *J* = 272 Hz), 124.1 (q, *J* = 4 Hz), 124.0 (q, *J* = 4 Hz), 28.9, 15.9 ppm.
^{19}F NMR (565 MHz, $CDCl_3$): δ = –63.6 ppm.
HRMS (EI): m/z calculated for $[C_{15}H_{13}F_3]^+$ ($[M]^+$): 250.0964, measured: 250.0954.

10.9 2-(4-Ethylphenyl)pyridine (1k)

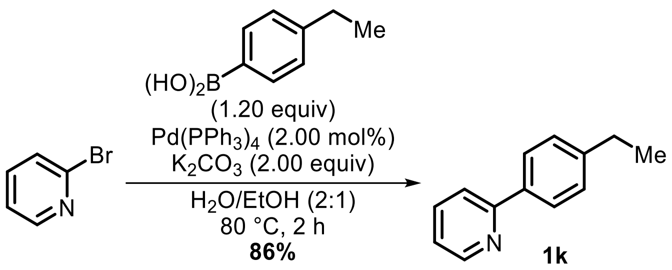

2-(4-Ethylphenyl)pyridine (**1k**) was prepared from 2-bromopyridine (500 mg, 3.16 mmol, 1.00 equiv), (4-ethylphenyl)boronic acid (569 mg, 3.80 mmol, 1.20 equiv), $Pd(PPh_3)_4$ (73 mg, 63.0 µmol, 2.00 mol%), K_2CO_3 (874 mg, 6.33 mmol, 2.00 equiv), H_2O (4.9 mL), and EtOH (2.4 mL) according to **GP1**. The reaction mixture was stirred at 80 °C for 2 h. **1k** (495 mg, 2.70 mmol, 86%) was purified using flash column chromatography (SiO_2, CyH/EtOAc, 9:1) and was as colorless to yellowish solid.

^{1}H NMR (400 MHz, $CDCl_3$): δ = 8.68 (dd, *J* = 4.8, 1.4 Hz, 1H), 7.92 (d, *J* = 8.2 Hz, 2H), 7.77 – 7.69 (m, 2H), 7.31 (d, *J* = 8.0 Hz, 2H), 7.21 (ddd, *J* = 6.7, 4.8, 2.0 Hz, 1H), 2.71 (q, *J* = 7.6 Hz, 2H), 1.28 (t, *J* = 7.6 Hz, 3H) ppm.
$^{13}C\{^1H\}$ NMR (151 MHz, $CDCl_3$): δ = 157.6, 149.6, 145.5, 137.0, 128.4, 127.0, 122.0, 120.5, 28.8, 15.6 ppm.

The spectroscopic data are consistent to those reported in literature.[14]

10.10 2-Methyl-4-phenylbut-2-yl acetate (1l)

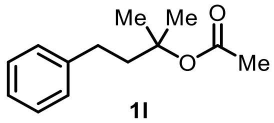

1l

2-Methyl-4-phenylbut-2-yl acetate (**1l**) was prepared from 2-methyl-4-phenylbutan-2-ol (Carbinol Muguet, 1.00 g, 6.09 mmol, 1.00 equiv), acetic anhydride (1.24 g, 1.15 mL, 12.2 mmol, 2.00 equiv), DMAP (74.4 mg, 609 µmol, 10 mol%), and pyridine (10 mL) according to **GP2** with a reaction time of 16 h. The product was purified by flash column chromatography (SiO_2, *n*-pentane/Et_2O, 20:1). **1l** (1.01 g, 4.90 mmol, 81%) was isolated as colorless liquid.

^{1}H NMR (400 MHz, $CDCl_3$): δ = 7.31 – 7.26 (m, 2H), 7.21 – 7.15 (m, 3H), 2.68 – 2.60 (m, 2H), 2.10 – 2.03 (m, 2H), 1.98 (s, 3H), 1.50 (s, 6H) ppm.
^{13}C{^{1}H} NMR (151 MHz, $CDCl_3$): δ = 170.8, 142.4, 128.7, 126.1, 82.3, 43.0, 30.7, 26.4, 22.7 ppm.

The spectroscopic data fits to those reported in literature.[15]

10.11 3-Methyl-5-phenylpentyl acetate (1m)

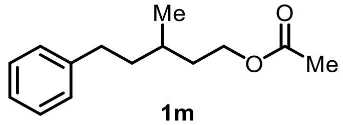

1m

3-Methyl-5-phenylpentyl acetate (**1m**) was prepared from 3-methyl-5-phenylpentan-1-ol (Phenoxanol, 326 mg, 1.83 mmol, 1.00 equiv), acetic anhydride (373 mg, 345 µL, 3.65 mmol, 2.00 equiv), DMAP (22.3 mg, 183 µmol, 10 mol%), and pyridine (2 mL) according to **GP2** with a reaction time of 16 h. The product was purified by flash column chromatography (SiO_2, *n*-pentane/Et_2O, 20:1). **1m** (346 mg, 1.57 mmol, 86%) was isolated as a colorless liquid.

^{1}H NMR (700 MHz, $CDCl_3$): δ = 7.30 – 7.26 (m, 2H), 7.20 – 7.16 (m, 3H), 4.16 – 4.07 (m, 2H), 2.67 (ddd, J = 13.6, 10.3, 5.7 Hz, 1H), 2.59 (ddd, J = 13.7, 10.3, 6.0 Hz, 1H), 1.73 (dtd, J = 13.6, 7.3, 5.3 Hz, 1H), 1.70 – 1.56 (m, 2H), 1.53 – 1.46 (m, 2H), 0.98 (d, J = 6.7 Hz, 3H) ppm.

$^{13}C\{^1H\}$ **NMR** (176 MHz, $CDCl_3$): δ = 171.5, 142.9, 128.7, 128.6, 126.0, 63.2, 39.1, 35.7, 33.6, 29.8, 21.4, 19.7 ppm.
HRMS (EI): m/z calculated for $[C_{14}H_{20}O_2]^+$ ($[M]^+$): 220.1458, measured: 220.1454.

10.12 3-Methyl-5-phenylpentyl 4-bromobenzoate (1n)

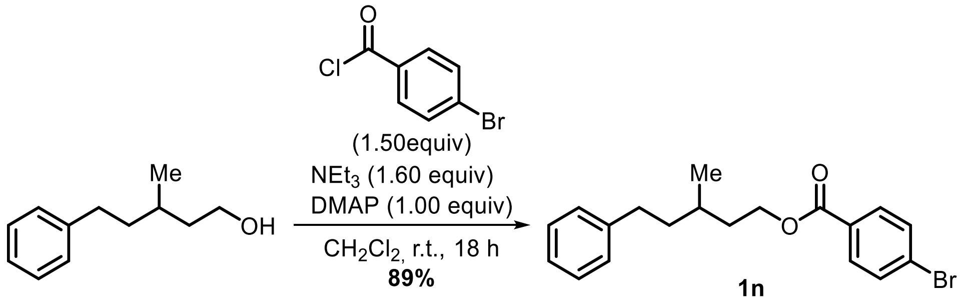

To a solution of 3-methyl-5-phenylpentan-1-ol (Phenoxanol, 190 mg, 1.07 mmol, 1.00 equiv) in dry CH_2Cl_2 (7 mL) was added 4-bromobenzoyl chloride (351 mg, 1.60 mmol, 1.50 equiv), NEt_3 (162 mg, 223 µL, 1.60 mmol, 1.50 equiv), and DMAP (130 mg, 1.07 mmol, 1.00 equiv) subsequently. The reaction mixture was stirred at r.t. for 18 h before the solvent was removed under reduced pressure. The product was purified by flash column chromatography (SiO_2, n-pentane/Et_2O, 10:1). **1n** (366 mg, 949 µmol, 89%) was isolated as yellowish oil.

^1H **NMR** (700 MHz, $CDCl_3$): δ= 7.88 – 7.83 (m, 2H), 7.59 – 7.55 (m, 2H), 7.28 – 7.25 (m, 2H), 7.19 – 7.16 (m, 3H), 4.40 – 4.32 (m, 2H), 2.70 (ddd, *J* = 13.7, 9.8, 5.7 Hz, 1H), 2.61 (ddd, *J* = 13.8, 10.0, 5.9 Hz, 1H), 1.89 – 1.84 (m, 1H), 1.74 – 1.66 (m, 2H), 1.65 – 1.59 (m, 1H), 1.56 – 1.52 (m, 1H), 1.03 (d, *J* = 6.5 Hz, 3H) ppm.
$^{13}C\{^1H\}$ **NMR** (176 MHz, $CDCl_3$): δ = 166.0, 142.6, 131.8, 131.2, 129.5, 128.5, 128.1, 125.8, 63.7, 38.8, 35.5, 33.4, 29.6, 19.6 ppm.
HRMS (ESI, pos): m/z calculated for $[C_{19}H_{21}BrO_2Na]^+$ ($[M+Na]^+$): 383.0617, measured: 383.0602.

10.13 Phenyl 2-(4-isobutylphenyl)propanoate (1o)

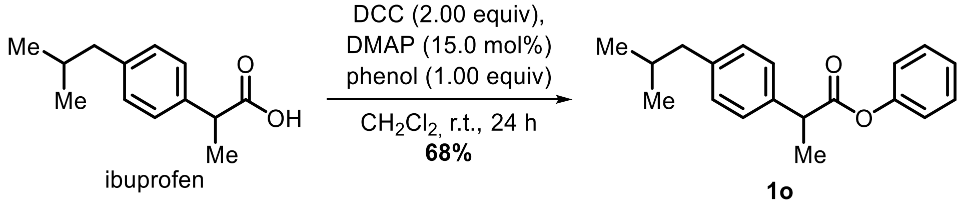

To a stirred solution of ibuprofen (2.00 g, 9.70 mmol, 1.00 equiv) and dicyclohexylcarbodiimide (DCC, 4.00 g, 19.4 mmol, 2.00 equiv) in CH_2Cl_2 (15 mL) were added 4-dimethylaminepyridine (DMAP, 177 mg, 1.45 mmol, 15.0 mol%) and phenol (912 mg, 9.70 mmol, 1.00 equiv). The reaction mixture was stirred at r.t. for 24 h. After filtration, the filtrate was concentrated under reduced pressure and the residue was purified by flash column chromatography (SiO_2, *n*-pentane/EtOAc, 20:1) to afford the product in a mixture with DCC. The mixture was dissolved in CH_2Cl_2, transferred into a separation funnel, and HCl (aq., 1 M, 40 mL) was added. After shaking, the two phases were separated and the aqueous phase was extracted with CH_2Cl_2 (4 × 20 mL). The combined organic phases were dried over $MgSO_4$ and the solvent was evaporated under reduced pressure. ^{1}H NMR analysis still showed DCC in the product, thus, the colorless liquid was dissolved in CH_2Cl_2 (20 mL) and HCl (aq., 1 M, 20 mL) was added in order to hydrolyse the DCC to dicyclohexylurea (DCU). After 3 h of stirring at r.t., the two phases were separated, the aqueous phase was extracted with CH_2Cl_2 (3 × 10 mL) and the combined organic phases were dried over $MgSO_4$, filtered, and the solvent was evaporated under reduced pressure. DCU preticipated and after filtration product **1o** (1.84 g, 6.52 mmol, 68%) was isolated as colorless oil.

^{1}H NMR (400 MHz, $CDCl_3$): δ = 7.38 – 7.28 (m, 4H), 7.22 – 7.17 (m, 1H), 7.15 (d, *J* = 7.9 Hz, 2H), 7.00 (d, *J* = 7.5 Hz, 2H), 3.94 (q, *J* = 7.1 Hz, 1H), 2.48 (d, *J* = 7.2 Hz, 2H), 1.91–1.81 (m, 1H), 1.61 (d, *J* = 7.1 Hz, 3H), 0.92 (d, *J* = 6.6 Hz, 6H) ppm.
^{13}C{^{1}H} NMR (151 MHz, $CDCl_3$): δ = 173.5, 151.2, 141.1, 137.6, 129.8, 129.6, 127.5, 126.0, 121.7, 45.6, 45.4, 30.5, 22.7, 18.8 ppm.

The spectroscopic data are consistent with those reported in literature.[16]

10.14 [1,1'-Biphenyl]-4-carbaldehyde (5b)

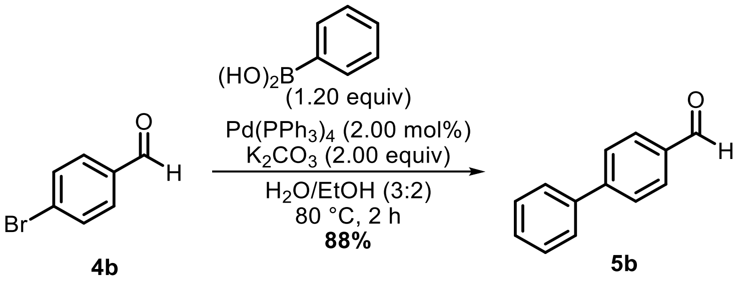

[1,1'-Biphenyl]-4-carbaldehyde (**5b**) was prepared from 4-bromobenzaldehyde (**4b**) (2.00 g, 10.8 mmol, 1.00 equiv), phenylboronic acid (1.58 g, 13.0 mmol, 1.20 equiv), $Pd(PPh_3)_4$ (249 mg, 220 μmol, 2.00 mol%), K_2CO_3 (3.00 g, 21.6 mmol, 2.00 equiv), H_2O (13.2 mL), and EtOH (8.8 mL) according to **GP1**. The reaction mixture was stirred for 2 h. **5b** (1.72 g, 9.44 mmol, 88%) was purified using flash column chromatography (SiO_2, *n*-pentane/EtOAc, 20:1) and was isolated as colorless solid.

^{1}H NMR (600 MHz, $CDCl_3$): δ = 10.06 (s, 1H), 7.96 (d, *J* = 8.1 Hz, 2H), 7.76 (d, *J* = 8.3 Hz, 2H), 7.64 (dt, *J* = 8.2, 1.8 Hz, 2H), 7.49 (td, *J* = 6.9, 1.7 Hz, 2H), 7.44 – 7.41 (m, 1H) ppm.

^{13}C{^{1}H} NMR (151 MHz, $CDCl_3$): δ = 192.3, 147.6, 140.1, 135.5, 130.6, 129.4, 128.8, 128.0, 127.7 ppm.

The spectroscopic data are consistent with those reported in literature.[17]

11. Synthesis of Trifluoromethoxylated Products

11.1 (1-(Trifluoromethoxy)ethyl)benzene (2a)

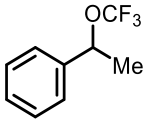

2a

(1-(Trifluoromethoxy)ethyl)benzene (**2a**) was prepared from ethylbenzene (53.0 mg, 61.2 µL, 500 µmol, 1.00 equiv) according to **GP3** with a reaction time of 4 h. **2a** was observed in a 66% ^{19}F NMR yield.

^{1}H NMR (400 MHz, Chloroform-*d*): δ = 7.41 – 7.31 (m, 5H), 5.30 (q, *J* = 6.6 Hz, 1H), 1.64 (d, *J* = 6.6, 3H) ppm.
^{13}C{^{1}H} NMR (176 MHz, $CDCl_3$): δ 140.8, 129.00, 128.8, 126.1, 122.0 (q, *J* = 255 Hz), 23.7 ppm.
^{19}F NMR (565 MHz, $CDCl_3$): δ = –57.9 ppm.

The spectroscopic data are consistent with those reported in literature.[18]

11.2 4-((Trifluoromethoxy)methyl)-1,1'-biphenyl (2b)

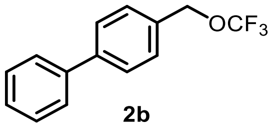

2b

4-((Trifluoromethoxy)methyl)-1,1'-biphenyl (**2b**) was prepared from 4-methyl-1,1'-biphenyl (**1b**) (85 mg, 500 µmol, 1.00 equiv) according to **GP3** with a reaction time of 4 h. **2b** (24.0 mg, 95.1 µmol, 19% (26% ^{19}F NMR yield)) was purified by flash column chromatography (SiO_2, CyH) and was isolated as colorless solid.

^{1}H NMR (400 MHz, $CDCl_3$): δ = 7.61 (dd, *J* = 12.4, 7.8 Hz, 4H), 7.46 (t, *J* = 7.1 Hz, 4H), 7.37 (t, *J* = 7.3 Hz, 1H), 5.03 (s, 2H) ppm.
^{13}C{^{1}H} NMR (151 MHz, $CDCl_3$): δ = 142.0, 140.5, 132.8, 129.0, 128.7, 127.8, 127.6, 127.3, 121.8 (q, *J* = 256 Hz), 69.0 (q, *J* = 3 Hz) ppm.
^{19}F NMR (376 MHz, $CDCl_3$): δ = –60.2 ppm.

The spectroscopic data are consistent with those reported in literature.[5]

11.3 4-(1-(Trifluoromethoxy)ethyl)-1,1'-biphenyl (2c)

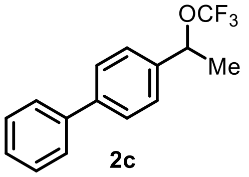

2c

4-(1-(Trifluoromethoxy)ethyl-1,1'-biphenyl (**2c**) was prepared from 4-ethyl-1,1'-biphenyl (**1c**) (91.0 mg, 500 µmol, 1.00 equiv) according to **GP3** with a reaction time of 4 h. **2c** (73.0 mg, 270 µmol, 55% (60% ^{19}F NMR yield)) was purified by flash column chromatography (SiO_2, *n*-pentane) and was isolated as colorless solid.

^{1}H NMR (500 MHz, $CDCl_3$): δ = 7.63 – 7.58 (m, 4H), 7.48 – 7.42 (m, 4H), 7.39 – 7.35 (m, 1H), 5.36 (q, *J* = 6.6 Hz, 1H), 1.68 (d, *J* = 6.6 Hz, 3H) ppm.
^{13}C{^{1}H} NMR (151 MHz, $CDCl_3$): δ = 141.8, 140.9, 139.7, 129.2, 127.9, 127.7, 127.5, 126.6, 122.1 (q, *J* = 255 Hz), 77.3, 23.7 ppm.
^{19}F NMR (565 MHz, $CDCl_3$): δ = –57.8 ppm.

The spectroscopic data are consistent with those reported in literature.[5]

11.4 4-Methyl-4'-(1-(trifluoromethoxy)ethyl)-1,1'-biphenyl (2e) and 4-ethyl-4'-((trifluoromethoxy)methyl)-1,1'-biphenyl (2e')

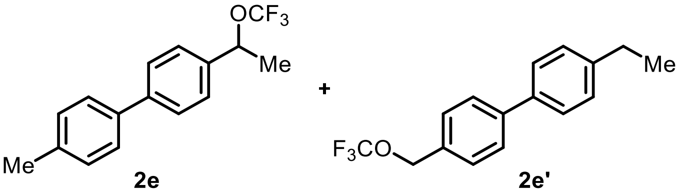

2e **2e'**

4-Methyl-4'-(1-(trifluoromethoxy)ethyl)-1,1'-biphenyl (**2e**) and 4-ethyl-4'-((trifluoromethoxy)methyl)-1,1'-biphenyl (**2e'**) were prepared from 4-ethyl-4'-methyl-1,1'-biphenyl (**1e**) (100 mg, 509 µmol, 1.00 equiv) according to **GP3** with a reaction time of 4 h. **2e** and **2e'** (combined: 33.0 mg, 118 µmol, 23% (25% ^{19}F NMR yield)) were purified by flash column chromatography (SiO_2, CyH) and were isolated as colorless crystals in a 6.5:1 (**2e**/**2e'**) mixture.

2e:

^{1}**H NMR** (400 MHz, $CDCl_3$): δ = 7.58 (d, *J* = 8.3 Hz, 2H), 7.48 (d, *J* = 8.1 Hz, 2H), 7.40 (d, *J* = 8.2 Hz, 2H), 7.25 (d, *J* = 8.0 Hz, 2H), 5.34 (q, *J* = 6.7 Hz, 1H), 2.40 (s, 3H), 1.67 (d, *J* = 6.5 Hz, 3H) ppm.

^{13}**C NMR** (176 MHz, $CDCl_3$): δ = 141.5, 139.2, 137.8, 137.5, 129.7, 127.3, 127.1, 126.3, 121.8 (q, *J* = 255 Hz), 77.3–77.1 (m), 23.4, 21.3 ppm.

^{19}**F NMR** (376 MHz, $CDCl_3$): δ = –57.8 ppm.

The spectroscopic data are consistent with those reported in literature.[5]

2e':

^{19}**F NMR** (376 MHz, $CDCl_3$): δ = –60.1 ppm.

The spectroscopic data are consistent with those reported in literature.[5]

11.5 4-Isopropyl-4'-(1-(trifluoromethoxy)ethyl)-1,1'-biphenyl (2f)

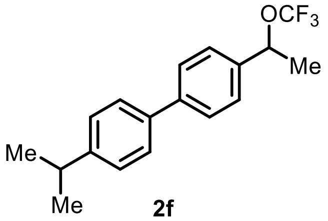

2f

4-Isopropyl-4'-(1-(trifluoromethoxy)ethyl)-1,1'-biphenyl (**2f**) was prepared from 4-ethyl-4'-isopropyl-1,1'-biphenyl (**1f**) (114 mg, 509 µmol, 1.00 equiv) according to **GP3** with a reaction time of 4 h. **2f** (32.0 mg, 104 µmol, 21% (22% ^{19}F NMR yield)) was purified by flash column chromatography (SiO_2, CyH) and was isolated as colorless crystals.

^{1}**H NMR** (400 MHz, $CDCl_3$): δ = 7.58 (d, *J* = 8.3 Hz, 2H), 7.51 (d, *J* = 8.3 Hz, 2H), 7.40 (d, *J* = 8.3 Hz, 2H), 7.31 (d, *J* = 8.2 Hz, 2H), 5.34 (q, *J* = 6.5 Hz, 1H), 2.95 (sept, *J* = 6.8 Hz, 1H), 1.67 (d, *J* = 6.6 Hz, 3H), 1.29 (d, *J* = 6.9 Hz, 6H) ppm.

^{13}**C NMR** (176 MHz, $CDCl_3$): δ = 148.4, 141.5, 139.1, 138.2, 127.4, 127.2, 127.1, 126.3, 121.8 (q, *J* = 274, 265 Hz), 77.2–77.1 (m), 34.0, 29.9, 24.1, 23.4 ppm.

^{19}**F NMR** (376 MHz, $CDCl_3$): δ = –57.81 ppm.

HRMS (EI): m/z calculated for $[C_{18}H_{19}F_3O]^+$($[M]^+$): 308.1388, measured: 308.1394.

11.6 1-(Tert-butyl)-3-(1-(trifluoromethoxy)ethyl)benzene (2g)

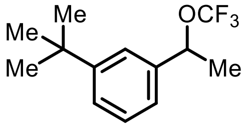

2g

1-(*Tert*-butyl)-3-(1-(trifluoromethoxy)ethyl)benzene (**2g**) was prepared from 1-(tert-butyl)-3-ethylbenzene (82.0 mg, 95.3 µL, 500 µmol, 1.00 equiv) according to **GP3** with a reaction time of 4 h. **2g** (calculated mass from ^{1}H-NMR: 31.5 mg, 128 µmol, 26% (41% ^{19}F NMR yield)) was purified by flash column chromatography (SiO_2, *n*-pentane) and was isolated as colorless liquid in an inseparable 2:1 mixture with the starting material.

^{1}H NMR (600 MHz, $CDCl_3$): δ = 7.42 – 7.38 (m, 2H), 7.35 (t, *J* = 7.6 Hz, 1H), 7.21 (d, *J* = 7.3 Hz, 1H), 5.34 (q, *J* = 6.5 Hz, 1H), 1.68 (d, *J* = 6.6 Hz, 3H), 1.37 (s, 9H) ppm.
^{13}C{^{1}H} NMR (151 MHz, $CDCl_3$): δ = 151.7, 140.1, 128.4, 125.5, 122.9, 122.8, 121.8 (q, *J* = 255 Hz), 77.7 (q, *J* = 2 Hz), 34.9, 31.4, 23.5 ppm.
^{19}F NMR (565 MHz, $CDCl_3$): δ = –57.7 ppm.
HRMS (EI): m/z calculated for $[C_{13}H_{17}F_3O]^+$ ($[M+]^+$): 246.1226, measured: 246.1222.

11.7 4-Fluoro-4'-(1-(trifluoromethoxy)ethyl)-1,1'-biphenyl (2h)

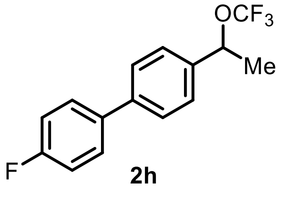

2h

4-Fluoro-4'-(1-(trifluoromethoxy)ethyl)-1,1'-biphenyl (**2h**) was prepared from 4-ethyl-4'-fluoro-1,1'-biphenyl (**1h**) (100 mg, 500 µmol, 1.00 equiv) according to **GP3** with a reaction time of 4 h. **2h** (50.0 mg, 180 µmol, 36% (51% ^{19}F NMR)) was purified by flash column chromatography (SiO_2, CyH) and was isolated as colorless solid.

^{1}H NMR (700 MHz, $CDCl_3$): δ = 7.60 – 7.55 (m, 4H), 7.47 – 7.43 (m, 2H), 7.16 (t, *J* = 8.6 Hz, 2H), 5.38 (q, *J* = 6.6 Hz, 1H), 1.70 (d, *J* = 6.6 Hz, 3H) ppm.
^{13}C{^{1}H} NMR (176 MHz, $CDCl_3$): δ = 162.9 (d, *J* = 247 Hz), 140.8, 139.8, 137.0 (d, *J* = 3.2 Hz), 129.0 (d, *J* = 8 Hz), 127.6, 126.6, 122.0 (q, *J* = 255 Hz), 116.1 (d, *J* = 21 Hz), 77.2, 23.7 ppm.
^{19}F NMR (376 MHz, $CDCl_3$): δ = –57.9, –115.2 – –115.3 (m) ppm.

HRMS (EI): m/z calculated for $[C_{15}H_{12}F_4O]^+([M]^+)$: 284.0819, measured: 284.0828.

11.8 4'-(1-(Trifluoromethoxy)ethyl)-[1,1'-biphenyl]-4-carbonitrile (2i)

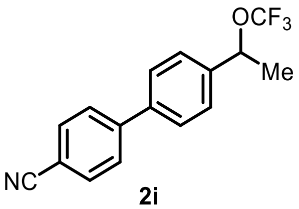

2i

4'-(1-(Trifluoromethoxy)ethyl)-[1,1'-biphenyl]-4-carbonitrile (**2i**) was prepared from 4'-ethyl-[1,1'-biphenyl]-4-carbonitrile (**1i**) (105 mg, 500 µmol, 1.00 equiv) according to **GP3** with a reaction time of 4 h. **2i** (76 mg, 261 µmol, 52% (52% ^{19}F NMR yield)) was purified by flash column chromatography (SiO_2, CyH → CyH/EtOAc, 9:1) and was isolated as yellowish oil.

^{1}H NMR (400 MHz, $CDCl_3$): δ = 7.76 – 7.72 (m, 2H), 7.68 (d, *J* = 8.4 Hz, 2H), 7.63 – 7.58 (m, 2H), 7.47 (d, *J* = 8.3 Hz, 2H), 5.36 (q, *J* = 6.6 Hz, 1H), 1.67 (d, *J* = 6.5 Hz, 3H) ppm.
^{13}C{^{1}H} NMR (176 MHz, $CDCl_3$): δ = 145.3, 141.3, 139.6, 133.0, 128.1, 127.9, 126.8, 122.0 (q, *J* = 255 Hz), 119.2, 111.6, 77.0 (q, *J* = 2 Hz), 23.7 ppm.
^{19}F NMR (376 MHz, $CDCl_3$) δ = –58.0 ppm.
HRMS (EI): m/z calculated for $[C_{16}H_{12}F_3NO]^+([M]^+)$: 291.0866, measured: 291.0853.

11.9 4'-(1-(Trifluoromethoxy)ethyl)-3-(trifluoromethyl)-1,1'-biphenyl (2j)

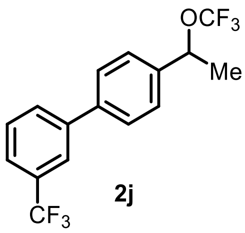

2j

4'-(1-(Trifluoromethoxy)ethyl)-3-(trifluoromethyl)-1,1'-biphenyl (**2j**) was prepared from 4'-ethyl-3-(trifluoromethyl)-1,1'-biphenyl (**1j**) (93.0 mg, 371 µmol, 1.00 equiv) according to **GP3** with a reaction time of 4 h. **2j** (35 mg, 126 µmol, 34% (40% ^{19}F NMR yield)) was purified by flash column chromatography (SiO_2, CyH) and was isolated as colorless oil.

^{1}**H NMR** (400 MHz, $CDCl_3$): δ = 7.83 (s, 1H), 7.76 (d, *J* = 7.6 Hz, 1H), 7.62 (dd, *J* = 7.6, 5.5 Hz, 3H), 7.59 – 7.53 (m, 1H), 7.49 – 7.44 (m, 2H), 5.36 (q, *J* = 6.6 Hz, 1H), 1.68 (d, *J* = 6.5 Hz, 3H) ppm.
^{13}**C{^{1}H} NMR** (176 MHz, $CDCl_3$): δ = 141.7, 140.7, 140.3, 131.6 (q, *J* = 32 Hz), 130.7, 129.7, 127.8, 126.8, 124.5 (q, 272 Hz), 124.5 (q, *J* = 4 Hz), 124.3 (q, *J* = 4 Hz), 122.0 (q, *J* = 255 Hz), 77.1 (q, *J* = 3 Hz), 23.7 ppm.
^{19}**F NMR** (376 MHz, $CDCl_3$) δ = –57.9, –62.5 ppm.
HRMS (EI): m/z calculated for $[C_{16}H_{12}F_6O]^+$($[M]^+$): 334.0787, measured: 334.0790.

11.10 2-(4-(1-(Trifluoromethoxy)ethyl)phenyl)pyridine (2k)

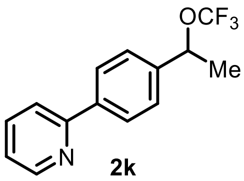

2k

2-(4-(1-(Trifluoromethoxy)ethyl)phenyl)pyridine (**2k**) was prepared from 2-(4-ethylphenyl)pyridine (**1k**) (125 mg, 682 µmol, 1.00 equiv) according to **GP3** with a reaction time of 16 h. **2k** (50 mg, 187 µmol, 28% (65% ^{19}F NMR yield)) was purified by flash column chromatography (SiO_2, CyH/EtOAc, 9:1) and was isolated as yellowish oil that solidifies in the fridge at 5 °C.

^{1}**H NMR** (600 MHz, $CDCl_3$): δ = 8.70 (d, *J* = 4.6 Hz, 1H), 8.01 (d, *J* = 8.3 Hz, 2H), 7.79 – 7.72 (m, 2H), 7.46 (d, *J* = 8.2 Hz, 2H), 7.26 – 7.23 (m, 1H), 5.36 (q, *J* = 6.6 Hz, 1H), 1.67 (d, *J* = 6.6 Hz, 3H) ppm.
^{13}**C{^{1}H} NMR** (151 MHz, $CDCL_3$): δ = 157.2, 150.1, 141.5, 139.9, 137.2, 127.5, 126.5, 122.7, 122.0 (q, J = 255 Hz), 120.9, 77.3 – 77.2 (m), 23.7 ppm.
^{19}**F NMR** (565 MHz, $CDCl_3$): δ = –57.9 ppm.
HRMS (EI): m/z calculated for $[C_{14}H_{12}F_3NO]^+$($[M]^+$): 267.0866, measured: 267.0870.

11.11 2-Methyl-4-phenyl-4-(trifluoromethoxy)butanyl acetate (2l)

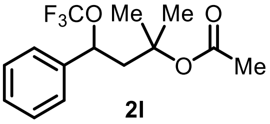

2l

2-Methyl-4-phenyl-4-(trifluoromethoxy)butan-2-yl acetate (**2l**) was prepared from 2-methyl-4-phenylbutan-2-yl acetate (**1l**) (105 mg, 509 µmol, 1.00 equiv) according to

GP3 with a reaction time of 4 h. **2l** (15.0 mg, 50.0 µmol, 10% (30% ^{19}F NMR yield)) was purified by flash column chromatography (SiO_2, *n*-pentane) and was isolated as colorless liquid.

^{1}H NMR (700 MHz, $CDCl_3$): δ = 7.38 – 7.30 (m, 5H), 5.30 (dd, *J* = 8.5, 4.0 Hz, 1H), 2.54 (dd, *J* = 15.3, 8.5 Hz, 1H), 2.24 (dd, *J* = 15.2, 4.0 Hz, 1H), 1.89 (s, 3H), 1.55 (s, 3H), 1.49 (s, 3H) ppm.
^{13}C{^{1}H} NMR (176 MHz, $CDCl_3$): δ = 170.8, 140.5, 129.0, 128.8, 126.5, 121.9 (q, *J* = 256 Hz), 80.5, 78.3 (q, *J* = 3 Hz), 47.3, 27.5, 26.9, 22.5 ppm.
^{19}F NMR (376 MHz, $CDCl_3$): δ = –57.4 ppm.
HRMS (EI): m/z calculated for $[C_{12}H_{14}F_3O]^+$ ($[M-H_3CCO_2^-]^+$): 231.0991, measured: 231.0983.

11.12 3-Methyl-5-phenyl-5-(trifluoromethoxy)pentyl acetate (2m)

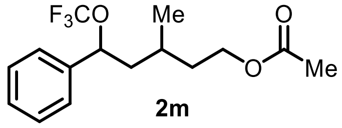

2m

3-Methyl-5-phenyl-5-(trifluoromethoxy)pentyl acetate (**2m**) was prepared from 3-methyl-5-phenylpentyl acetate (**1m**) (110 mg, 500 µmol, 1.00 equiv) according to **GP3** with a reaction time of 4 h. **2m** (47 mg, 154 µmol, 30% (33% ^{19}F NMR yield)) was purified by flash column chromatography (SiO_2, *n*-pentane/Et_2O, 15:1) and was isolated as colorless liquid in a mixture of diastereomers.

^{1}H NMR (600 MHz, $CDCl_3$): δ = 7.36 – 7.31 (m, 5H), 5.16 – 5.13 (m, 1H), 4.13 – 4.06 (m, 2H), 2.03 (s, 3H), 1.78 – 1.45 (m, 5H), 0.99 (d, *J* = 6.6 Hz, 3H) ppm.
^{13}C and ^{19}F NMR data of major diastereomer:
^{13}C{^{1}H} NMR (151 MHz, $CDCl_3$): δ = 171.5, 140.3, 129.0, 128.8, 126.3, 123.7 (q, *J* = 256 Hz), 79.3 (q, *J* = 2.3 Hz), 62.6, 45.2, 35.8, 26.6, 21.3, 20.0 ppm.
^{19}F NMR (564 MHz, $CDCl_3$): δ = –57.5 ppm.
HRMS (EI): m/z calculated for $[C_{15}H_{19}F_3O_3]^+$ ($[M+]^+$): 304.1281, measured: 304.1268.

11.13 3-Methyl-5-phenyl-5-(trifluoromethoxy)pentyl 4-bromobenzoate (2n)

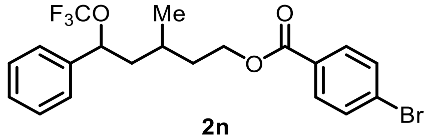

2n

3-Methyl-5-phenyl-5-(trifluoromethoxy)pentyl 4-bromobenzoate **2n** was prepared from 3-methyl-5-phenylpentyl 4-bromobenzoate **1n** (180 mg, 500 µmol, 1.00 equiv) according to **GP3** with a reaction time of 16 h. **2n** (64 mg, 144 µmol, 29% (30% ^{19}F NMR yield)) was purified by flash column chromatography (SiO_2, CyH/EtOAc, 19:1) and was isolated in a mixture of diastereomers as yellowish oil.

^{1}H NMR (400 MHz, $CDCl_3$): δ = 8.09 – 7.77 (m, 2H), 7.61 – 7.39 (m, 2H), 7.37 – 7.13 (m, 5H), 5.23 – 5.04 (m, 1H), 4.42 – 4.19 (m, 2H), 2.13 – 1.78 (m, 3H), 1.74 – 1.52 (m, 2H), 1.10 – 0.96 (m, 3H) ppm.
^{13}C NMR and ^{19}F NMR data of one diastereomer:
^{13}C{^{1}H} NMR (151 MHz, $CDCl_3$): δ = 165.9, 140.0, 131.8, 131.7, 131.2, 128.8, 128.6, 121.8 (q, *J* = 256 Hz), 121.0, 79.1, 63.2, 45.0, 35.1, 26.4, 19.7 ppm.
^{19}F NMR (376 MHz, $CDCl_3$): δ = –57.5 ppm.
HRMS (EI): m/z calculated for $[C_{20}H_{20}BrF_3O_3]^+$($[M]^+$): 444.0542, measured: 444.0539.

11.14 Phenyl 2-(4-(2-methyl-1-(trifluoromethoxy)propyl)phenyl)propanoate (2o)

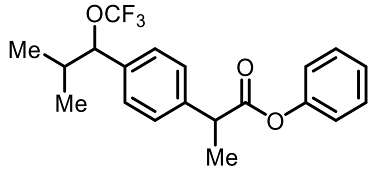

2o

Phenyl 2-(4-(2-methyl-1-(trifluoromethoxy)propyl)phenyl)propanoate (**2o**) was prepared from phenyl 2-(4-isobutylphenyl)propanoate (**1o**) (145 mg, 513 µmol, 1.00 equiv) according to **GP3** with a reaction time of 36 h. **2o** (100 mg, 273 µmol, 52% (70% ^{19}F NMR yield)) was purified by flash column chromatography (SiO_2, EtOAc/CyH, 1%) and was isolated in a mixture of diastereomers as yellowish oil that solidifies in the fridge at 5 °C.

^{1}H NMR (600 MHz, $CDCl_3$): δ = 7.40 (d, *J* = 8.2 Hz, 2H), 7.34 (tt, *J* = 7.6, 2.2 Hz, 2H), 7.29 – 7.27 (m, 2H), 7.22 – 7.19 (m, 1H), 7.00 – 6.97 (m, 2H), 4.78 (d, *J* = 7.1 Hz, 1H),

3.98 (q, J = 7.2 Hz, 1H), 2.09 (dhept, J = 14.5, 7.0 Hz, 1H), 1.63 (d, J = 7.2 Hz, 3H), 1.02 (d, J = 6.7 Hz, 3H), 0.83 (d, J = 6.8 Hz, 3H) ppm.

$^{13}C\{^1H\}$ NMR (176 MHz, $CDCl_3$): δ = 173.1, 150.9, 140.0, 138.0, 129.5, 127.6, 127.2, 126.0, 122.0 (q, J = 255 Hz), 121.5, 85.8, 45.5, 34.5, 18.7, 18.6, 18.2 ppm.

^{19}F NMR (565 MHz, $CDCl_3$): δ = –57.6 ppm.

HRMS (EI): m/z calculated for $[C_{20}H_{21}F_3O_3]^+([M]^+)$: 366.1437, measured: 366.1440.

11.14.1 Gram scale: Phenyl 2-(4-(2-methyl-1-(trifluoromethoxy)propyl)phenyl)propanoate (2o)

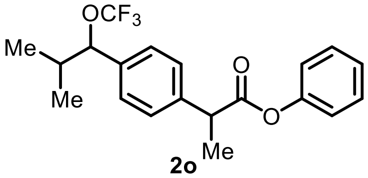

2o

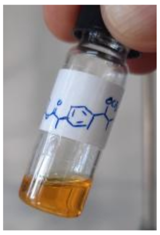

Phenyl-2 (4-(2-methyl-1-(trifluoromethoxy)propyl)phenyl)propanoate (**2o**) was prepared from phenyl 2-(4-isobutylphenyl)propanoate (**1o**)(1.00 g, 3.54 mmol, 1.00 equiv) according to **GP3** with a reaction time of 36 h. **2o** (668 mg, 1.82 mmol, 51% (54% ^{19}F NMR yield)) was purified by flash column chromatography (SiO_2, EtOAc/CyH, 1%) and was isolated in a mixture of diastereomers as yellowish oil that solidifies in the fridge at 5 °C.

11.15 (Trifluoromethoxy)cyclohexane (4a)

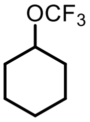

4a

(Trifluoromethoxy)cyclohexane (**4a**) was prepared from cyclohexane (42.0 mg, 53.9 µL, 500 µmol, 1.00 equiv) according to **GP4** with a reaction time of 2 h.

4a couldn't be isolated due to high volatility (51% ^{19}F NMR yield).

^{19}F NMR (376 MHz, $CDCl_3$): δ = –58.6 ppm.

The ^{19}F NMR shift is consistent with those reported in the literature.[19]

11.16 (Trifluoromethoxy)cyclododecane (4b)

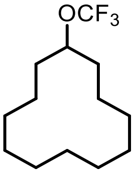

4b

(Trifluoromethoxy)cyclododecane (**4b**) was prepared from cyclododecane (84.0 mg, 500 µmol, 1.00 equiv) according to **GP4** with a reaction time of 18 h. **4b** (60.0 mg, 238 µmol, 48% (48% ^{19}F NMR yield)) was purified by flash column chromatography (SiO_2, *n*-pentane) and was isolated as colorless liquid.

^{1}H NMR (600 MHz, $CDCl_3$) δ = 4.36 (tt, *J* = 7.3, 4.6 Hz, 1H), 1.82 – 1.75 (m, 2H), 1.65 – 1.58 (m, 2H), 1.46 – 1.33 (m, 18H) ppm.
^{13}C{^{1}H} NMR (176 MHz, $CDCl_3$): δ = 122.2 (q, *J* = 253 Hz), 78.2 (q, *J* = 2 Hz), 30.1, 24.3, 24.0, 23.6, 23.5, 20.9 ppm.
^{19}F NMR (376 MHz, $CDCl_3$): δ = –58.7 ppm.
HRMS (EI): m/z calculated for $[C_{12}H_{23}]^+$ ($[M–OCF_3]^+$): 167.1794, measured: 167.1794.

The ^{19}F NMR shift is consistent with those reported in the literature.[20]

11.17 Bis(trifluoromethoxy)cyclododecane (4c)

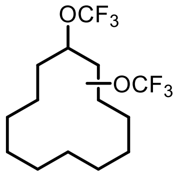

4c

Bis(trifluoromethoxy)cyclododecane (**4c**) was prepared from (trifluoromethoxy)cyclododecane (**4b**) (125 mg, 500 µmol, 1.00 equiv) according to **GP4** with a reaction time of 18 h. **4c** (42.0 mg, 125 µmol, 25% (25% ^{19}F NMR yield)) was purified by flash column chromatography (SiO_2, *n*-pentane) and was isolated as colorless liquid.

The product was isolated as a mixture of different regioisomers. Therefore, there are different sets of signals in the ^{1}H, ^{19}F, and ^{13}C NMR, which makes a distinguishment between the different sets very complicated. The signals are given without assignment. Two quartetts resulting from the C–F coupling are shown.

^{1}H NMR (400 MHz, $CDCl_3$): δ = 4.32 (m, 2 H), 1.86 – 1.55 (m, 8H), 1.54 – 1.25 (m, 12H) ppm.

^{13}C{^{1}H} NMR (151 MHz, CD_2Cl_2): δ = 124.5, 123.0, 122.0 (q, *J* = 250 Hz),121.8 (q, *J* = 250 Hz), 121.3, 120.3, 118.8, 78.5, 78.5, 78.5, 78.5, 78.0 (q, *J* = 2 Hz), 77.9, 77.9, 77.9, 77.9, 77.9, 77.8, 77.8, 77.5, 77.5, 77.5, 77.5, 30.6, 30.4, 30.3, 30.3, 29.9, 29.9, 29.8, 28.9, 28.8, 28.7, 27.1, 25.7, 24.5, 24.4, 24.3, 23.8, 23.7, 23.4, 21.9, 21.8, 20.7, 20.6, 20.5, 20.5, 19.7, 19.6, 19.1 ppm.

^{19}F NMR (376 MHz, $CDCl_3$): δ = –57.7, –57.8, –57.8, –58.0, –58.0 ppm.

HRMS (EI): m/z calculated for $[C_{13}H_{22}F_3O]^+$ ($[M–OCF_3^{\cdot}]^+$): 251.1617, measured: 251.1631.

11.18 (3*S*,5*S*,7*S*)-1-(Trifluoromethoxy)adamantane (4d) and (1*R*,3*R*,5*R*,7*R*)-2-(trifluoromethoxy)adamantane (4d')

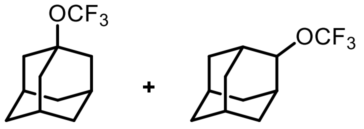

4d **4d'**

(3*S*,5*S*,7*S*)-1-(Trifluoromethoxy)adamantane (**4d**) and (1*R*,3*R*,5*R*,7*R*)-2-(trifluoromethoxy)adamantane (**4e**) were prepared from adamantane (69.0 mg, 500 µmol, 1.00 equiv) according to **GP4** with a reaction time of 4 h. **4d** and **4d'** were obtained in a 14% and 17% ^{19}F NMR yield, respectively.

4d:

^{19}F NMR (565 MHz, $CDCl_3$): δ = –49.0 ppm.

The ^{19}F NMR shift is consistent with those reported in the literature.[20]

4d':

^{19}F NMR (565 MHz, $CDCl_3$): δ = –57.3 ppm.

HRMS (EI): m/z calculated for $[C_{10}H_{14}F_3O]^+$ ($[M–H^{\cdot}]^+$): 219.0991, measured: 219.1049.

11.19 (3a*R*,8*S*,9a*S*)-3a,6,6,9a-Tetramethyl-8-(trifluoromethoxy)decahydronaphtho[2,1-b]furan-2(1H)-one (4e)

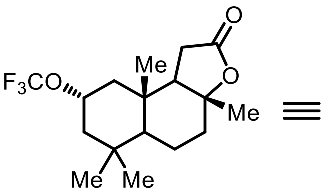

4e

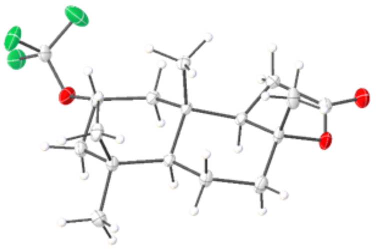

X-ray
CCDC 2435030

(3a*R*,8*S*,9a*S*)-Tetramethyl-8-(trifluoromethoxy)decahydronaphtho[2,1-b]furan-2(1*H*)-one (**4e**) was prepared from (3a*R*)-(+)-sclareolide (250 mg, 1.00 mmol, 1.00 equiv) according to **GP4** with a reaction time of 2 h. **4e** (64.0 mg, 200 µmol, 20% (25% ^{19}F NMR yield), 27% brsm) was purified by flash column chromatography (SiO_2, CyH/EtOAc, 5:1) and was isolated as a colorless to light yellowish solid.

^1H NMR (700 MHz, $CDCl_3$): δ = 4.47 (tt, *J* = 11.5, 4.6 Hz, 1H), 2.45 (dd, *J* = 16.2, 14.7 Hz, 1H), 2.28 (dd, *J* = 16.1, 6.5 Hz, 1H), 2.11 (dt, *J* = 12.0, 3.3 Hz, 1H), 2.02 (dd, *J* = 14.7, 6.5 Hz, 1H), 1.96 – 1.89 (m, 3H), 1.71 (td, *J* = 13.1, 12.6 Hz, 1H), 1.41 – 1.36 (m, 2H), 1.34 (d, *J* = 1.0 Hz, 3H), 1.23 (t, *J* = 11.8 Hz, 1H), 1.12 (dd, *J* = 12.7, 2.8 Hz, 1H), 0.99 (s, 3H), 0.98 (s, 3H), 0.92 (s, 3H) ppm.
$^{13}C\{^1H\}$ NMR (176 MHz, $CDCl_3$): δ = 176.3, 122.0 (q, *J* = 254 Hz), 109.7, 86.1, 73.3, 59.0, 56.1, 48.1, 45.6, 38.7, 37.56, 35.1, 33.4, 29.0, 22.0, 20.5, 16.4 ppm.
^{19}F NMR (376 MHz, $CDCl_3$): δ = –57.7 ppm.
HRMS (EI): m/z calculated for $[C_{17}H_{25}F_3O_3]^+$ ($[M]^+$): 334.1750, measured: 334.1751.
X-ray: Crystals were grown by slow evaporation of a solution of **4f** in CH_2Cl_2 and CyH at ambient temperature.

11.20 Trifluoromethyl benzoate (6a)

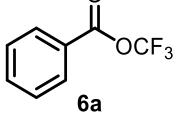

6a

Trifluoromethyl benzoate (6a) was prepared from benzaldehyde (**5a**) (53.0 mg, 51.0 µL, 1.00 equiv) according to **GP5** with a reaction time of 2 h. **6a** was obtained in 30% ^{19}F NMR yield.

^{19}F NMR (565 MHz, $CDCl_3$): δ = –58.1 ppm.

The ^{19}F NMR shift is consistent with those reported in the literature.[21]

11.21 Trifluoromethyl [1,1'-biphenyl]-4-carboxylate (6b)

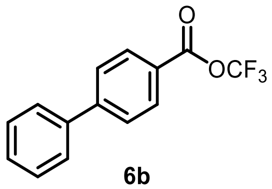

6b

Trifluoromethyl [1,1'-biphenyl]-4-carboxylate (**6b**) was prepared from [1,1'-biphenyl]-4-carbaldehyde (**5b**, 266 mg, 1.00 mmol, 1.00 equiv) according to **GP5** with a reaction time of 30 min. Purified by flash chromatography (SiO_2, *n*-pentane/Et_2O, 30:1 → 10:1). **6b** was isolated as colorless solid (45.0 mg, 170 µmol, 17%, 62% brsm, (20% ^{19}F NMR yield)).

^{1}H NMR (600 MHz, $CDCl_3$): δ = 8.16 – 8.12 (m, 2H), 7.75 – 7.72 (m, 2H), 7.65 (dd, *J* = 8.2, 1.1 Hz, 2H), 7.53 – 7.49 (m, 2H), 7.47 – 7.43 (m, 1H) ppm.
^{13}C{^{1}H} NMR (151 MHz, $CDCl_3$): δ = 159.3, 148.2, 139.6, 131.5, 129.4, 129.1, 127.9, 127.7, 125.5, 120.3 (q, *J* = 265 Hz) ppm.
^{19}F NMR (565 MHz, $CDCl_3$): δ = –56.8 ppm.

The spectroscopic data are consistent to those reported in literature.[22]

11.22 Trifluoromethyl octanoate (6c)

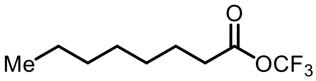

6c

Trifluoromethyl octanoate **(6c)** was prepared from octanal (52.0 mg, 79.2 µL, 500 µmol, 1.00 equiv) according to **GP5** with a reaction time of 2 h.
6c was obtained in 46% ^{19}F NMR yield. Compound **6c** was too volatile to be isolated.

^{19}F NMR ($CDCl_3$, 376 MHz): δ = –58.6 ppm.

11.23 Trifluoromethyl 3-phenylpropanoate (6d)

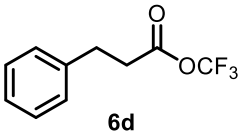

6d

Trifluoromethyl 3-phenylpropanoate (**6d**) was prepared from 3-phenylpropanal (68.0 mg, 67.3 µL, 500 µmol, 1.00 equiv) according to **GP5**. **6d** was obtained in 32% ^{19}F NMR yield.

^{19}F NMR (565 MHz, $CDCl_3$): δ = –57.6 ppm.

The spectroscopic data are consistent to those reported in literature.[22]

11.24 Phenyl 2-(4-(2-methyl-1-(trifluoromethoxy-^{13}C)propyl)phenyl)propanoate ([^{13}C]-2o)

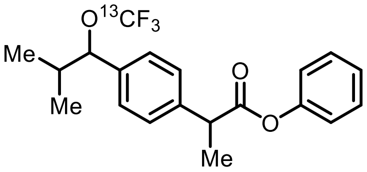

[^{13}C]-**2o**

Phenyl-2-(4-(2-methyl-1-(trifluoromethoxy-^{13}C)propyl)phenyl)propanoate ([^{13}C]-**2o**) was prepared from phenyl 2-(4-isobutylphenyl)propanoate **1o** (145 mg, 513 µmol, 1.00 equiv) and [^{13}C]-BTMP (172 mg, 1.00 mmol, 2.00 equiv) according to **GP3** with a reaction time of 36 h. [^{13}C]-**2o** (75 mg, 204 µmol, 41% (42% ^{19}F NMR yield)) was purified by flash column chromatography (SiO_2, CyH/EtOAc, 99:1) and was isolated as yellowish oil.

^{1}H NMR (600 MHz, $CDCl_3$): δ = 7.40 (d, J = 8.1 Hz, 1H), 7.34 (t, J = 7.9 Hz, 2H), 7.28 (d, J = 8.1 Hz, 1H), 7.20 (t, J = 7.3 Hz, 1H), 7.00 (t, J = 7.6 Hz, 2H), 4.78 (dd, J = 7.1, 3.2 Hz, 1H), 3.98 (q, J = 7.4 Hz, 1H), 2.10 (dhept, J = 13.4, 6.8 Hz, 1H), 1.63 (d, J = 7.2 Hz, 2H), 1.03 (d, J = 6.7 Hz, 2H), 0.84 (d, J = 6.8 Hz, 2H) ppm.
^{13}C{^{1}H} NMR (151 MHz, $CDCl_3$): δ = 173.0, 150.9, 140.0, 138.0, 129.5, 127.6, 127.2, 126.0, 122.0 (q, J = 255 Hz), 121.5, 85.8, 45.5, 34.4, 18.7, 18.5, 18.2 ppm.
^{19}F NMR (376 MHz, $CDCl_3$): δ = –57.8 (d, J = 255 Hz) ppm.
HRMS (EI): m/z calculated for $[C_{19}{}^{13}CH_{21}F_3O_3]^+$ ($[M]^+$): 367.1471, measured: 367.1469.

12. Synthesis of S2, S4, S5, S6, and S8

12.1 2-(4-Ethylphenyl)-4,4,5,5-tetramethyl-1,3,2-dioxaborolane (S2)

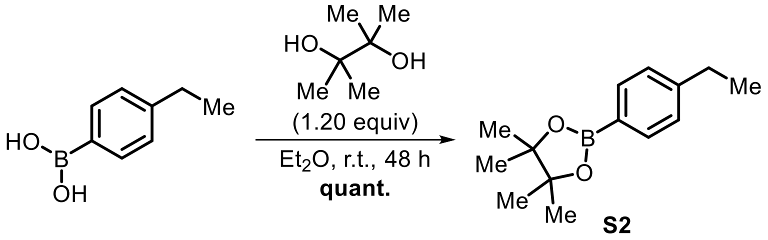

Synthesis of **S2** was performed according to a literature known procedure.[23] A one-necked round-bottom flask was charged with pinacol (945 mg, 8.00 mmol, 1.20 equiv) in Et_2O (13 mL). (4-Ethylphenyl)boronic acid (1.00 g, 6.67 mmol, 1.00 equiv) was added. The solution was allowed to stir for 48 h at r.t. before the solvent was evaporated under reduced pressure. The product was purified by flash column chromatography (SiO_2, CyH/EtOAc, 10:1). Product **S2** (1.55 g, 6.67 mmol, quant.) was isolated as colorless solid.

^{1}H NMR (400 MHz, $CDCl_3$): δ = 7.74 (d, *J* = 8.0 Hz, 2H), 7.22 (d, *J* = 8.2 Hz, 2H), 2.66 (q, *J* = 7.6 Hz, 2H), 1.34 (s, 12H), 1.24 (t, *J* = 7.6 Hz, 3H) ppm.
^{13}C{^{1}H} NMR (176 MHz, $CDCl_3$): δ = 147.9, 135.0, 127.5, 83.8, 29.3, 25.0, 15.6 ppm.

The spectroscopic data are consistent to those reported in literature.[23]

12.2 *N*-(4'-Ethyl-[1,1'-biphenyl]-4-yl)acetamide (S4)

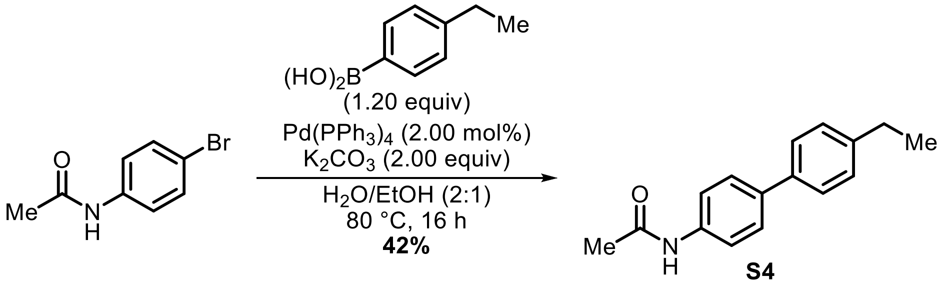

N-(4'-Ethyl-[1,1'-biphenyl]-4-yl)acetamide **S4** was prepared from *N*-(4-bromophenyl)acetamide (1.00 g, 4.67 mmol, 1.00 equiv), (4-ethylphenyl)boronic acid (919 mg, 5.60 mmol, 1.20 equiv), $Pd(PPh_3)_4$ (108 mg, 93.0 µmol, 2.00 mol%), K_2CO_3 (1.29 g, 9.34 mmol, 2.00 equiv), H_2O (12.8 mL), and EtOH (6.4 mL) using **GP1** with a reaction time of 16 h. Purified by flash column chromatography (SiO_2, EtOAc/CyH, 60%). **S4** (463 mg, 1.93 mmol, 42%) was isolated as yellowish solid.

^{1}H NMR (400 MHz, $CDCl_3$): δ = 7.58 – 7.42 (m, 7H), 7.24 (d, J = 2.7 Hz, 2H), 2.68 (q, J = 7.6 Hz, 2H), 2.19 (s, 3H), 1.26 (t, J = 7.6 Hz, 3H) ppm.
^{13}C{^{1}H} NMR (151 MHz, $CDCl_3$): δ = 168.5, 143.4, 138.0, 137.3, 137.0, 128.4, 127.6, 126.9, 120.3, 28.6, 24.8, 15.7 ppm.

The spectroscopic data are consistent to those reported in literature.[24]

12.3 4-(4-Phenoxyphenyl)butan-2-one (S5)

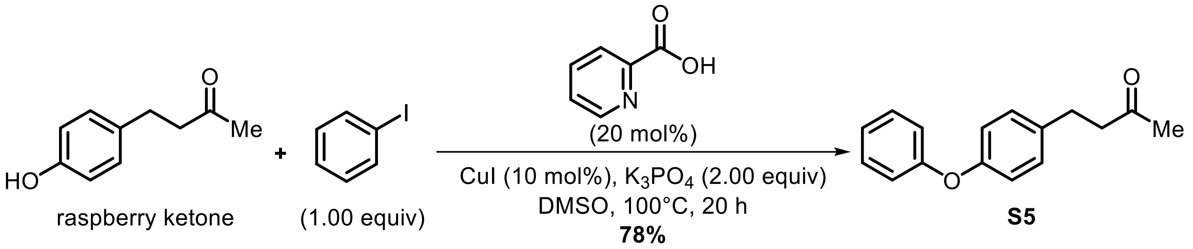

Synthesis of **S5** was performed according to a literature known procedure.[25] A 50 mL Schlenk flask was charged with CuI (187 mg, 980 µmol, 10 mol%), picolinic acid (241 mg, 1.97 mmol, 20 mol%), iodobenzene (2.00 g, 1.10 mL, 9.80 mmol, 1.00 equiv), K_3PO_4 (4.16 g, 19.6 mmol, 2.00 equiv), 4-(4-hydroxyphenyl)butan-2-one (raspberry ketone, 1.93 g, 11.8 mmol, 1.20 equiv) and DMSO (20 mL) under argon atmosphere. The mixture was stirred for 20 h at 100 °C before it was cooled to room temperature and diluted with a 1:1 mixture of NH_4Cl solution (sat., aq., 100 mL) and water (100 mL). The mixture was extracted with CH_2Cl_2 (2 × 100 mL) and chloroform (100 mL). The combined organic phases were washed with NaOH solution (5 w%, aq., 100 mL) and brine (100 mL), dried over anhydrous $MgSO_4$, filtered and the solvent was evaporated under reduced pressure. The product was purified by flash column chromatography (SiO_2, CyH/EtOAc, 6:1) and **S5** (1.83 g, 7.62 mmol, 78%) was isolated as yellowish oil.

^{1}H NMR (400 MHz, $CDCl_3$): δ = 7.35 – 7.29 (m, 2H), 7.17 – 7.13 (m, 2H), 7.11 – 7.06 (m, 1H), 7.01 – 6.97 (m, 2H), 6.96 – 6.91 (m, 2H), 2.91 – 2.85 (m, 2H), 2.79 – 2.74 (m, 2H), 2.15 (s, 3H) ppm.
^{13}C{^{1}H} NMR (151 MHz, $CDCl_3$): δ = 208.2, 157.8, 155.7, 136.2, 130, 129.8, 123.3, 119.4, 118.9, 45.5, 30.4, 29.3 ppm.

The spectroscopic data fits to those reported in literature.[25]

12.4 (*E*)-4-(4-Phenoxyphenyl)but-3-en-2-one (S6)

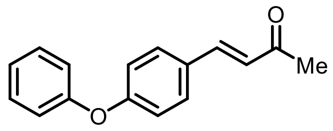

S6

(*E*)-4-(4-Phenoxyphenyl)but-3-en-2-one (**S6**) was prepared from 4-(4-phenoxy-phenyl)butan-2-one (**S5**) (120 mg, 500 µmol, 1.00 equiv) according to **GP3** with a reaction time of 18 h. Purified by flash column chromatography (SiO_2, EtOAc/CyH, 1%). **S6** (27 mg, 113 µmol, 23%) was isolated as yellowish oil.

^{1}H NMR (400 MHz, $CDCl_3$): δ = 7.51 (d, *J* = 8.5 Hz, 2H), 7.49 (d, *J* = 15.8 Hz, 1H), 7.40 – 7.36 (m, 2H), 7.20 – 7.15 (m, 1H), 7.08 – 7.04 (m, 2H), 7.01 – 6.98 (m, 2H), 6.64 (d, *J* = 16.3 Hz, 1H), 2.38 (s, 3H) ppm.
^{13}C{^{1}H} NMR (151 MHz, $CDCl_3$): δ = 198.7, 160.1, 156.3, 143.1, 130.3, 129.4, 126.3, 124.6, 120.1, 119.4, 118.8, 27.8 ppm.
HRMS (ESI): *m/z* calculated for $[C_{16}H_{14}O_2Na]^+$ $[M+Na]^+$: 261.0886; found: 261.0919.

12.5 4-(((8*R*,9*S*,13*S*,14*S*)-13-Methyl-17-oxo-7,8,9,11,12,13,14,15,16,17-decahydro-6*H*-cyclopenta[*a*]phenanthren-3-yl)oxy)benzaldehyde (S10)

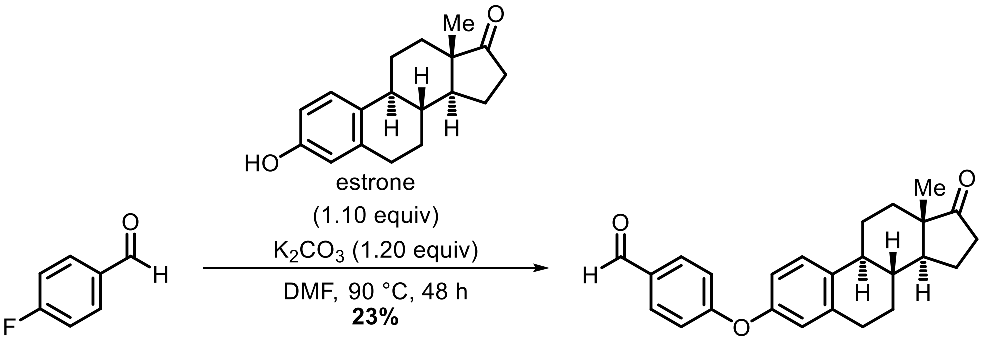

S10

Synthesis of **S10** was performed according to a literature known procedure.[26] 4-Fluorobenzaldehyde (200 mg, 0.172 mL, 1.61 mmol, 1.00 equiv), estrone (479 mg, 1.77 mmol, 1.10 equiv) and K_2CO_3 (267 mg, 1.93 mmol, 1.20 equiv) were placed in a heat gun-dried Schlenk flask under argon atmosphere. DMF (11 mL) was added and the mixture was heated to 90 °C. The mixture was stirred at this temperature for 48 h before it was cooled to room temperature, diluted with CH_2Cl_2 and washed with water.

The two phases were separated, the organic phase was washed with brine and dried over Na_2SO_4. After filtration, the solvent was evaporated under reduced pressure. The product was purified by flash column chromatography (SiO_2, CyH/EtOAc, 5:1). Product **S10** (139 mg, 371 µmol, 23%) was isolated as a yellowish solid.

^{1}H NMR (500 MHz, $CDCl_3$): δ = 9.85 (s, 1H), 7.78 – 7.75 (m, 2H), 7.25 (d, *J* = 9.7 Hz, 1H), 7.00 – 6.97 (m, 2H), 6.80 (dd, *J* = 8.5, 2.6 Hz, 1H), 6.76 (dd, *J* = 2.4, 1.2 Hz, 1H), 2.86 – 2.82 (m, 2H), 2.46 (dd, *J* = 20.0, 8.7 Hz, 1H), 2.39 – 2.34 (m, 1H), 2.31 – 2.19 (m, 1H), 2.14 – 2.06 (m, 1H), 2.04 – 1.95 (m, 2H), 1.61 – 1.38 (m, 7H), 0.87 (s, 3H) ppm.
^{13}C{^{1}H} NMR (151 MHz, $CDCl_3$): δ = 221.1, 191.1, 163.9, 153.2, 139.1, 136.9, 132.3, 131.4, 127.4, 120.8, 118.1, 117.7, 50.8, 48.3, 44.5, 38.5, 36.2, 31.9, 29.8, 26.7, 26.2, 21.9, 14.2 ppm.
HRMS (ESI, pos): m/z calculated for $[C_{25}H_{27}O_3]^+$ ($[M+H]^+$): 375.1955, measured: 375.1956.

13 Limitations

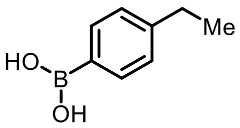

S1

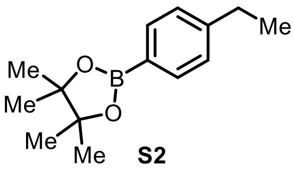

S2

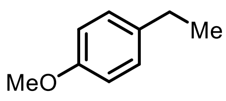

S3

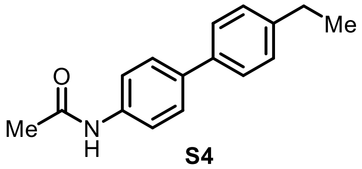

S4

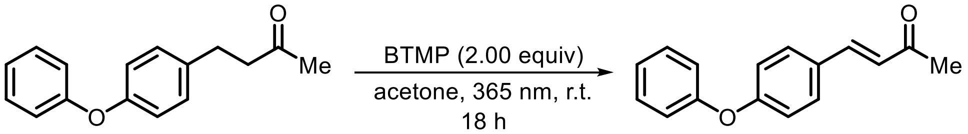

S5
raspberry ketone derivative

S6

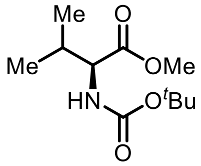

S7
L-valine derivative

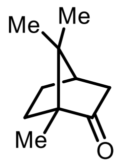

S8
camphor

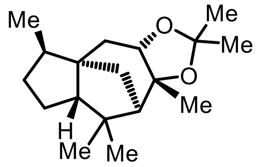

S9
ambrocenide

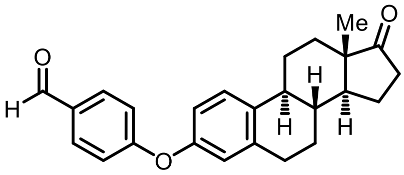

S10
estrone derivative

14. X-ray Data of (3a*R*,8*S*,9a*S*)-3a,6,6,9a-Tetramethyl-8-(trifluoromethoxy)decahydronaphtho[2,1-b]furan-2(1*H*)-one (4e)

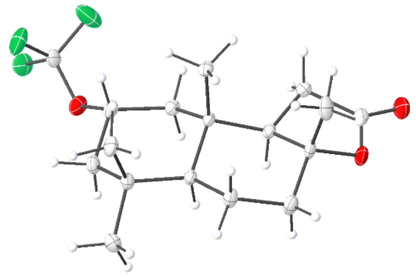

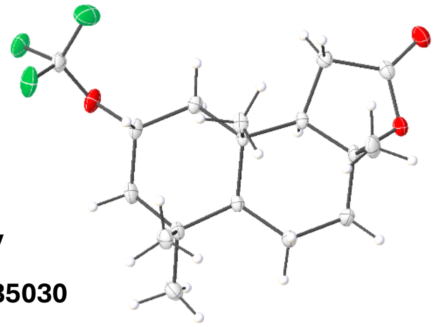

X-ray

CCDC 2435030

Table S7. Crystal data of 4e (CCDC 2435030)

Identification code	compound **4e** (CCDC 2435030)
Empirical formula	$C_{17}H_{25}F_3O_3$
Formula weight	334.381
Temperature/K	150.00
Crystal system	orthorhombic
Space group	$P2_12_12_1$
a/Å	8.3909(1)
b/Å	13.6555(1)
c/Å	14.4806(1)
α/°	90
β/°	90
γ/°	90
Volume/Å^3	1659.22(3)
Z	4
ρ_{calc}g/cm^3	1.339
μ/mm^{-1}	0.952
F(000)	714.8
Crystal size/mm^3	0.58 × 0.39 × 0.2
Radiation	Cu Kα (λ = 1.54178)
2Θ range for data collection/°	12.2 to 149.36
Index ranges	-10 ≤ h ≤ 10, -17 ≤ k ≤ 17, -18 ≤ l ≤ 16
Reflections collected	21856
Independent reflections	3322 [R_{int} = 0.0644, R_{sigma} = 0.0521]
Data/restraints/parameters	3322/0/212
Goodness-of-fit on F^2	0.533
Final R indexes [I>=2σ (I)]	R_1 = 0.0631, wR_2 = 0.1563
Final R indexes [all data]	R_1 = 0.0635, wR_2 = 0.1570
Largest diff. peak/hole / e Å^{-3}	0.67/-0.46
Flack parameter	0.10(5)

15. NMR and HRMS Spectra of Synthesized Substrates

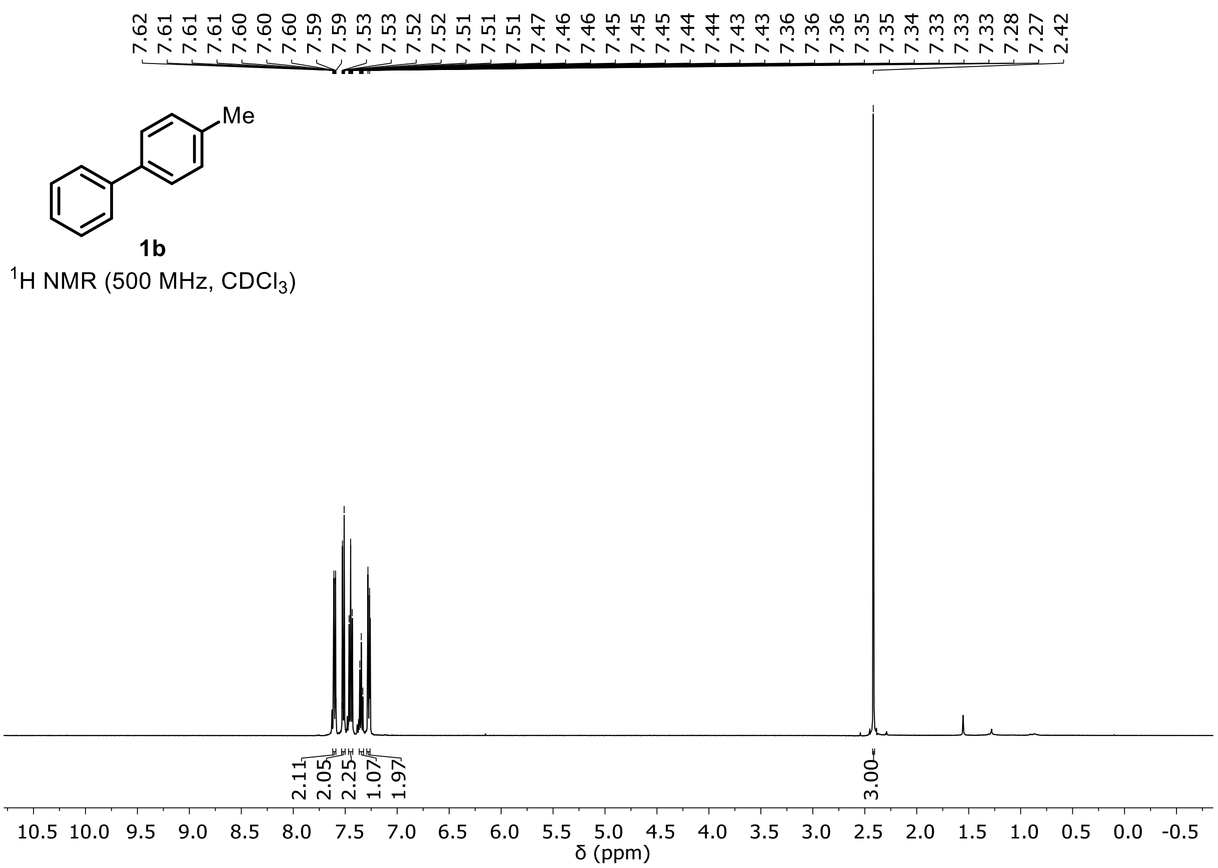

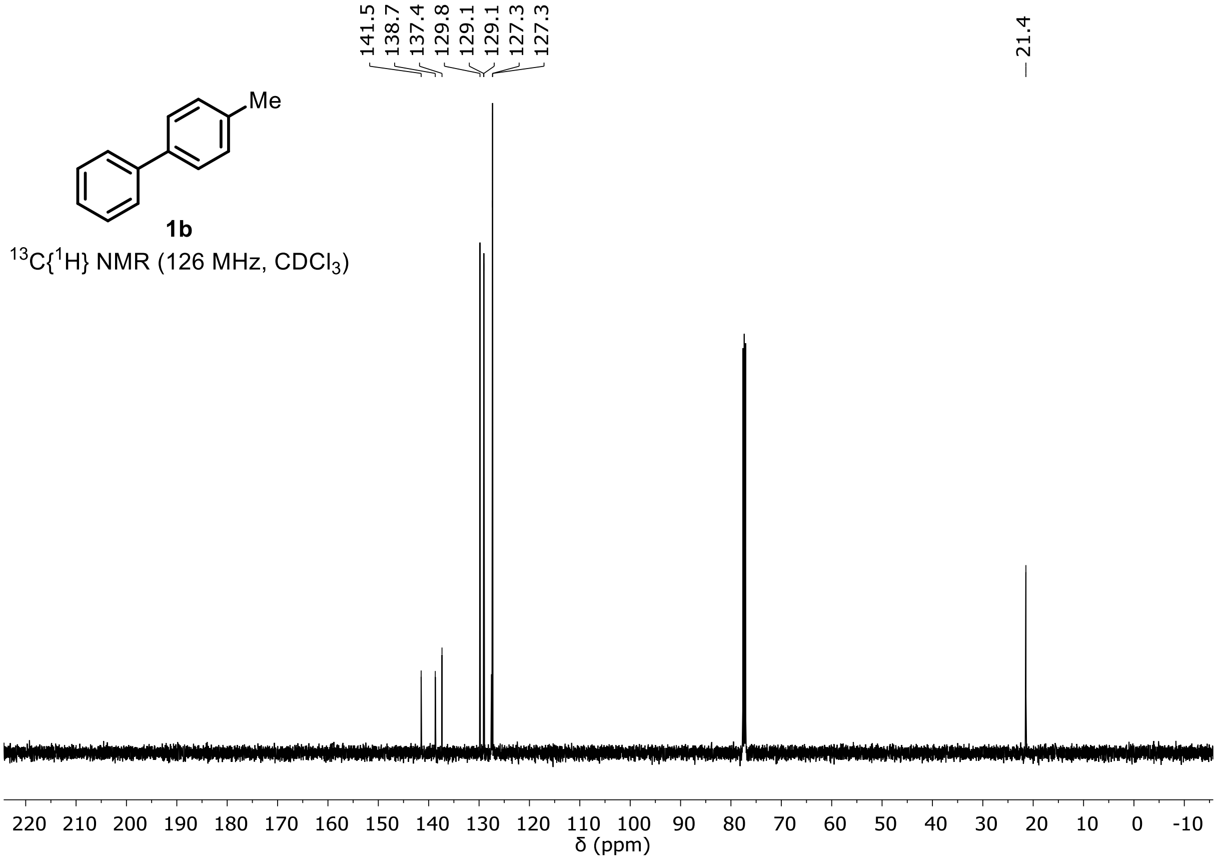

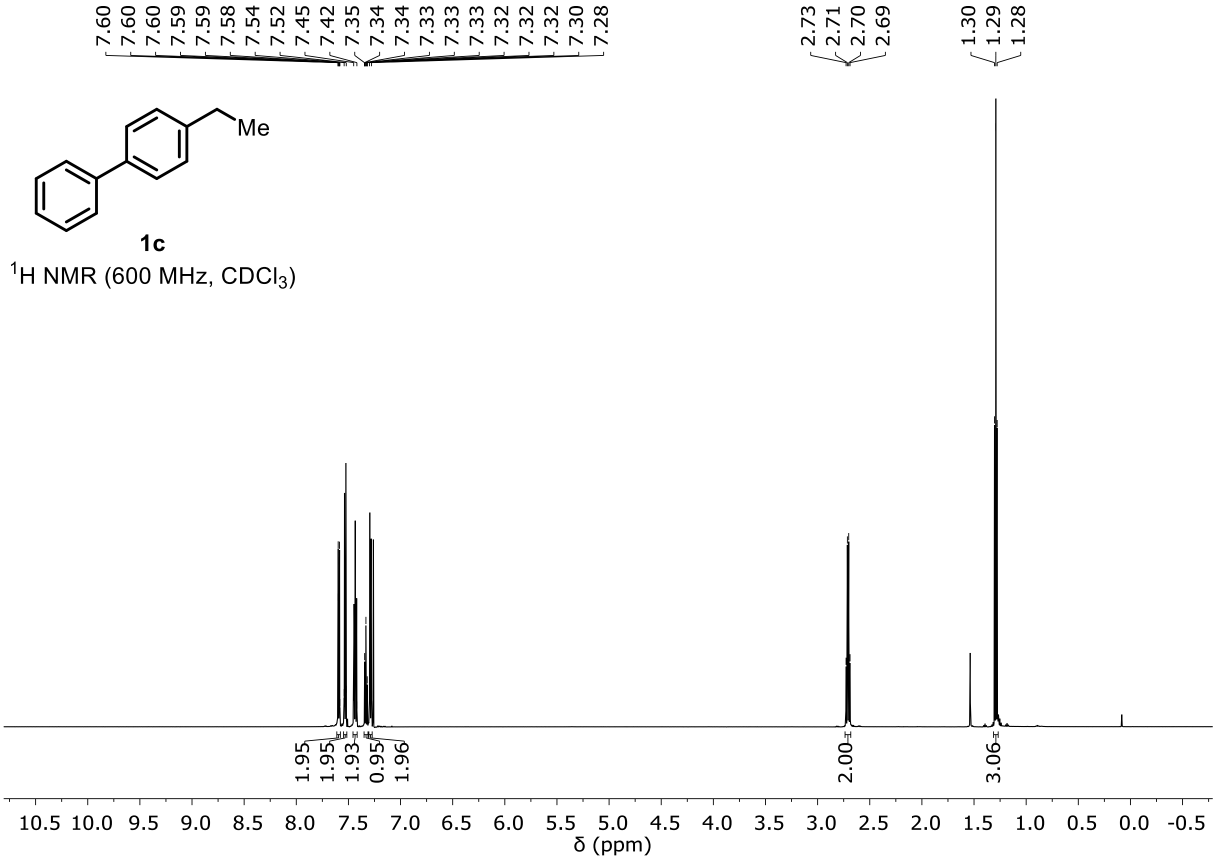

1c

^{1}H NMR (600 MHz, $CDCl_3$)

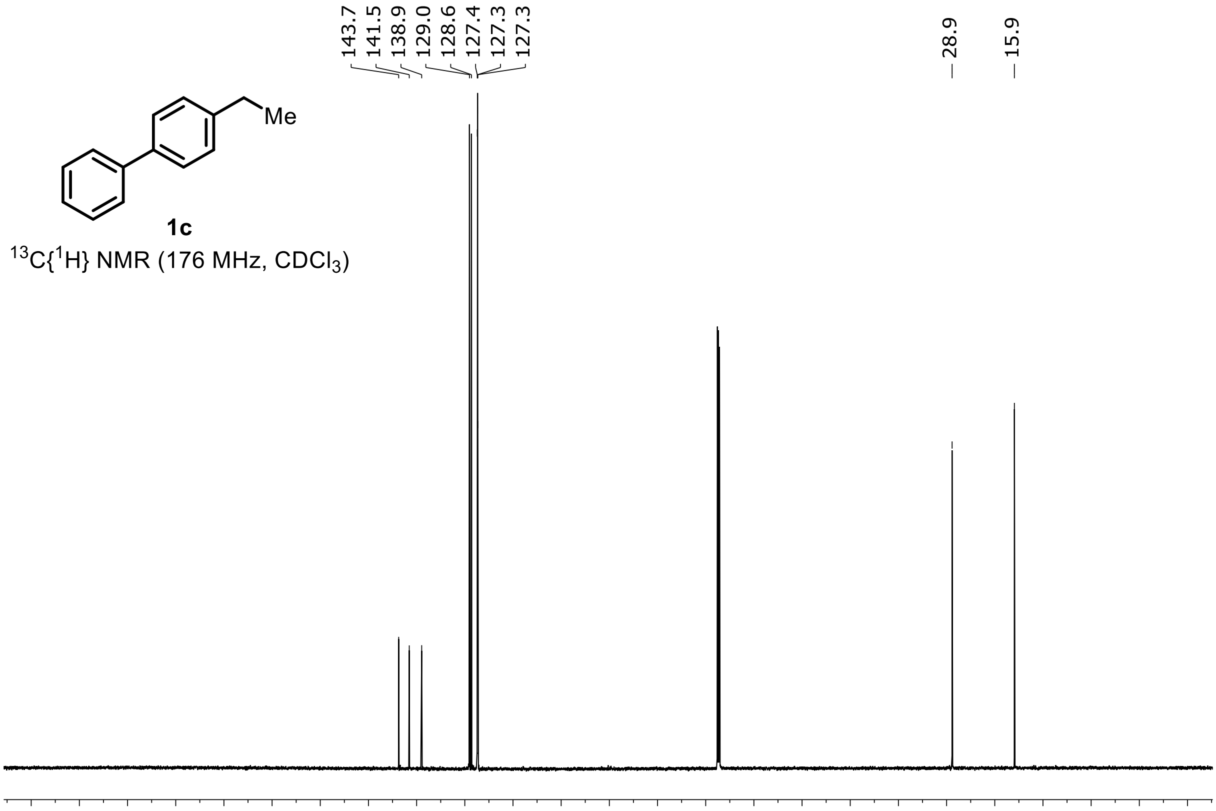

1c

$^{13}C\{^{1}H\}$ NMR (176 MHz, $CDCl_3$)

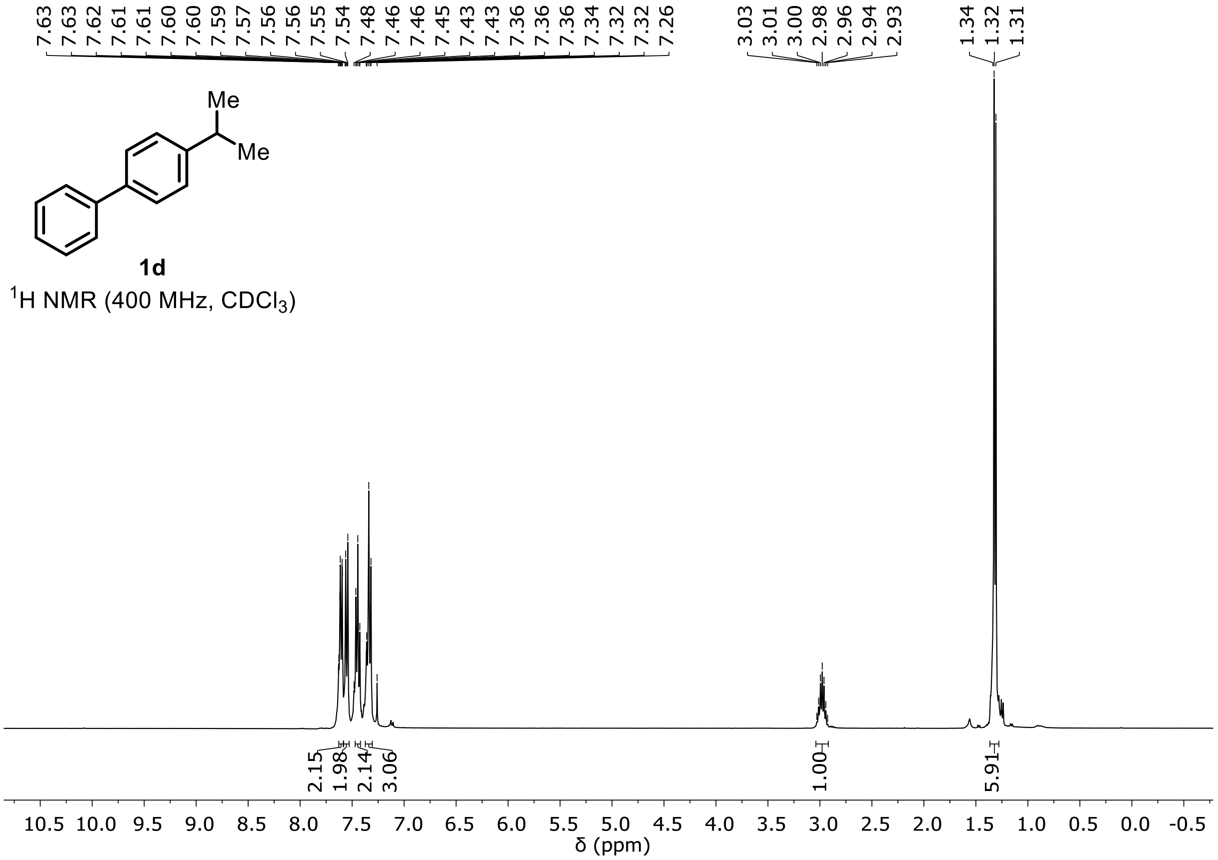
7.63
7.63
7.62
7.61
7.61
7.60
7.60
7.59
7.57
7.56
7.56
7.55
7.54
7.48
7.46
7.46
7.45
7.43
7.43
7.36
7.36
7.36
7.34
7.32
7.32
7.26
3.03
3.01
3.00
2.98
2.96
2.94
2.93
1.34
1.32
1.31
Me
Me
1d
1H NMR (400 MHz, CDCl3)
2.15
1.98
2.14
3.06
1.00
5.91
10.5 10.0 9.5 9.0 8.5 8.0 7.5 7.0 6.5 6.0 5.5 5.0 4.5 4.0 3.5 3.0 2.5 2.0 1.5 1.0 0.5 0.0 -0.5
δ (ppm)

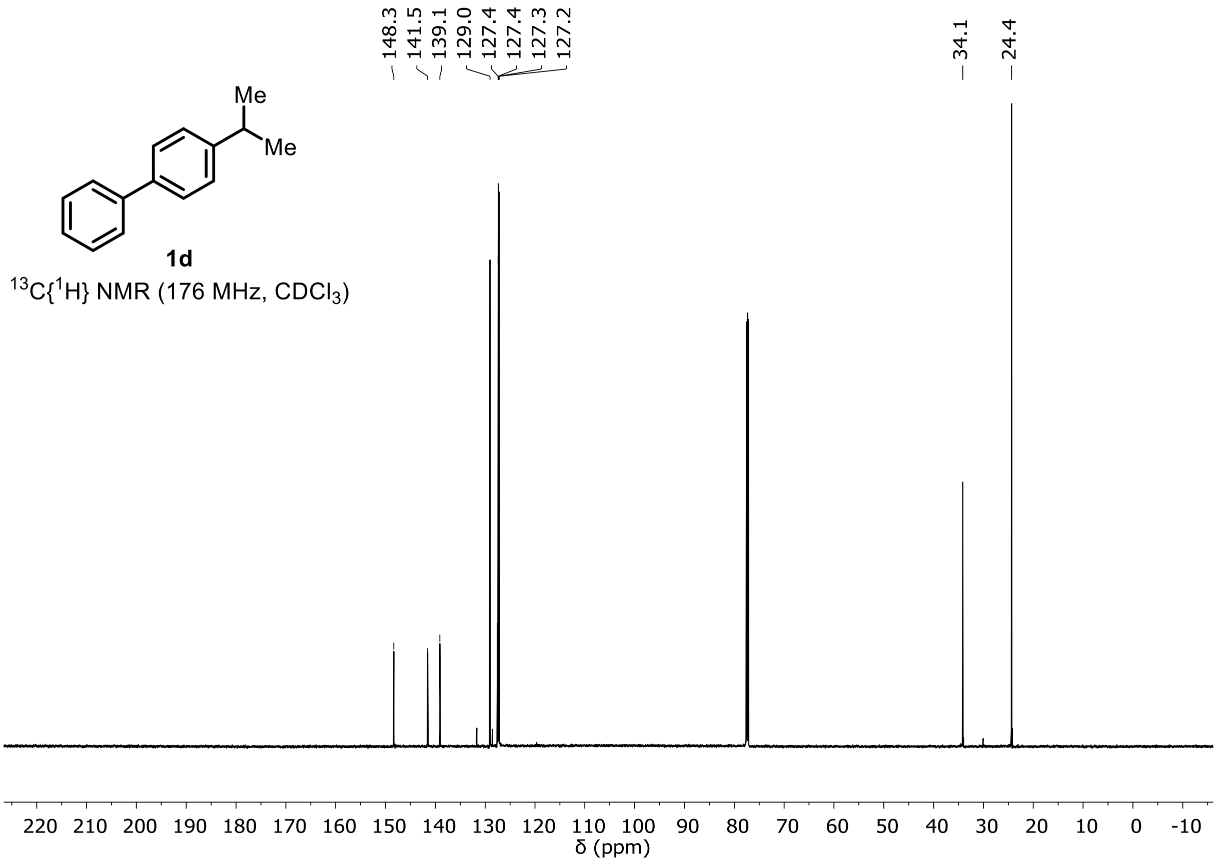
148.3
141.5
139.1
129.0
127.4
127.4
127.3
127.2
34.1
24.4
Me
Me
1d
13C{1H} NMR (176 MHz, CDCl3)
220 210 200 190 180 170 160 150 140 130 120 110 100 90 80 70 60 50 40 30 20 10 0 -10
δ (ppm)

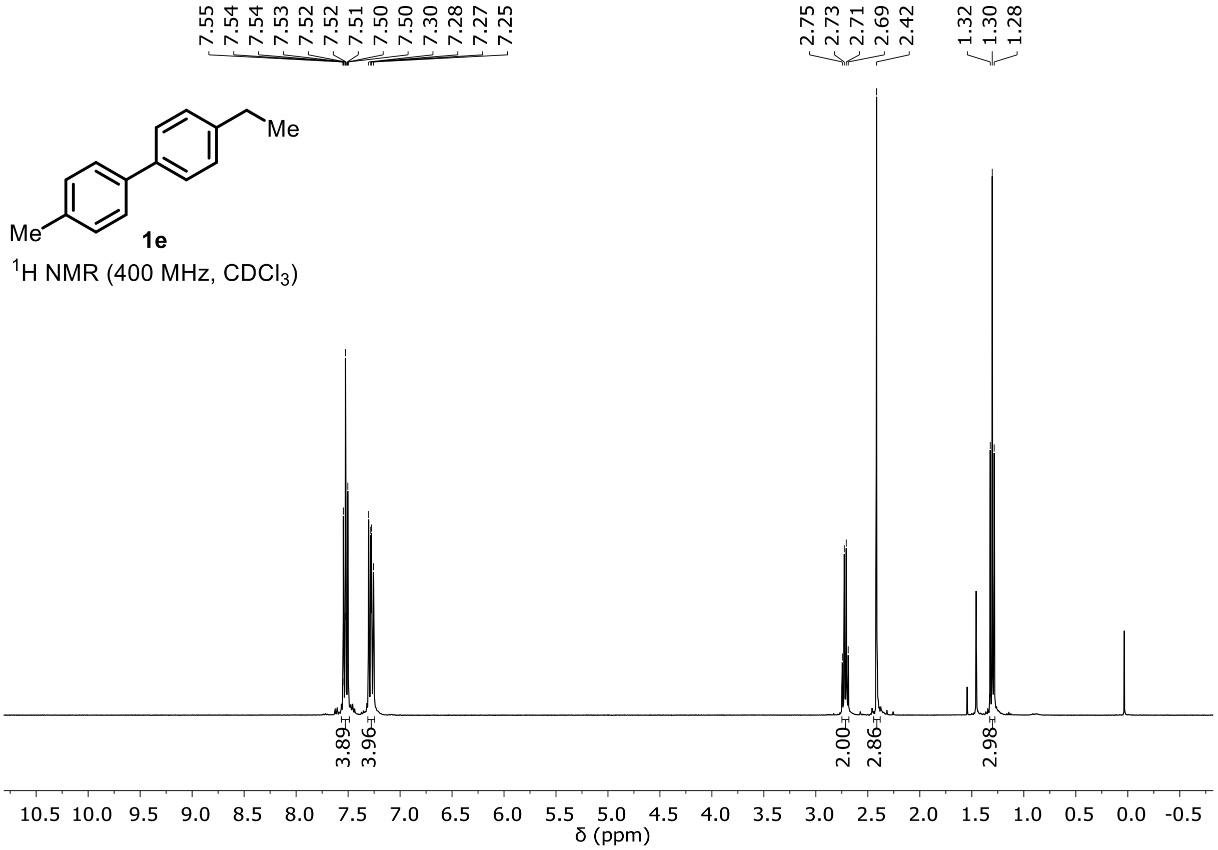
7.55 7.54 7.54 7.53 7.52 7.52 7.51 7.50 7.50 7.30 7.28 7.27 7.25
2.75 2.73 2.71 2.69 2.42
1.32 1.30 1.28
Me
Me
1e
^{1}H NMR (400 MHz, $CDCl_3$)
3.89 3.96 2.00 2.86 2.98
10.5 10.0 9.5 9.0 8.5 8.0 7.5 7.0 6.5 6.0 5.5 5.0 4.5 4.0 3.5 3.0 2.5 2.0 1.5 1.0 0.5 0.0 -0.5
δ (ppm)

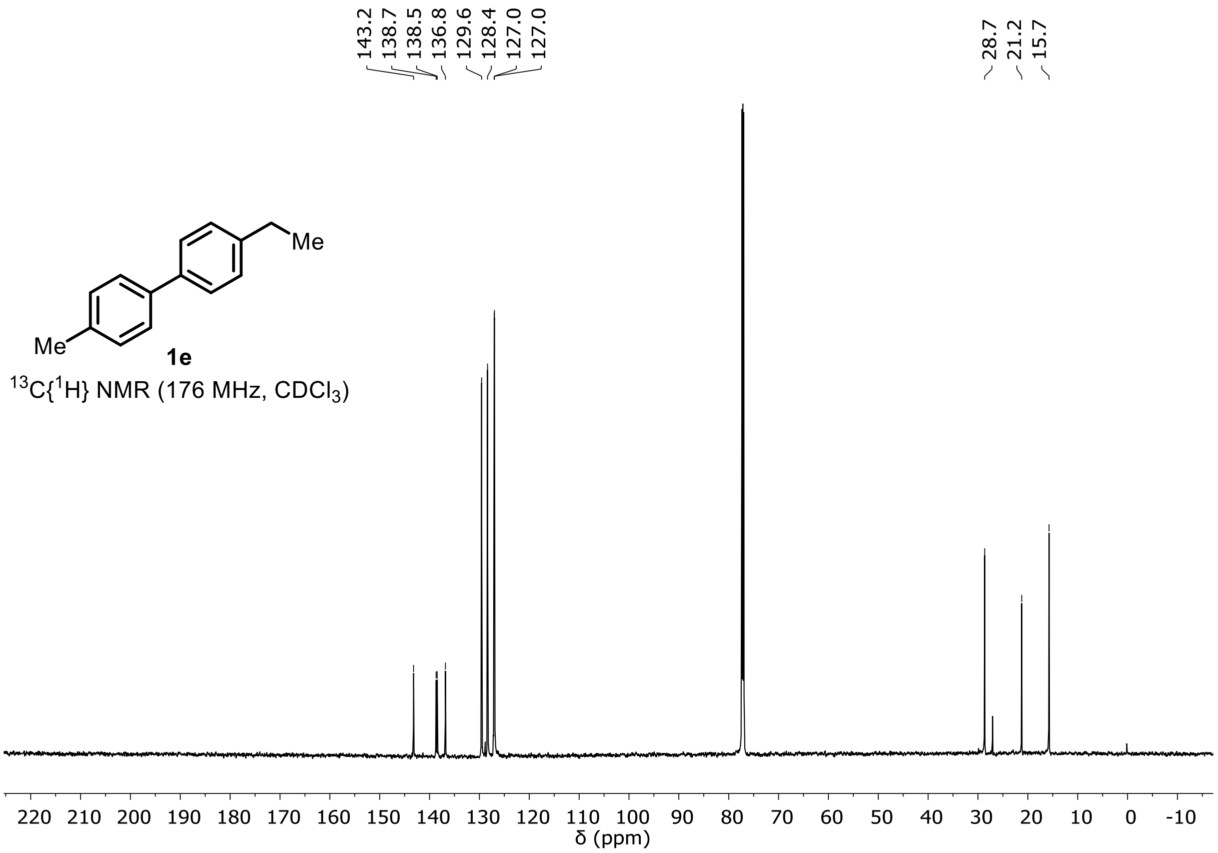
143.2 138.7 138.5 136.8 129.6 128.4 127.0 127.0
28.7 21.2 15.7
Me
Me
1e
^{13}C{^{1}H} NMR (176 MHz, $CDCl_3$)
220 210 200 190 180 170 160 150 140 130 120 110 100 90 80 70 60 50 40 30 20 10 0 -10
δ (ppm)

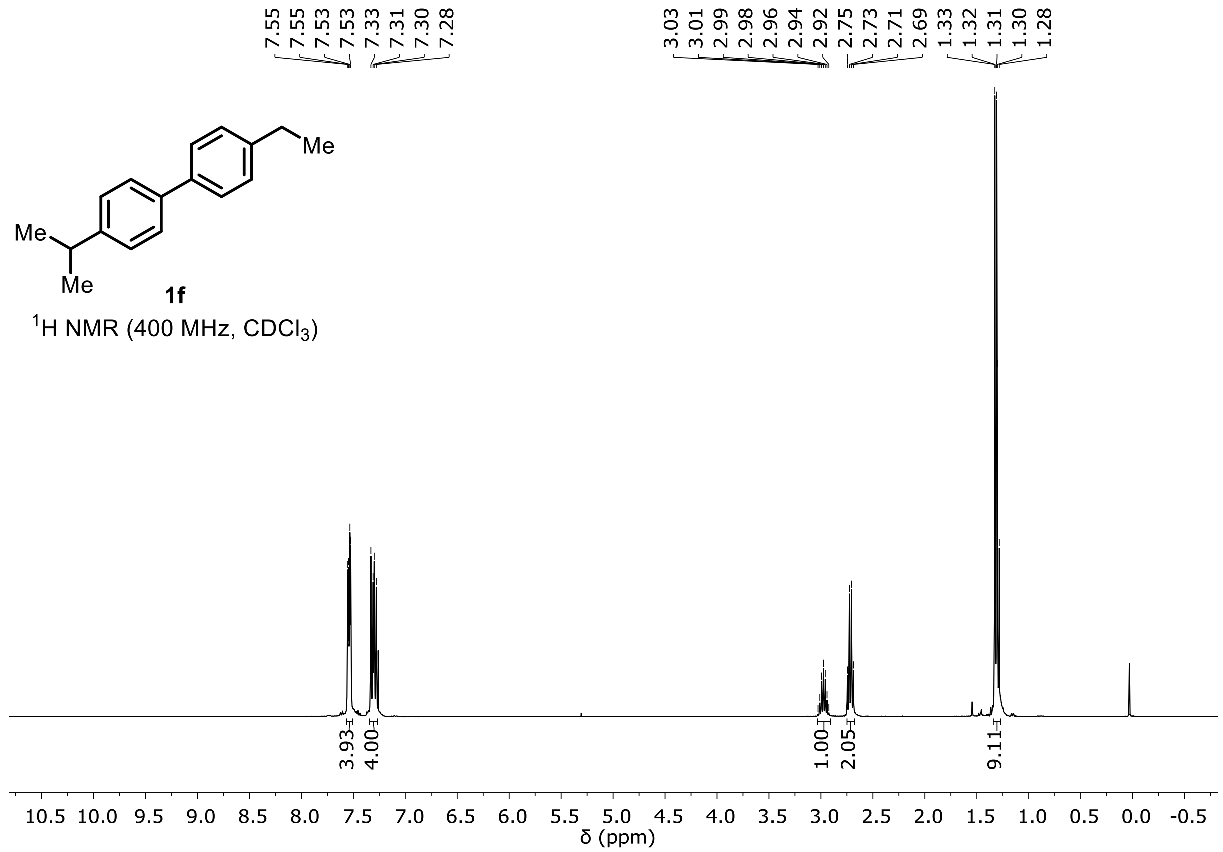
7.55 7.55 7.53 7.53 7.33 7.31 7.30 7.28
3.03 3.01 2.99 2.98 2.96 2.94 2.92 2.75 2.73 2.71 2.69 1.33 1.32 1.31 1.30 1.28
Me
Me
Me
1f
^{1}H NMR (400 MHz, CDCl$_3$)
3.93
4.00
1.00
2.05
9.11
10.5 10.0 9.5 9.0 8.5 8.0 7.5 7.0 6.5 6.0 5.5 5.0 4.5 4.0 3.5 3.0 2.5 2.0 1.5 1.0 0.5 0.0 -0.5
δ (ppm)

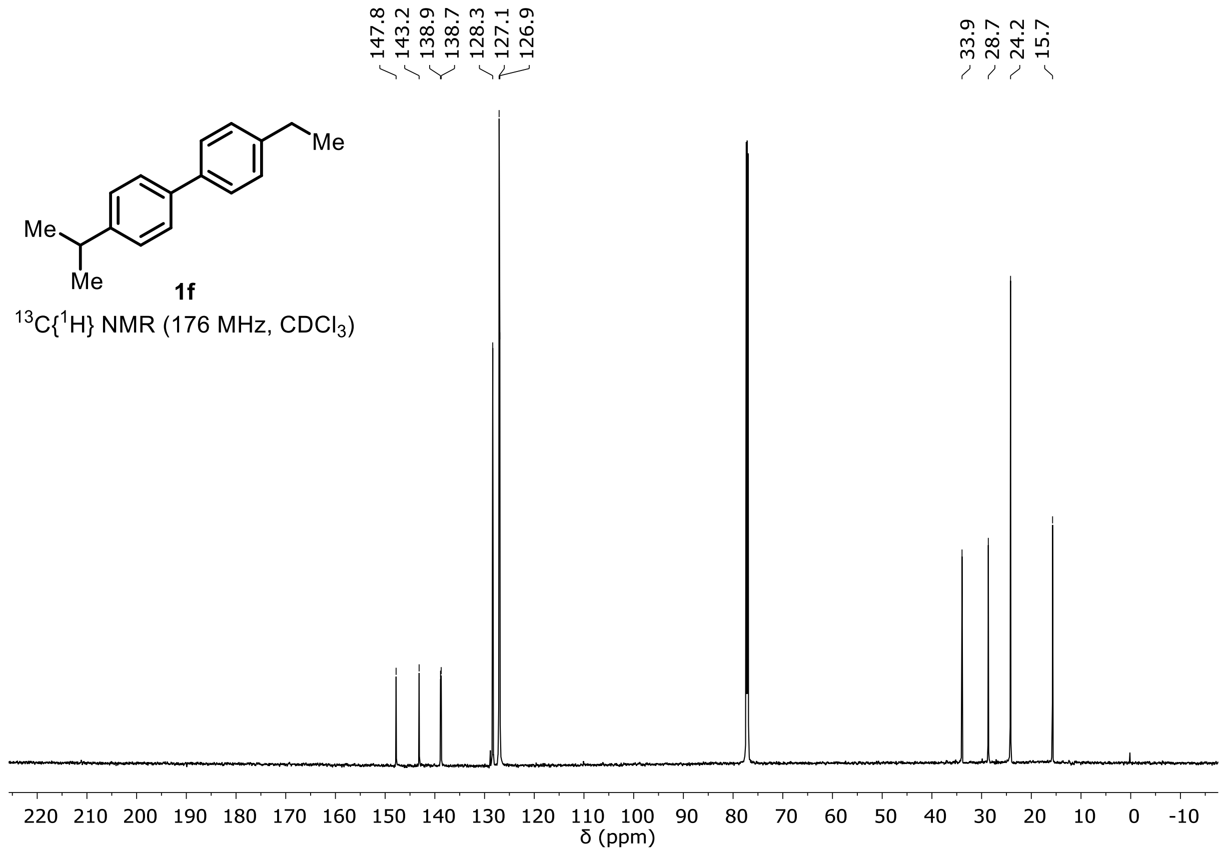
147.8 143.2 138.9 138.7 128.3 127.1 126.9
33.9 28.7 24.2 15.7
Me
Me
Me
1f
^{13}C{^{1}H} NMR (176 MHz, CDCl$_3$)
220 210 200 190 180 170 160 150 140 130 120 110 100 90 80 70 60 50 40 30 20 10 0 -10
δ (ppm)

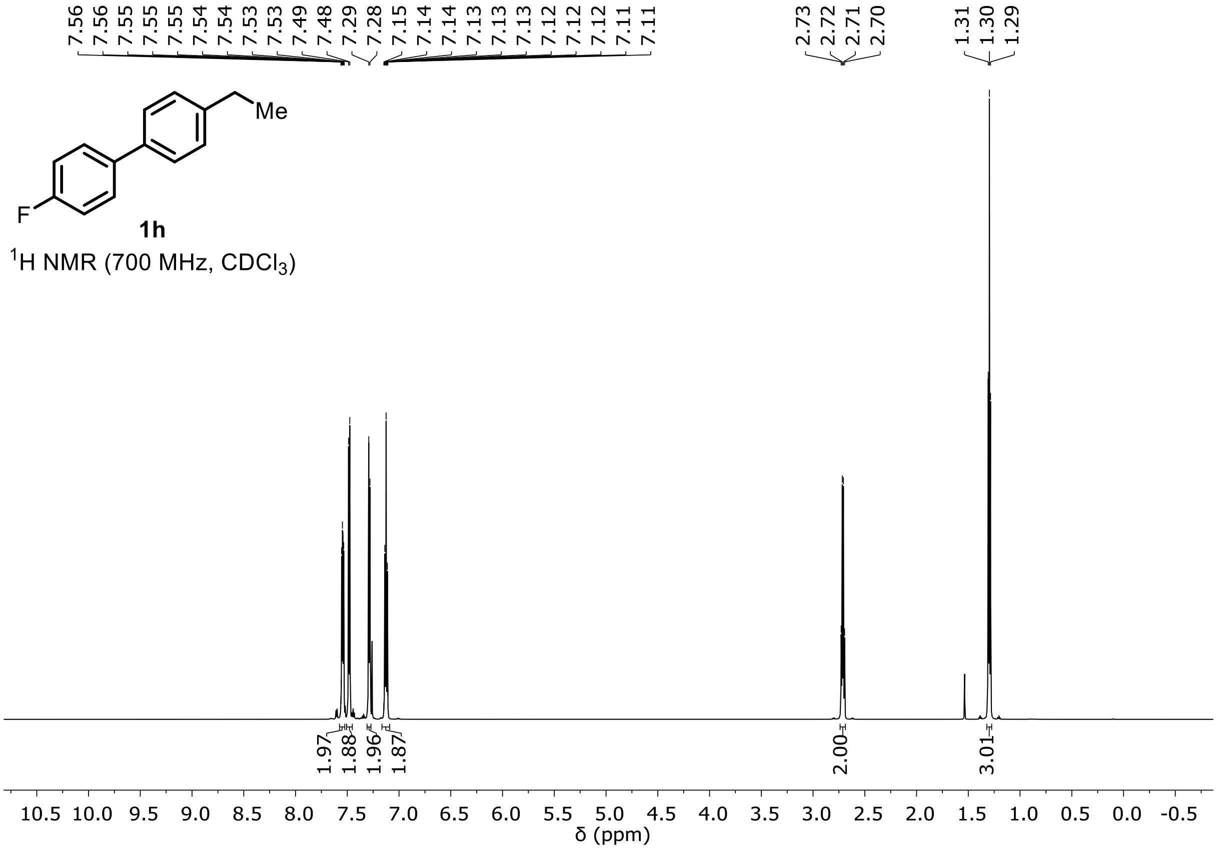
7.56 7.56 7.55 7.55 7.55 7.54 7.54 7.53 7.53 7.49 7.48 7.29 7.28 7.15 7.14 7.14 7.13 7.13 7.13 7.12 7.12 7.12 7.11 7.11
2.73 2.72 2.71 2.70
1.31 1.30 1.29
Me
F
1h
1H NMR (700 MHz, CDCl3)
1.97 1.88 1.96 1.87
2.00
3.01
10.5 10.0 9.5 9.0 8.5 8.0 7.5 7.0 6.5 6.0 5.5 5.0 4.5 4.0 3.5 3.0 2.5 2.0 1.5 1.0 0.5 0.0 -0.5
δ (ppm)

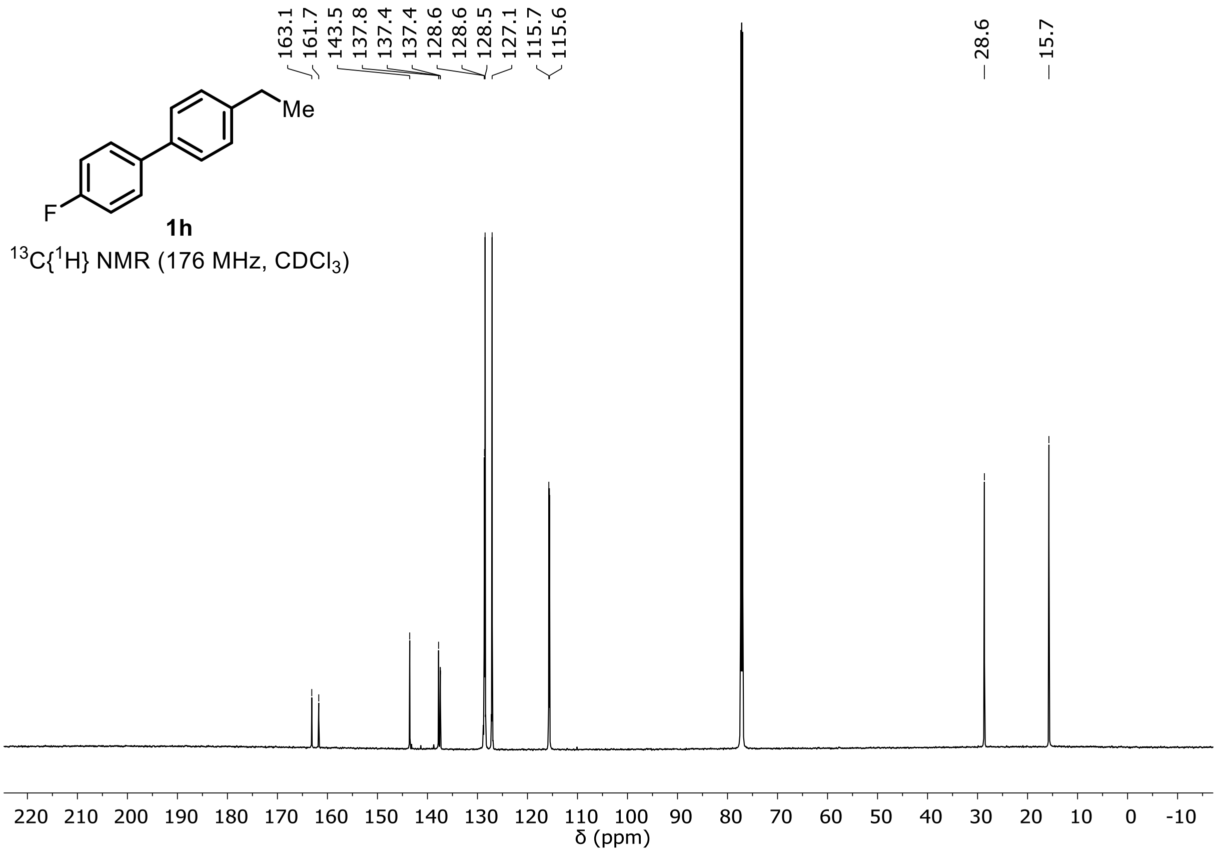
163.1 161.7 143.5 137.8 137.4 137.4 128.6 128.6 128.5 127.1 115.7 115.6
28.6
15.7
Me
F
1h
13C{1H} NMR (176 MHz, CDCl3)
220 210 200 190 180 170 160 150 140 130 120 110 100 90 80 70 60 50 40 30 20 10 0 -10
δ (ppm)

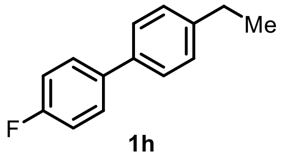

1h

^{19}F NMR (565 MHz, $CDCl_3$)

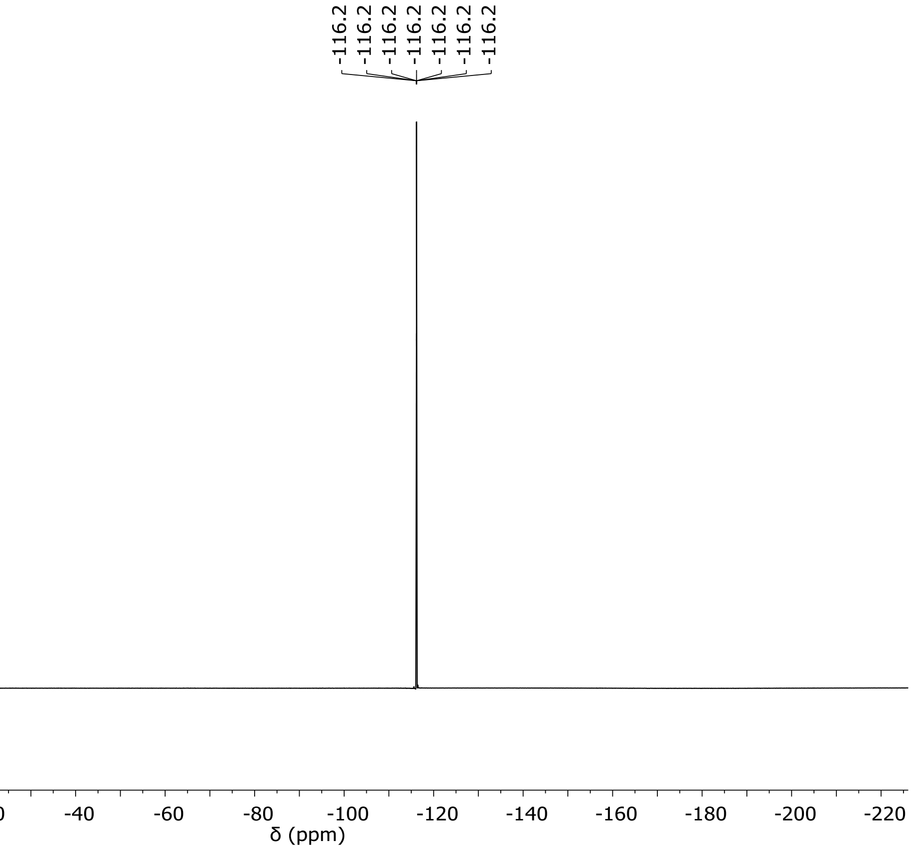

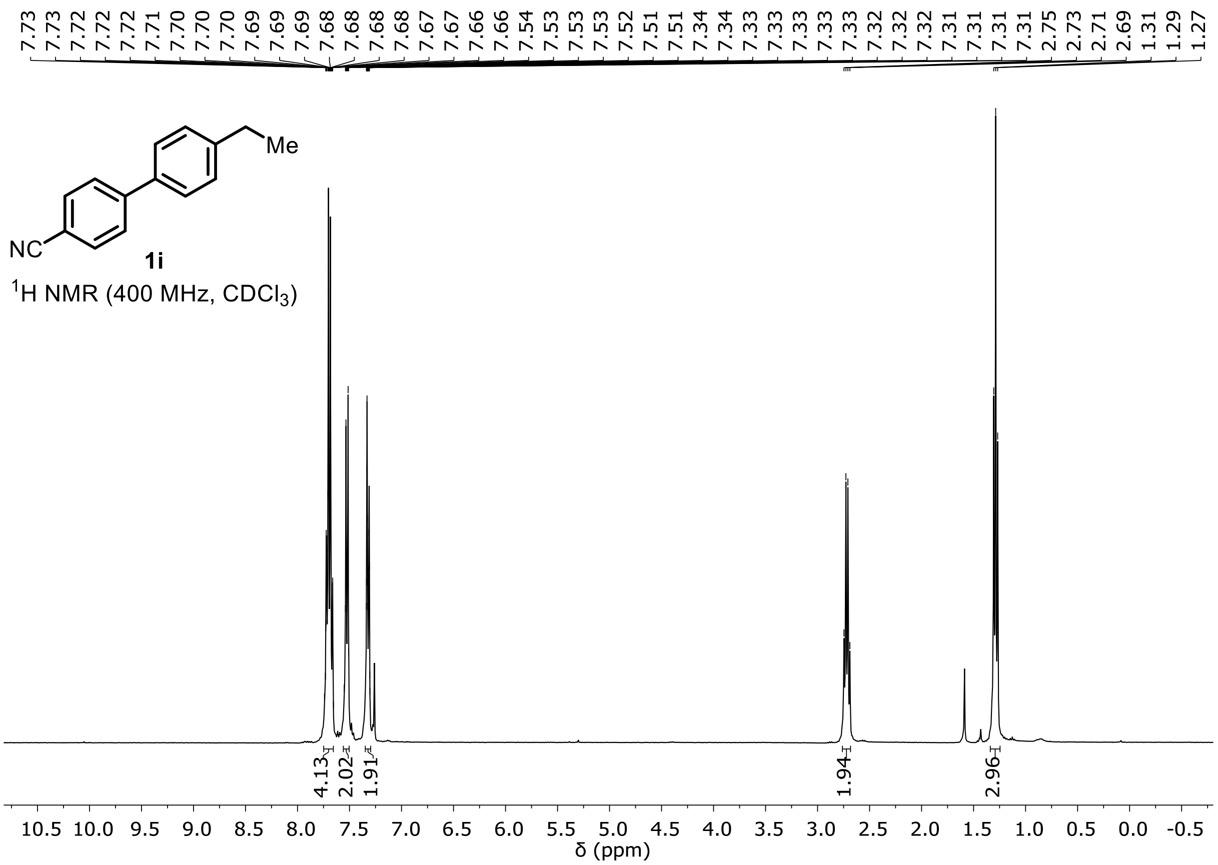
7.73 7.73 7.72 7.72 7.72 7.71 7.70 7.70 7.70 7.69 7.69 7.69 7.68 7.68 7.68 7.68 7.67 7.67 7.66 7.66 7.54 7.53 7.53 7.53 7.52 7.51 7.51 7.34 7.34 7.33 7.33 7.33 7.33 7.33 7.32 7.32 7.32 7.31 7.31 7.31 7.31 2.75 2.73 2.71 2.69 1.31 1.29 1.27
Me
NC
1i
^{1}H NMR (400 MHz, $CDCl_3$)
4.13
2.02
1.91
1.94
2.96
10.5 10.0 9.5 9.0 8.5 8.0 7.5 7.0 6.5 6.0 5.5 5.0 4.5 4.0 3.5 3.0 2.5 2.0 1.5 1.0 0.5 0.0 -0.5
δ (ppm)

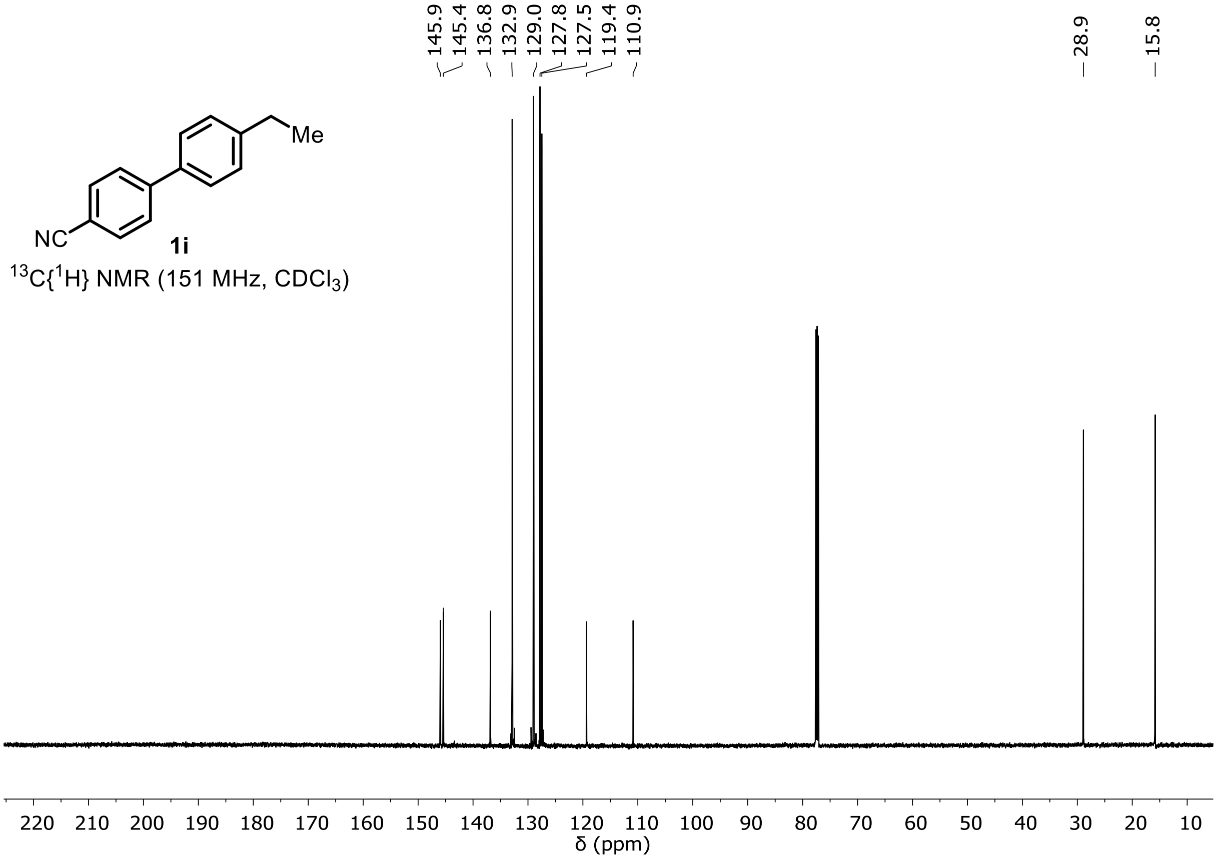
145.9 145.4 136.8 132.9 129.0 127.8 127.5 119.4 110.9
28.9
15.8
Me
NC
1i
$^{13}C\{^1H\}$ NMR (151 MHz, $CDCl_3$)
220 210 200 190 180 170 160 150 140 130 120 110 100 90 80 70 60 50 40 30 20 10
δ (ppm)

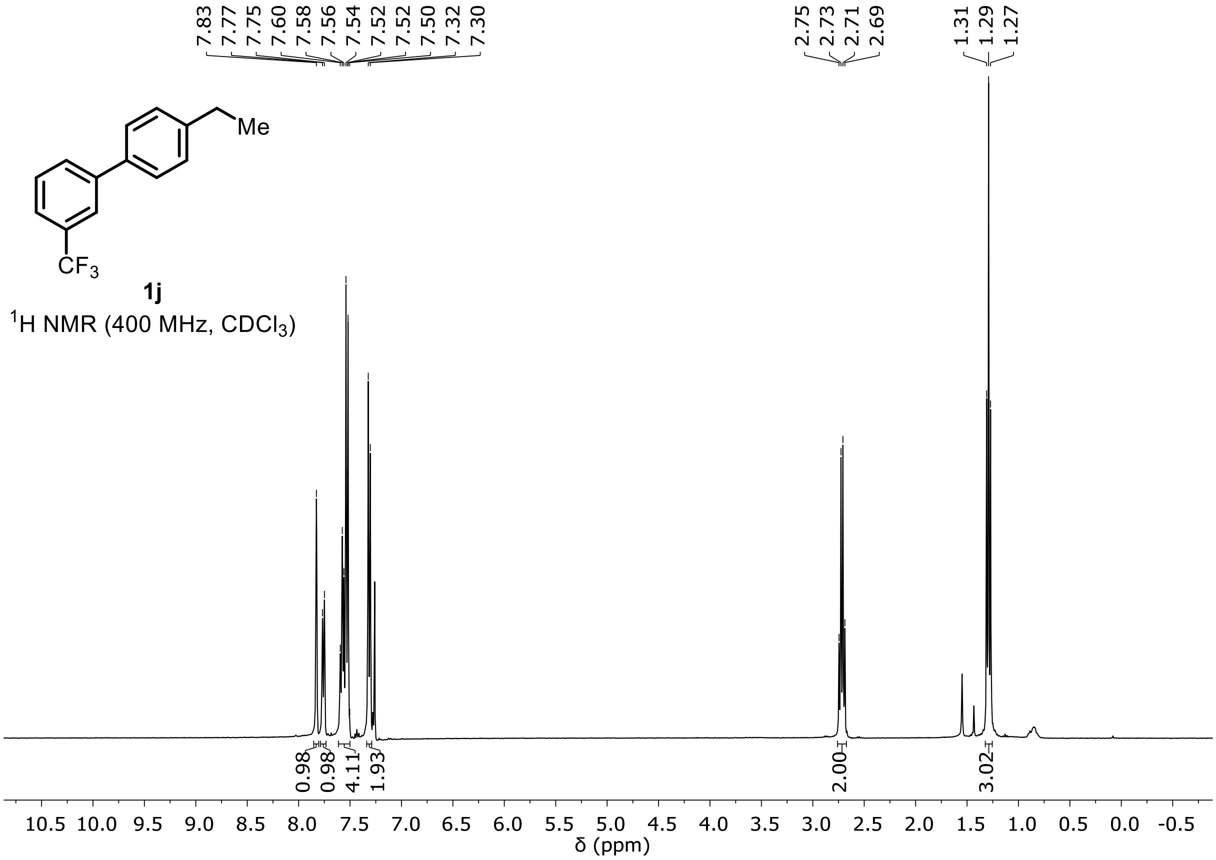

7.83
7.77
7.75
7.60
7.58
7.56
7.54
7.52
7.52
7.50
7.32
7.30
2.75
2.73
2.71
2.69
1.31
1.29
1.27
Me
CF3
1j
1H NMR (400 MHz, CDCl3)
0.98
0.98
4.11
1.93
2.00
3.02
10.5 10.0 9.5 9.0 8.5 8.0 7.5 7.0 6.5 6.0 5.5 5.0 4.5 4.0 3.5 3.0 2.5 2.0 1.5 1.0 0.5 0.0 -0.5
δ (ppm)

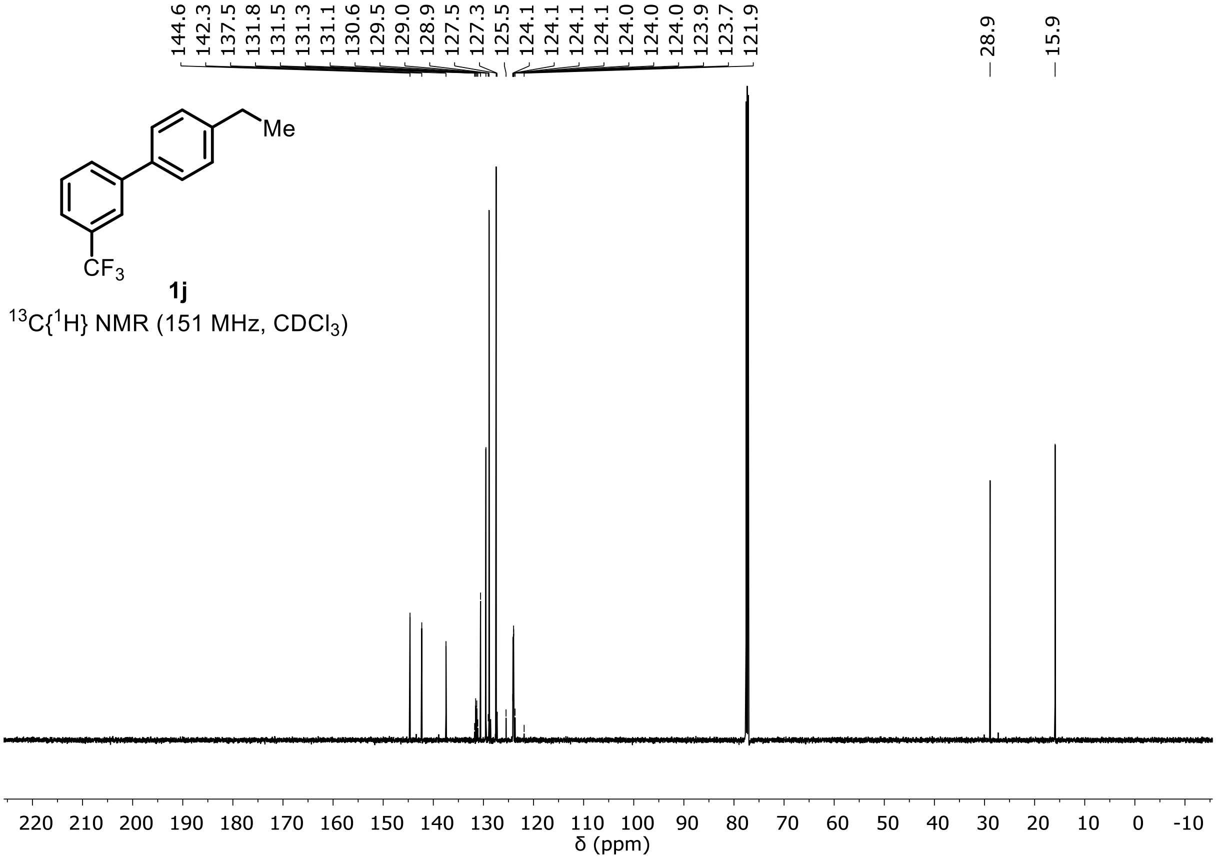

144.6
142.3
137.5
131.8
131.5
131.3
131.1
130.6
129.5
129.0
128.9
127.5
127.3
125.5
124.1
124.1
124.1
124.1
124.0
124.0
124.0
123.9
123.7
121.9
28.9
15.9
Me
CF3
1j
13C{1H} NMR (151 MHz, CDCl3)
220 210 200 190 180 170 160 150 140 130 120 110 100 90 80 70 60 50 40 30 20 10 0 -10
δ (ppm)

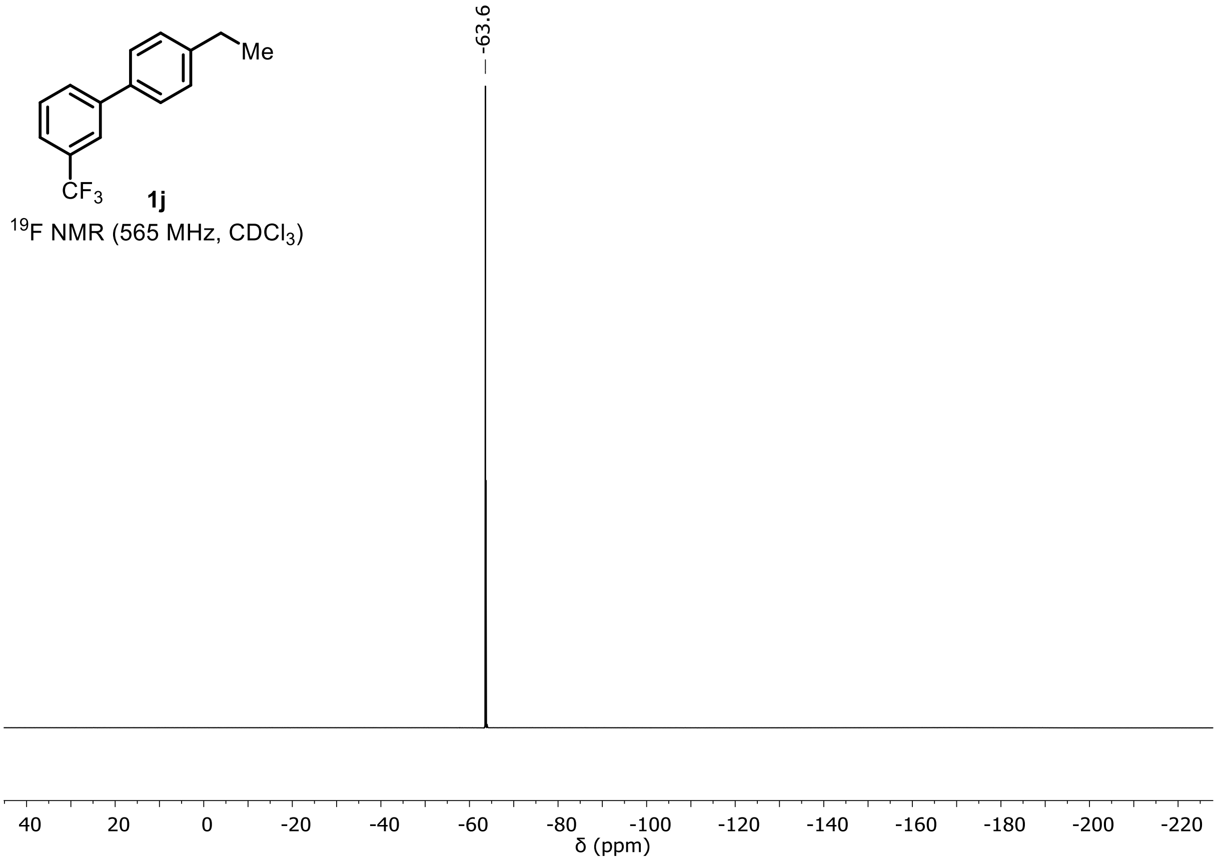

Me
CF3
1j
19F NMR (565 MHz, CDCl3)
-63.6
40
20
0
-20
-40
-60
-80
-100
-120
-140
-160
-180
-200
-220
δ (ppm)

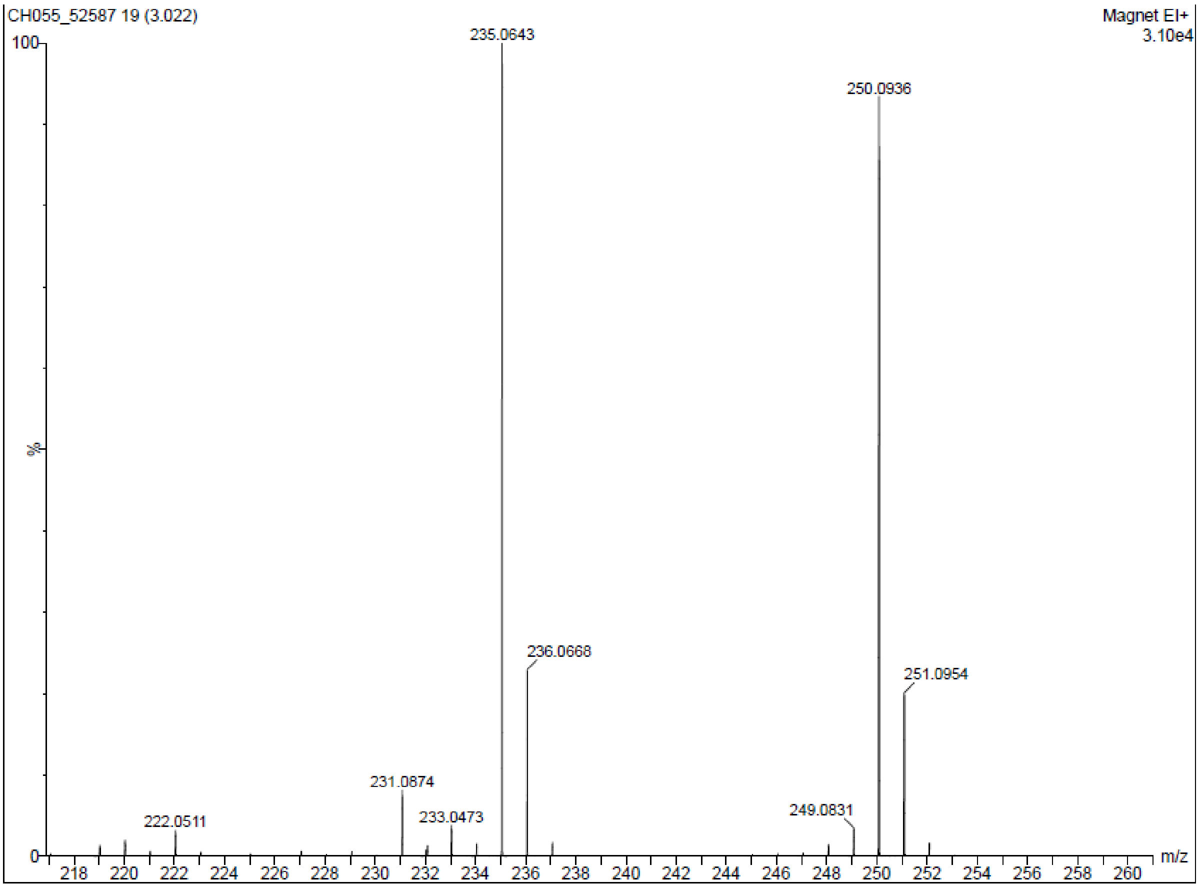

CH055_52587 19 (3.022)
Magnet EI+
3.10e4
100
%
0
235.0643
250.0936
236.0668
251.0954
231.0874
233.0473
222.0511
249.0831
m/z
218
220
222
224
226
228
230
232
234
236
238
240
242
244
246
248
250
252
254
256
258
260

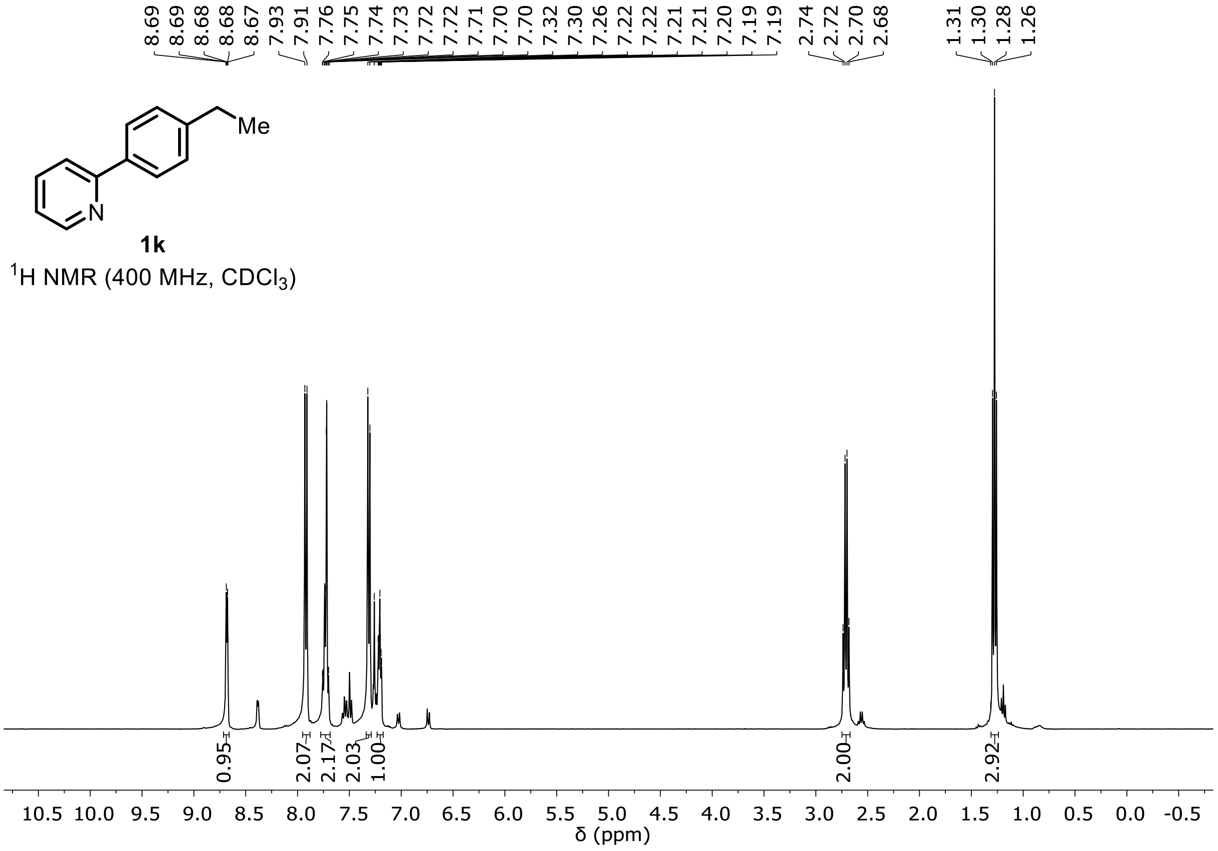
Me
N
1k
^{1}H NMR (400 MHz, CDCl$_3$)
8.69 8.69 8.68 8.68 8.67 7.93 7.91 7.76 7.75 7.74 7.73 7.72 7.72 7.71 7.70 7.70 7.32 7.30 7.26 7.22 7.22 7.21 7.21 7.20 7.19 7.19 2.74 2.72 2.70 2.68 1.31 1.30 1.28 1.26
0.95 2.07 2.17 2.03 1.00 2.00 2.92
δ (ppm)

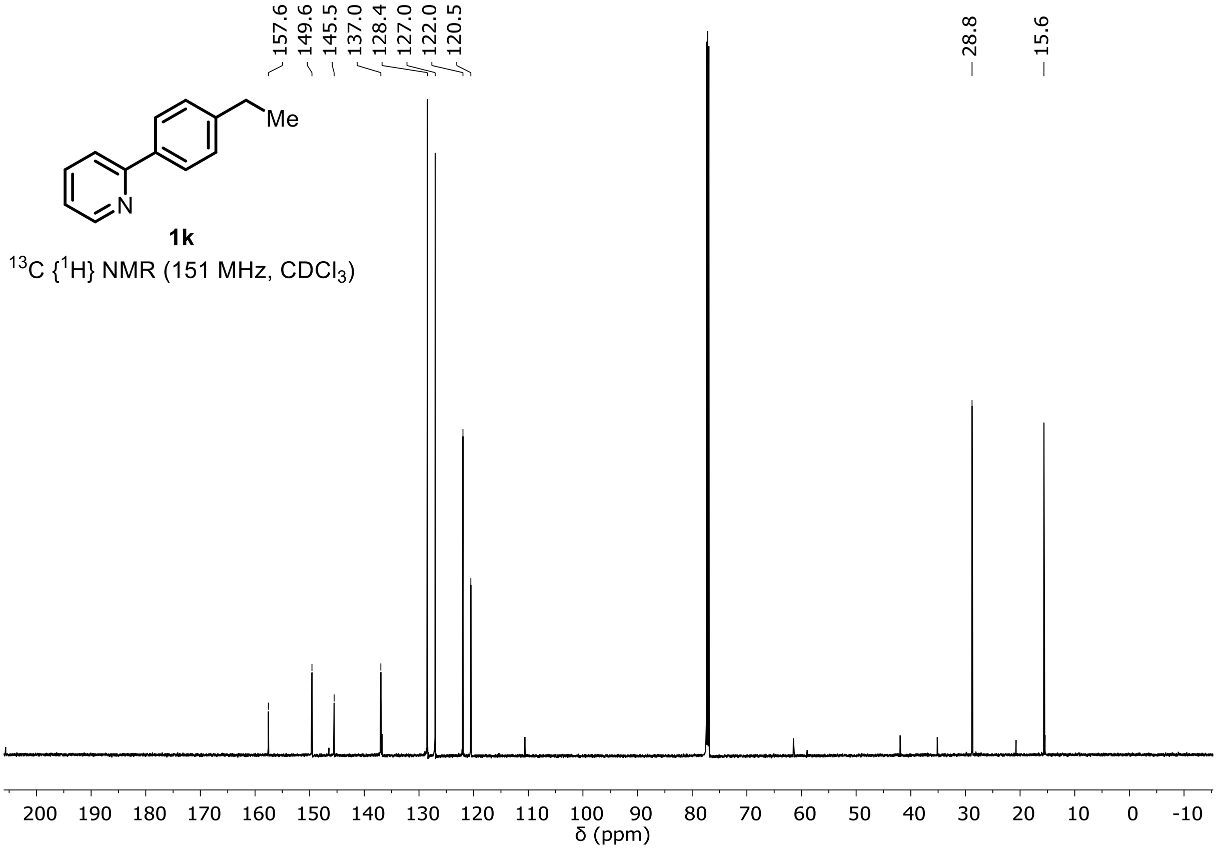
Me
N
1k
^{13}C {^{1}H} NMR (151 MHz, CDCl$_3$)
157.6 149.6 145.5 137.0 128.4 127.0 122.0 120.5 28.8 15.6
δ (ppm)

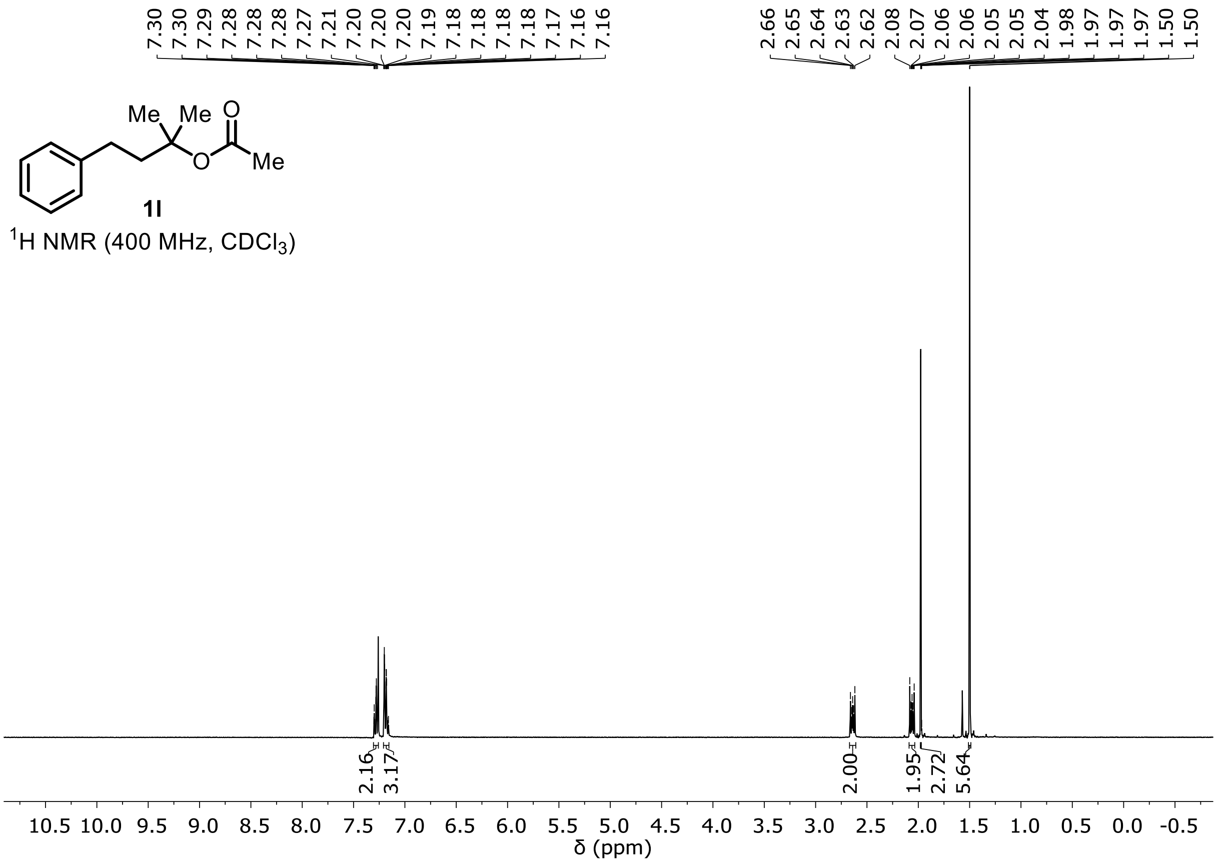
7.30 7.30 7.29 7.28 7.28 7.28 7.27 7.21 7.20 7.20 7.20 7.19 7.18 7.18 7.18 7.18 7.17 7.16 7.16
2.66 2.65 2.64 2.63 2.62 2.08 2.07 2.06 2.06 2.05 2.05 2.04 1.98 1.97 1.97 1.97 1.50 1.50
Me Me O
O
Me
1l
1H NMR (400 MHz, CDCl3)
2.16
3.17
2.00
1.95
2.72
5.64
10.5 10.0 9.5 9.0 8.5 8.0 7.5 7.0 6.5 6.0 5.5 5.0 4.5 4.0 3.5 3.0 2.5 2.0 1.5 1.0 0.5 0.0 -0.5
δ (ppm)

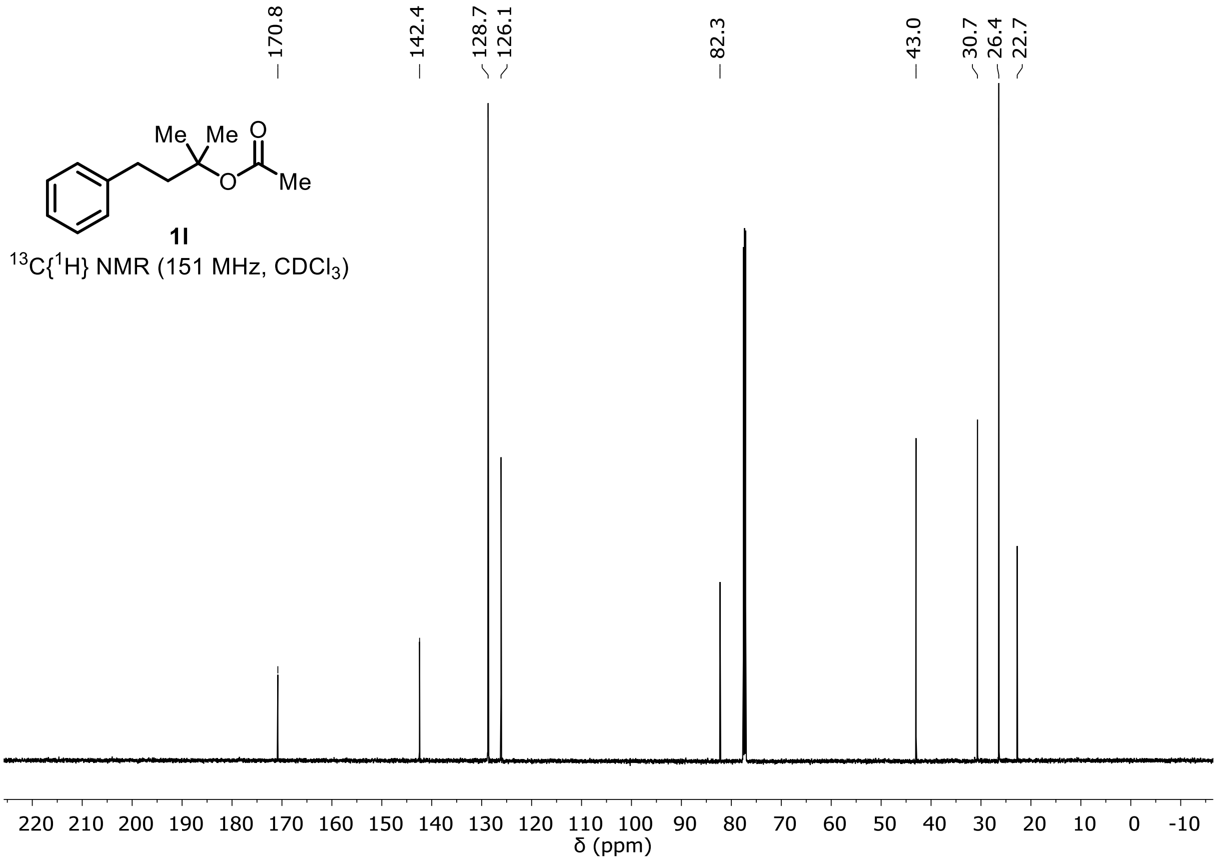
170.8
142.4
128.7
126.1
82.3
43.0
30.7
26.4
22.7
Me Me O
O
Me
1l
13C{1H} NMR (151 MHz, CDCl3)
220 210 200 190 180 170 160 150 140 130 120 110 100 90 80 70 60 50 40 30 20 10 0 -10
δ (ppm)

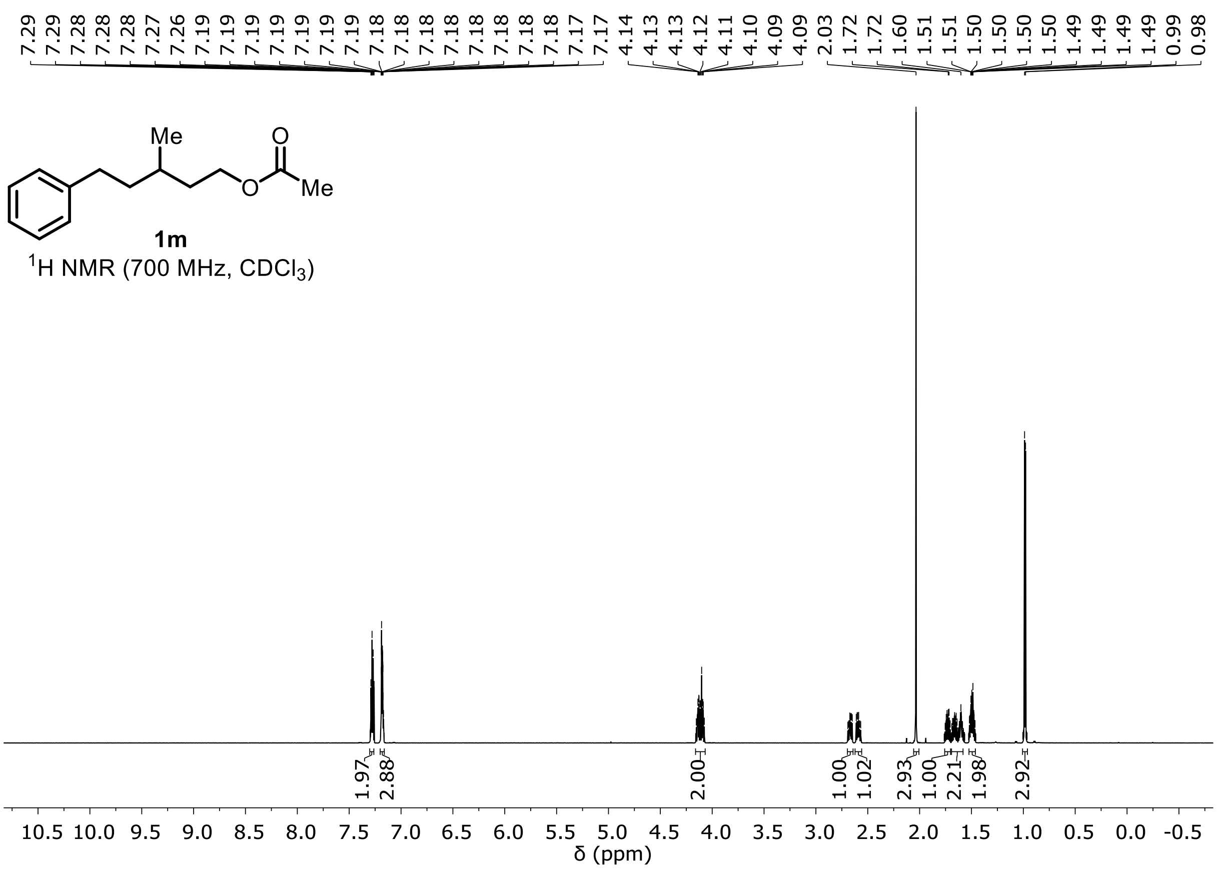
Me
O
O
Me
1m
1H NMR (700 MHz, CDCl3)
δ (ppm)

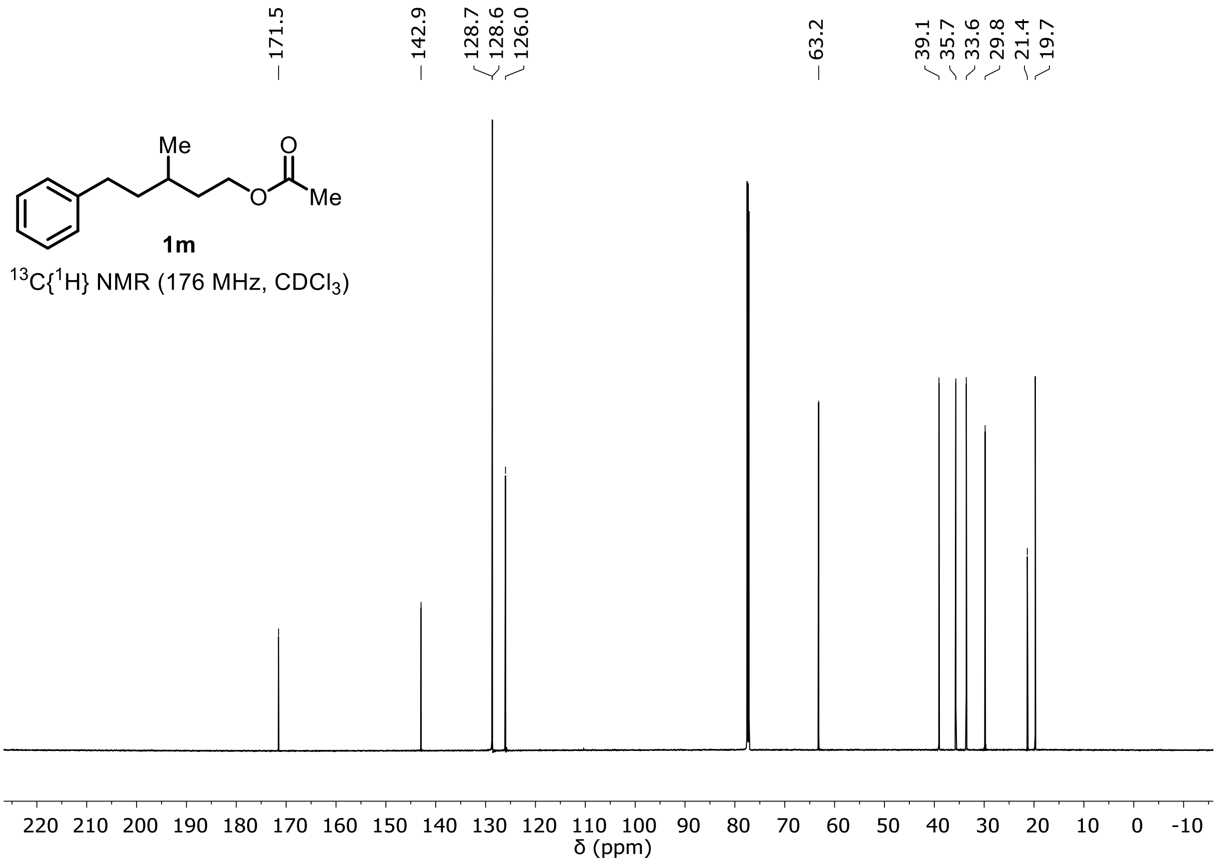
Me
O
O
Me
1m
13C{1H} NMR (176 MHz, CDCl3)
δ (ppm)

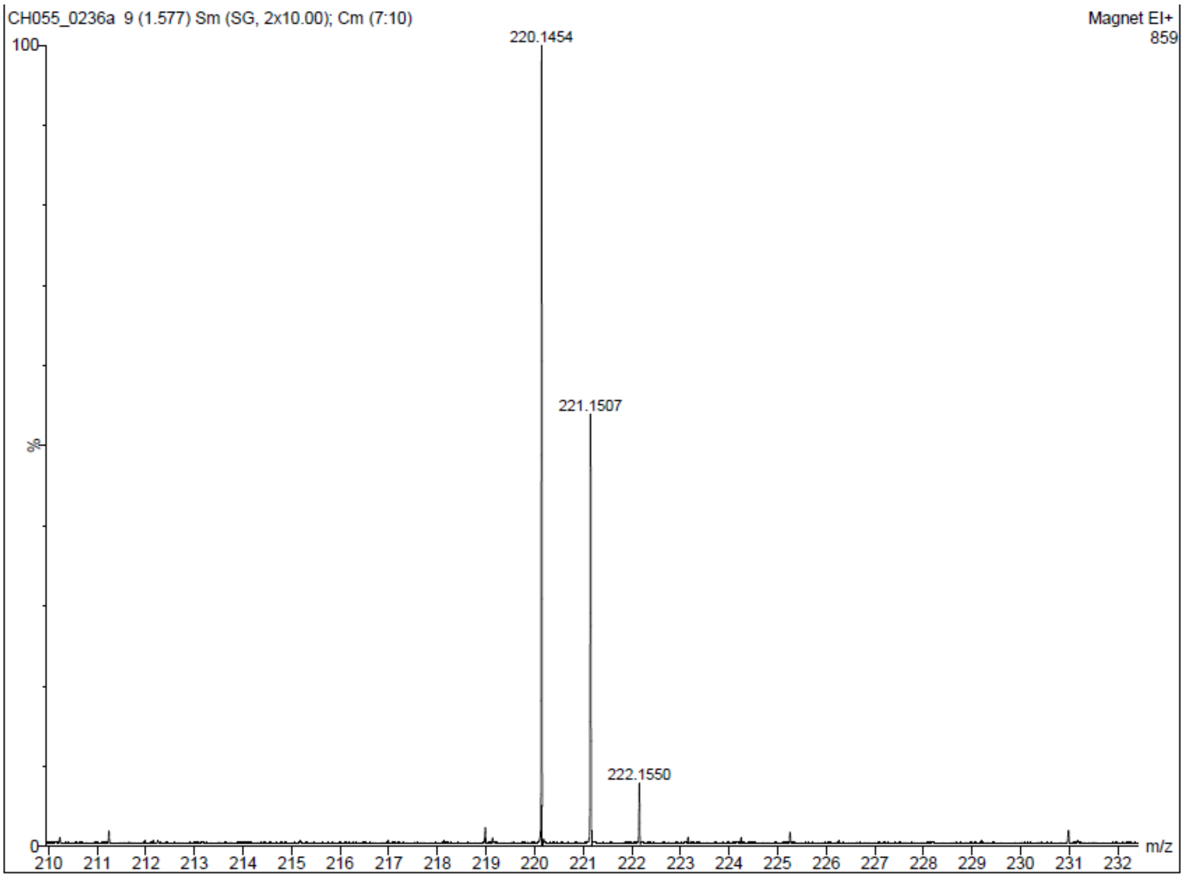
CH055_0236a 9 (1.577) Sm (SG, 2x10.00); Cm (7:10)
Magnet EI+
859
100
%
0
220.1454
221.1507
222.1550
m/z
210
211
212
213
214
215
216
217
218
219
220
221
222
223
224
225
226
227
228
229
230
231
232

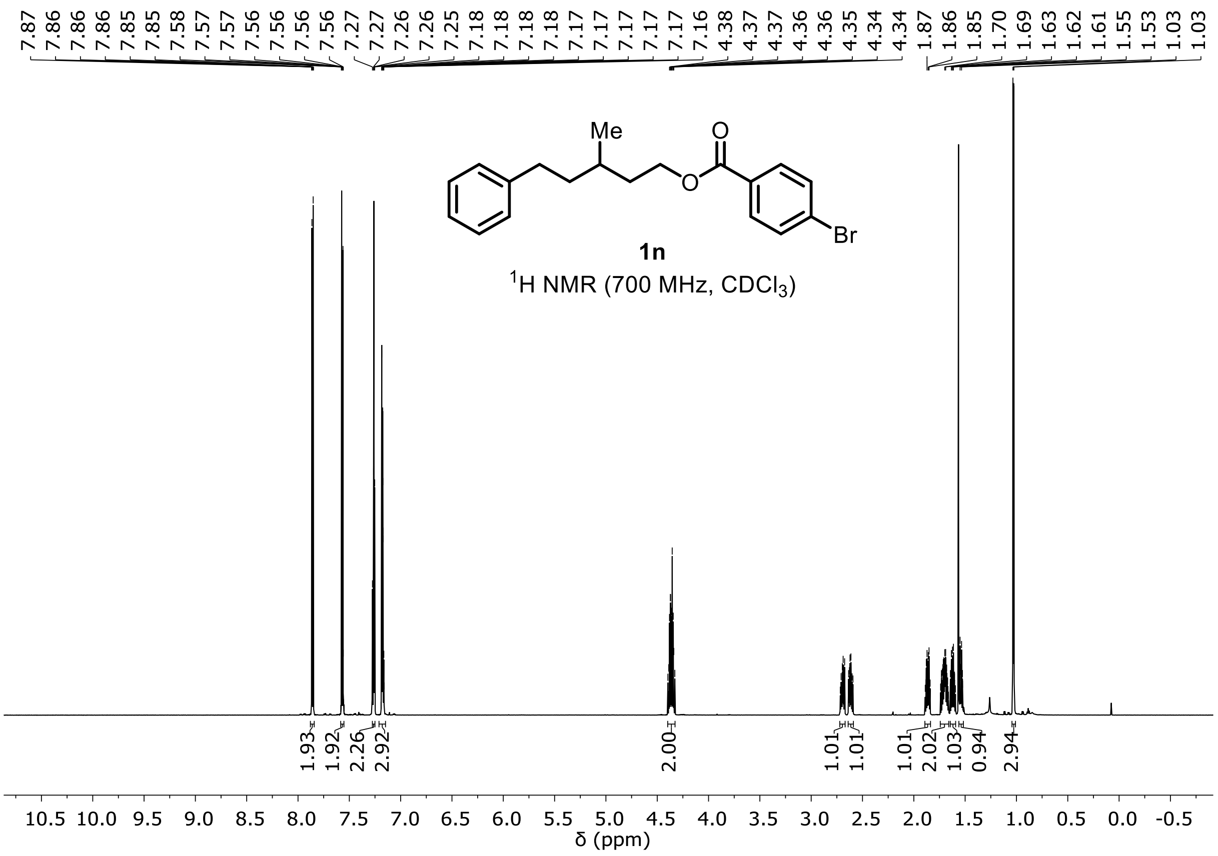
7.87 7.86 7.86 7.86 7.85 7.85 7.58 7.57 7.57 7.56 7.56 7.56 7.56 7.27 7.27 7.26 7.26 7.25 7.18 7.18 7.18 7.18 7.17 7.17 7.17 7.17 7.17 7.16 4.38 4.37 4.37 4.36 4.36 4.35 4.34 4.34 1.87 1.86 1.85 1.70 1.69 1.63 1.62 1.61 1.55 1.53 1.03 1.03
Me
O
O
Br
1n
1H NMR (700 MHz, CDCl3)
1.93 1.92 2.26 2.92 2.00 1.01 1.01 1.01 2.02 1.03 0.94 2.94
10.5 10.0 9.5 9.0 8.5 8.0 7.5 7.0 6.5 6.0 5.5 5.0 4.5 4.0 3.5 3.0 2.5 2.0 1.5 1.0 0.5 0.0 -0.5
δ (ppm)

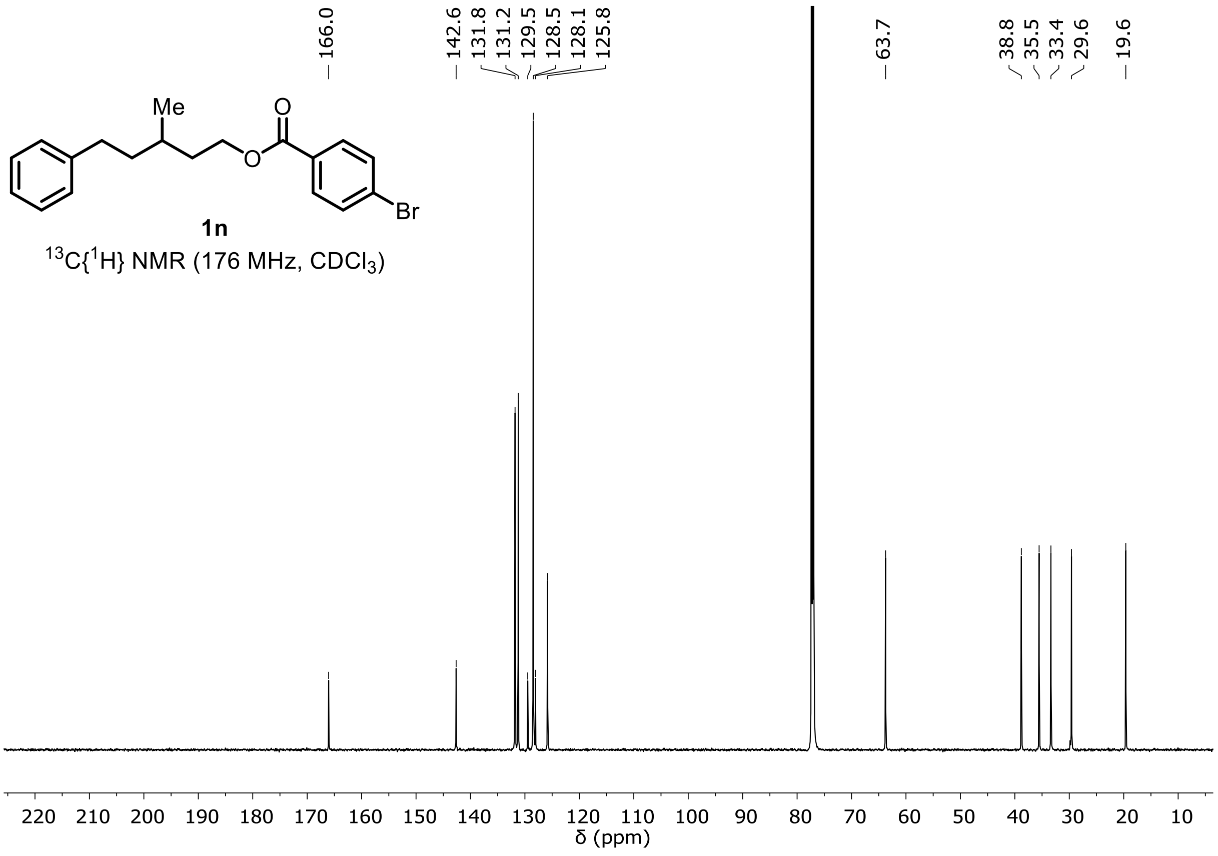
166.0 142.6 131.8 131.2 129.5 128.5 128.1 125.8 63.7 38.8 35.5 33.4 29.6 19.6
Me
O
O
Br
1n
13C{1H} NMR (176 MHz, CDCl3)
220 210 200 190 180 170 160 150 140 130 120 110 100 90 80 70 60 50 40 30 20 10
δ (ppm)

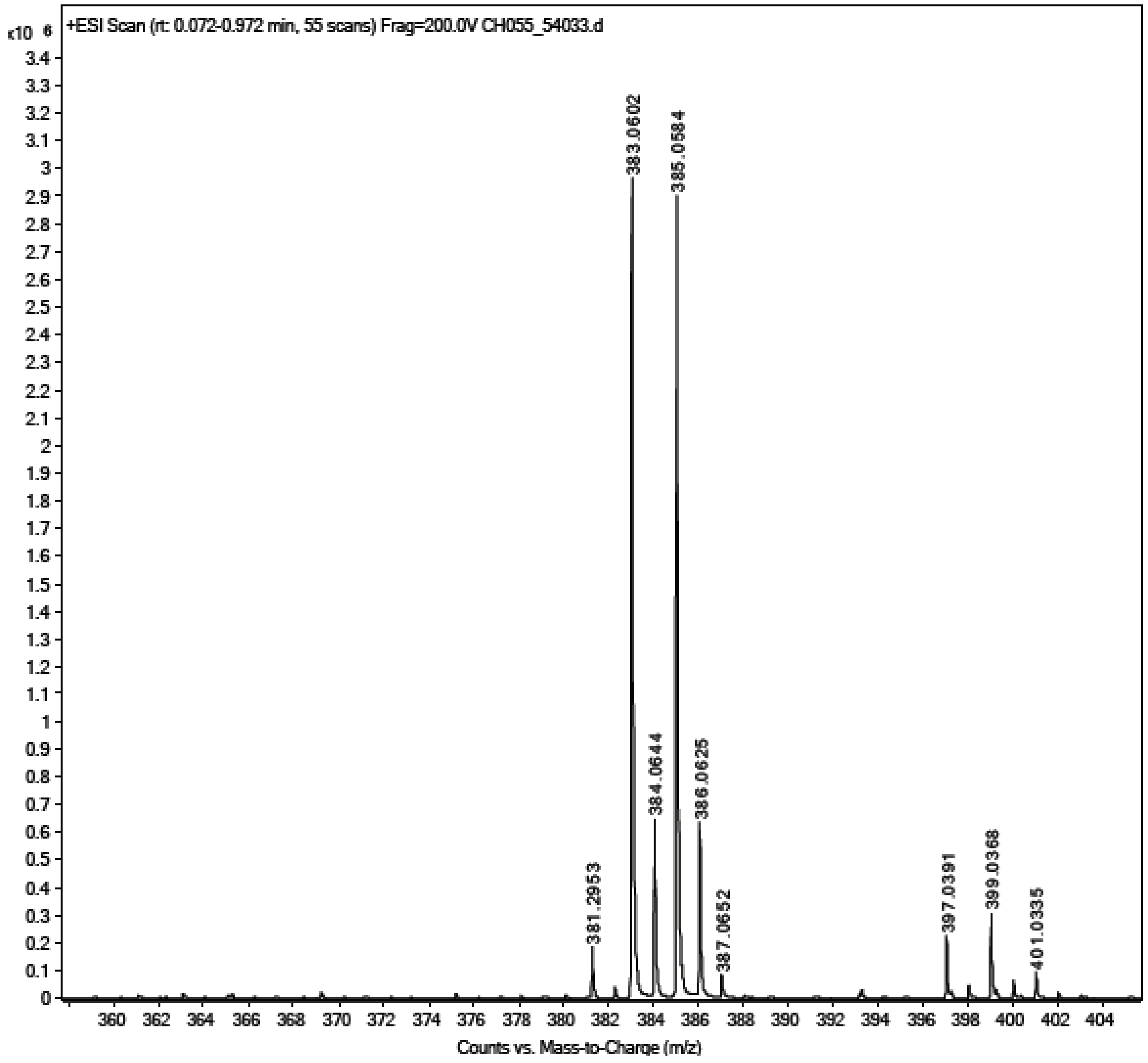

x10 6
+ESI Scan (rt: 0.072-0.972 min, 55 scans) Frag=200.0V CH055_54033.d
383.0602
385.0584
384.0644
386.0625
381.2953
387.0652
397.0391
399.0368
401.0335
Counts vs. Mass-to-Charge (m/z)

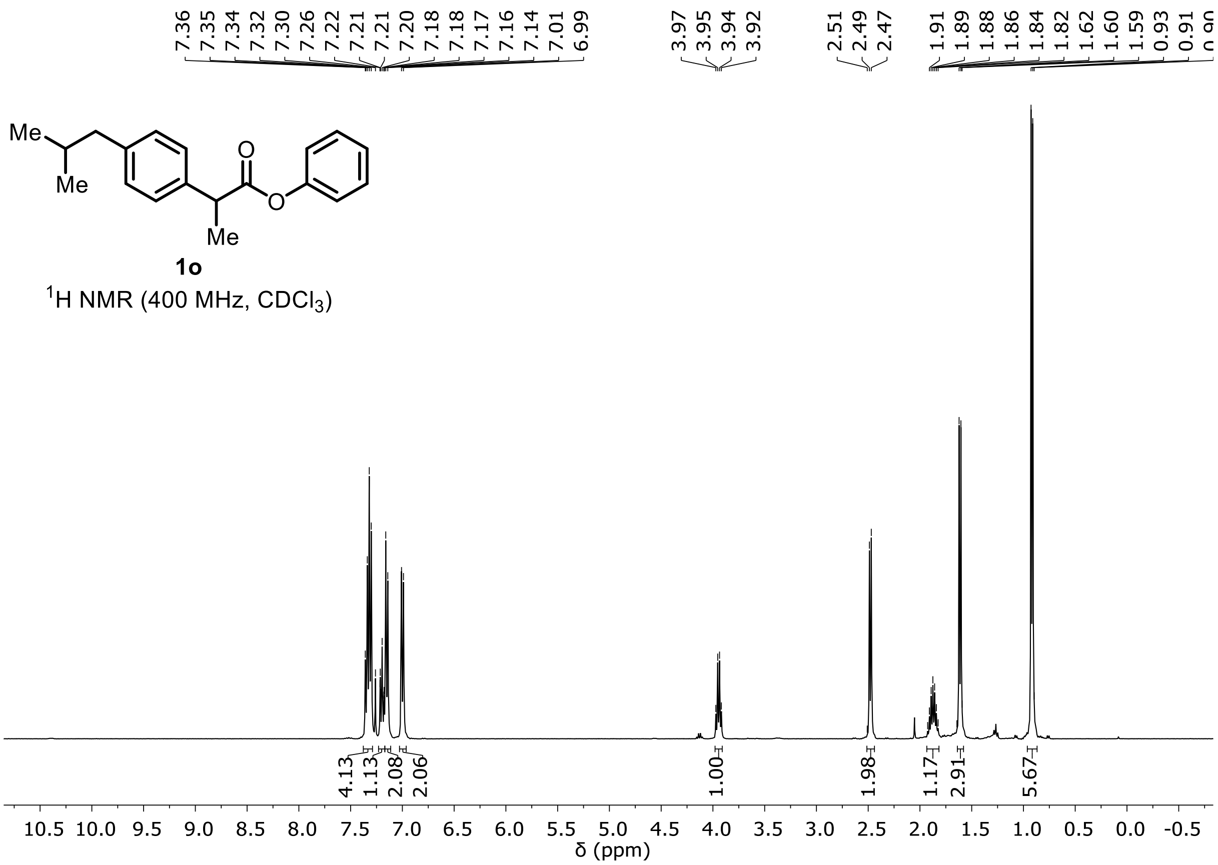
7.36 7.35 7.34 7.32 7.30 7.26 7.22 7.21 7.21 7.20 7.18 7.18 7.17 7.16 7.14 7.01 6.99
3.97 3.95 3.94 3.92
2.51 2.49 2.47
1.91 1.89 1.88 1.86 1.84 1.82 1.62 1.60 1.59 0.93 0.91
Me
Me
O
O
Me
1o
^{1}H NMR (400 MHz, CDCl$_3$)
4.13 1.13 2.08 2.06 1.00 1.98 1.17 2.91 5.67
10.5 10.0 9.5 9.0 8.5 8.0 7.5 7.0 6.5 6.0 5.5 5.0 4.5 4.0 3.5 3.0 2.5 2.0 1.5 1.0 0.5 0.0 -0.5
δ (ppm)

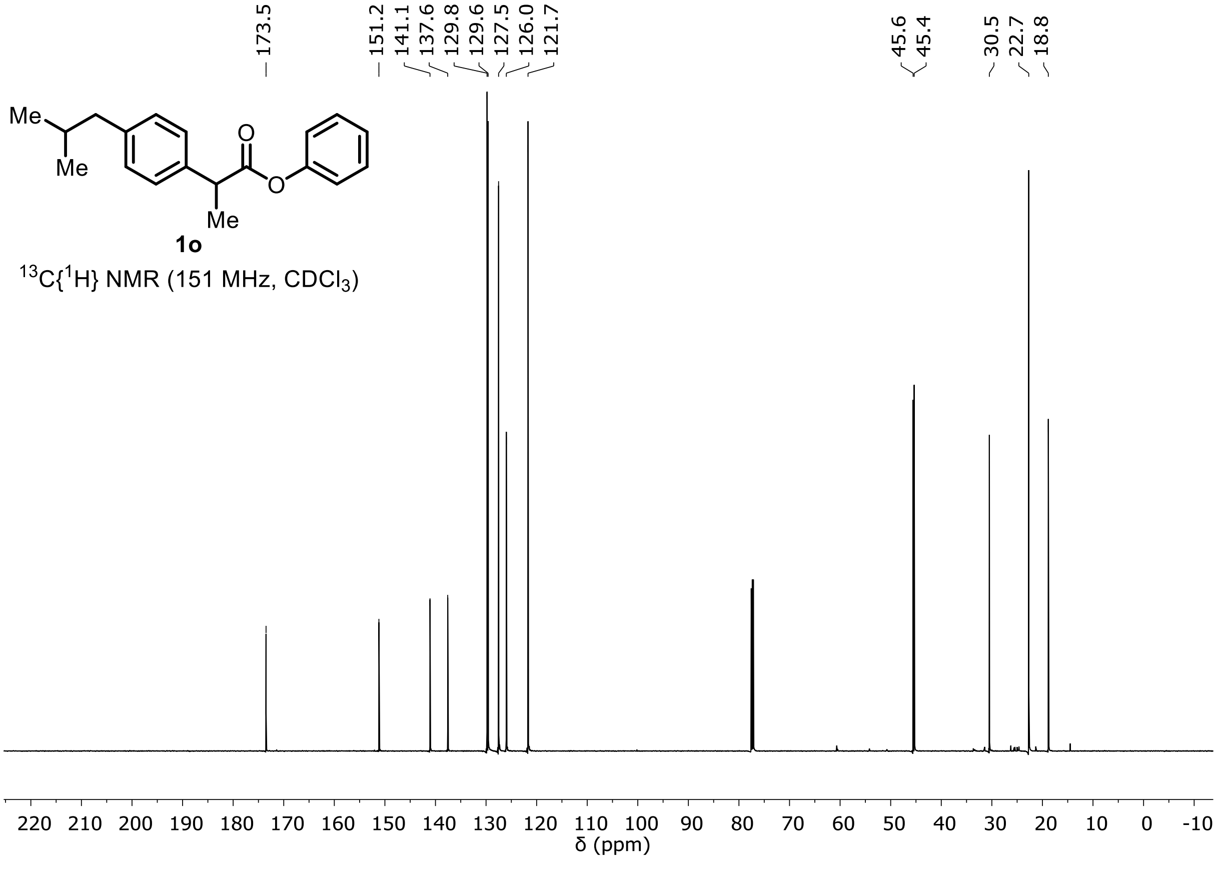
173.5
151.2 141.1 137.6 129.8 129.6 127.5 126.0 121.7
45.6 45.4
30.5 22.7 18.8
Me
Me
O
O
Me
1o
^{13}C{^{1}H} NMR (151 MHz, CDCl$_3$)
220 210 200 190 180 170 160 150 140 130 120 110 100 90 80 70 60 50 40 30 20 10 0 -10
δ (ppm)

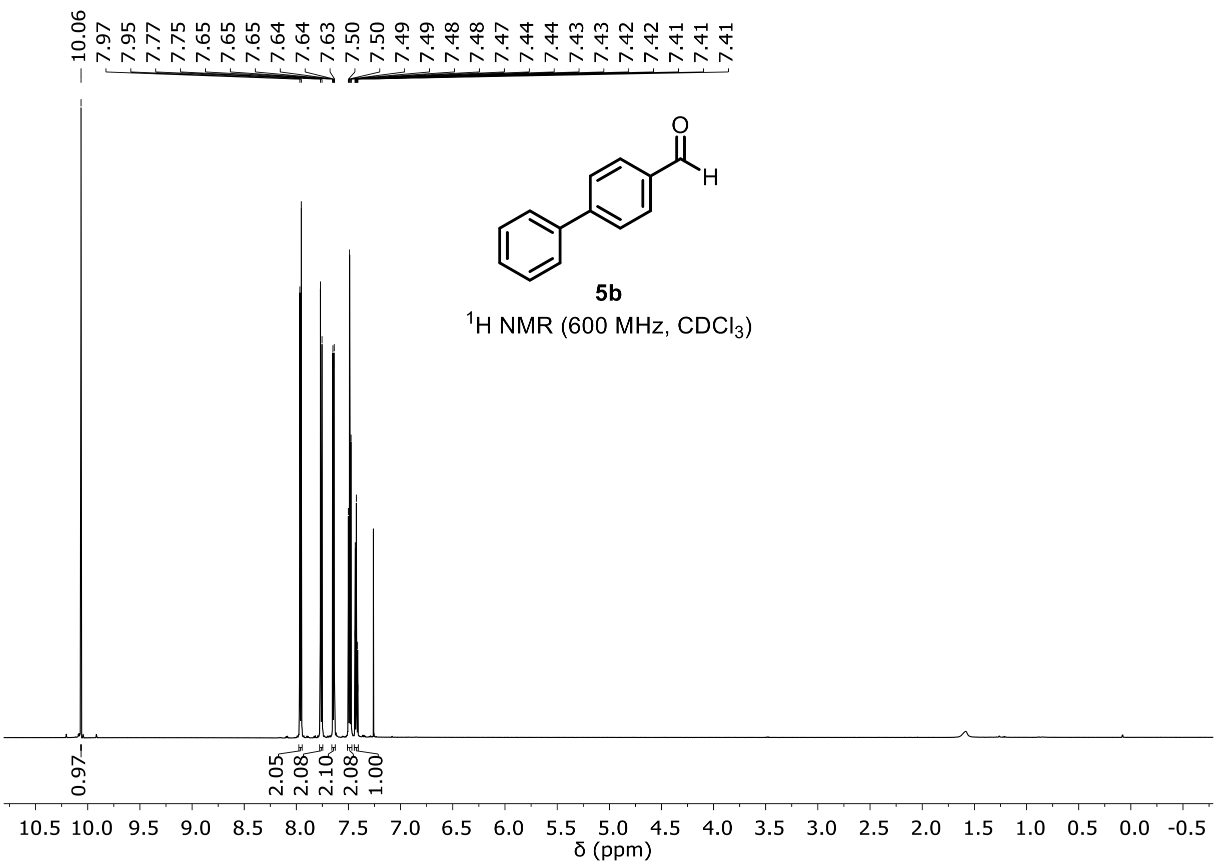
10.06
7.97
7.95
7.77
7.75
7.65
7.65
7.65
7.64
7.64
7.63
7.50
7.50
7.49
7.49
7.48
7.48
7.47
7.44
7.44
7.43
7.43
7.42
7.42
7.41
7.41
7.41
O
H
5b
1H NMR (600 MHz, CDCl3)
0.97
2.05
2.08
2.10
2.08
1.00
10.5 10.0 9.5 9.0 8.5 8.0 7.5 7.0 6.5 6.0 5.5 5.0 4.5 4.0 3.5 3.0 2.5 2.0 1.5 1.0 0.5 0.0 -0.5
δ (ppm)

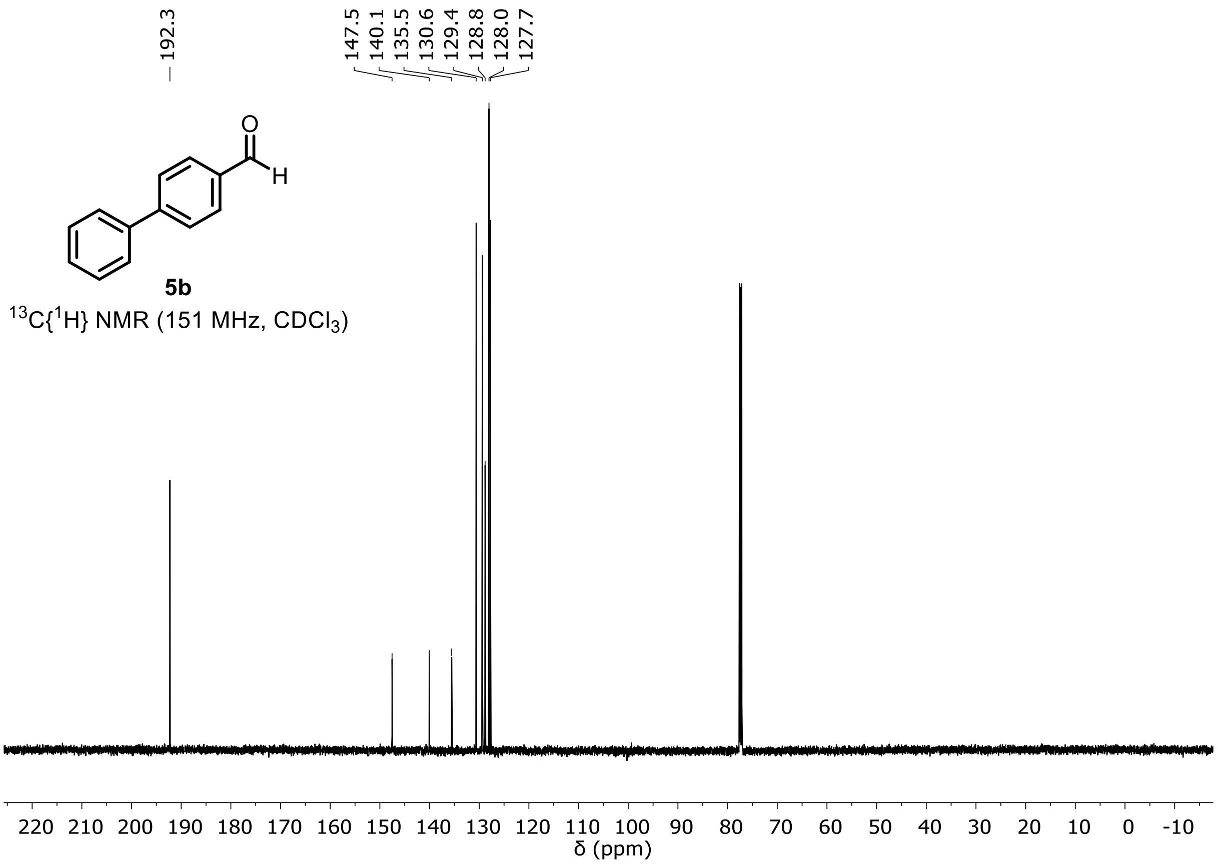
192.3
147.5
140.1
135.5
130.6
129.4
128.8
128.0
127.7
O
H
5b
13C{1H} NMR (151 MHz, CDCl3)
220 210 200 190 180 170 160 150 140 130 120 110 100 90 80 70 60 50 40 30 20 10 0 -10
δ (ppm)

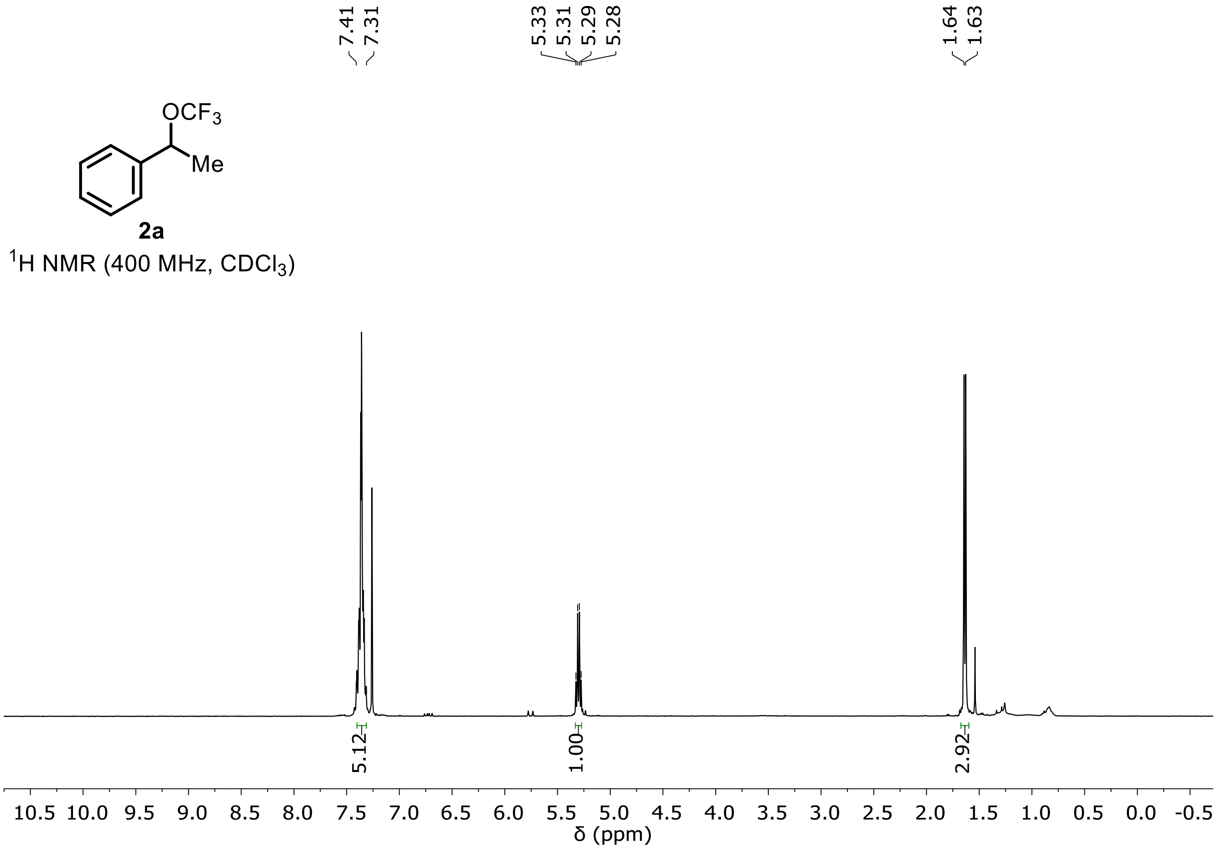
7.41
7.31
5.33
5.31
5.29
5.28
1.64
1.63
OCF3
Me
2a
1H NMR (400 MHz, CDCl3)
5.12
1.00
2.92
10.5 10.0 9.5 9.0 8.5 8.0 7.5 7.0 6.5 6.0 5.5 5.0 4.5 4.0 3.5 3.0 2.5 2.0 1.5 1.0 0.5 0.0 -0.5
δ (ppm)

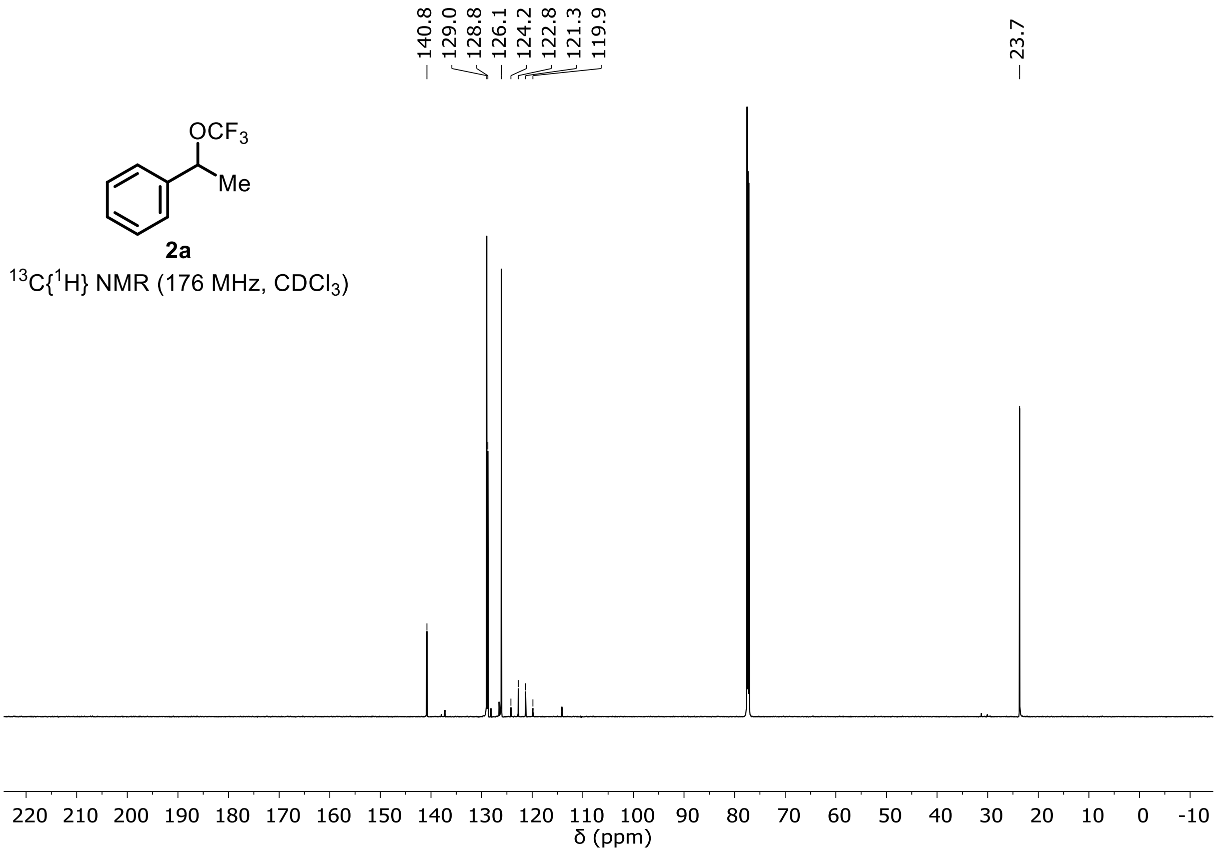
140.8
129.0
128.8
126.1
124.2
122.8
121.3
119.9
23.7
OCF3
Me
2a
13C{1H} NMR (176 MHz, CDCl3)
220 210 200 190 180 170 160 150 140 130 120 110 100 90 80 70 60 50 40 30 20 10 0 -10
δ (ppm)

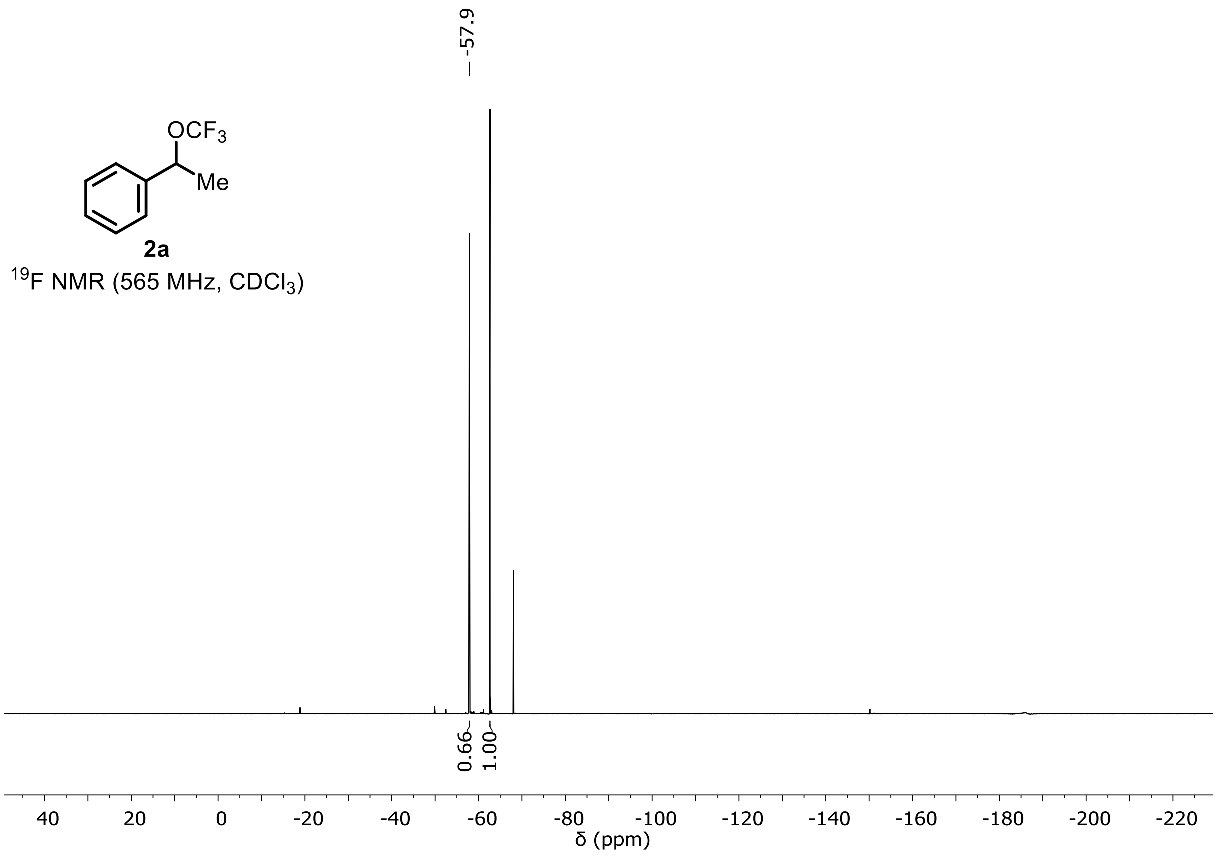

2a

^{19}F NMR (565 MHz, $CDCl_3$)

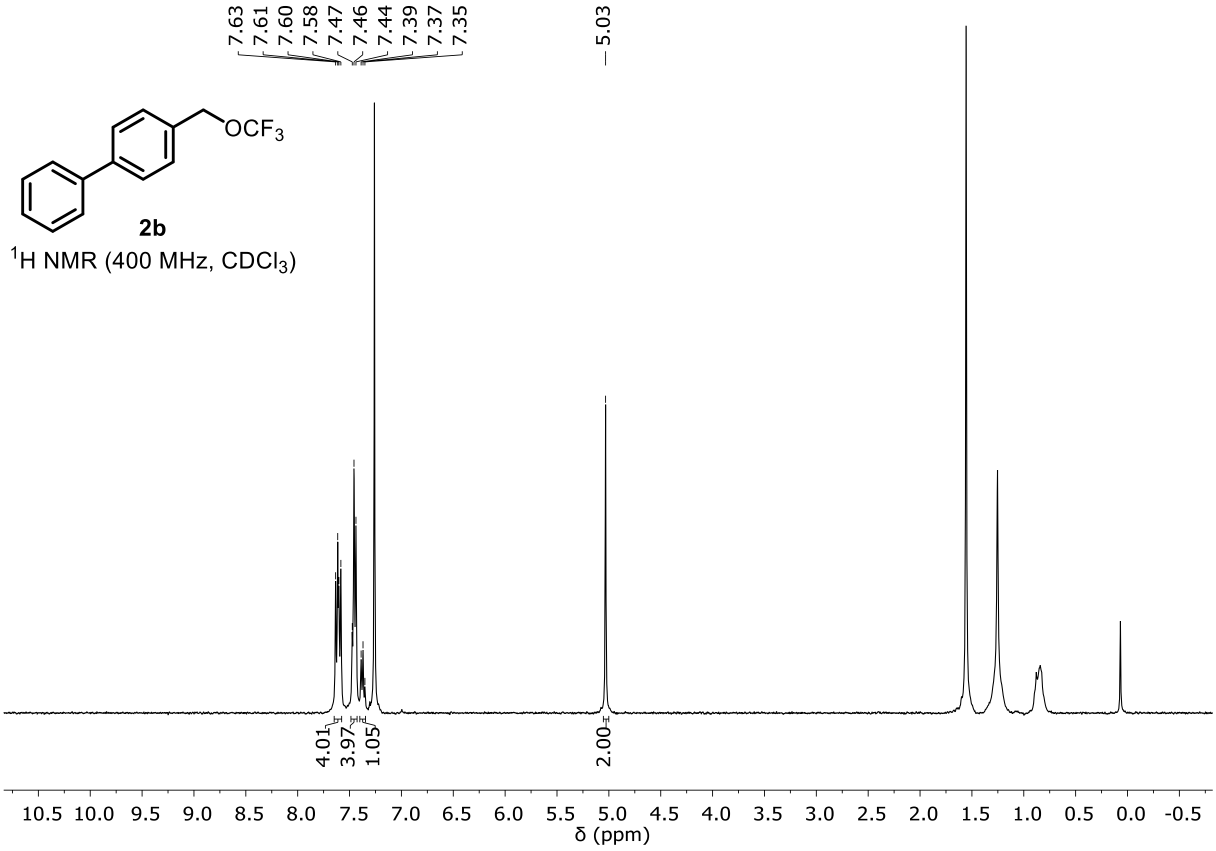
7.63 7.61 7.60 7.58 7.47 7.46 7.44 7.39 7.37 7.35
5.03
OCF3
2b
1H NMR (400 MHz, CDCl3)
4.01 3.97 1.05
2.00
10.5 10.0 9.5 9.0 8.5 8.0 7.5 7.0 6.5 6.0 5.5 5.0 4.5 4.0 3.5 3.0 2.5 2.0 1.5 1.0 0.5 0.0 -0.5
δ (ppm)

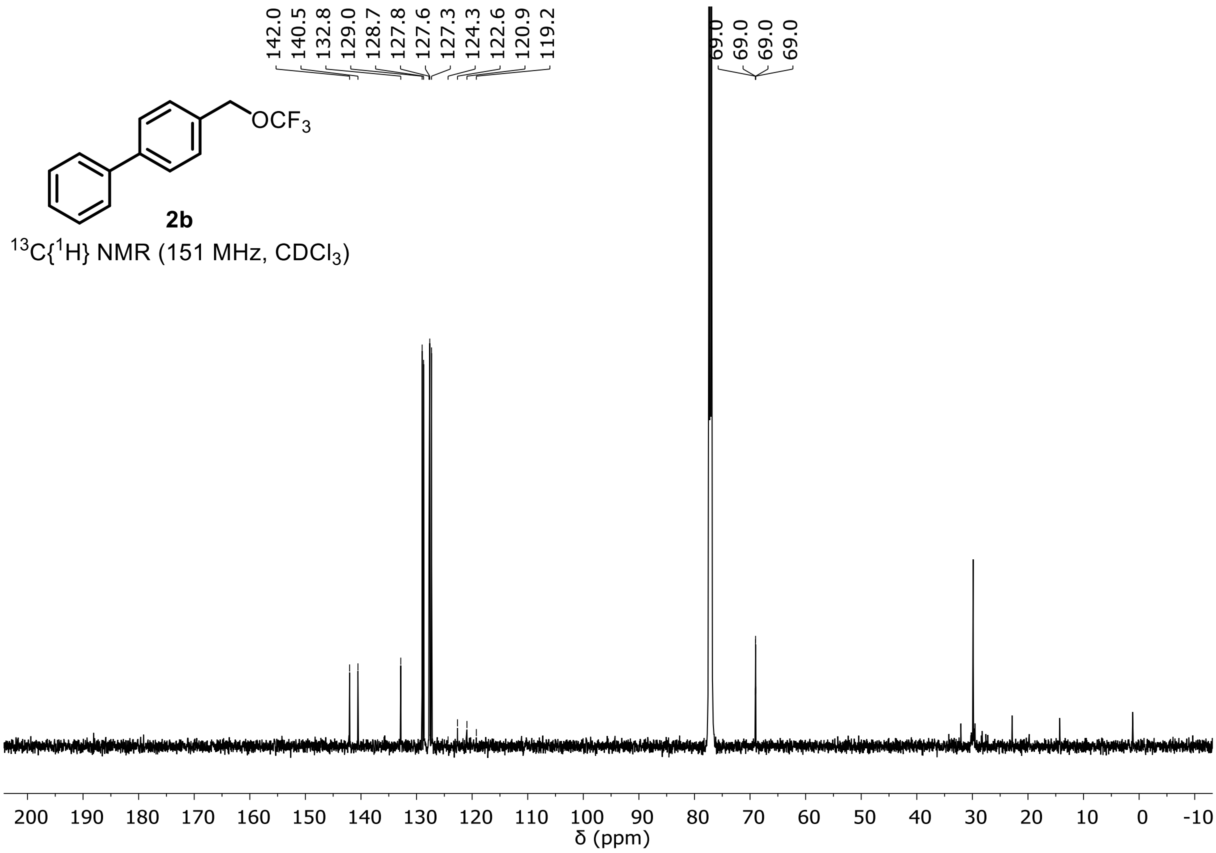
142.0 140.5 132.8 129.0 128.7 127.8 127.6 127.3 124.3 122.6 120.9 119.2
69.0 69.0 69.0 69.0
OCF3
2b
13C{1H} NMR (151 MHz, CDCl3)
200 190 180 170 160 150 140 130 120 110 100 90 80 70 60 50 40 30 20 10 0 -10
δ (ppm)

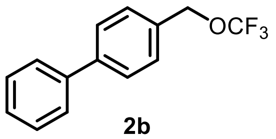

2b

^{19}F NMR (376 MHz, $CDCl_3$)

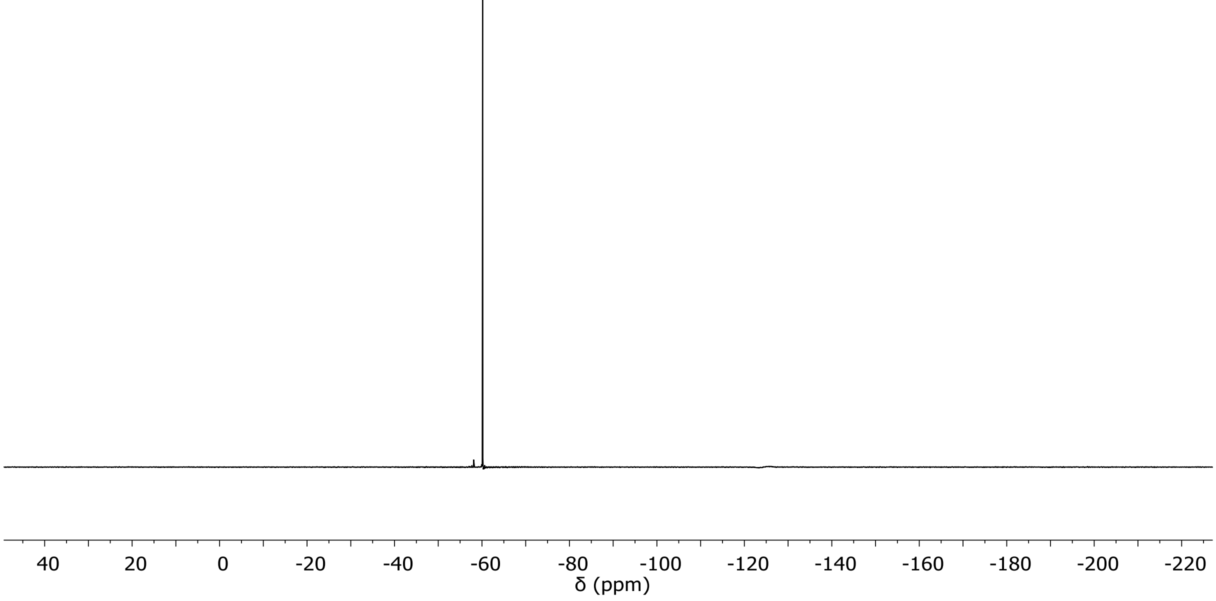

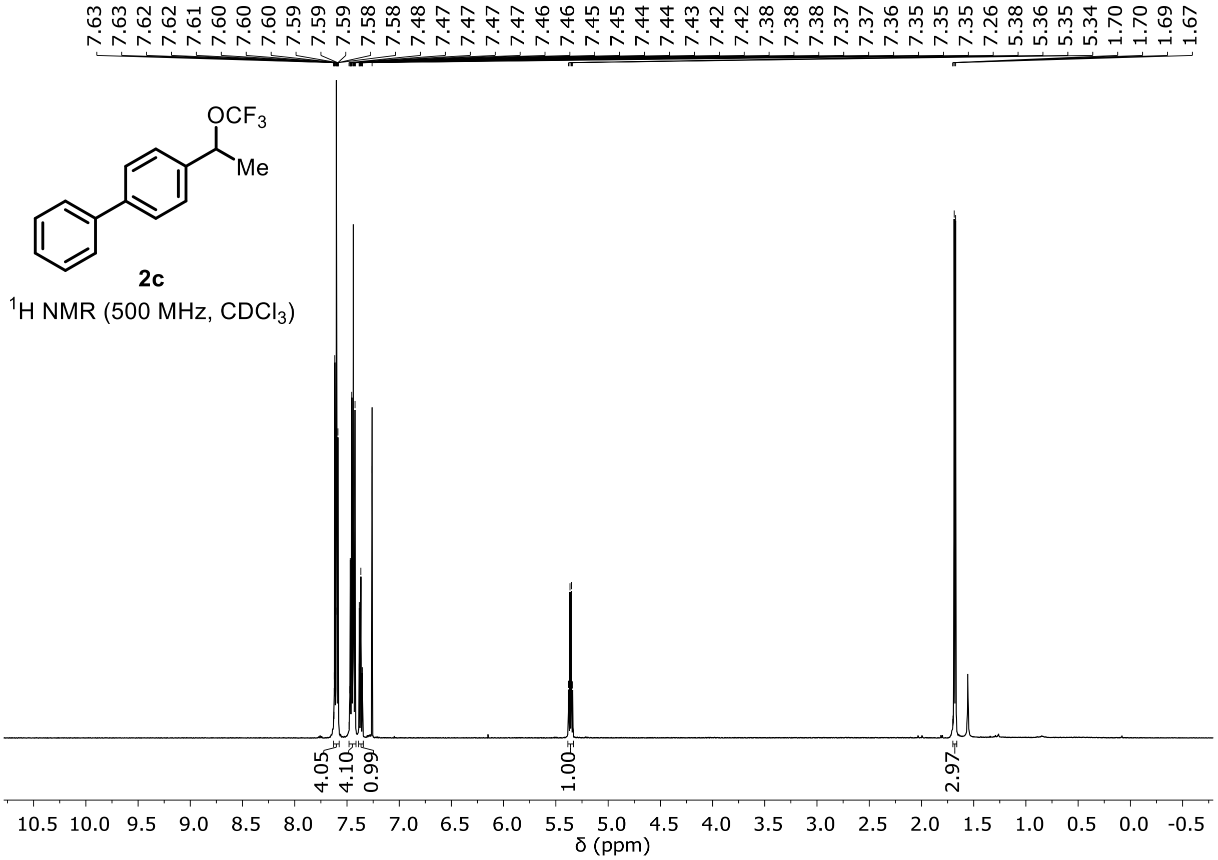

2c

^{1}H NMR (500 MHz, $CDCl_3$)

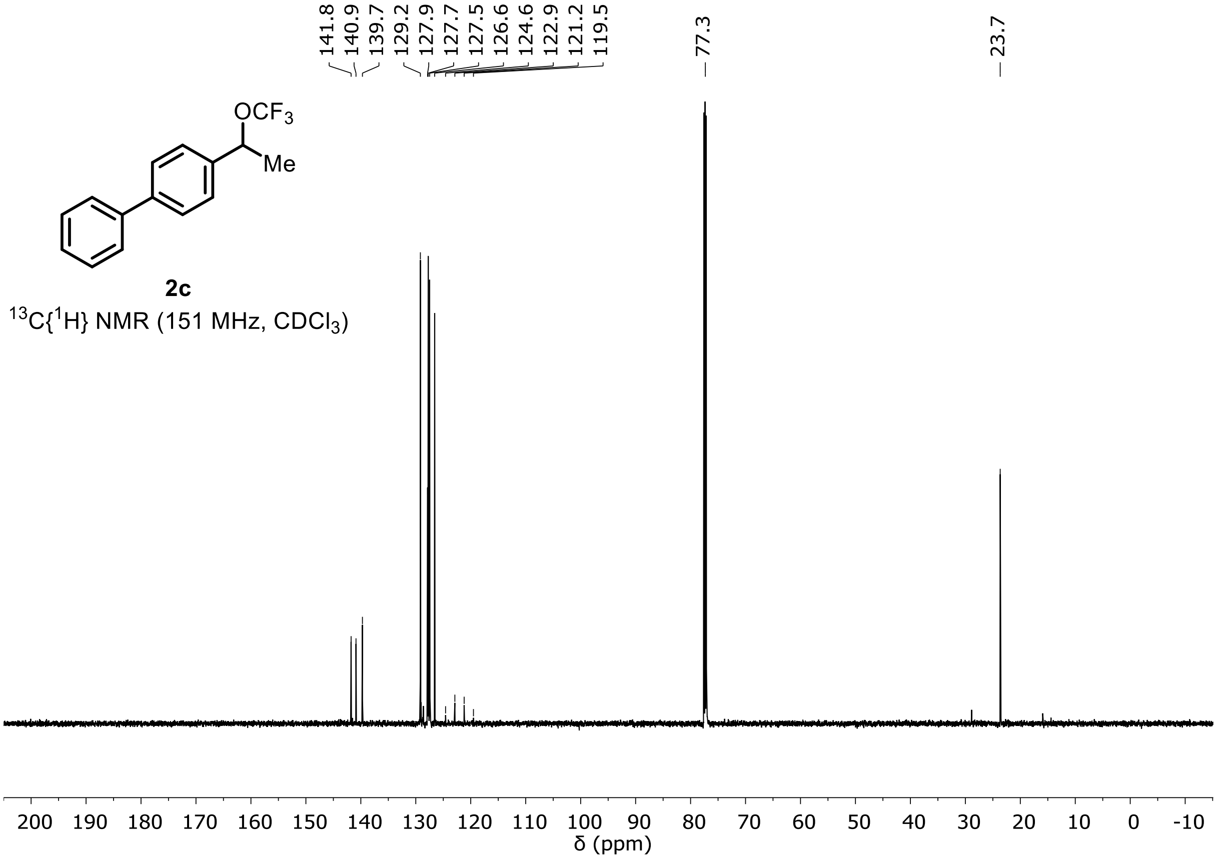

2c

$^{13}C\{^{1}H\}$ NMR (151 MHz, $CDCl_3$)

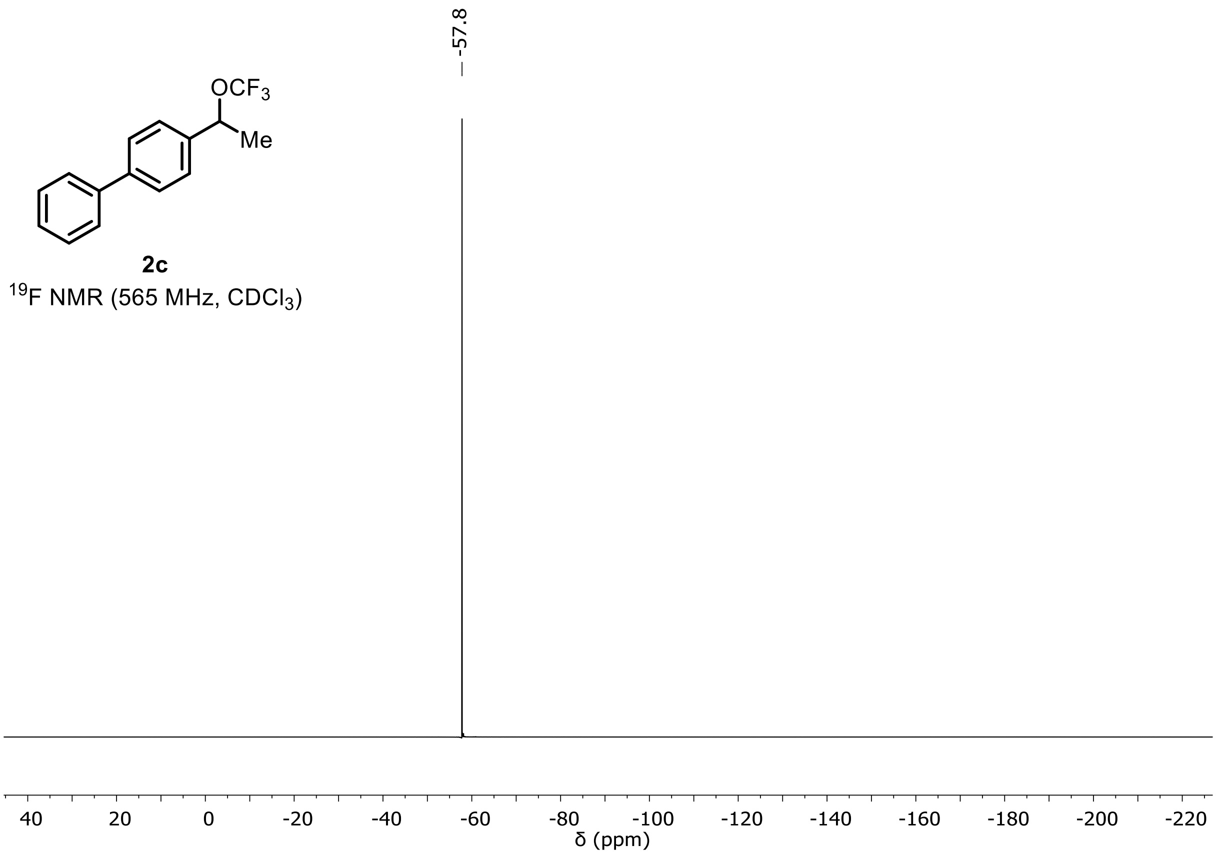

2c

^{19}F NMR (565 MHz, $CDCl_3$)

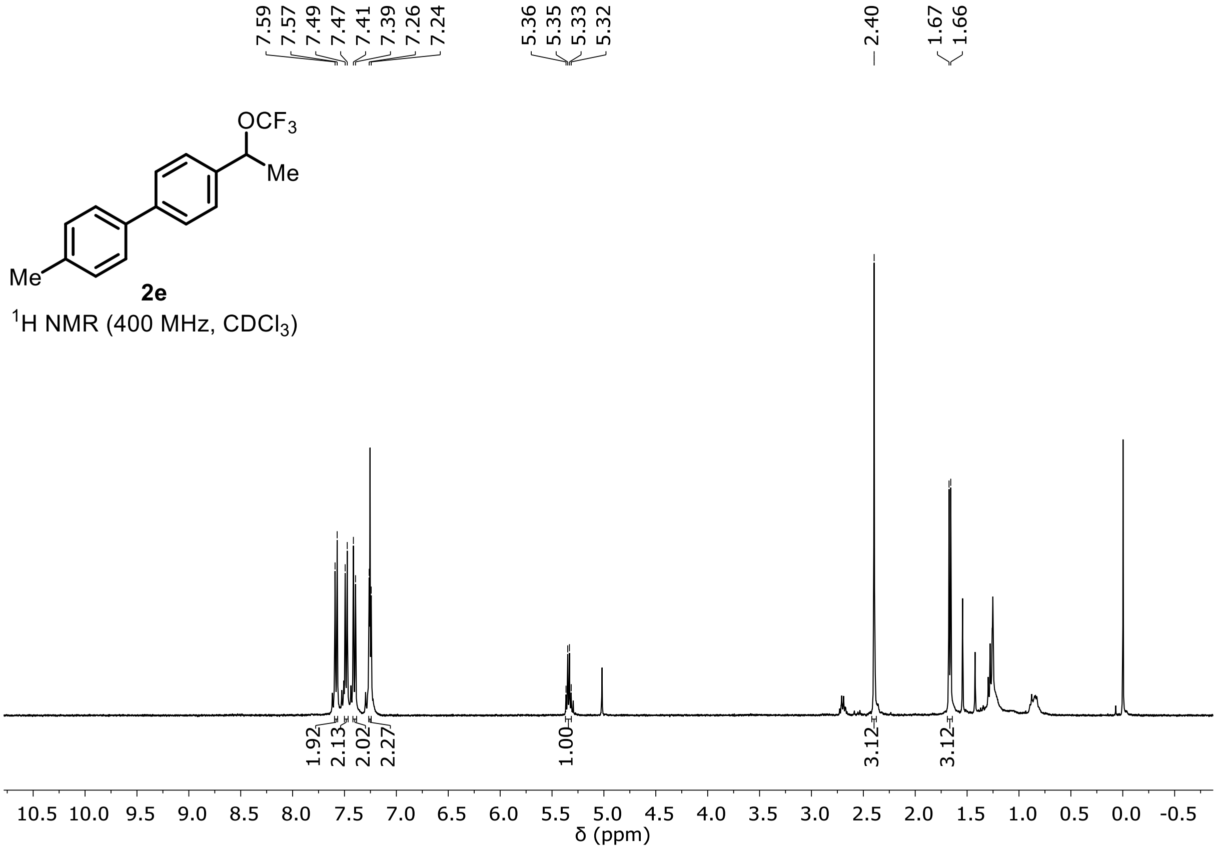
7.59 7.57 7.49 7.47 7.41 7.39 7.26 7.24
5.36 5.35 5.33 5.32
2.40
1.67 1.66
OCF3
Me
Me
2e
1H NMR (400 MHz, CDCl3)
1.92 2.13 2.02 2.27
1.00
3.12
3.12
10.5 10.0 9.5 9.0 8.5 8.0 7.5 7.0 6.5 6.0 5.5 5.0 4.5 4.0 3.5 3.0 2.5 2.0 1.5 1.0 0.5 0.0 -0.5
δ (ppm)

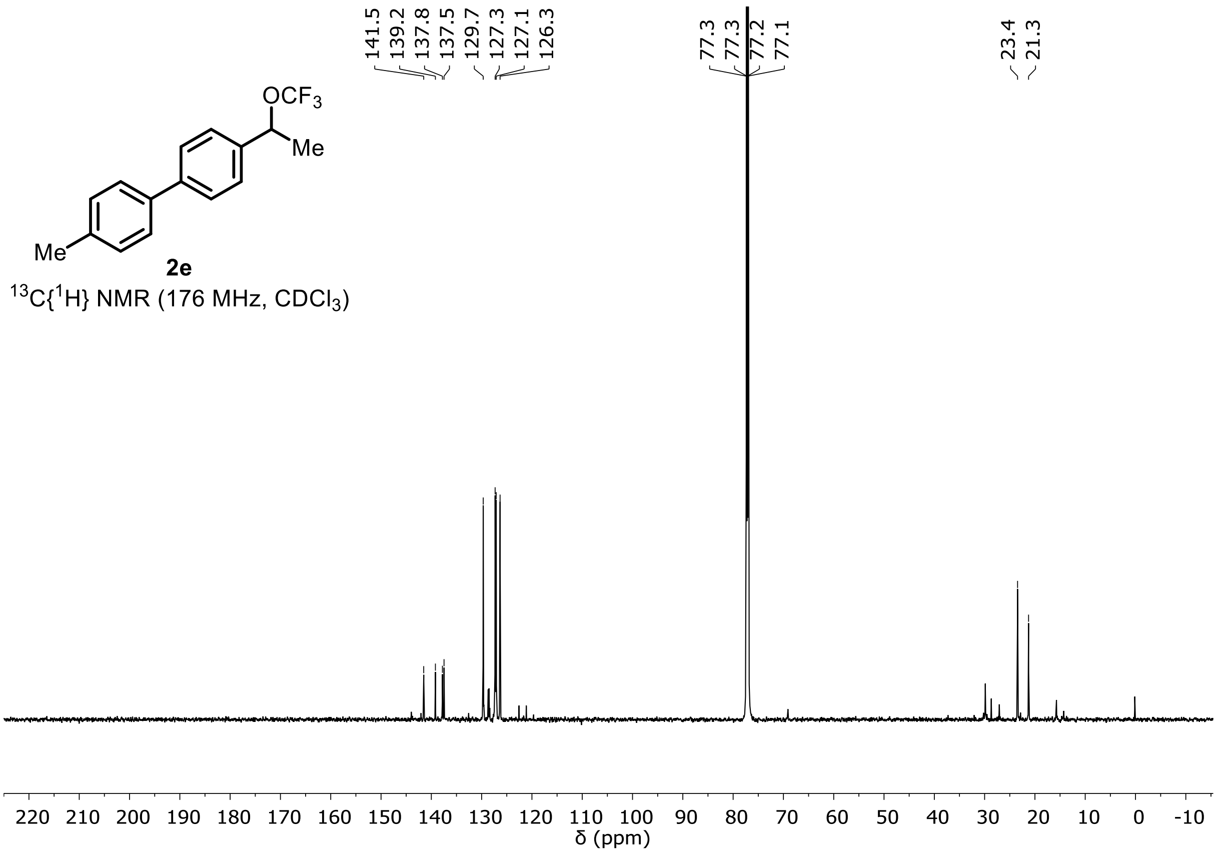
141.5 139.2 137.8 137.5 129.7 127.3 127.1 126.3
77.3 77.3 77.2 77.1
23.4 21.3
OCF3
Me
Me
2e
13C{1H} NMR (176 MHz, CDCl3)
220 210 200 190 180 170 160 150 140 130 120 110 100 90 80 70 60 50 40 30 20 10 0 -10
δ (ppm)

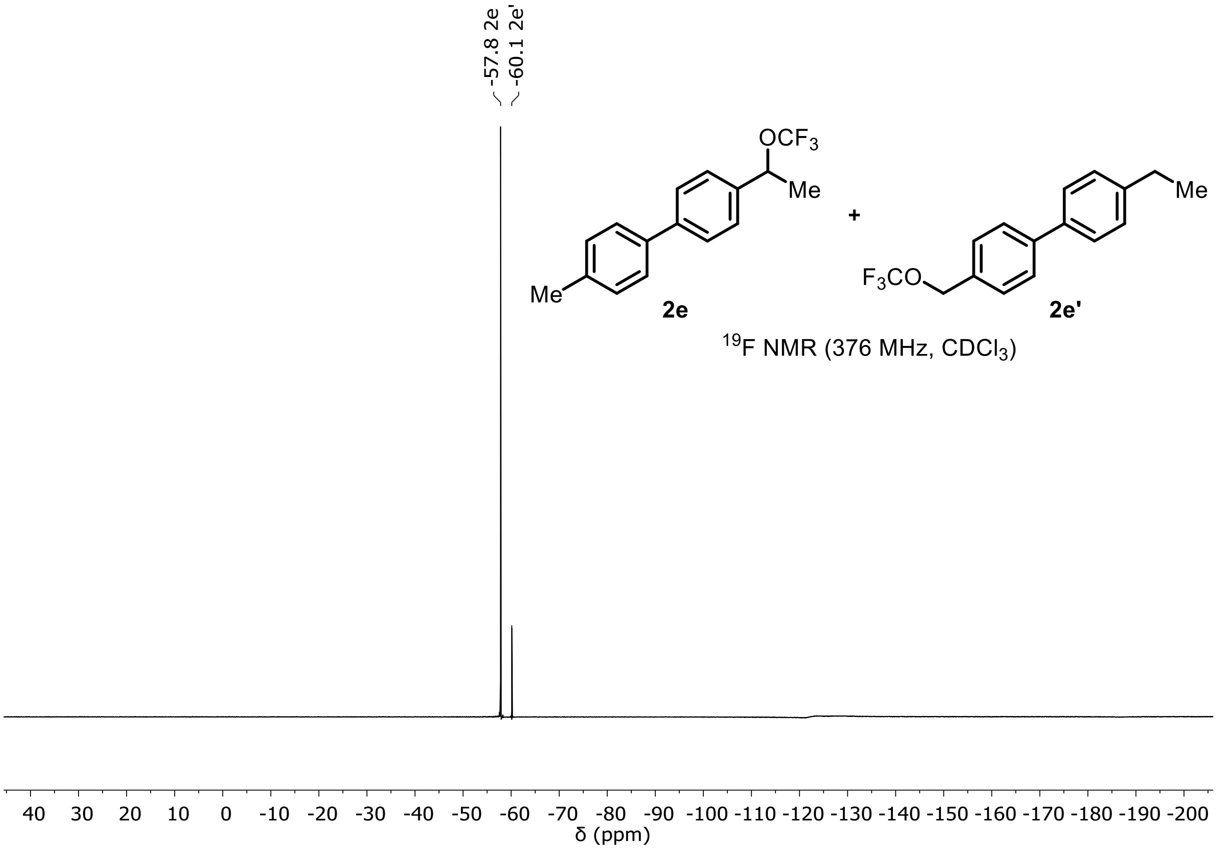
-57.8 2e
-60.1 2e'
OCF3
Me
+
Me
Me
F3CO
2e
2e'
19F NMR (376 MHz, CDCl3)
40 30 20 10 0 -10 -20 -30 -40 -50 -60 -70 -80 -90 -100 -110 -120 -130 -140 -150 -160 -170 -180 -190 -200
δ (ppm)

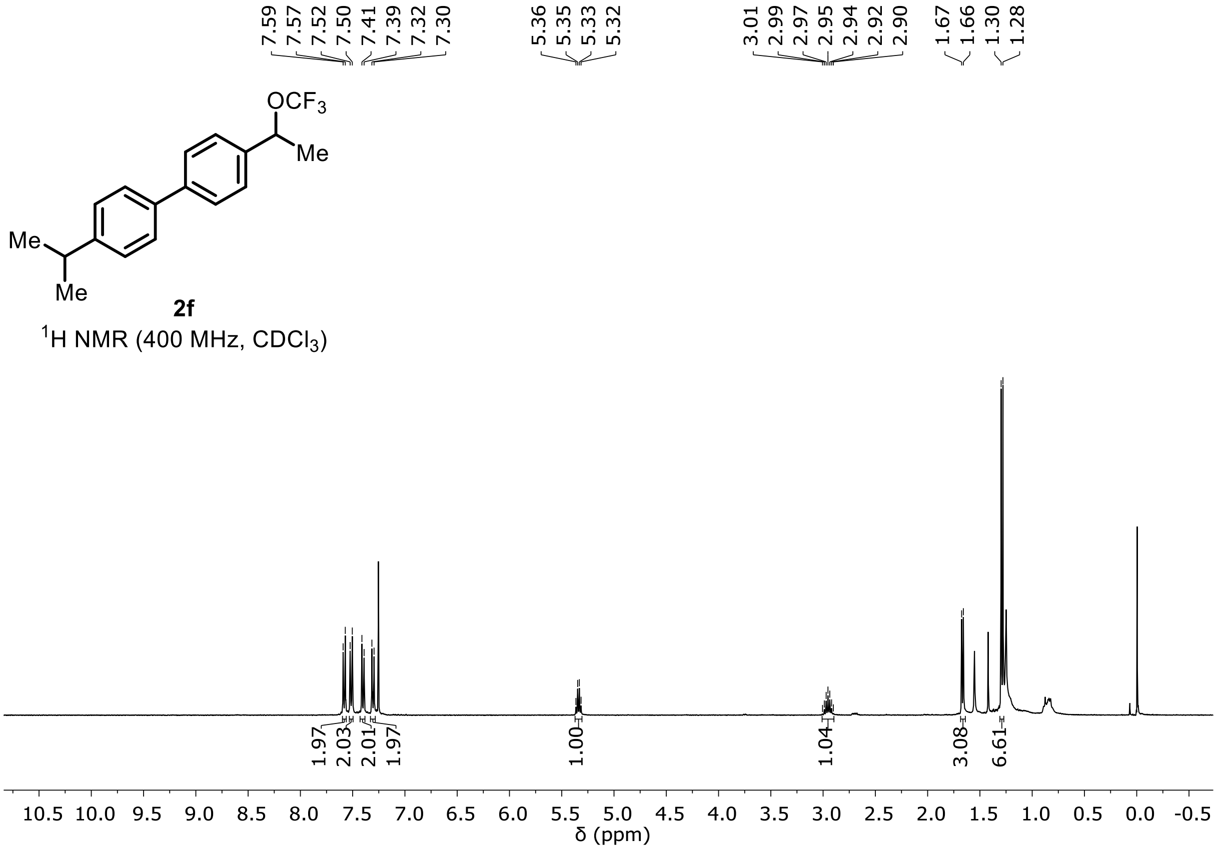

2f

^{1}H NMR (400 MHz, $CDCl_3$)

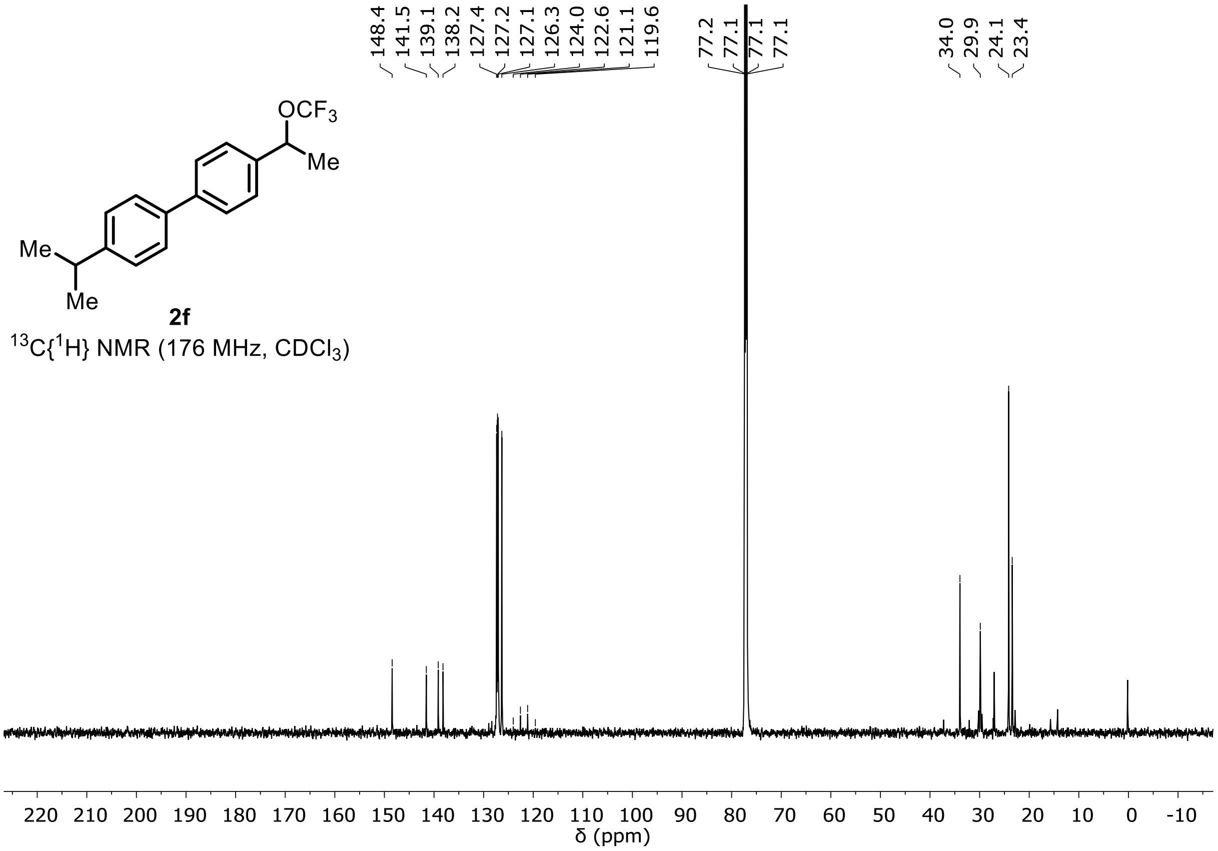

2f

$^{13}C\{^{1}H\}$ NMR (176 MHz, $CDCl_3$)

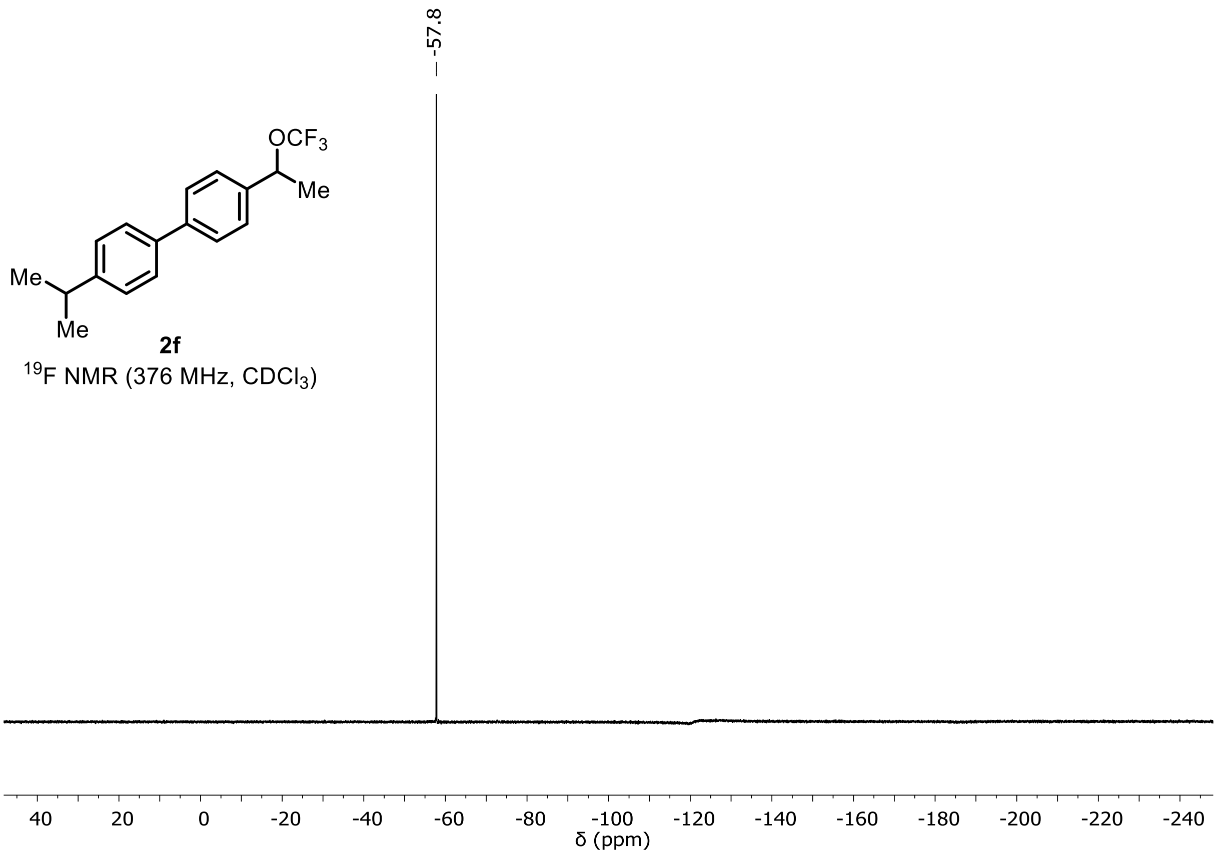

2f

^{19}F NMR (376 MHz, $CDCl_3$)

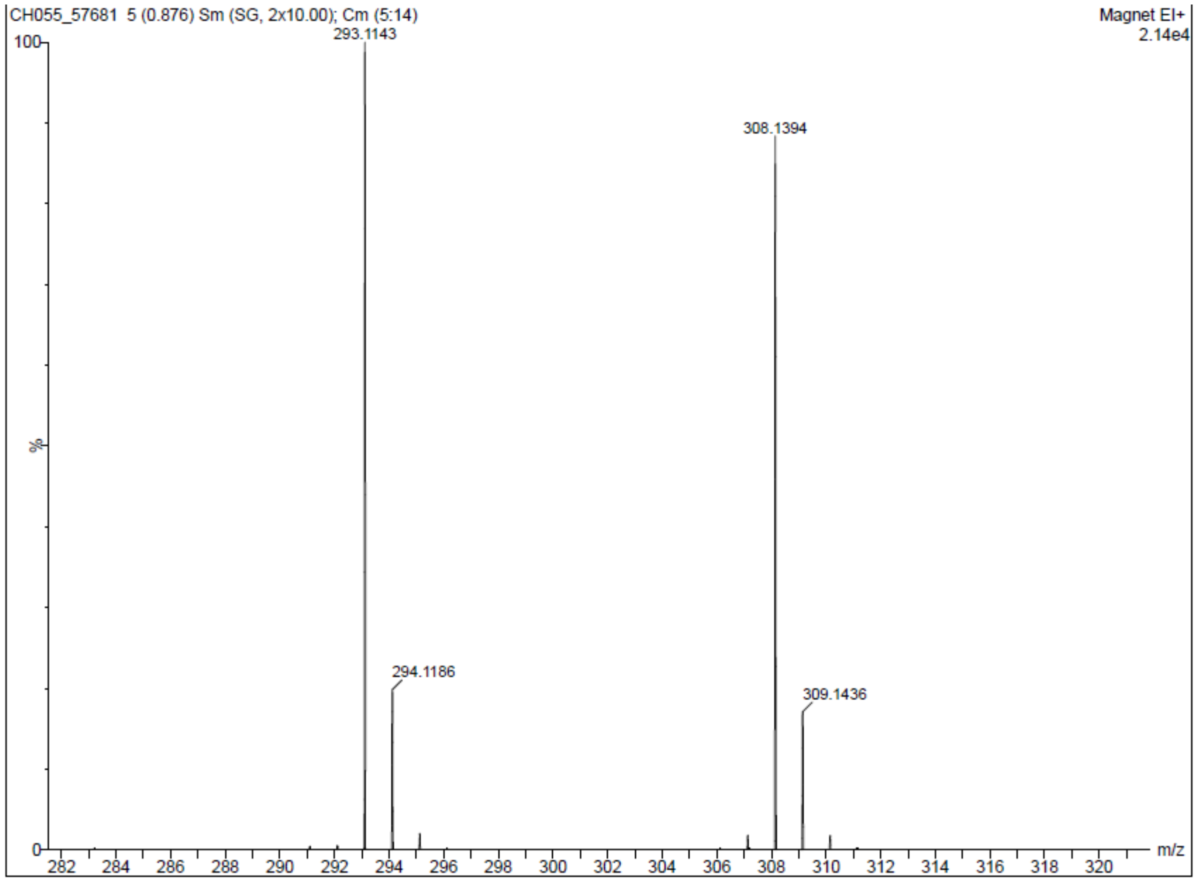

2g
^{1}H NMR (600 MHz, $CDCl_3$)

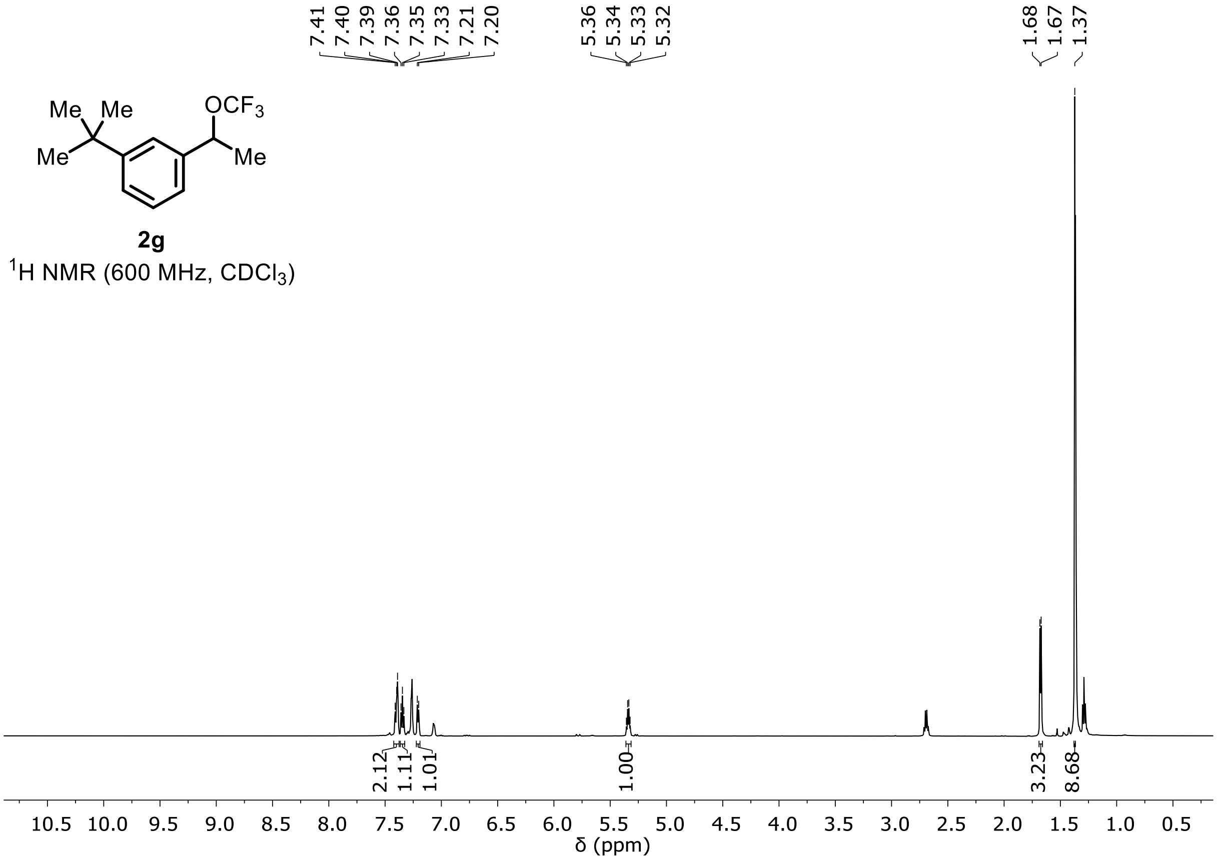

2g
$^{13}C\{^{1}H\}$ NMR (151 MHz, $CDCl_3$)

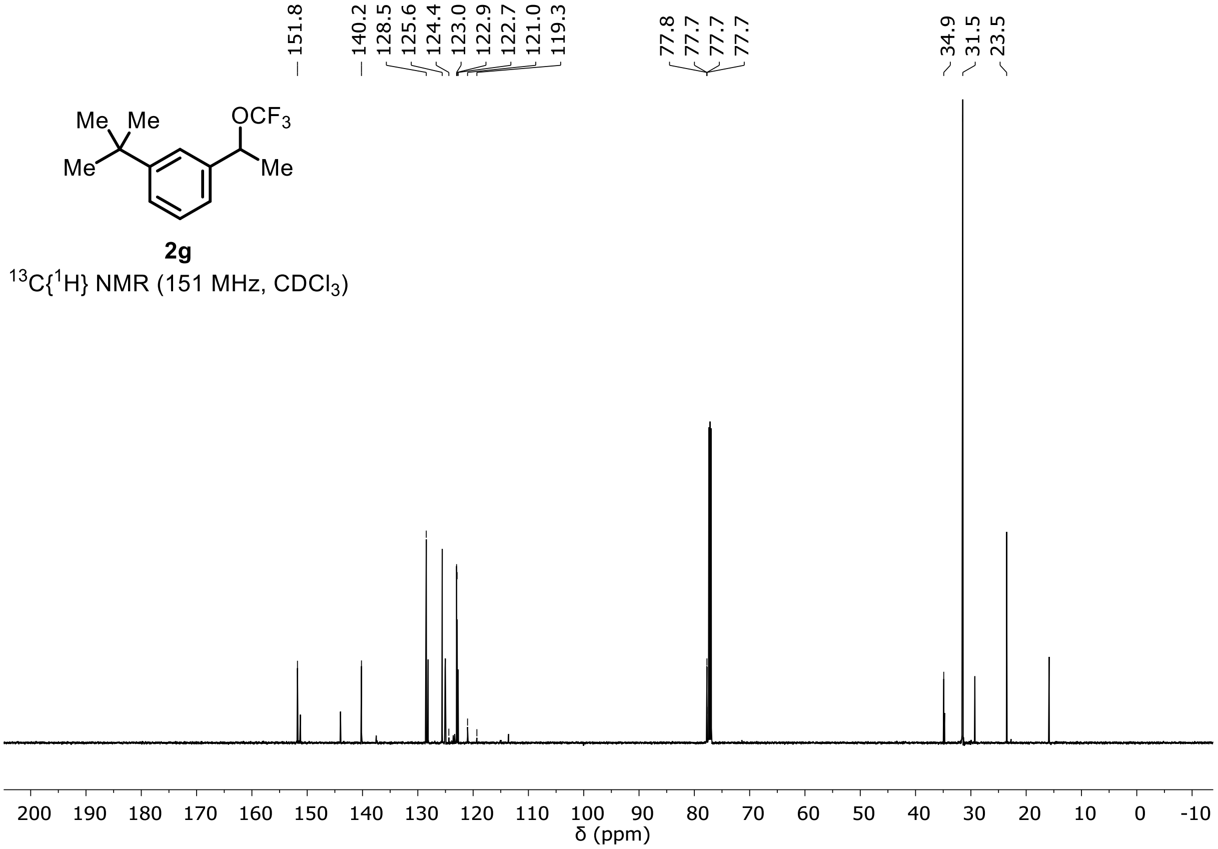

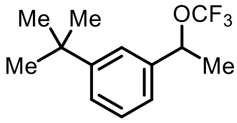

2g

^{19}F NMR (565 MHz, CDCl$_3$)

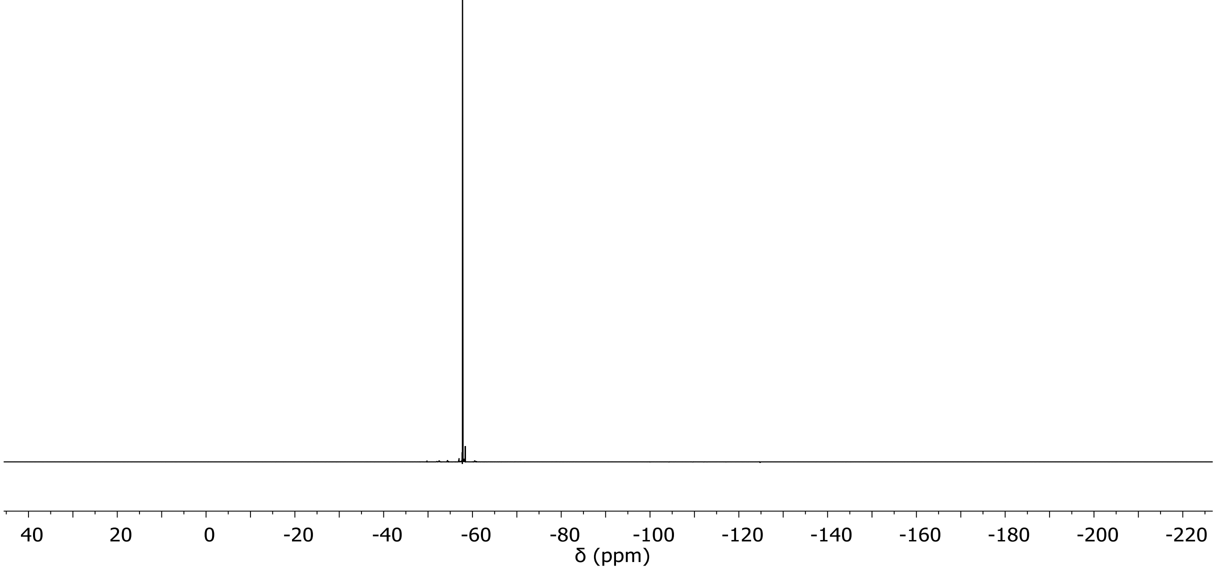

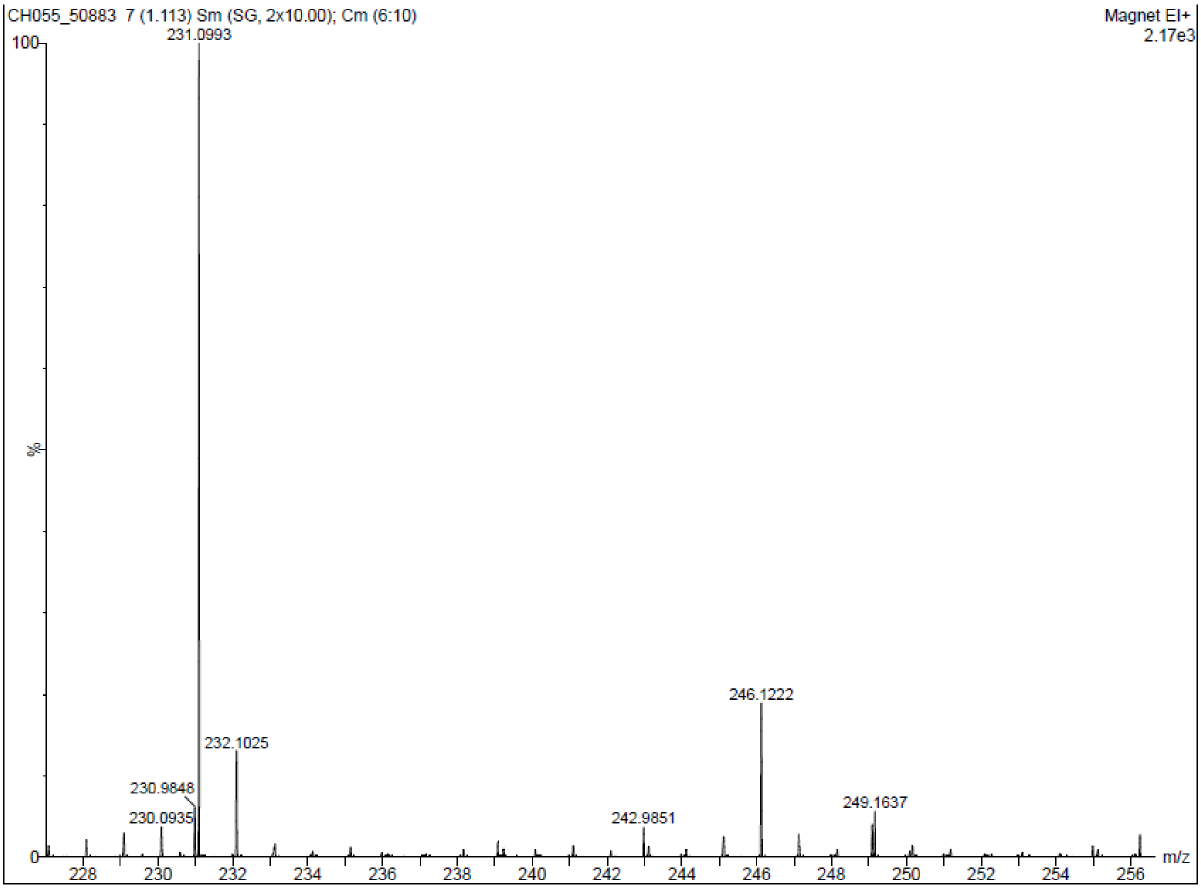

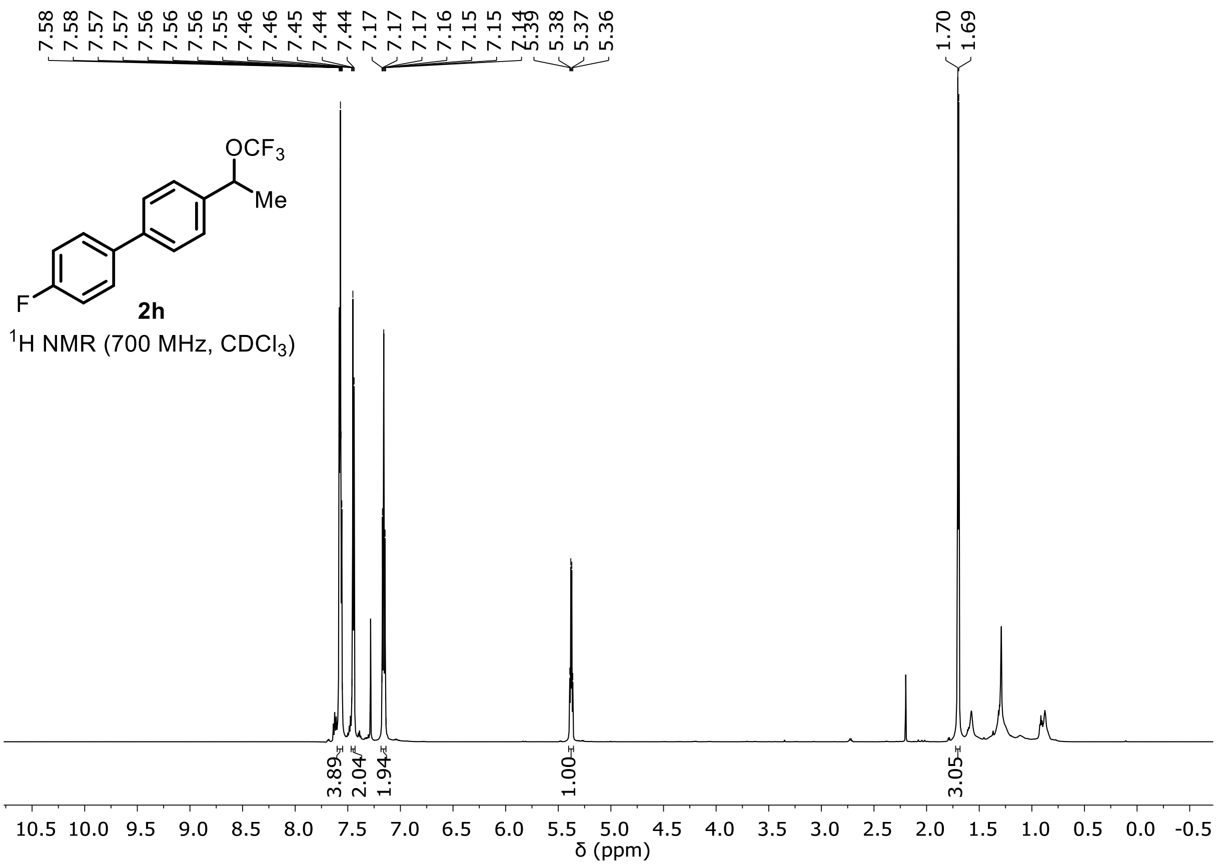

2h

^{1}H NMR (700 MHz, $CDCl_3$)

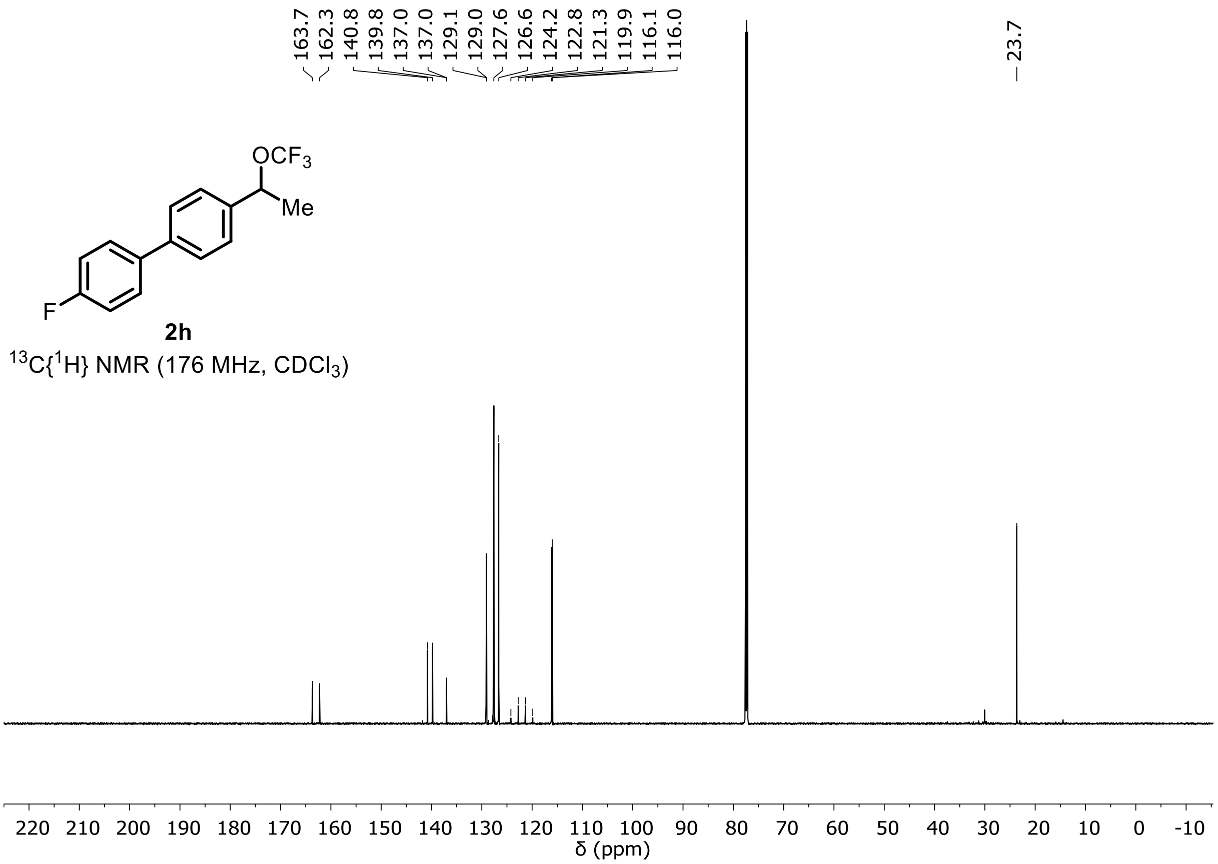

2h

$^{13}C\{^{1}H\}$ NMR (176 MHz, $CDCl_3$)

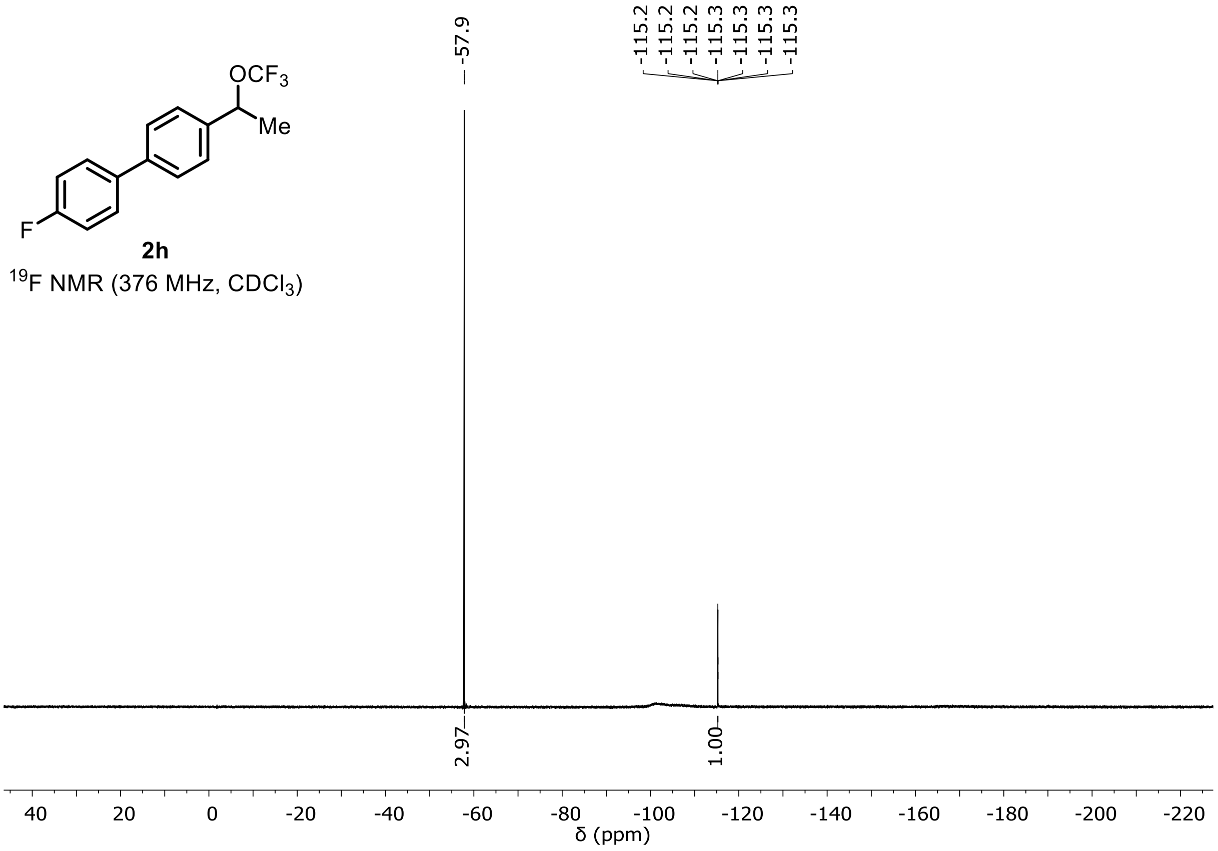

OCF3
Me
F
2h
19F NMR (376 MHz, CDCl3)
-57.9
-115.2
-115.2
-115.2
-115.3
-115.3
-115.3
-115.3
2.97
1.00
40 20 0 -20 -40 -60 -80 -100 -120 -140 -160 -180 -200 -220
δ (ppm)

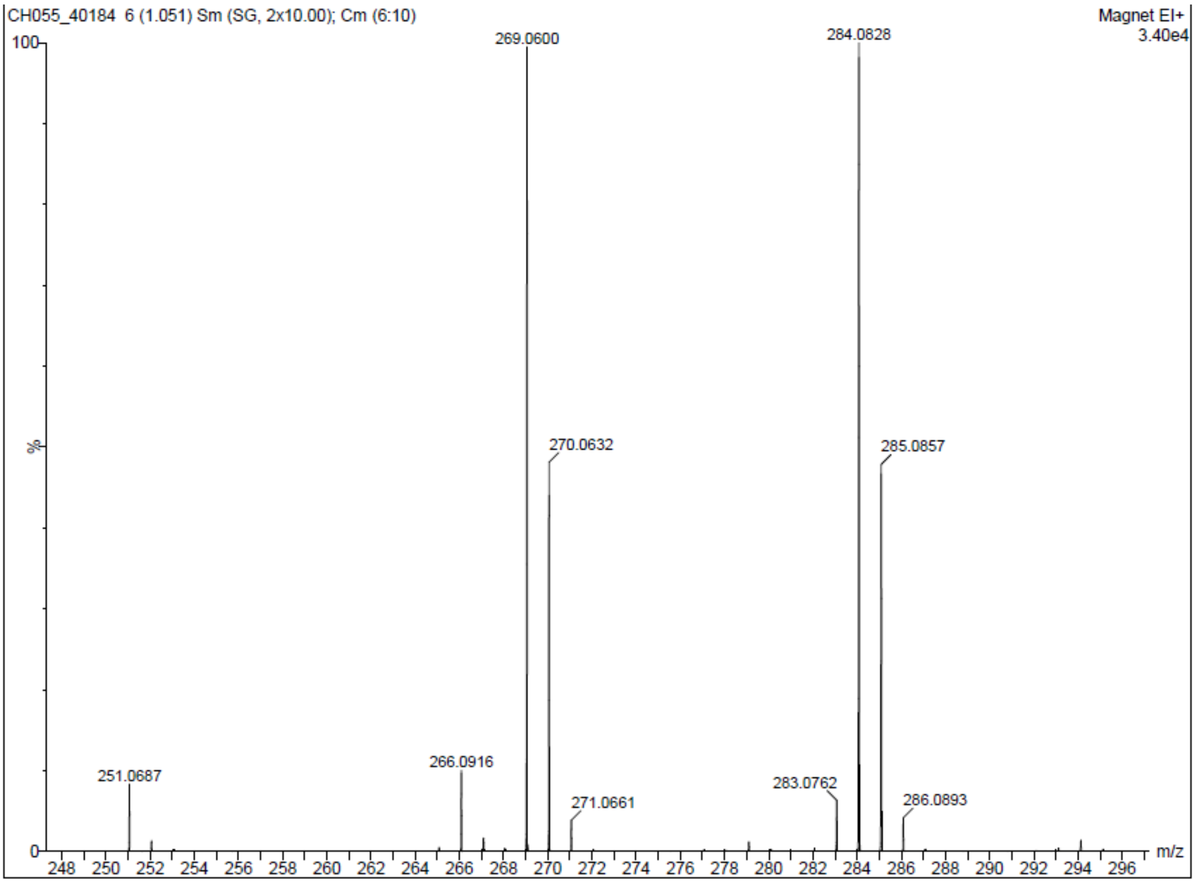

CH055_40184 6 (1.051) Sm (SG, 2x10.00); Cm (6:10)
Magnet EI+
3.40e4
100
%
0
269.0600
284.0828
270.0632
285.0857
251.0687
266.0916
271.0661
283.0762
286.0893
248 250 252 254 256 258 260 262 264 266 268 270 272 274 276 278 280 282 284 286 288 290 292 294 296
m/z

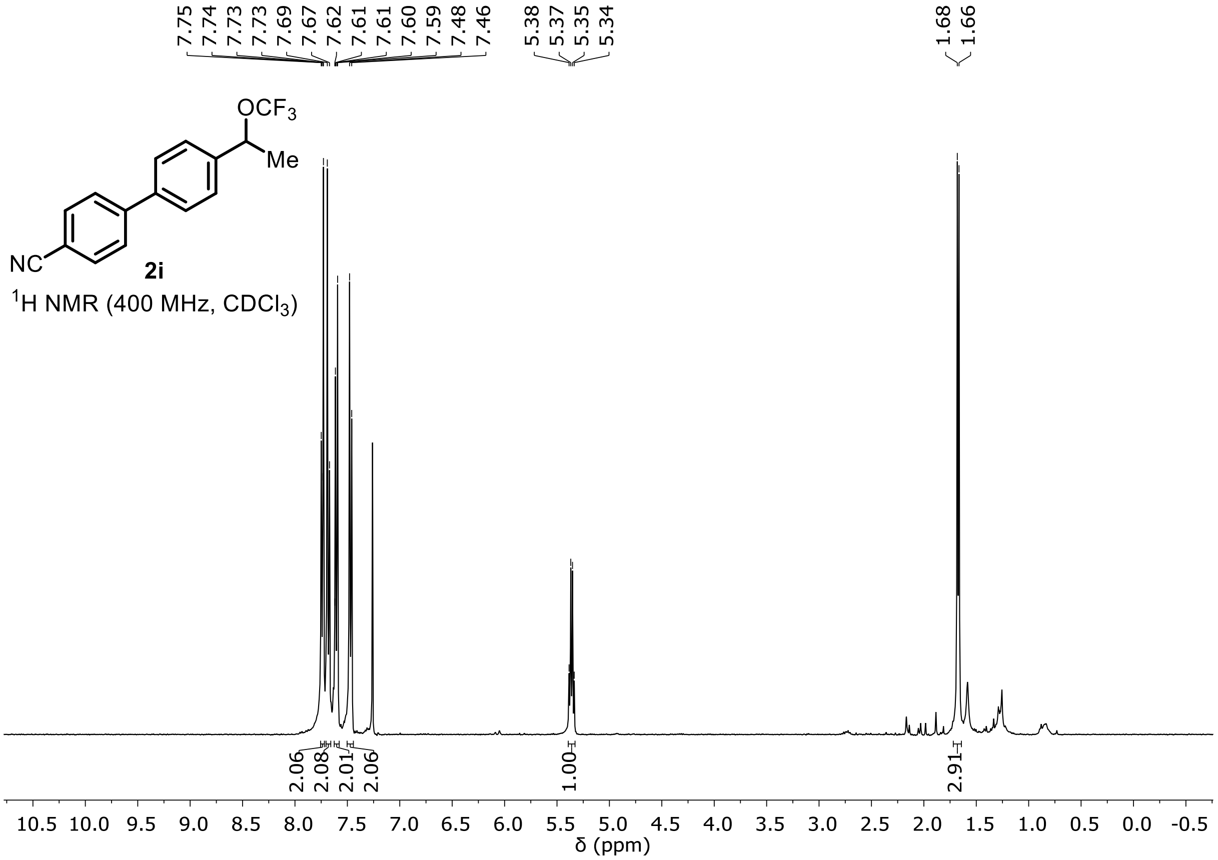
7.75 7.74 7.73 7.73 7.69 7.67 7.62 7.61 7.61 7.60 7.59 7.48 7.46
5.38 5.37 5.35 5.34
1.68 1.66
OCF$_3$
Me
NC
2i
^{1}H NMR (400 MHz, CDCl$_3$)
2.06 2.08 2.01 2.06
1.00
2.91
10.5 10.0 9.5 9.0 8.5 8.0 7.5 7.0 6.5 6.0 5.5 5.0 4.5 4.0 3.5 3.0 2.5 2.0 1.5 1.0 0.5 0.0 -0.5
δ (ppm)

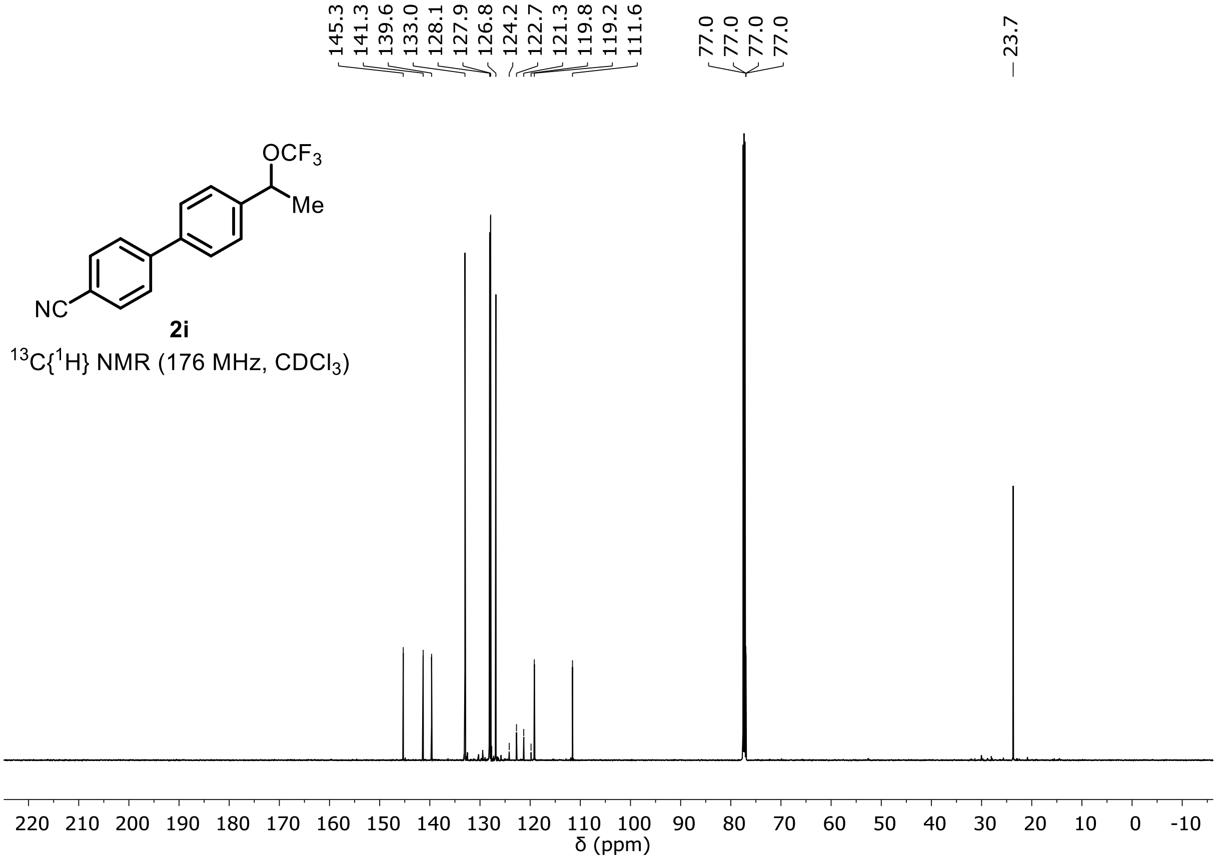
145.3 141.3 139.6 133.0 128.1 127.9 126.8 124.2 122.7 121.3 119.8 119.2 111.6
77.0 77.0 77.0 77.0
23.7
OCF$_3$
Me
NC
2i
^{13}C{^{1}H} NMR (176 MHz, CDCl$_3$)
220 210 200 190 180 170 160 150 140 130 120 110 100 90 80 70 60 50 40 30 20 10 0 -10
δ (ppm)

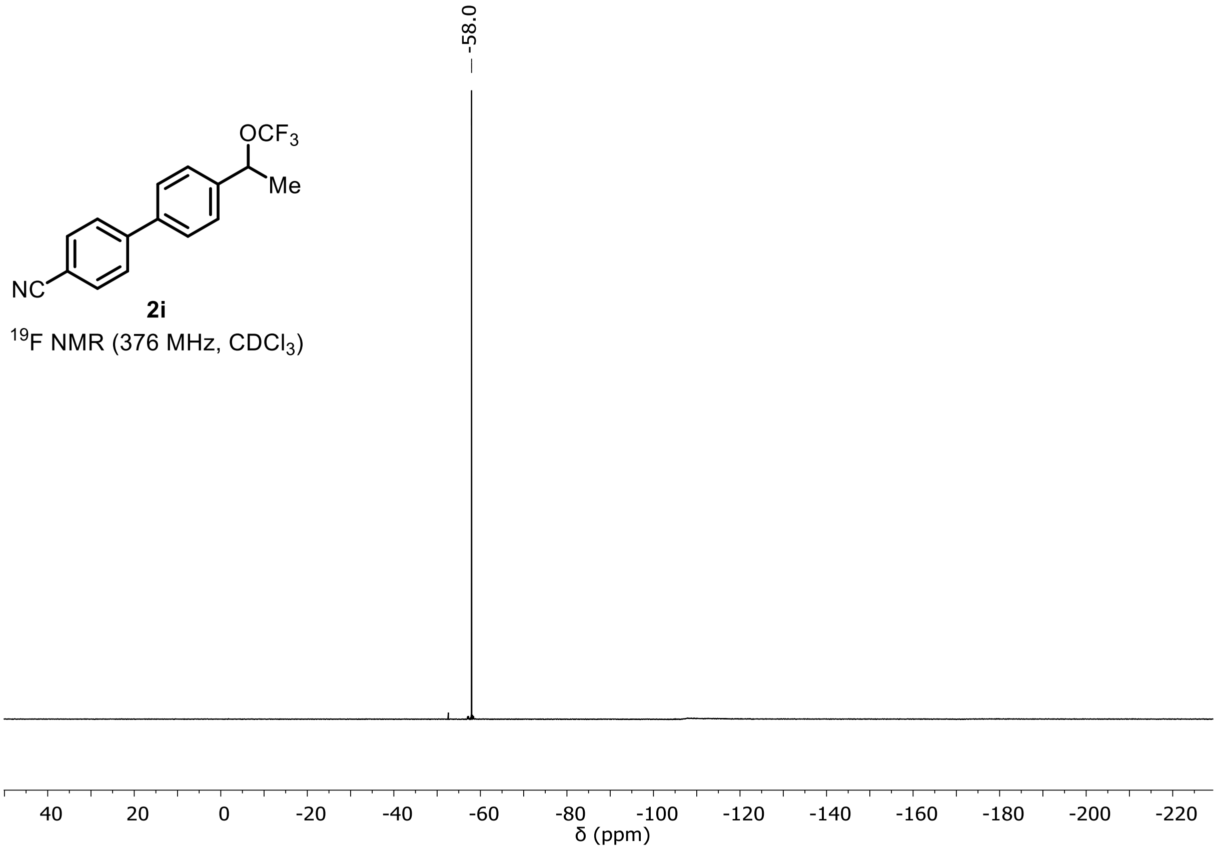

2i

^{19}F NMR (376 MHz, $CDCl_3$)

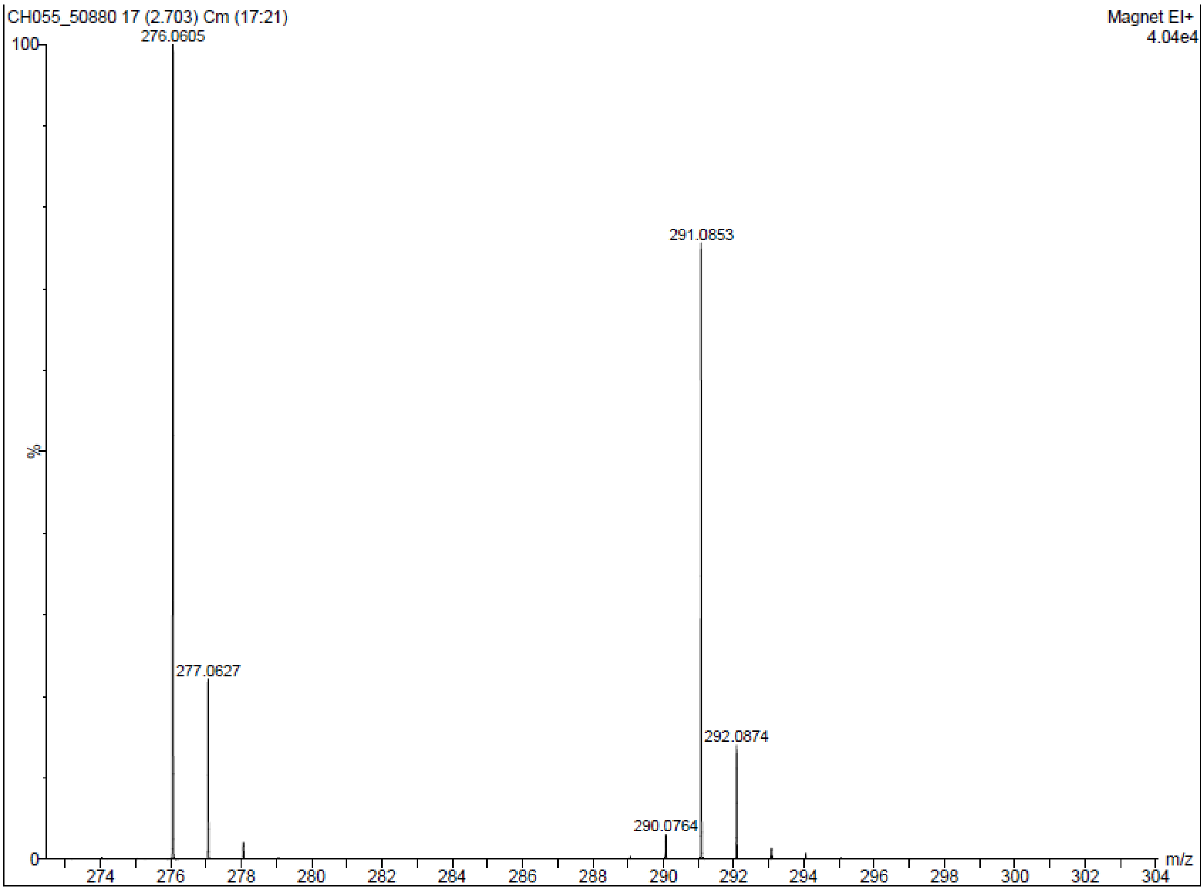

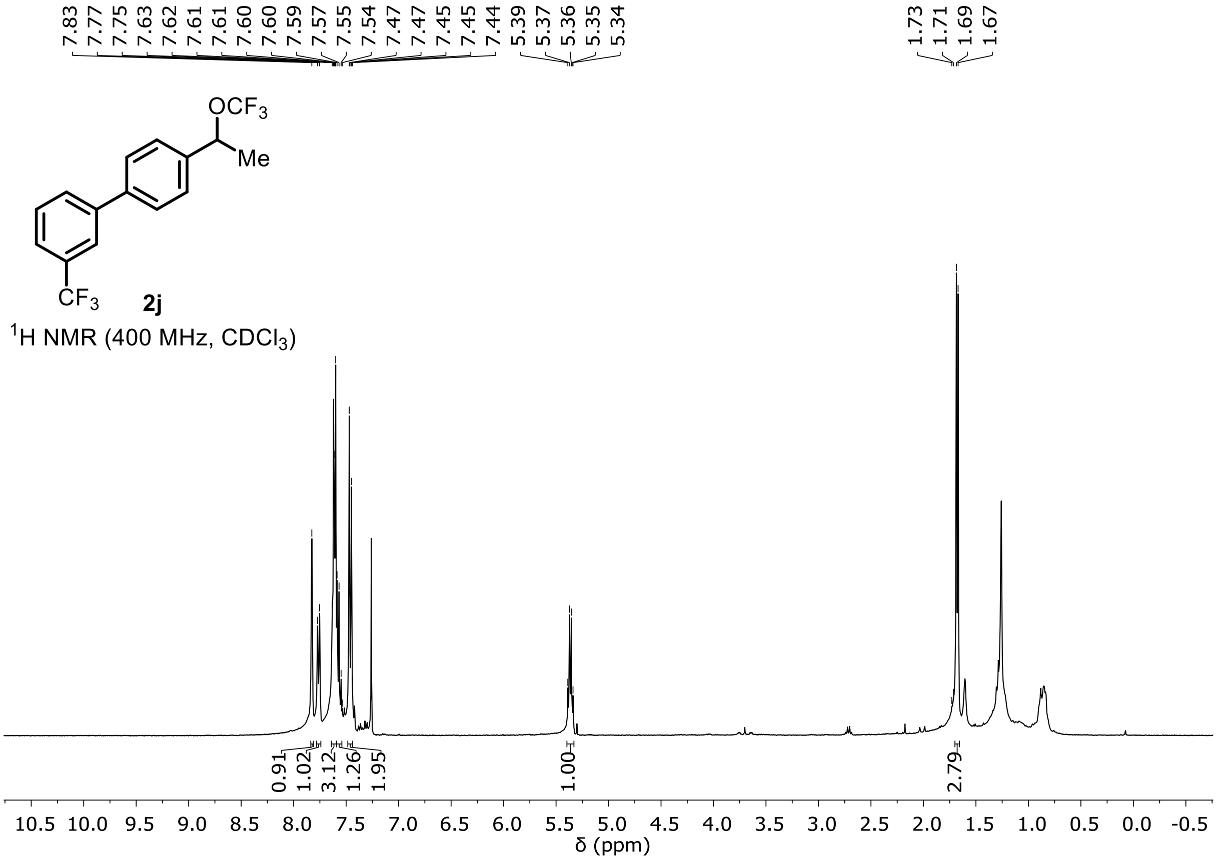
7.83 7.77 7.75 7.63 7.62 7.61 7.60 7.60 7.59 7.57 7.55 7.54 7.47 7.47 7.45 7.45 7.44 5.39 5.37 5.36 5.35 5.34
1.73 1.71 1.69 1.67
OCF3
Me
CF3
2j
1H NMR (400 MHz, CDCl3)
0.91 1.02 3.12 1.26 1.95
1.00
2.79
10.5 10.0 9.5 9.0 8.5 8.0 7.5 7.0 6.5 6.0 5.5 5.0 4.5 4.0 3.5 3.0 2.5 2.0 1.5 1.0 0.5 0.0 -0.5
δ (ppm)

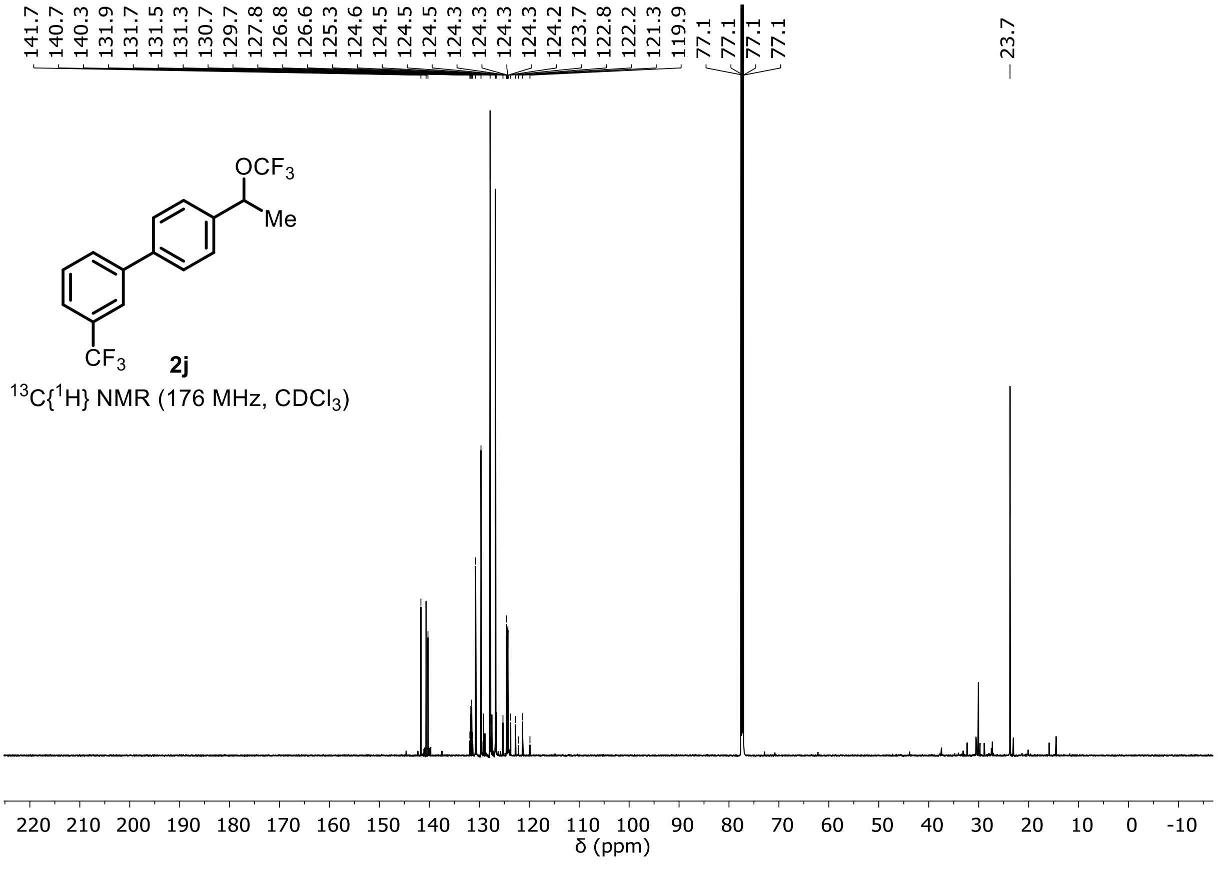
141.7 140.7 140.3 131.9 131.7 131.5 131.3 130.7 129.7 127.8 126.8 126.6 125.3 124.6 124.5 124.5 124.5 124.3 124.3 124.3 124.3 124.2 123.7 122.8 122.2 121.3 119.9 77.1 77.1 77.1 77.1
23.7
OCF3
Me
CF3
2j
13C{1H} NMR (176 MHz, CDCl3)
220 210 200 190 180 170 160 150 140 130 120 110 100 90 80 70 60 50 40 30 20 10 0 -10
δ (ppm)

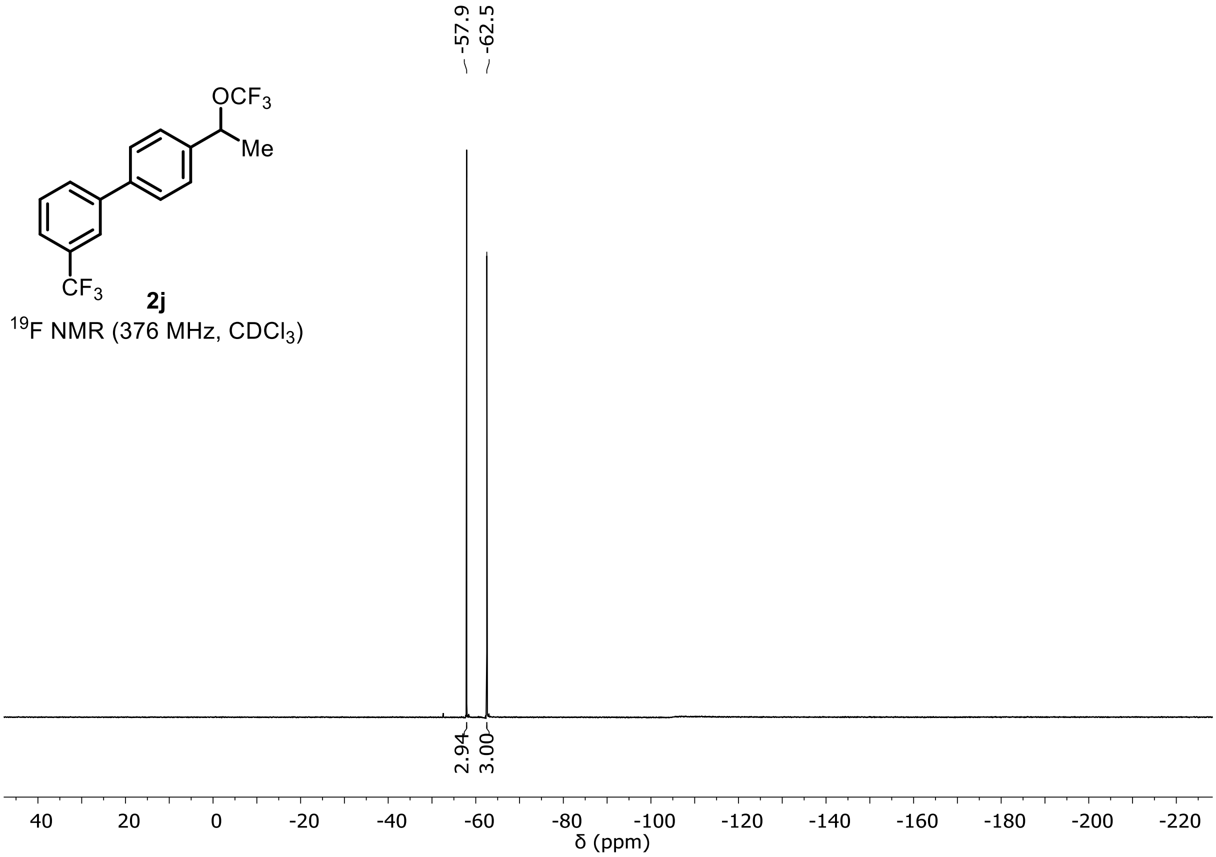
OCF3
Me
CF3
2j
19F NMR (376 MHz, CDCl3)
-57.9
-62.5
2.94
3.00
40
20
0
-20
-40
-60
-80
-100
-120
-140
-160
-180
-200
-220
δ (ppm)

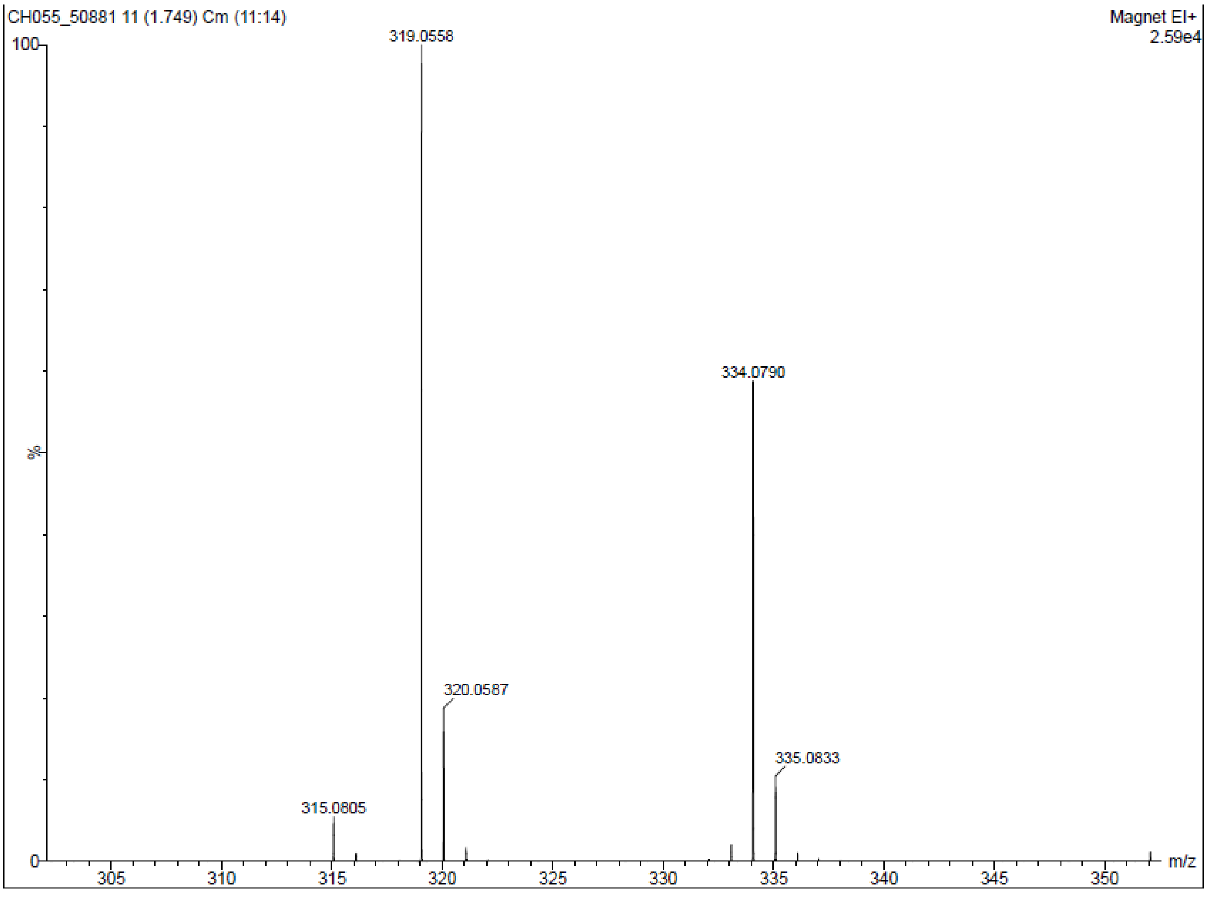
CH055_50881 11 (1.749) Cm (11:14)
Magnet EI+
2.59e4
100
%
0
319.0558
334.0790
320.0587
335.0833
315.0805
305
310
315
320
325
330
335
340
345
350
m/z

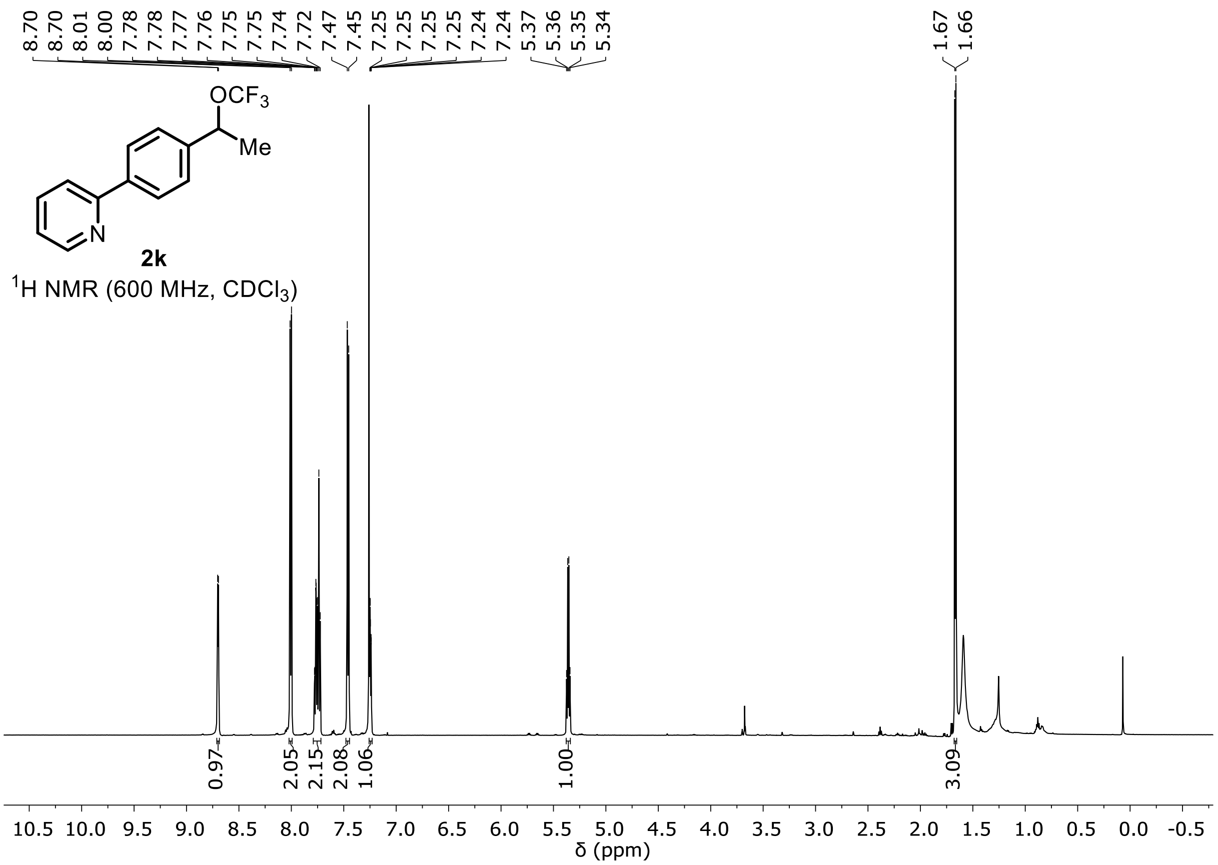
8.70
8.70
8.01
8.00
7.78
7.78
7.77
7.76
7.75
7.75
7.74
7.72
7.47
7.45
7.25
7.25
7.25
7.25
7.24
7.24
5.37
5.36
5.35
5.34
1.67
1.66
OCF3
Me
N
2k
1H NMR (600 MHz, CDCl3)
0.97
2.05
2.15
2.08
1.06
1.00
3.09
10.5 10.0 9.5 9.0 8.5 8.0 7.5 7.0 6.5 6.0 5.5 5.0 4.5 4.0 3.5 3.0 2.5 2.0 1.5 1.0 0.5 0.0 -0.5
δ (ppm)

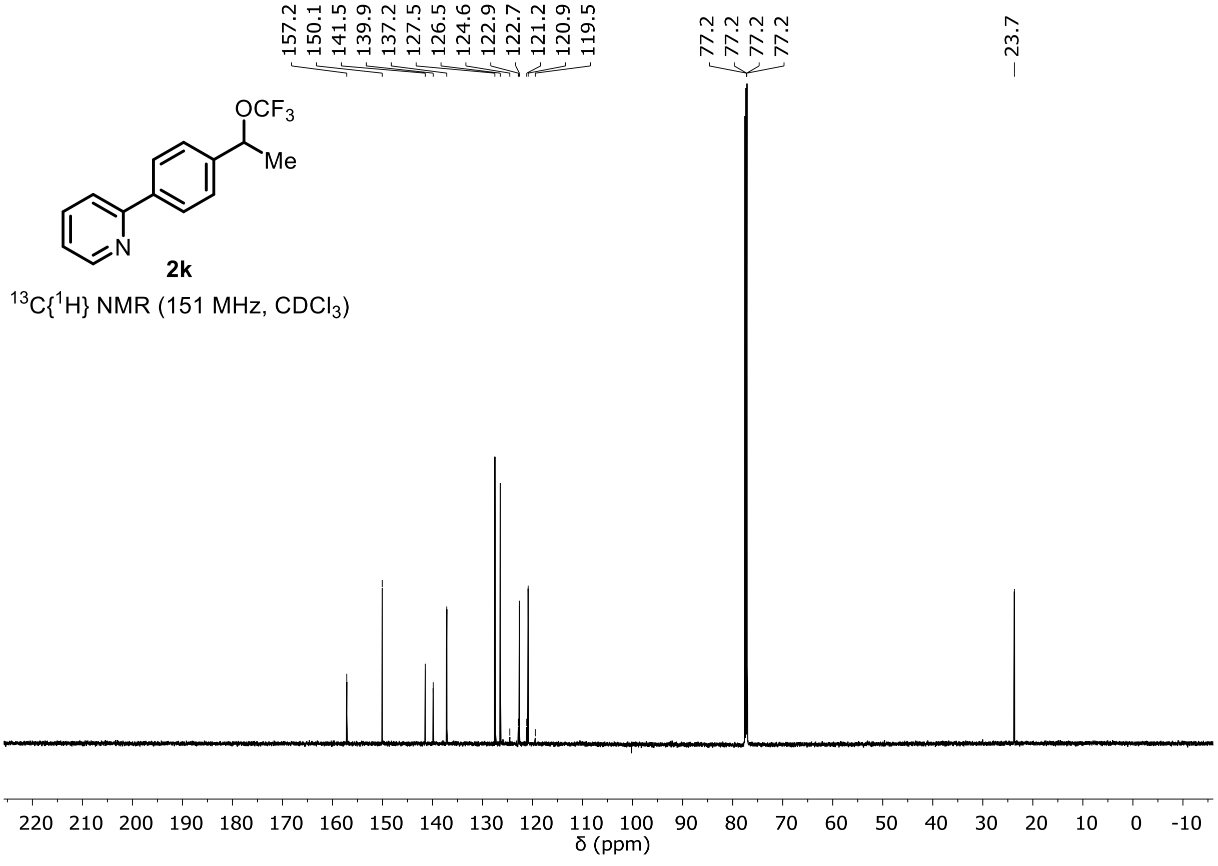
157.2
150.1
141.5
139.9
137.2
127.5
126.5
124.6
122.9
122.7
121.2
120.9
119.5
77.2
77.2
77.2
77.2
23.7
OCF3
Me
N
2k
13C{1H} NMR (151 MHz, CDCl3)
220 210 200 190 180 170 160 150 140 130 120 110 100 90 80 70 60 50 40 30 20 10 0 -10
δ (ppm)

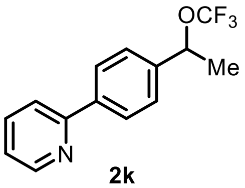

2k

^{19}F NMR (565 MHz, $CDCl_3$)

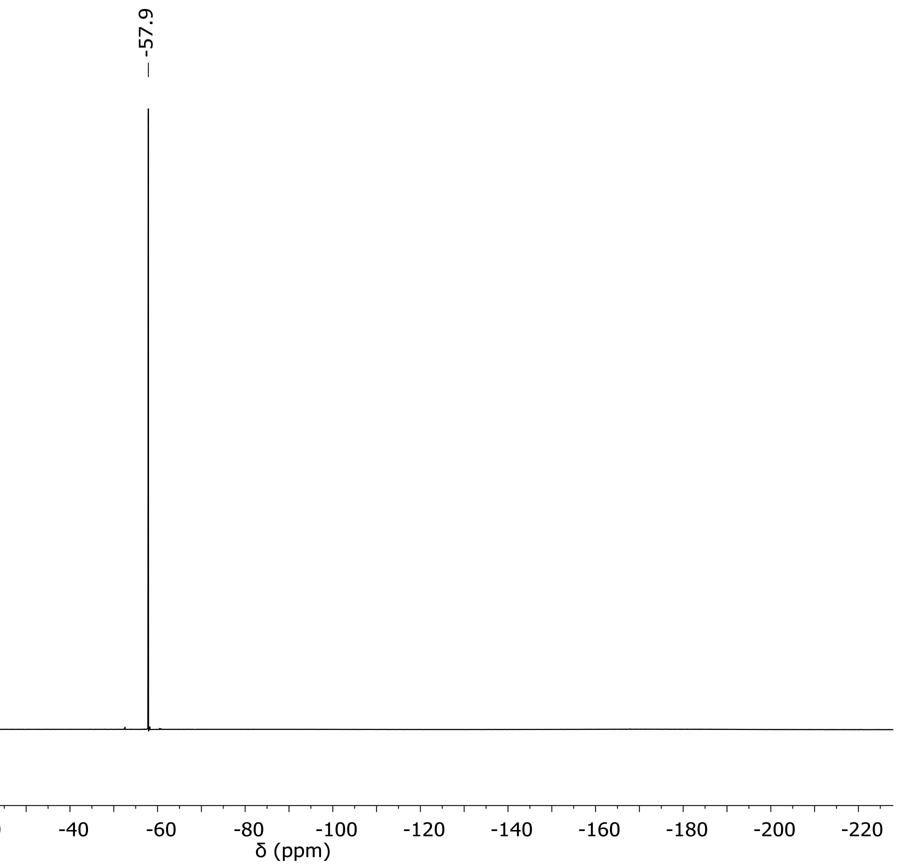

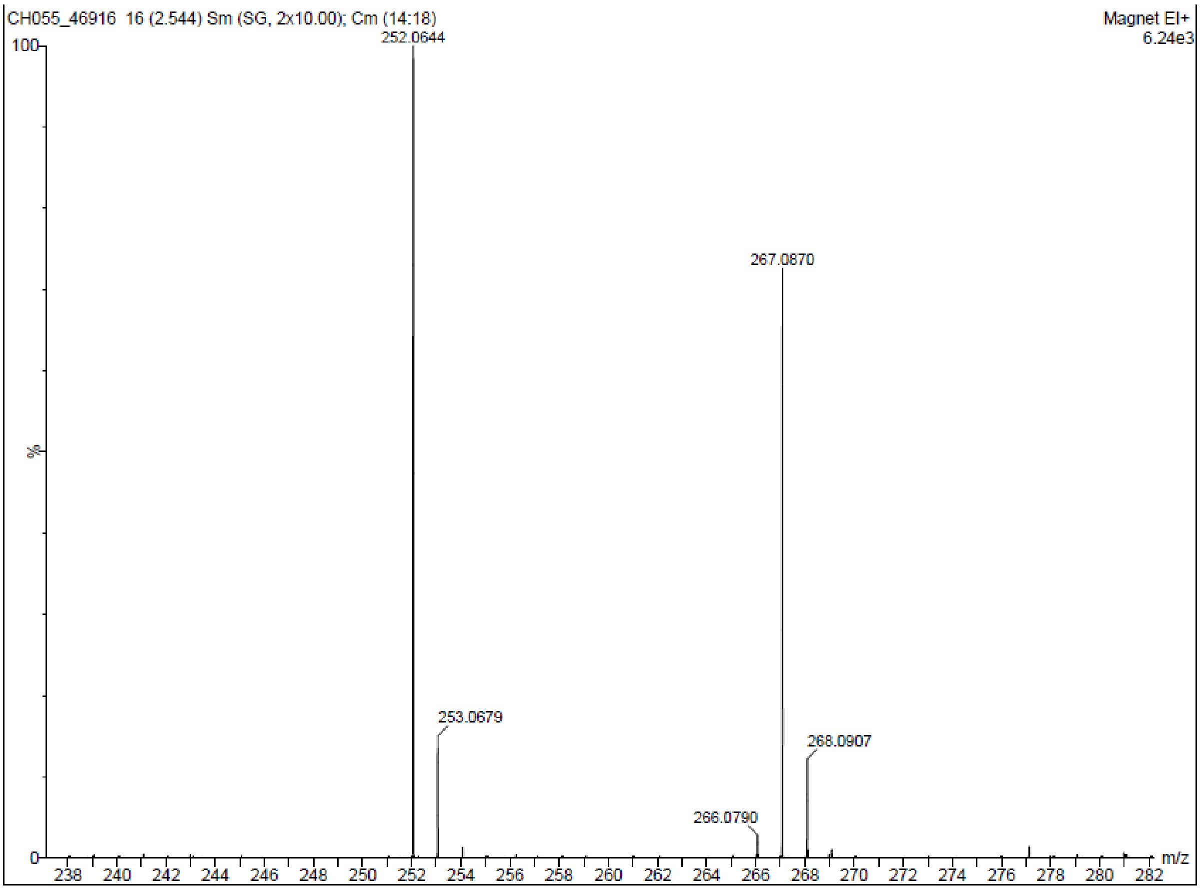

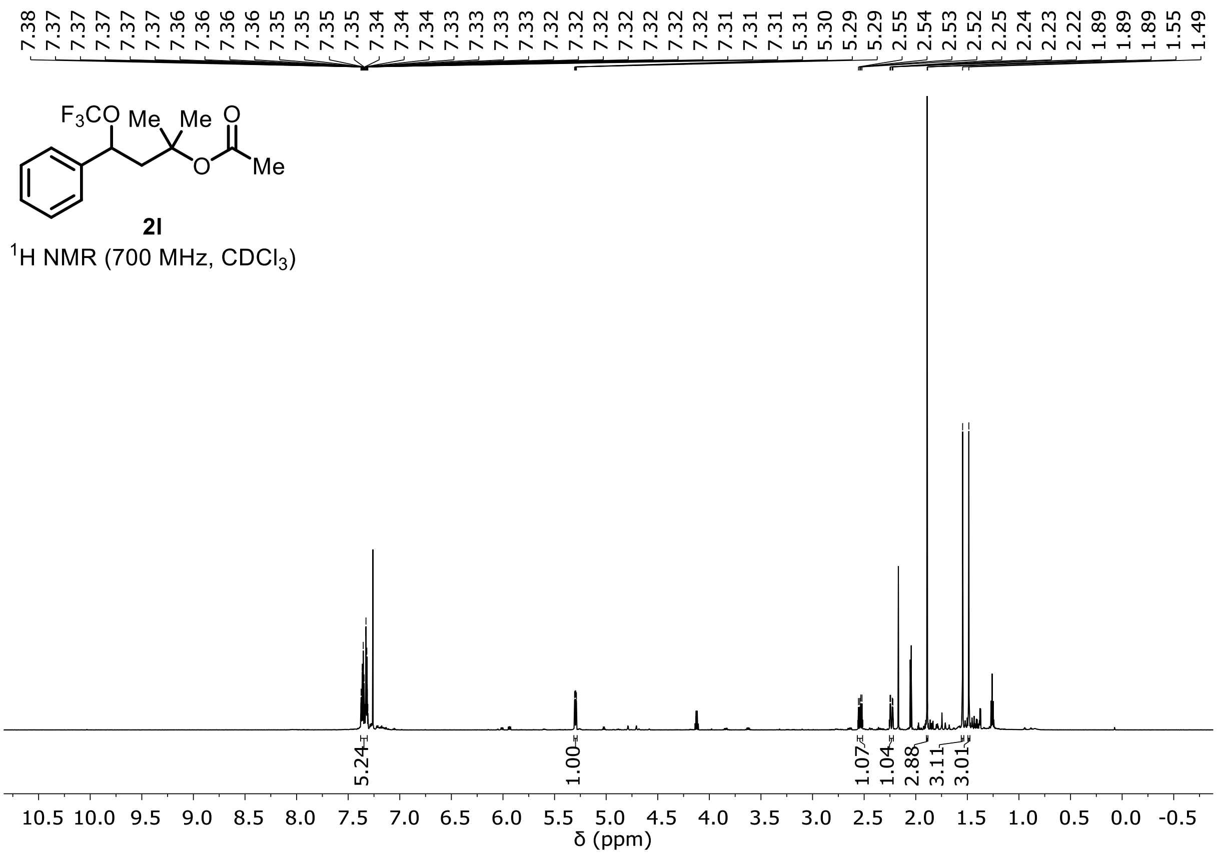
7.38 7.37 7.37 7.37 7.37 7.37 7.36 7.36 7.36 7.36 7.35 7.35 7.35 7.35 7.34 7.34 7.34 7.33 7.33 7.33 7.33 7.32 7.32 7.32 7.32 7.32 7.32 7.32 7.31 7.31 7.31 5.31 5.30 5.29 5.29 2.55 2.54 2.53 2.52 2.25 2.24 2.23 2.22 1.89 1.89 1.89 1.55 1.49
F3CO Me Me O
O Me
2l
1H NMR (700 MHz, CDCl3)
5.24 1.00 1.07 1.04 2.88 3.11 3.01
10.5 10.0 9.5 9.0 8.5 8.0 7.5 7.0 6.5 6.0 5.5 5.0 4.5 4.0 3.5 3.0 2.5 2.0 1.5 1.0 0.5 0.0 -0.5
δ (ppm)

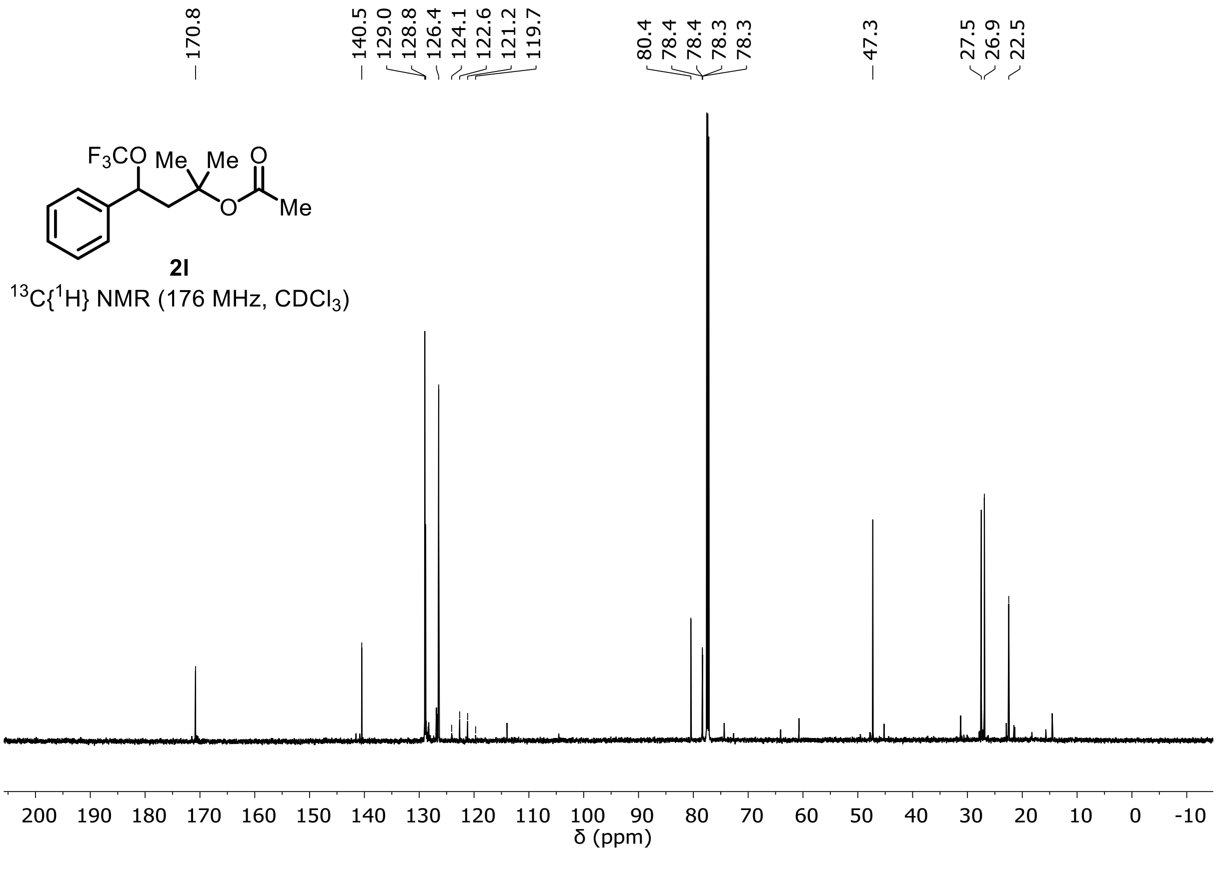
170.8 140.5 129.0 128.8 126.4 124.1 122.6 121.2 119.7 80.4 78.4 78.4 78.3 78.3 47.3 27.5 26.9 22.5
F3CO Me Me O
O Me
2l
13C{1H} NMR (176 MHz, CDCl3)
200 190 180 170 160 150 140 130 120 110 100 90 80 70 60 50 40 30 20 10 0 -10
δ (ppm)

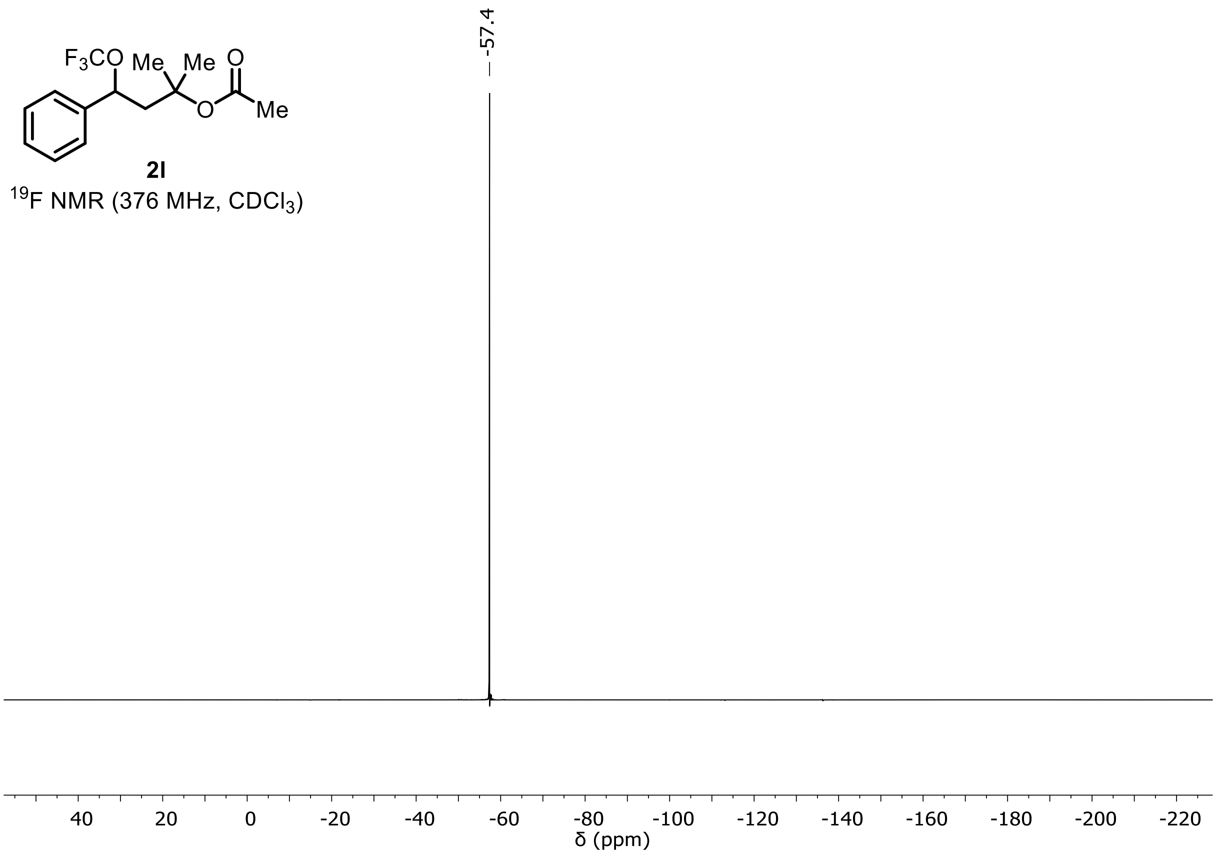
F3CO Me Me O
O
Me
2l
19F NMR (376 MHz, CDCl3)
-57.4
40 20 0 -20 -40 -60 -80 -100 -120 -140 -160 -180 -200 -220
δ (ppm)

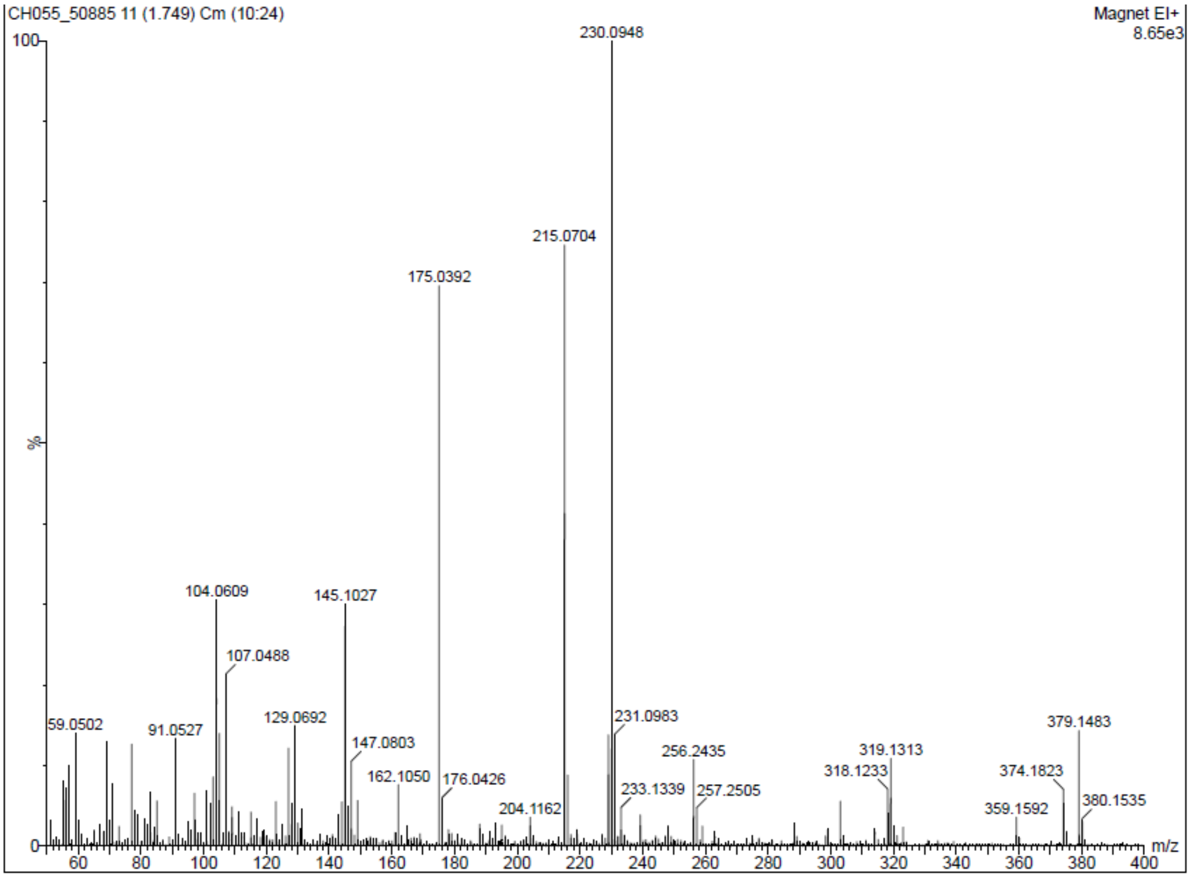
CH055_50885 11 (1.749) Cm (10:24)
Magnet EI+
8.65e3
230.0948
215.0704
175.0392
104.0609
145.1027
107.0488
59.0502
91.0527
129.0692
147.0803
231.0983
256.2435
319.1313
318.1233
379.1483
374.1823
162.1050
176.0426
233.1339
257.2505
380.1535
204.1162
359.1592
%
m/z

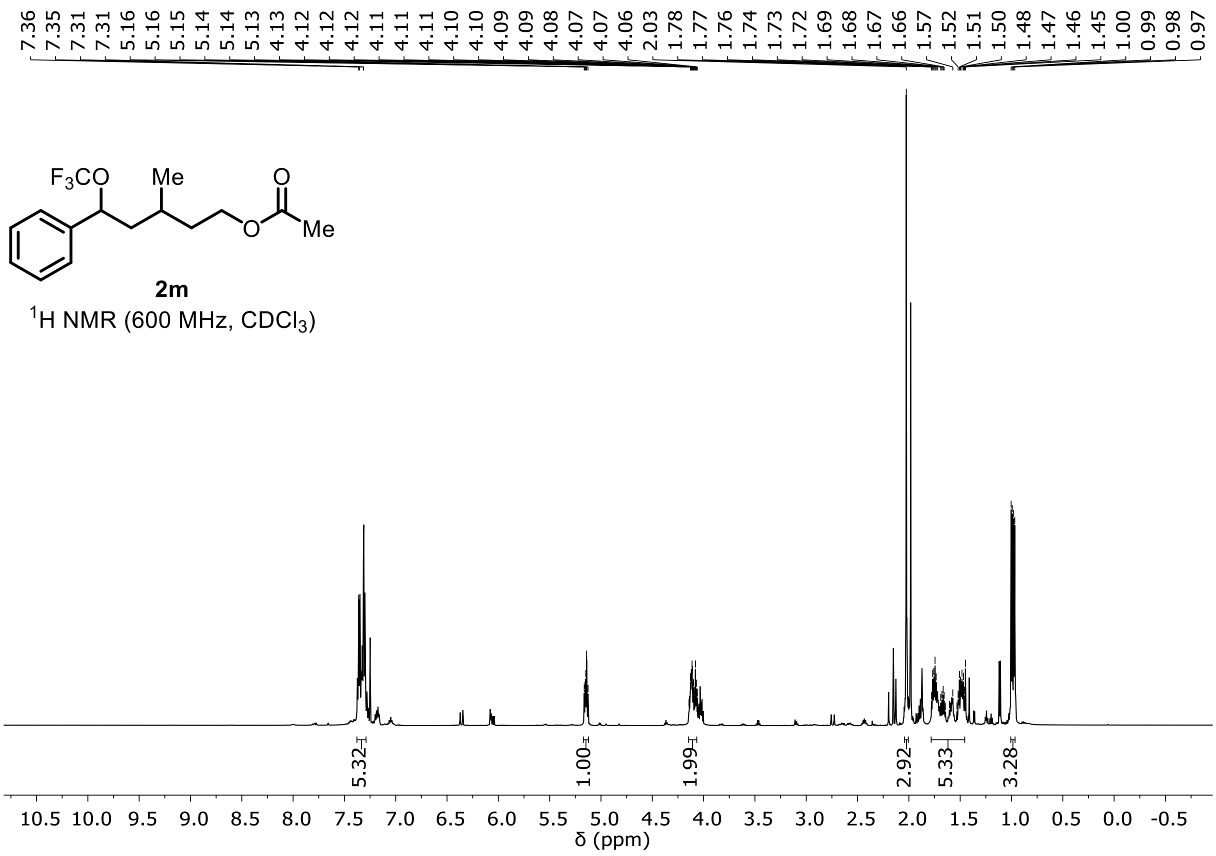

2m

^{1}H NMR (600 MHz, $CDCl_3$)

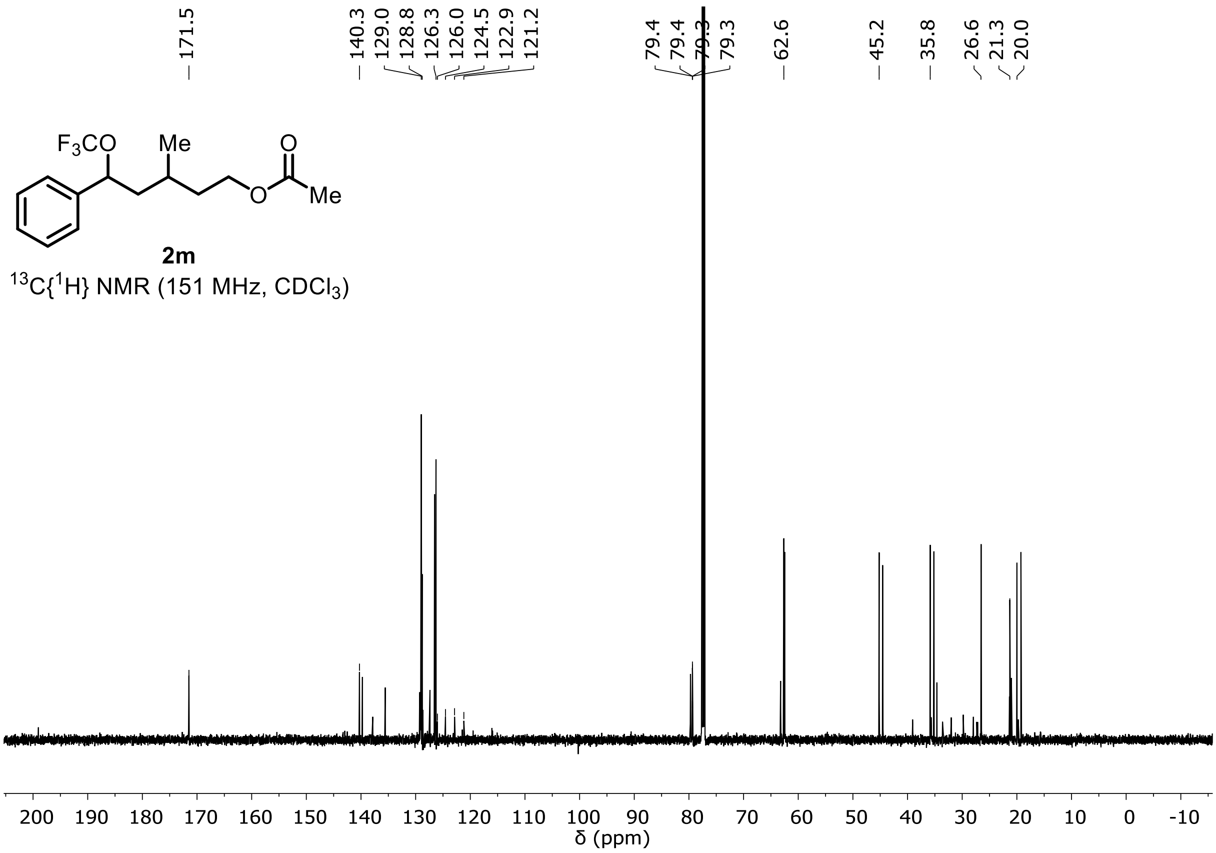

2m

$^{13}C\{^{1}H\}$ NMR (151 MHz, $CDCl_3$)

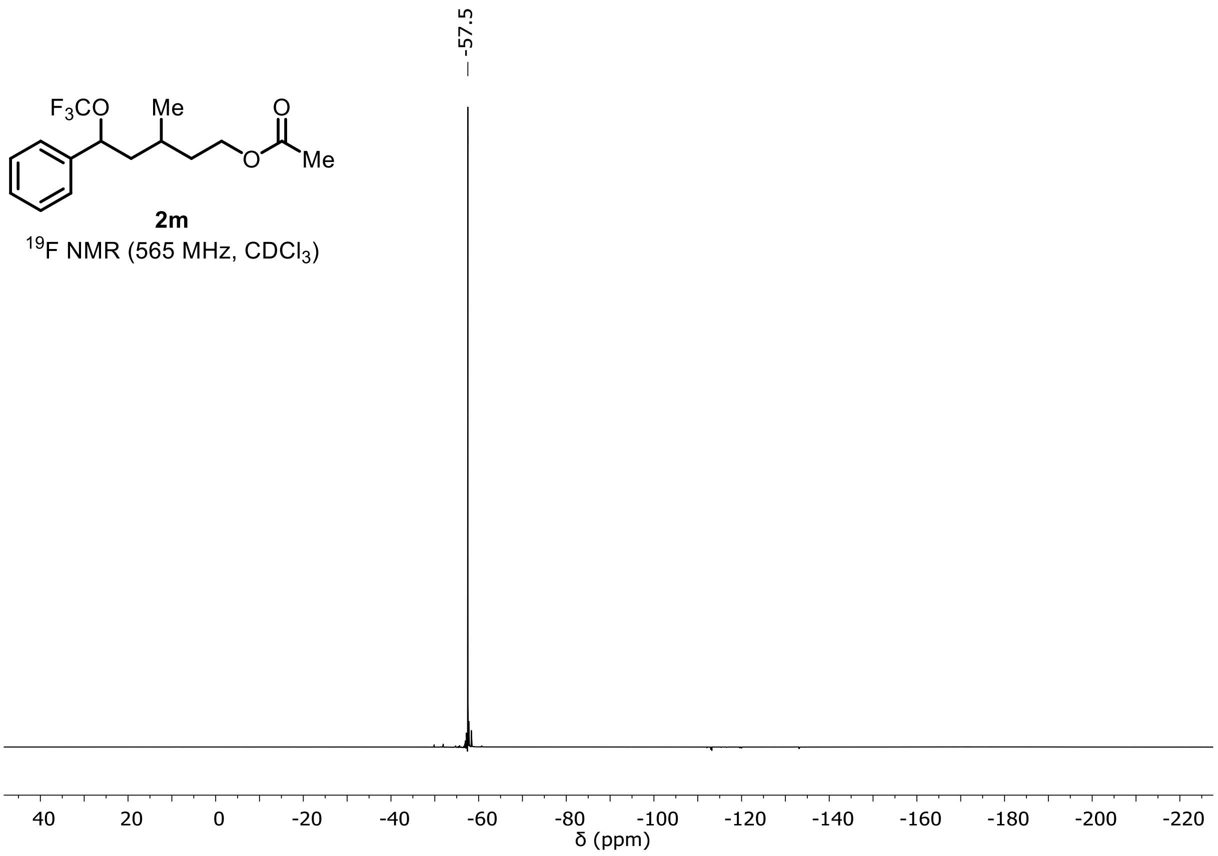

F3CO
Me
O
O
Me
2m
19F NMR (565 MHz, CDCl3)
-57.5
40
20
0
-20
-40
-60
-80
-100
-120
-140
-160
-180
-200
-220
δ (ppm)

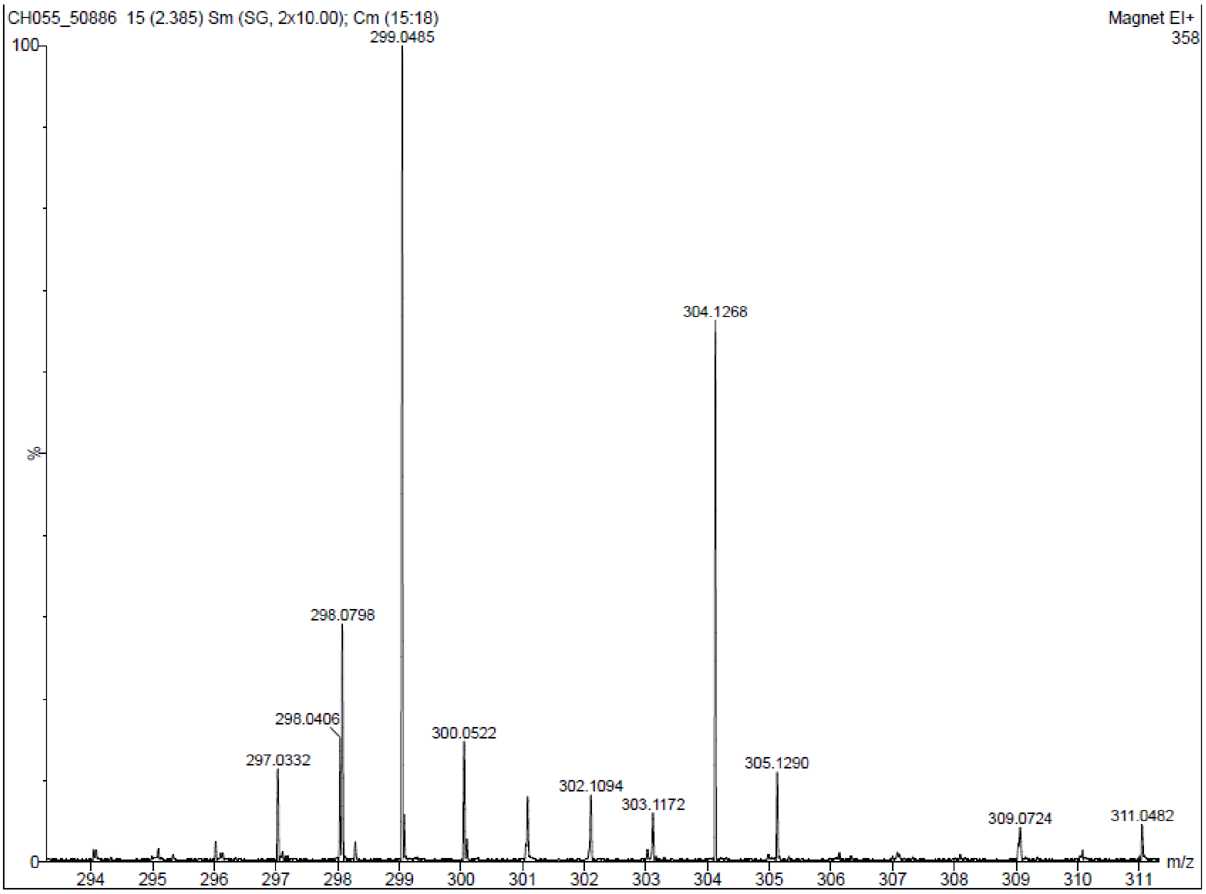

CH055_50886 15 (2.385) Sm (SG, 2x10.00); Cm (15:18)
Magnet EI+
358
100
%
0
299.0485
304.1268
298.0798
298.0406
300.0522
297.0332
305.1290
302.1094
303.1172
309.0724
311.0482
m/z
294
295
296
297
298
299
300
301
302
303
304
305
306
307
308
309
310
311

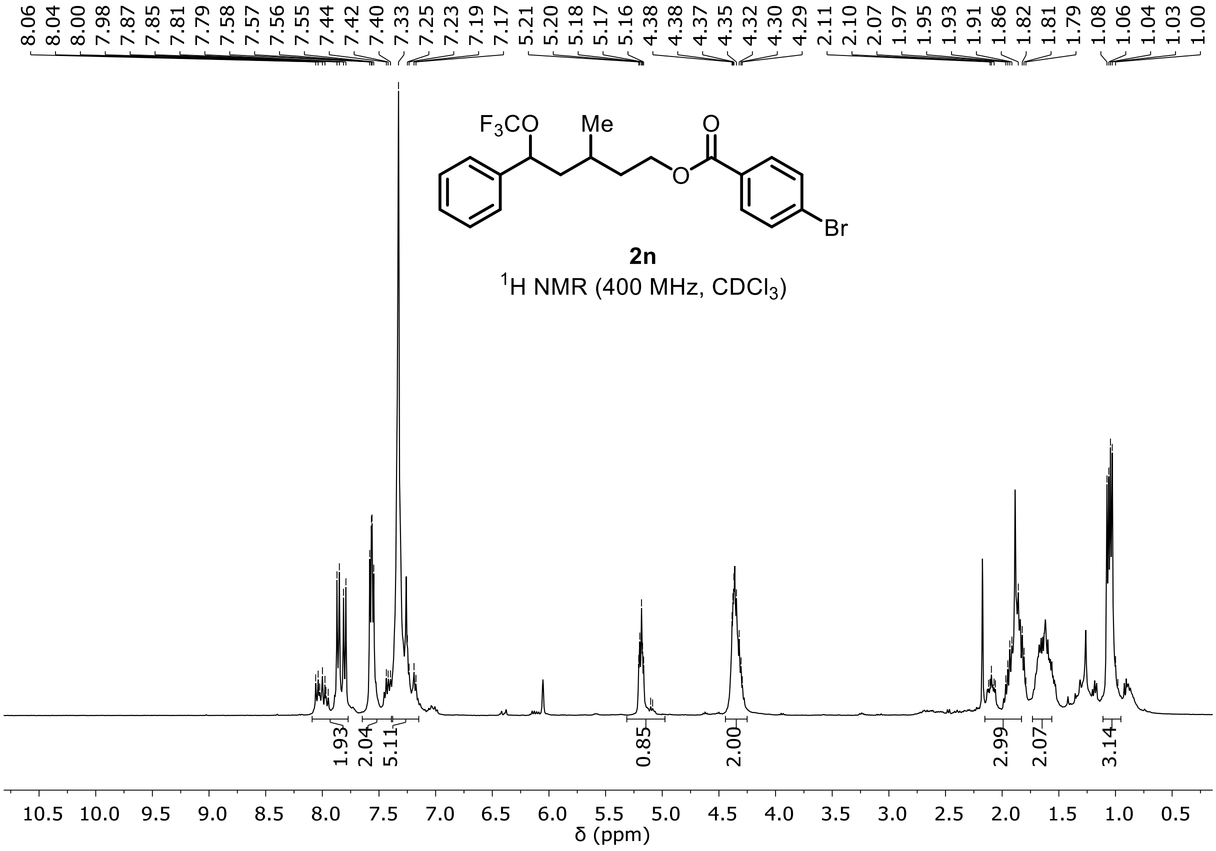
8.06 8.04 8.00 7.98 7.87 7.85 7.81 7.79 7.58 7.57 7.56 7.55 7.44 7.42 7.40 7.33 7.25 7.23 7.19 7.17 5.21 5.20 5.18 5.17 5.16 4.38 4.38 4.37 4.35 4.32 4.30 4.29 2.11 2.10 2.07 1.97 1.95 1.93 1.91 1.86 1.82 1.81 1.79 1.08 1.06 1.04 1.03 1.00
F3CO
Me
O
O
Br
2n
1H NMR (400 MHz, CDCl3)
1.93 2.04 5.11 0.85 2.00 2.99 2.07 3.14
10.5 10.0 9.5 9.0 8.5 8.0 7.5 7.0 6.5 6.0 5.5 5.0 4.5 4.0 3.5 3.0 2.5 2.0 1.5 1.0 0.5
δ (ppm)

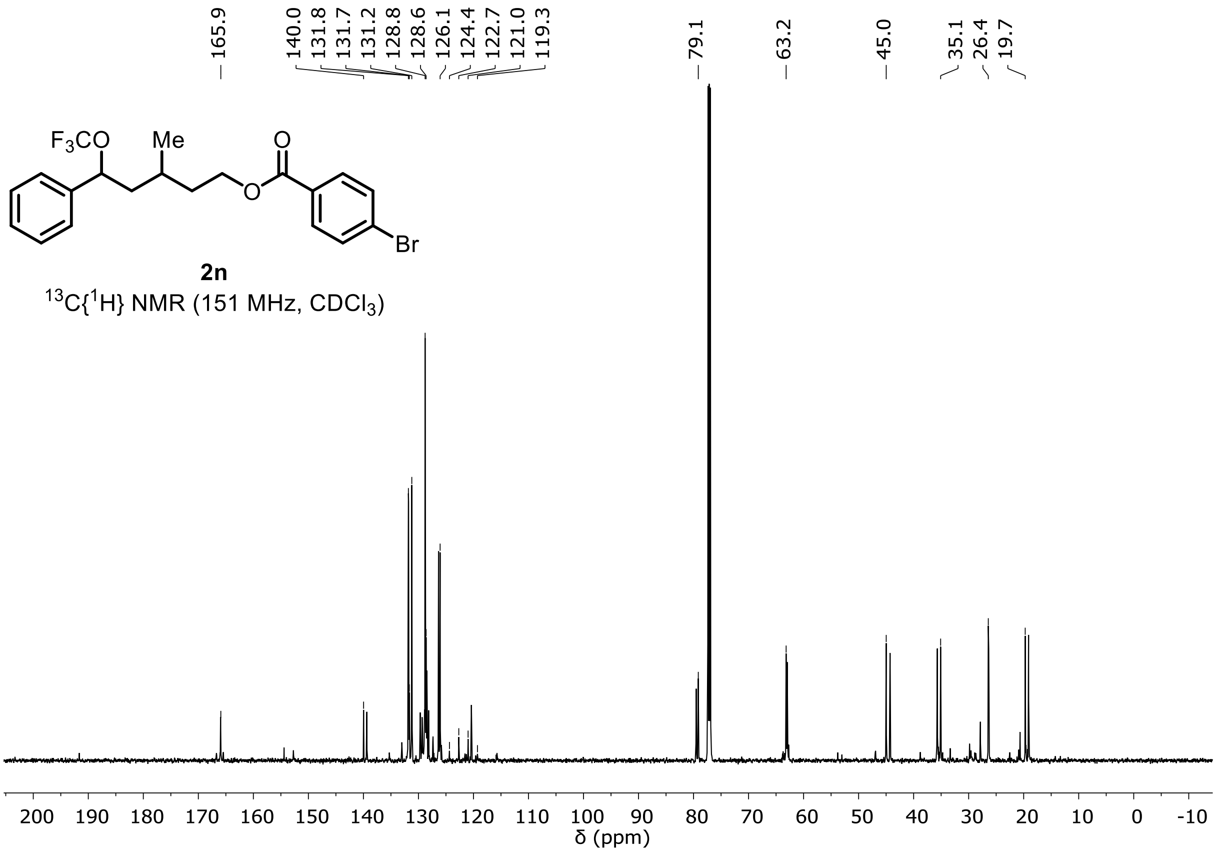
165.9
140.0 131.8 131.7 131.2 128.8 128.6 126.1 124.4 122.7 121.0 119.3
79.1
63.2
45.0
35.1 26.4 19.7
F3CO
Me
O
O
Br
2n
13C{1H} NMR (151 MHz, CDCl3)
200 190 180 170 160 150 140 130 120 110 100 90 80 70 60 50 40 30 20 10 0 -10
δ (ppm)

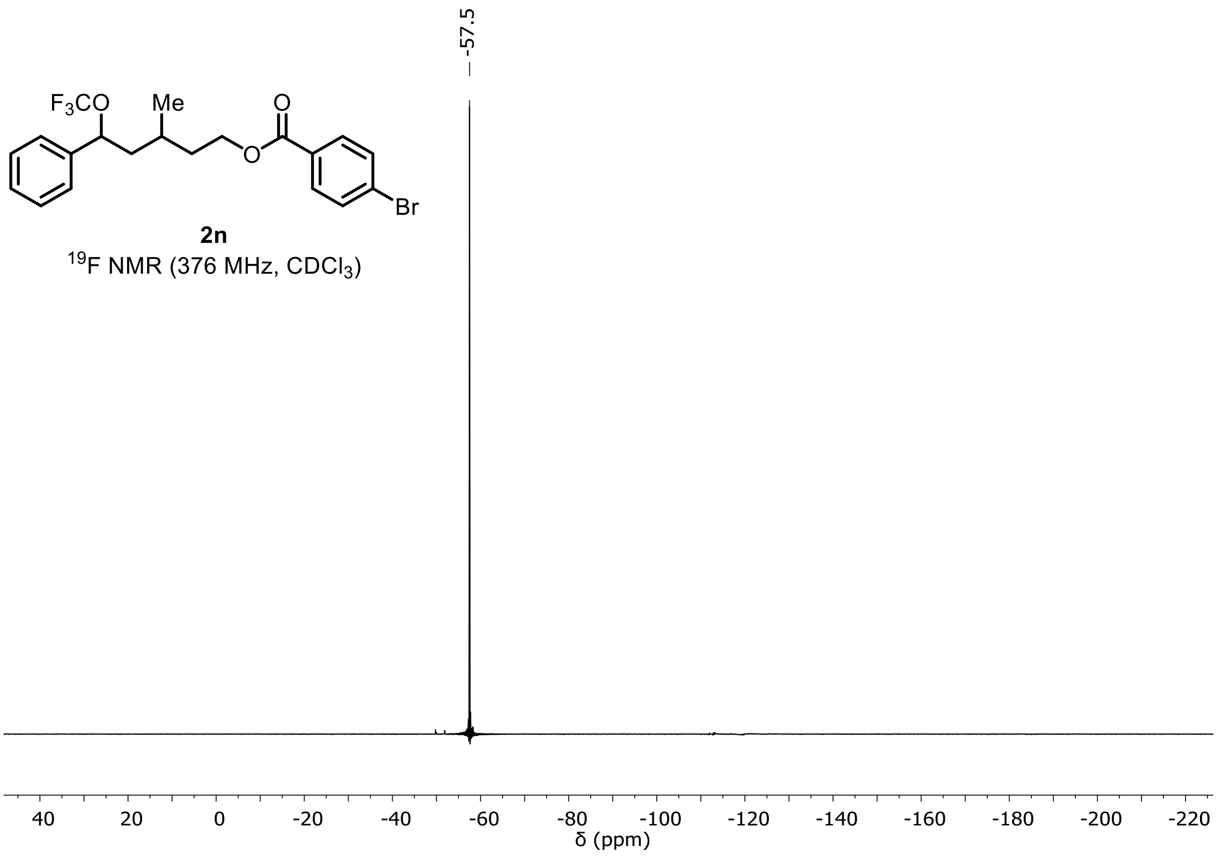
F3CO
Me
O
O
Br
2n
19F NMR (376 MHz, CDCl3)
-57.5
40 20 0 -20 -40 -60 -80 -100 -120 -140 -160 -180 -200 -220
δ (ppm)

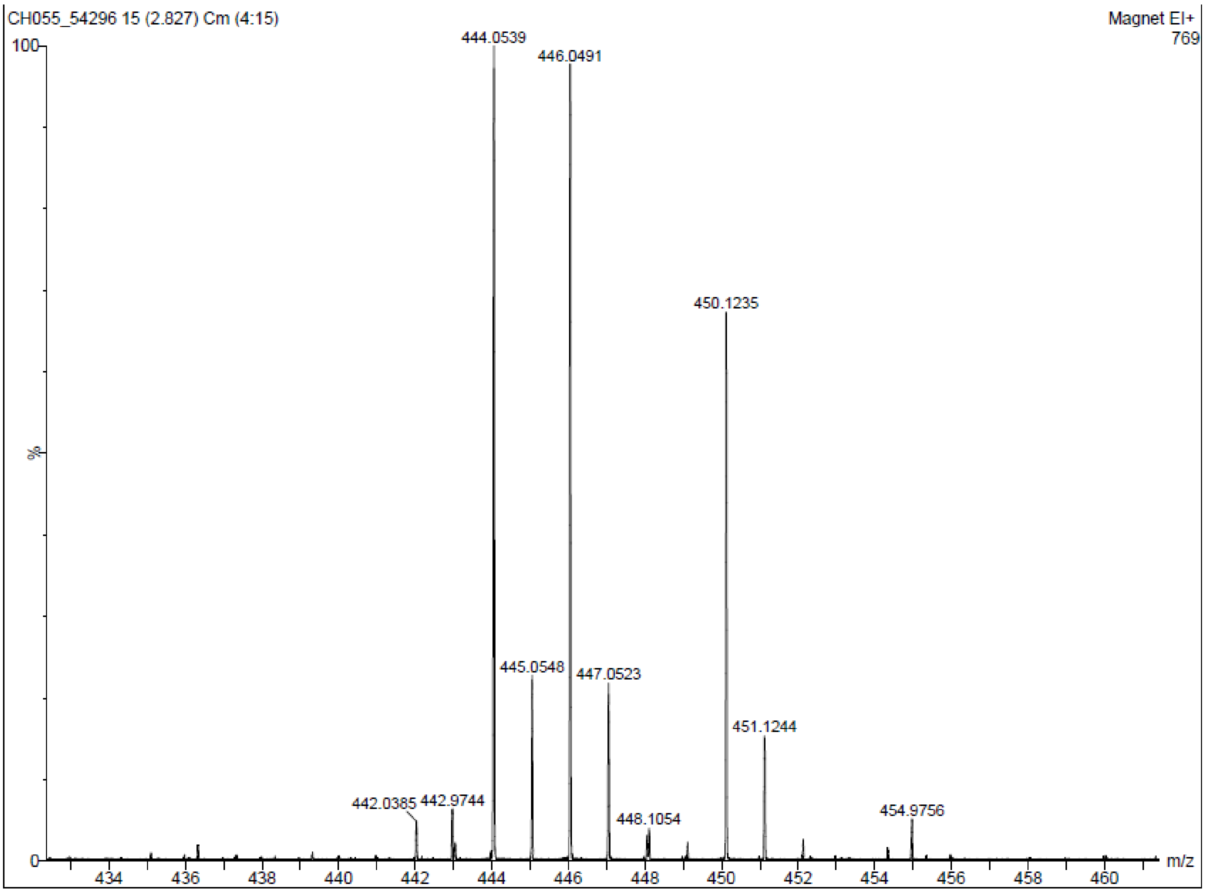
CH055_54296 15 (2.827) Cm (4:15)
Magnet EI+
769
100
%
0
444.0539
446.0491
450.1235
445.0548
447.0523
451.1244
442.0385
442.9744
448.1054
454.9756
434 436 438 440 442 444 446 448 450 452 454 456 458 460
m/z

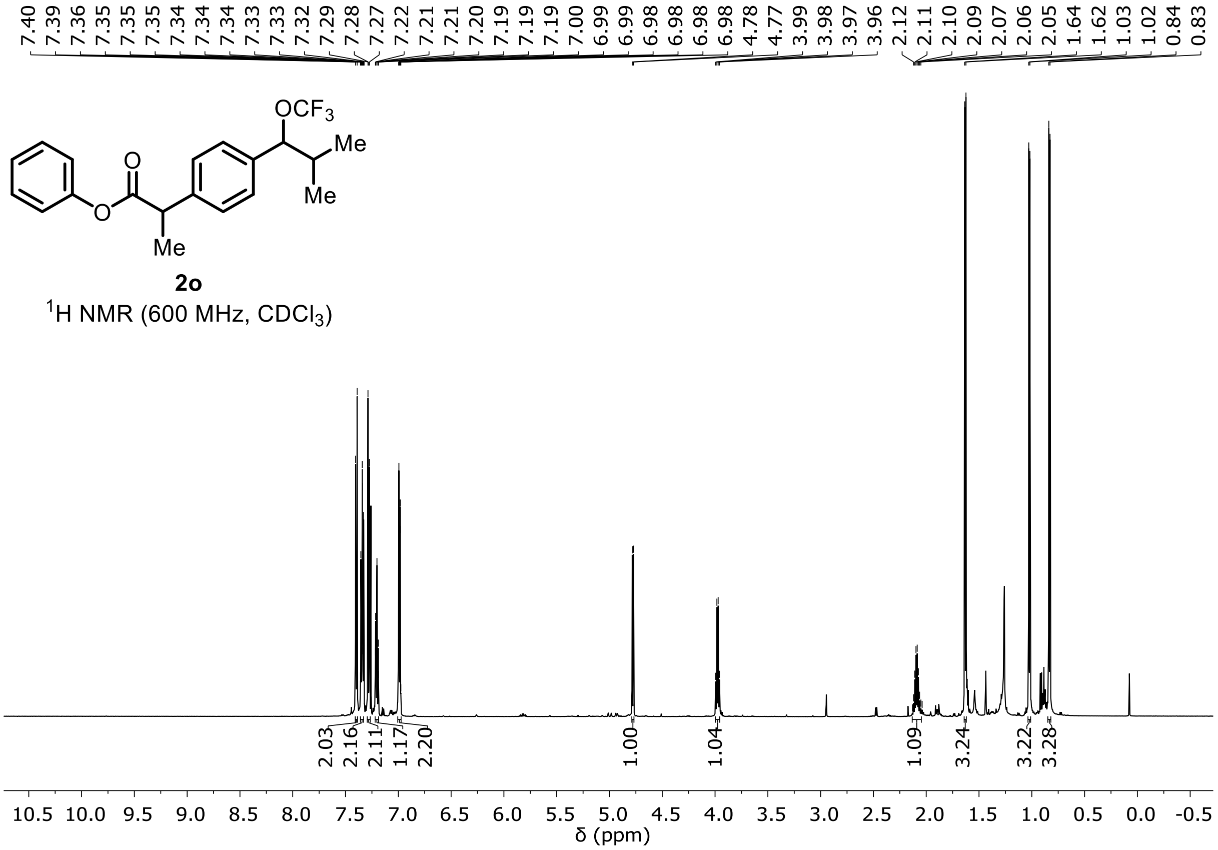
OCF3
Me
O
O
Me
Me
2o
1H NMR (600 MHz, CDCl3)
7.40 7.39 7.36 7.35 7.35 7.35 7.34 7.34 7.34 7.33 7.33 7.32 7.29 7.28 7.27 7.22 7.21 7.21 7.20 7.19 7.19 7.19 7.00 6.99 6.99 6.98 6.98 6.98 6.98 4.78 4.77 3.99 3.98 3.97 3.96 2.12 2.11 2.10 2.09 2.07 2.06 2.05 1.64 1.62 1.03 1.02 0.84 0.83
2.03 2.16 2.11 1.17 2.20 1.00 1.04 1.09 3.24 3.22 3.28
10.5 10.0 9.5 9.0 8.5 8.0 7.5 7.0 6.5 6.0 5.5 5.0 4.5 4.0 3.5 3.0 2.5 2.0 1.5 1.0 0.5 0.0 -0.5
δ (ppm)

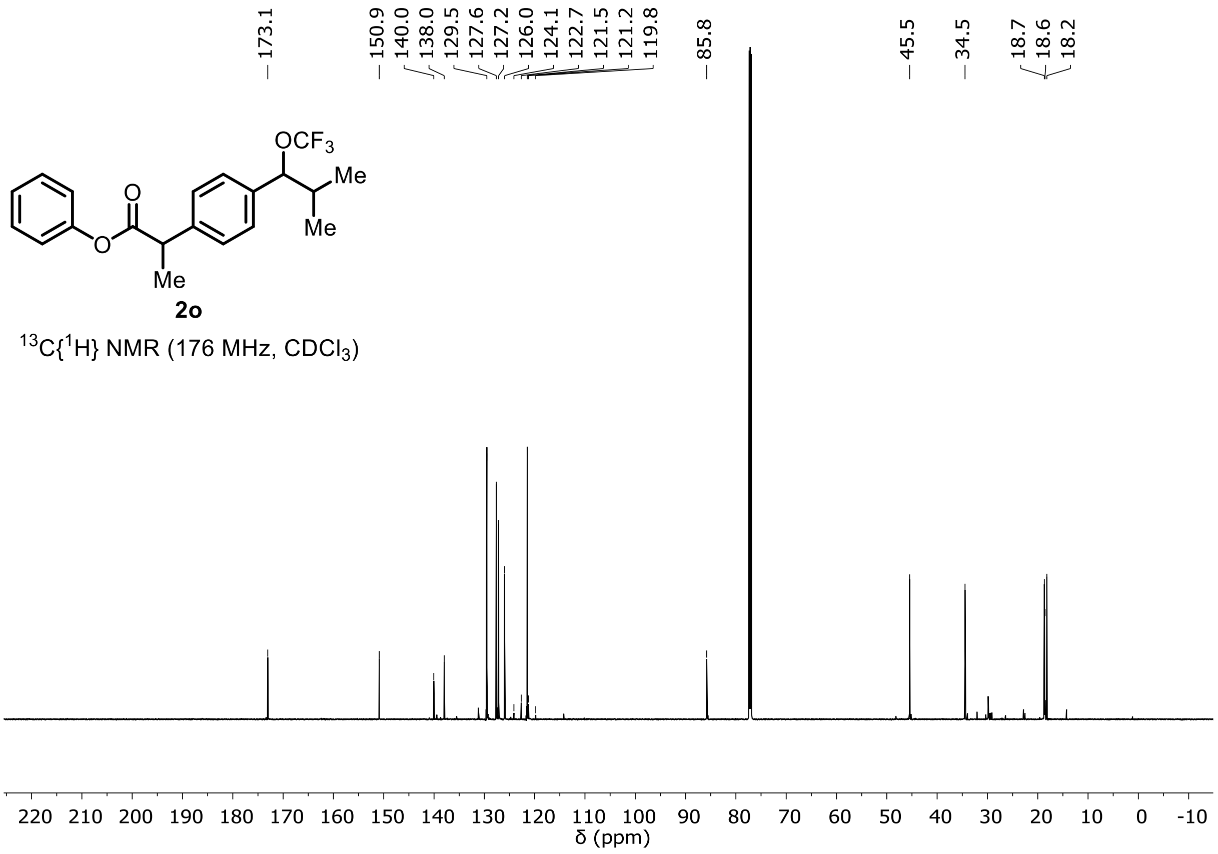
OCF3
Me
O
O
Me
Me
2o
13C{1H} NMR (176 MHz, CDCl3)
173.1 150.9 140.0 138.0 129.5 127.6 127.2 126.0 124.1 122.7 121.5 121.2 119.8 85.8 45.5 34.5 18.7 18.6 18.2
220 210 200 190 180 170 160 150 140 130 120 110 100 90 80 70 60 50 40 30 20 10 0 -10
δ (ppm)

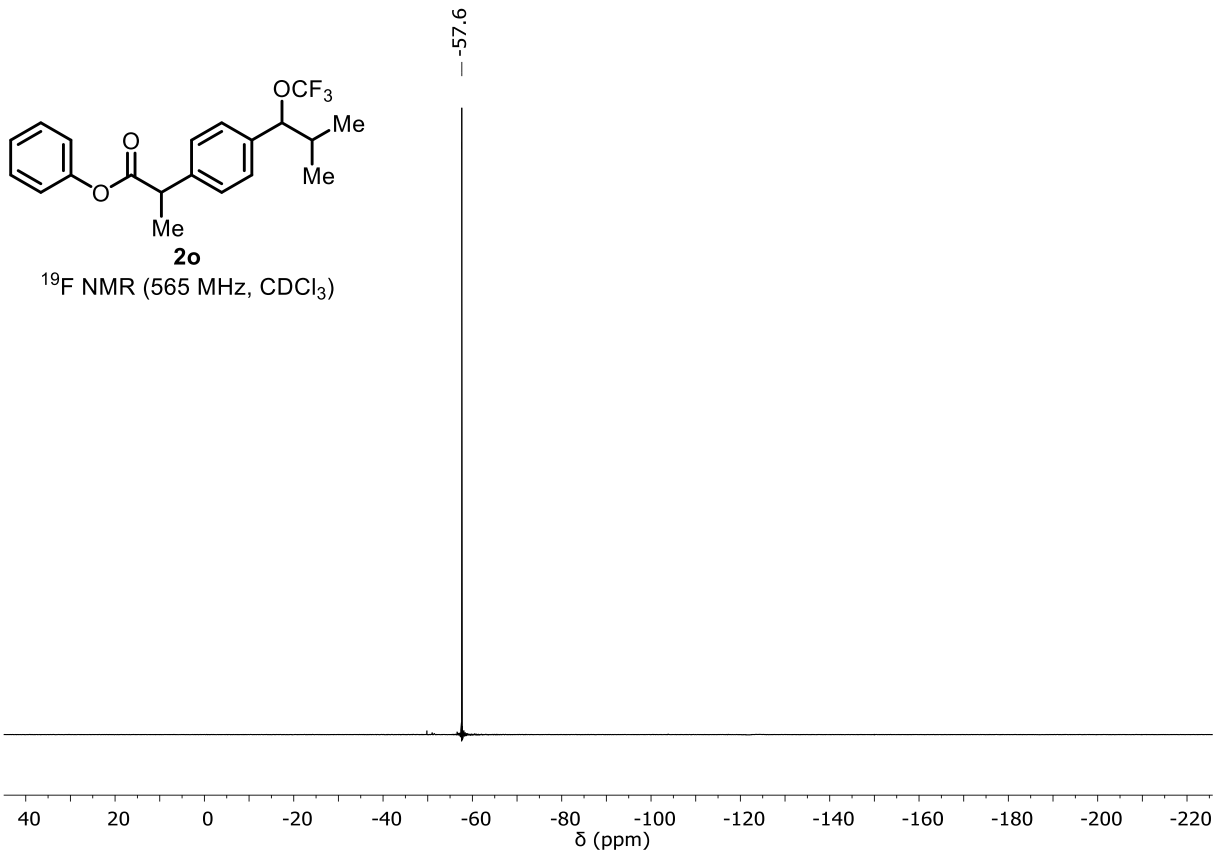

OCF3
Me
Me
O
O
Me
2o
19F NMR (565 MHz, CDCl3)
-57.6
40
20
0
-20
-40
-60
-80
-100
-120
-140
-160
-180
-200
-220
δ (ppm)

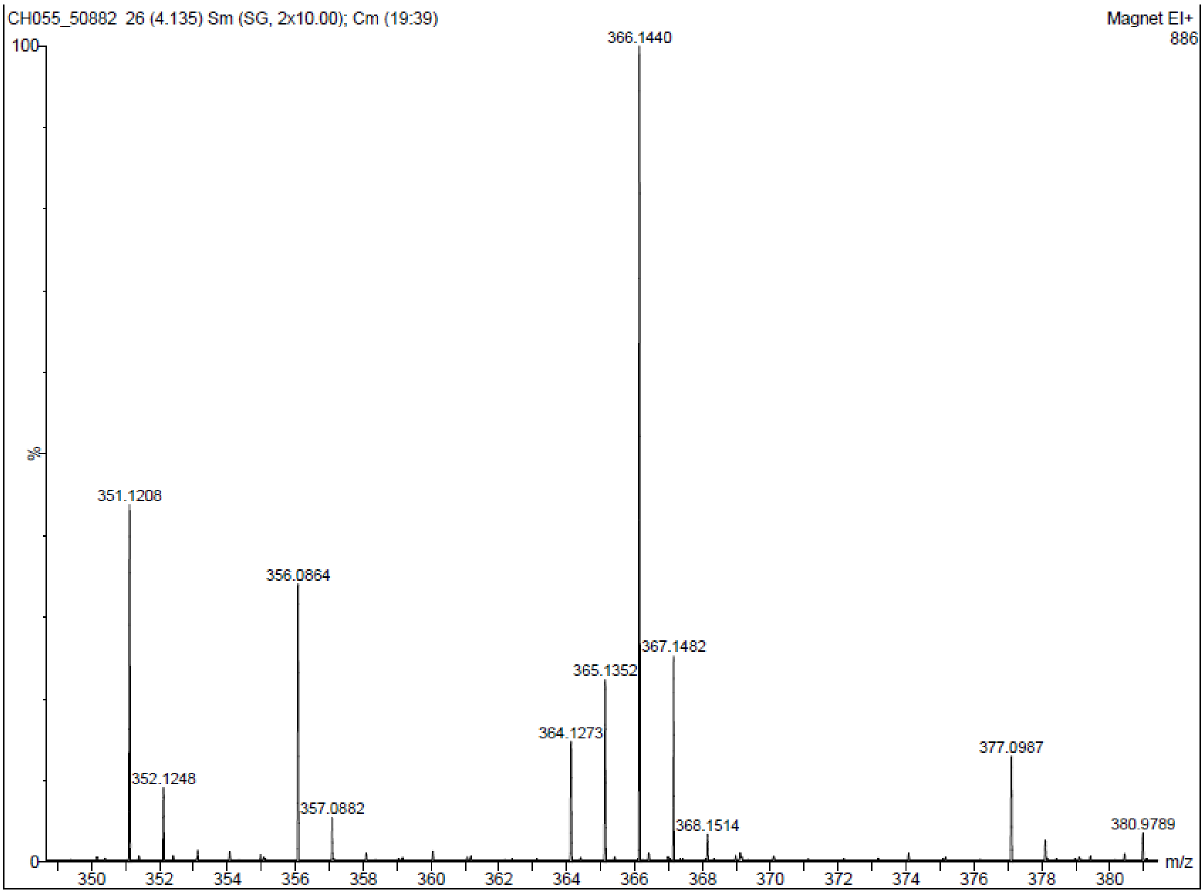

CH055_50882 26 (4.135) Sm (SG, 2x10.00); Cm (19:39)
Magnet EI+
886
100
%
0
366.1440
351.1208
356.0864
367.1482
365.1352
364.1273
377.0987
352.1248
357.0882
368.1514
380.9789
350
352
354
356
358
360
362
364
366
368
370
372
374
376
378
380
m/z

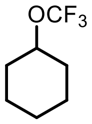

4a

^{19}F NMR (376 MHz, $CDCl_3$)

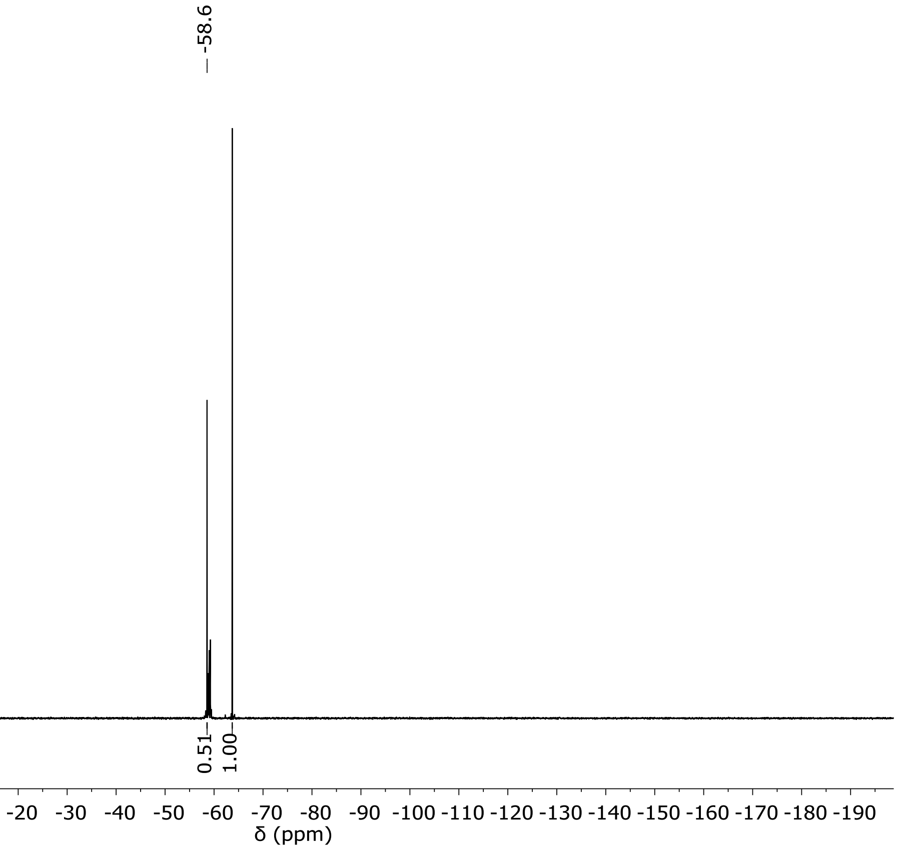

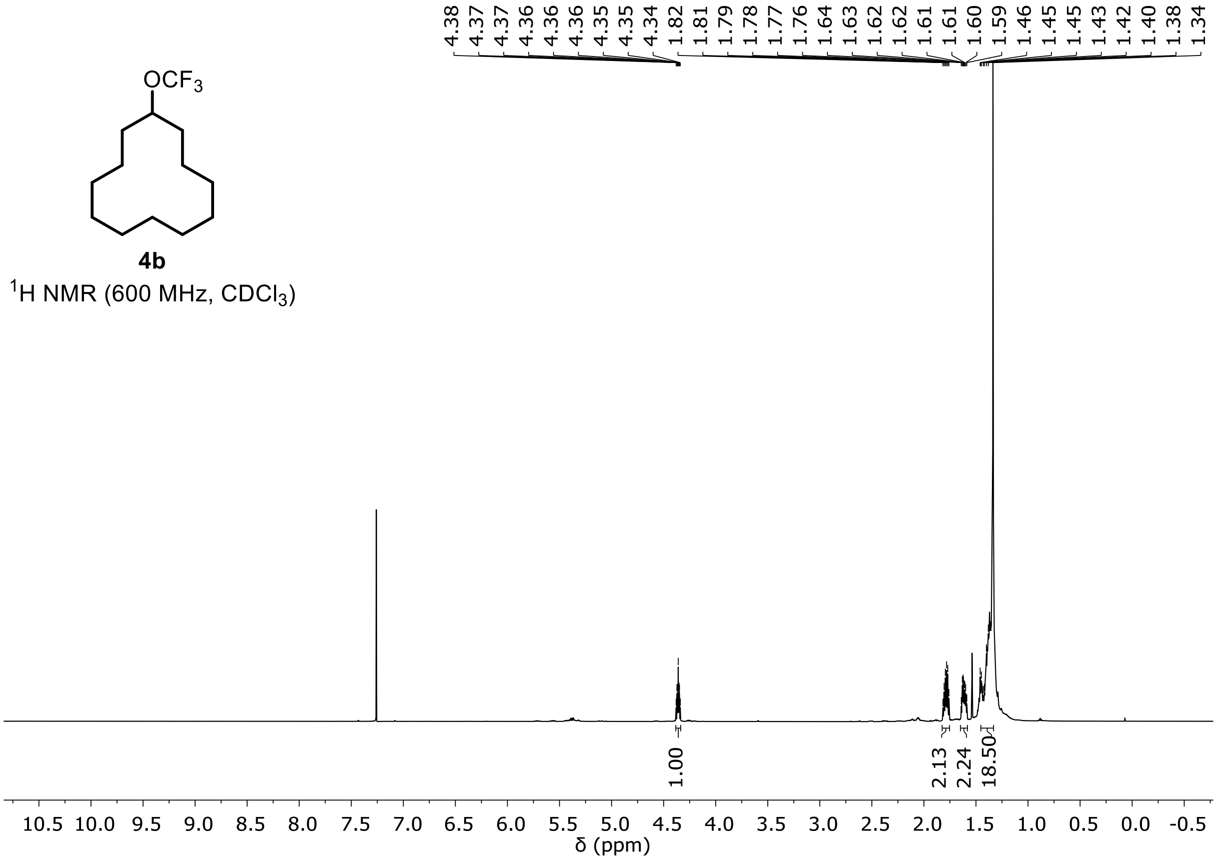
OCF3
4b
1H NMR (600 MHz, CDCl3)
4.38 4.37 4.37 4.36 4.36 4.35 4.35 4.34 1.82 1.81 1.79 1.78 1.77 1.76 1.64 1.63 1.62 1.62 1.61 1.61 1.60 1.59 1.46 1.45 1.45 1.43 1.42 1.40 1.38 1.34
1.00
2.13 2.24 18.50
10.5 10.0 9.5 9.0 8.5 8.0 7.5 7.0 6.5 6.0 5.5 5.0 4.5 4.0 3.5 3.0 2.5 2.0 1.5 1.0 0.5 0.0 -0.5
δ (ppm)

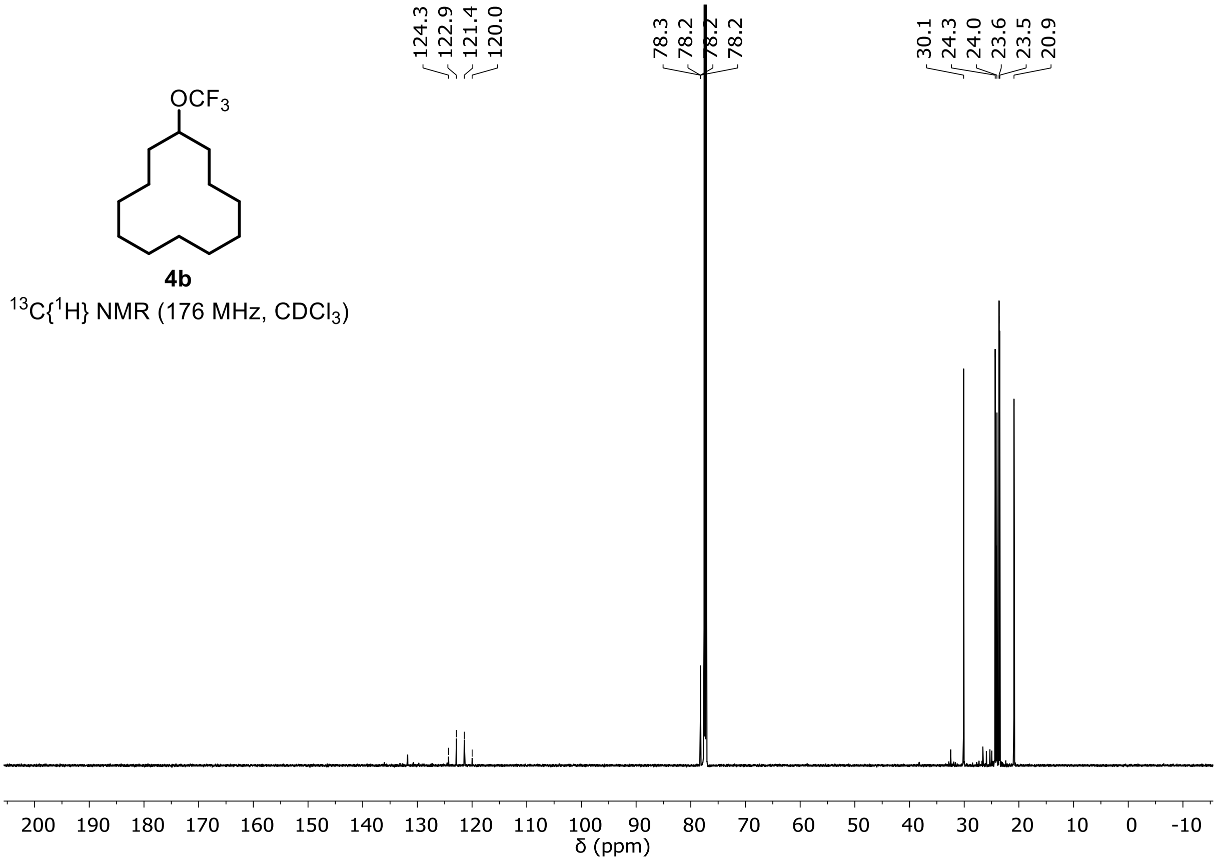
124.3 122.9 121.4 120.0
78.3 78.2 78.2 78.2
30.1 24.3 24.0 23.6 23.5 20.9
OCF3
4b
13C{1H} NMR (176 MHz, CDCl3)
200 190 180 170 160 150 140 130 120 110 100 90 80 70 60 50 40 30 20 10 0 -10
δ (ppm)

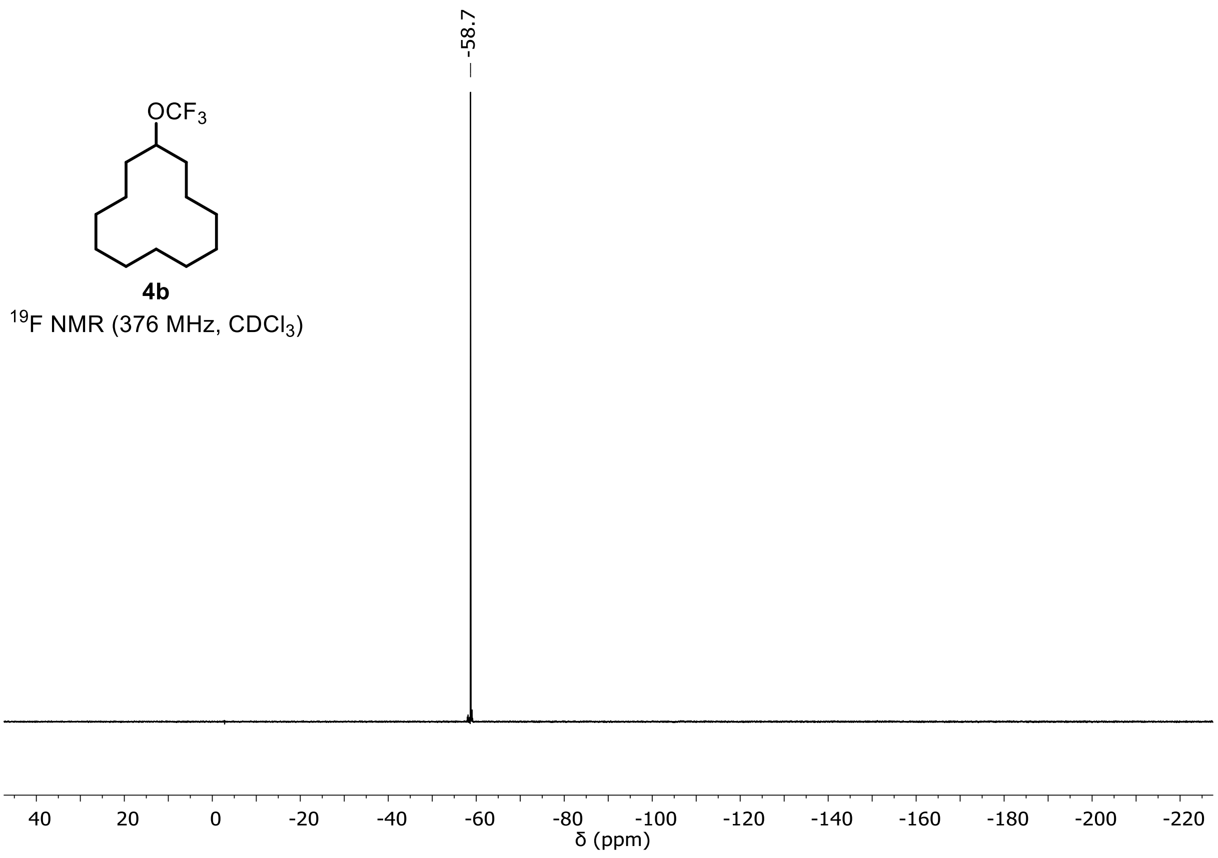
OCF$_3$
4b
^{19}F NMR (376 MHz, CDCl$_3$)
-58.7
40 20 0 -20 -40 -60 -80 -100 -120 -140 -160 -180 -200 -220
δ (ppm)

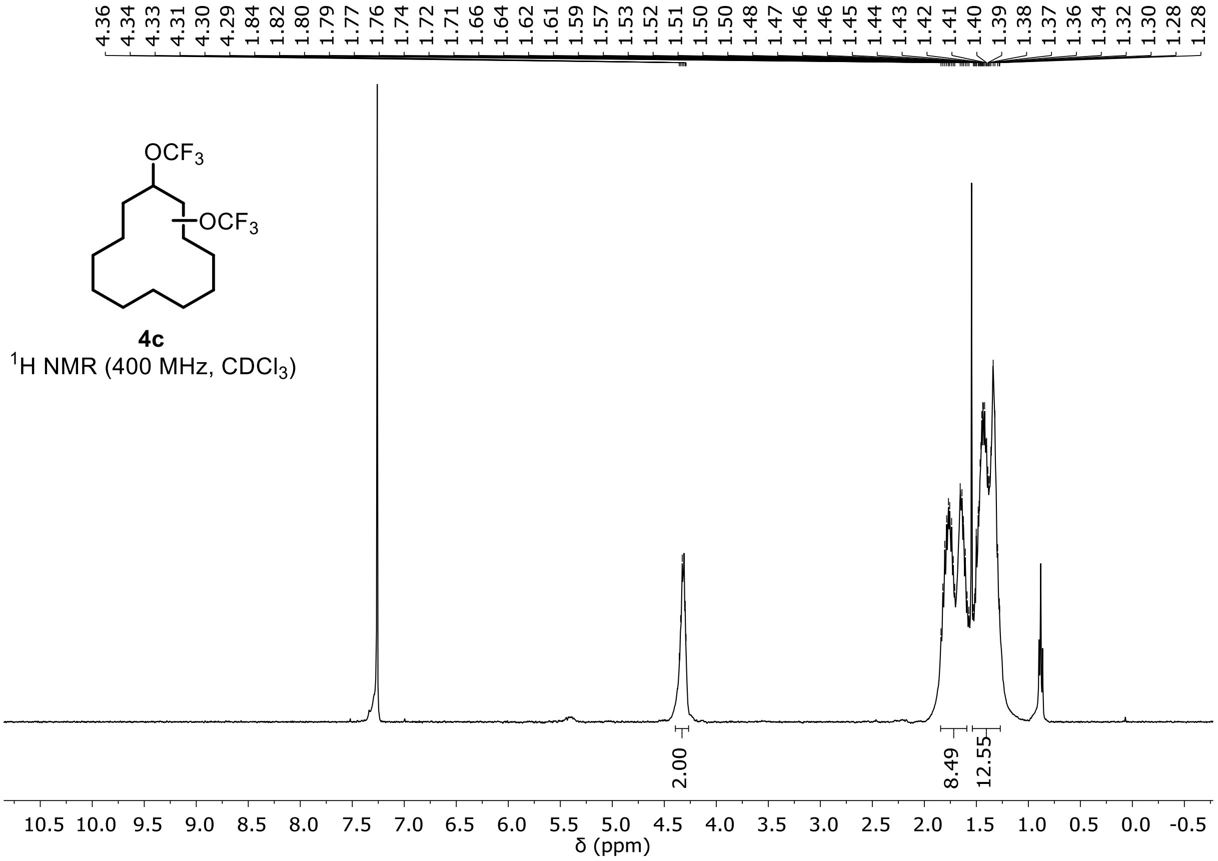
4.36 4.34 4.33 4.31 4.30 4.29 1.84 1.82 1.80 1.79 1.77 1.76 1.74 1.72 1.71 1.66 1.64 1.62 1.61 1.59 1.57 1.53 1.52 1.51 1.50 1.50 1.48 1.47 1.46 1.46 1.45 1.44 1.43 1.42 1.41 1.40 1.39 1.38 1.37 1.36 1.34 1.32 1.30 1.28 1.28
OCF3
OCF3
4c
1H NMR (400 MHz, CDCl3)
2.00
8.49
12.55
10.5 10.0 9.5 9.0 8.5 8.0 7.5 7.0 6.5 6.0 5.5 5.0 4.5 4.0 3.5 3.0 2.5 2.0 1.5 1.0 0.5 0.0 -0.5
δ (ppm)

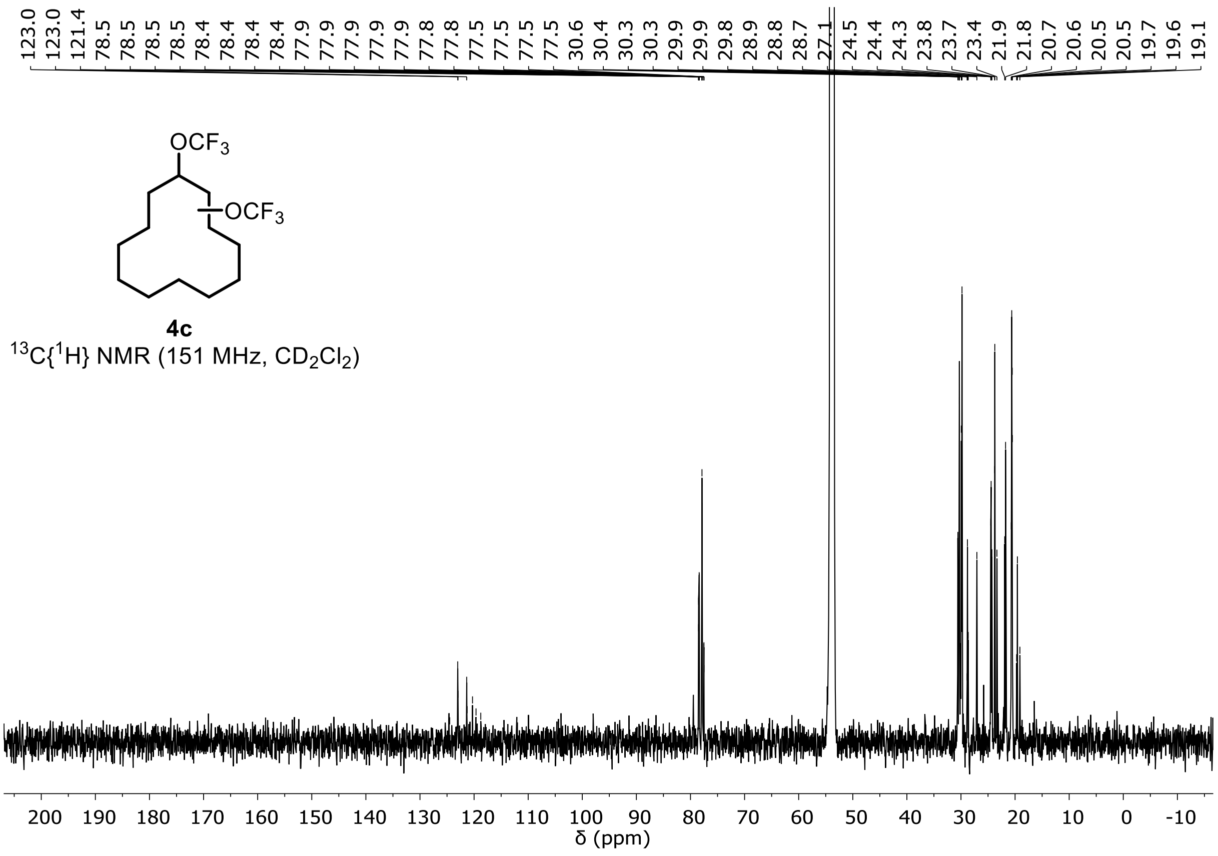
123.0 123.0 121.4 78.5 78.5 78.5 78.5 78.4 78.4 78.4 78.4 77.9 77.9 77.9 77.9 77.9 77.8 77.8 77.5 77.5 77.5 77.5 30.6 30.4 30.3 30.3 29.9 29.9 29.8 28.9 28.8 28.7 27.1 24.5 24.4 24.3 23.8 23.7 23.4 21.9 21.8 20.7 20.6 20.5 20.5 19.7 19.6 19.1
OCF3
OCF3
4c
13C{1H} NMR (151 MHz, CD2Cl2)
200 190 180 170 160 150 140 130 120 110 100 90 80 70 60 50 40 30 20 10 0 -10
δ (ppm)

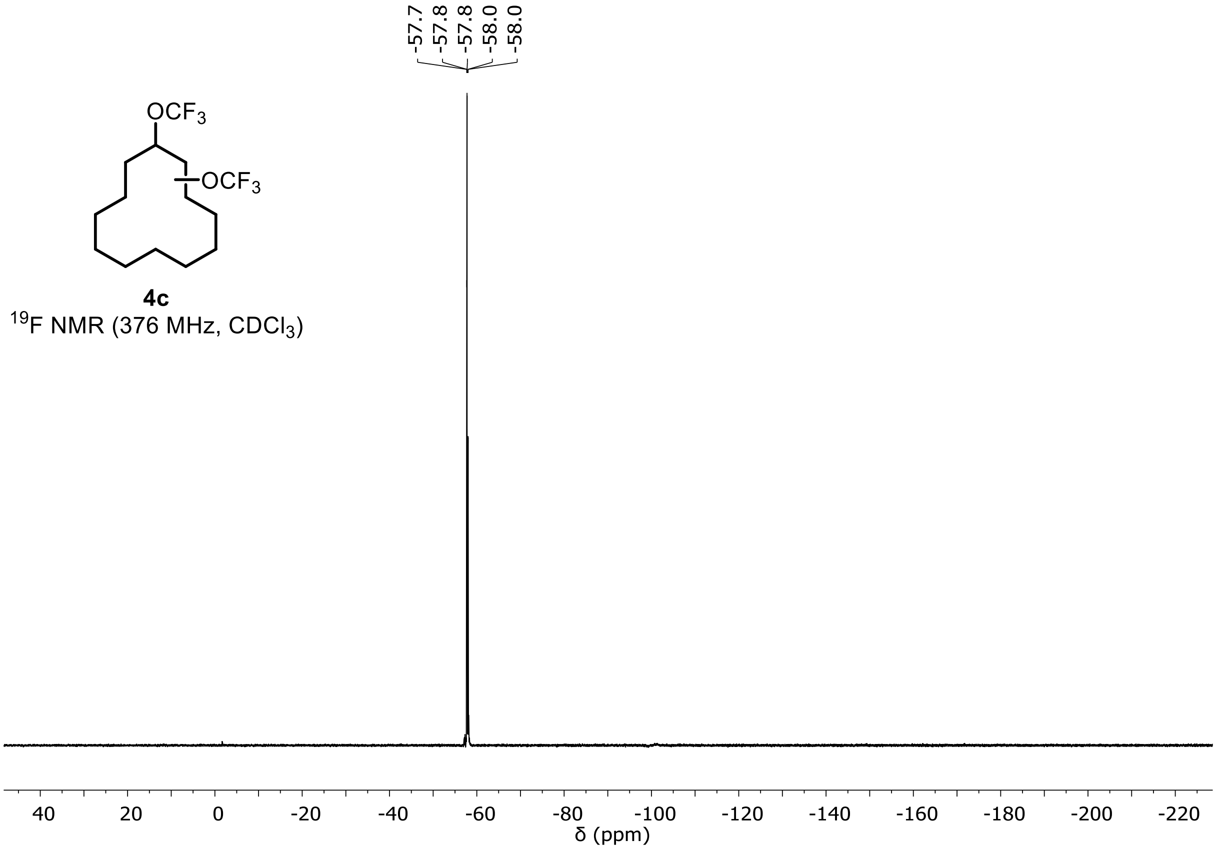

OCF3
OCF3
4c
19F NMR (376 MHz, CDCl3)
-57.7
-57.8
-57.8
-58.0
-58.0
40 20 0 -20 -40 -60 -80 -100 -120 -140 -160 -180 -200 -220
δ (ppm)

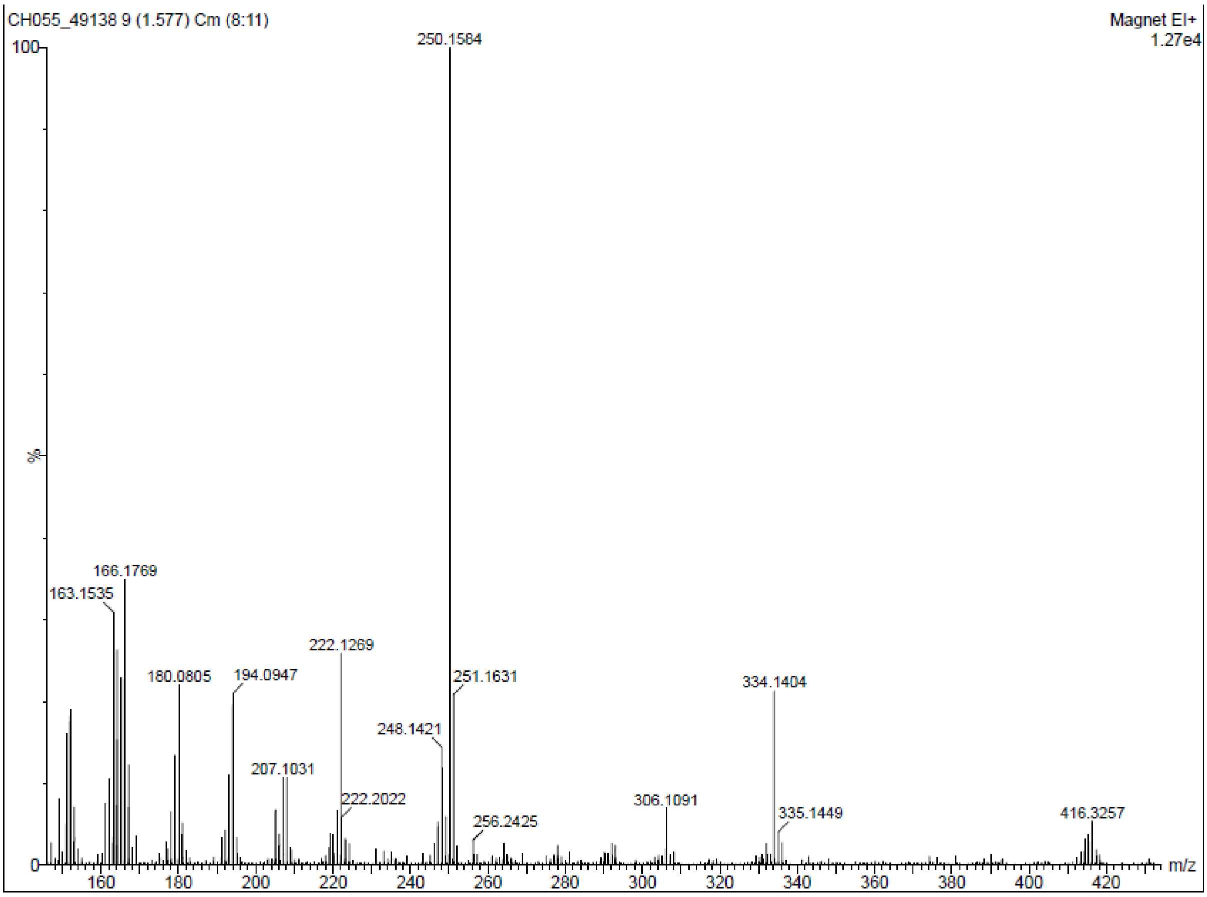

CH055_49138 9 (1.577) Cm (8:11)
Magnet EI+
1.27e4
100
%
0
250.1584
166.1769
163.1535
180.0805
194.0947
222.1269
251.1631
334.1404
248.1421
207.1031
222.2022
306.1091
256.2425
335.1449
416.3257
160 180 200 220 240 260 280 300 320 340 360 380 400 420
m/z

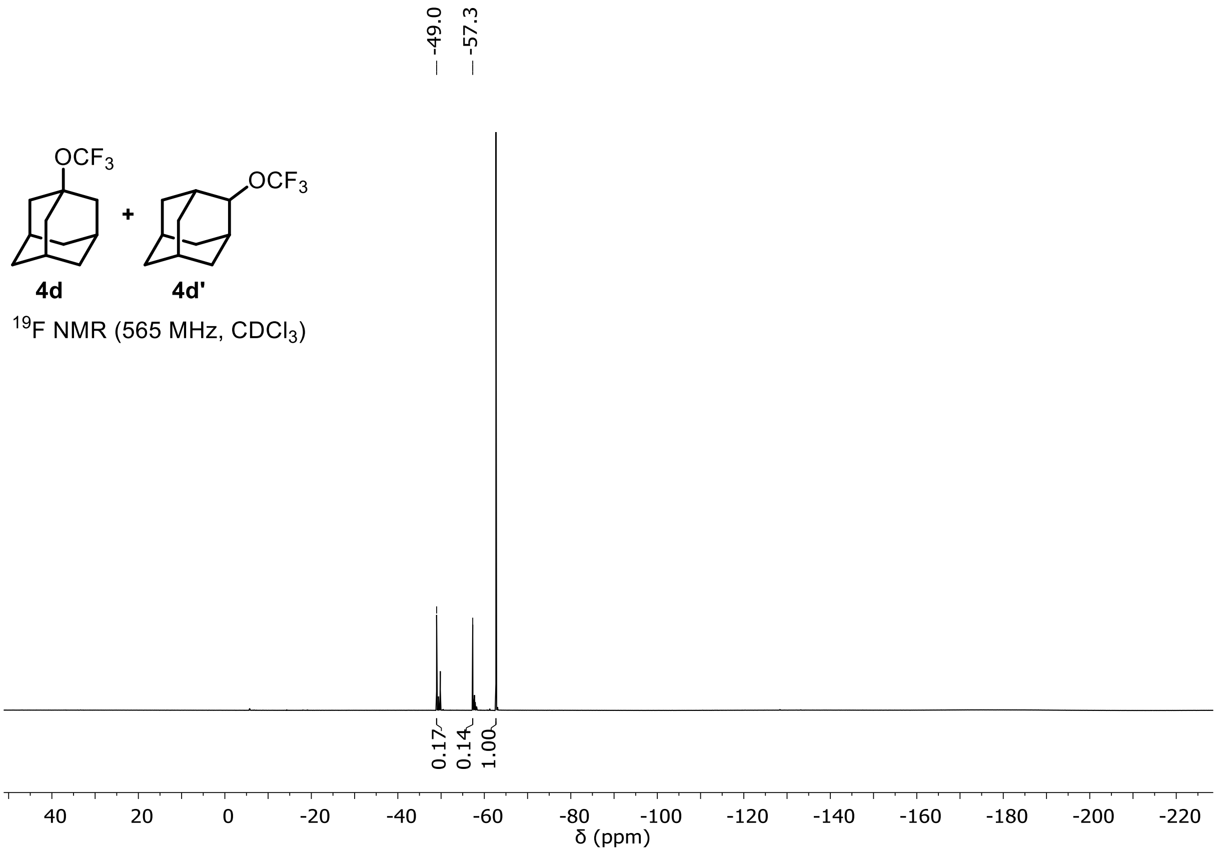

-49.0
-57.3
OCF3
OCF3
4d
4d'
19F NMR (565 MHz, CDCl3)
0.17
0.14
1.00
40 20 0 -20 -40 -60 -80 -100 -120 -140 -160 -180 -200 -220
δ (ppm)

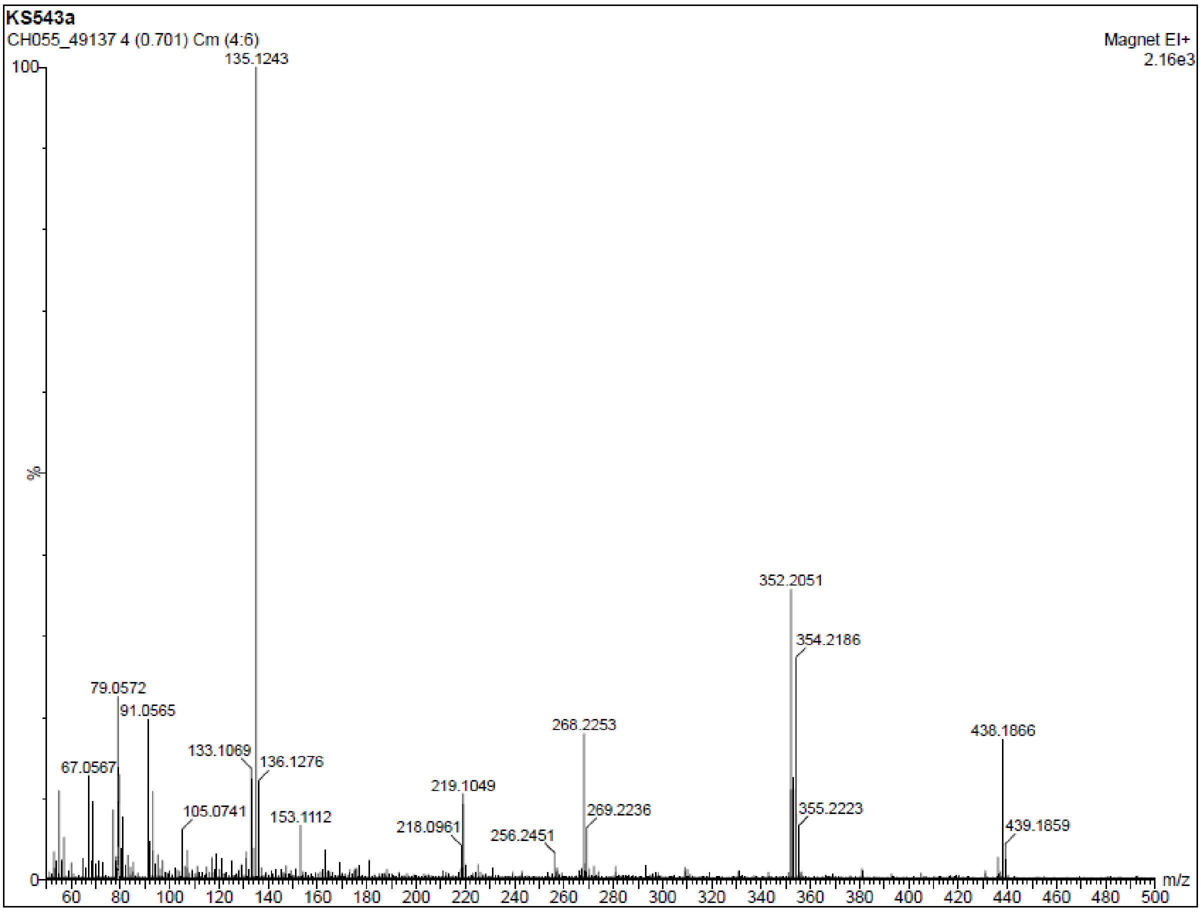

KS543a
CH055_49137 4 (0.701) Cm (4:6)
Magnet EI+
2.16e3
100
%
0
135.1243
352.2051
354.2186
79.0572
91.0565
268.2253
438.1866
67.0567
133.1069
136.1276
219.1049
105.0741
153.1112
269.2236
355.2223
218.0961
256.2451
439.1859
60 80 100 120 140 160 180 200 220 240 260 280 300 320 340 360 380 400 420 440 460 480 500
m/z

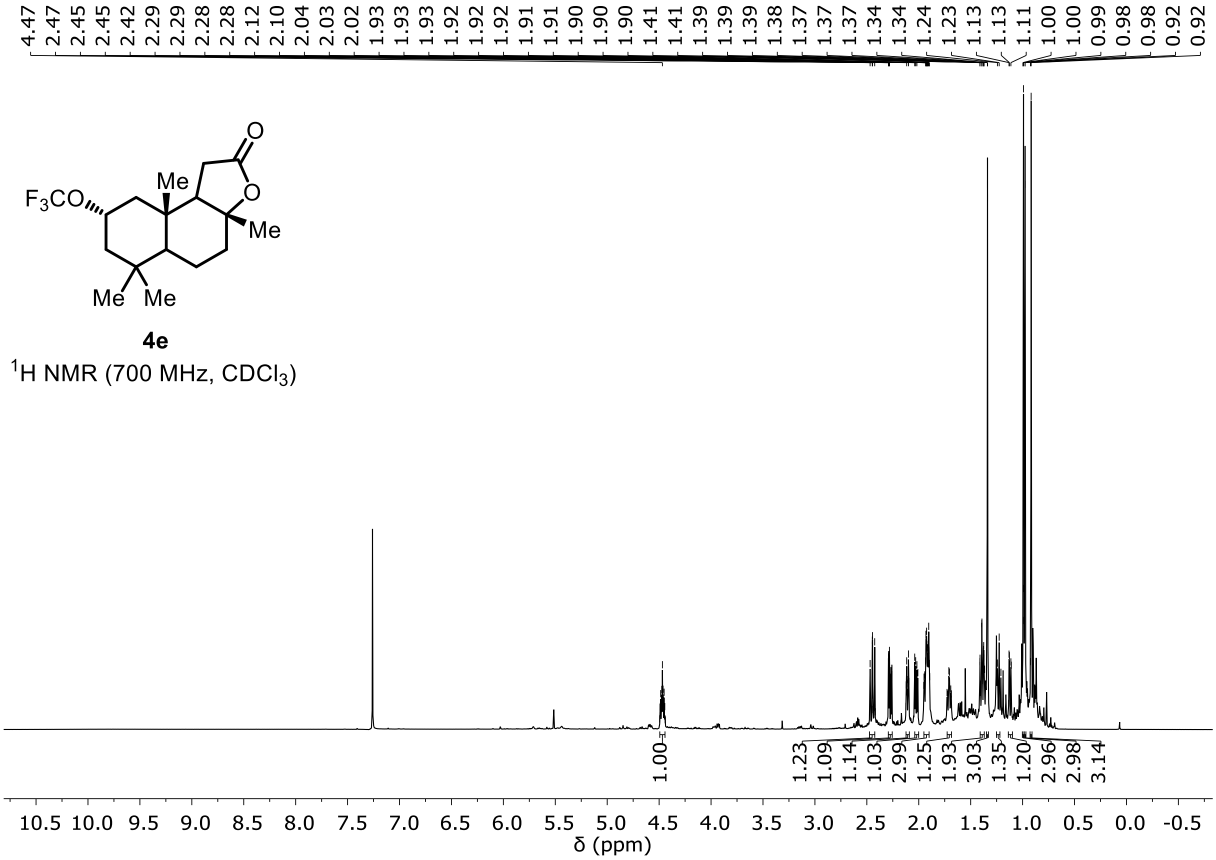
4.47 2.47 2.45 2.45 2.42 2.29 2.29 2.28 2.28 2.12 2.10 2.04 2.03 2.02 1.93 1.93 1.93 1.92 1.92 1.92 1.91 1.91 1.90 1.90 1.90 1.41 1.41 1.39 1.39 1.39 1.38 1.37 1.37 1.37 1.34 1.34 1.24 1.23 1.13 1.13 1.11 1.00 1.00 0.99 0.98 0.98 0.92 0.92
O
Me
F_3CO
O
Me
Me Me
4e
^{1}H NMR (700 MHz, $CDCl_3$)
1.00
1.23 1.09 1.14 1.03 2.99 1.25 1.93 3.03 1.35 1.20 2.96 2.98 3.14
10.5 10.0 9.5 9.0 8.5 8.0 7.5 7.0 6.5 6.0 5.5 5.0 4.5 4.0 3.5 3.0 2.5 2.0 1.5 1.0 0.5 0.0 -0.5
δ (ppm)

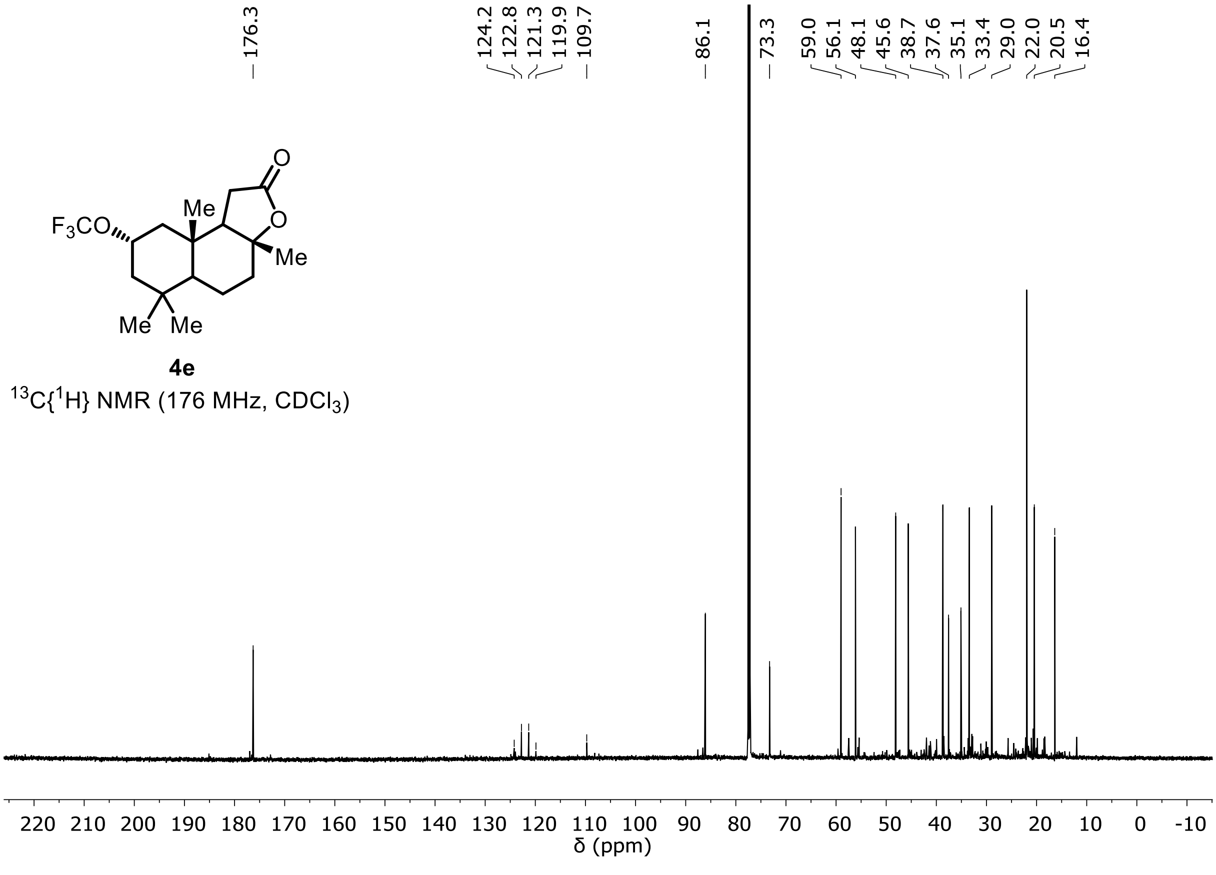
176.3
124.2 122.8 121.3 119.9 109.7
86.1
73.3
59.0 56.1 48.1 45.6 38.7 37.6 35.1 33.4 29.0 22.0 20.5 16.4
O
Me
F_3CO
O
Me
Me Me
4e
^{13}C{^{1}H} NMR (176 MHz, $CDCl_3$)
220 210 200 190 180 170 160 150 140 130 120 110 100 90 80 70 60 50 40 30 20 10 0 -10
δ (ppm)

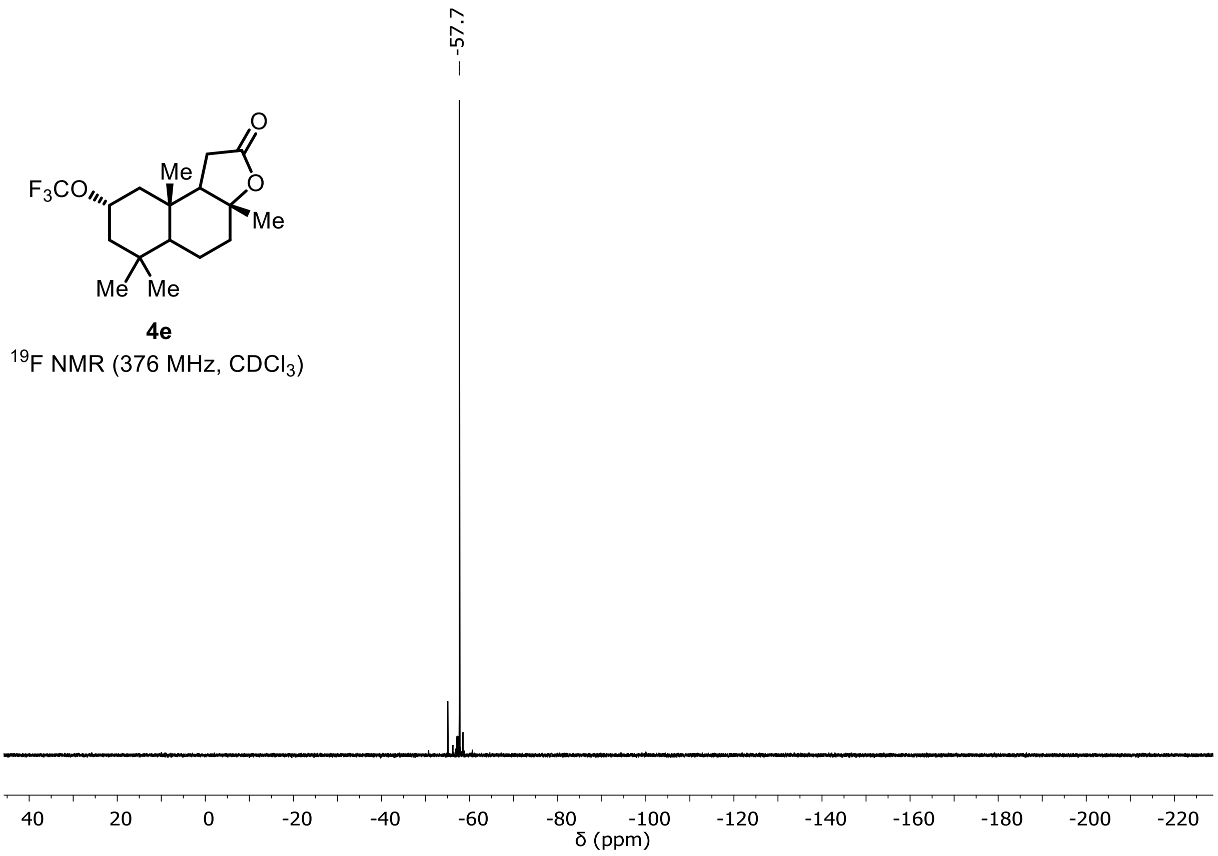
F3CO
Me
O
O
Me
Me
Me
4e
19F NMR (376 MHz, CDCl3)
-57.7
40
20
0
-20
-40
-60
-80
-100
-120
-140
-160
-180
-200
-220
δ (ppm)

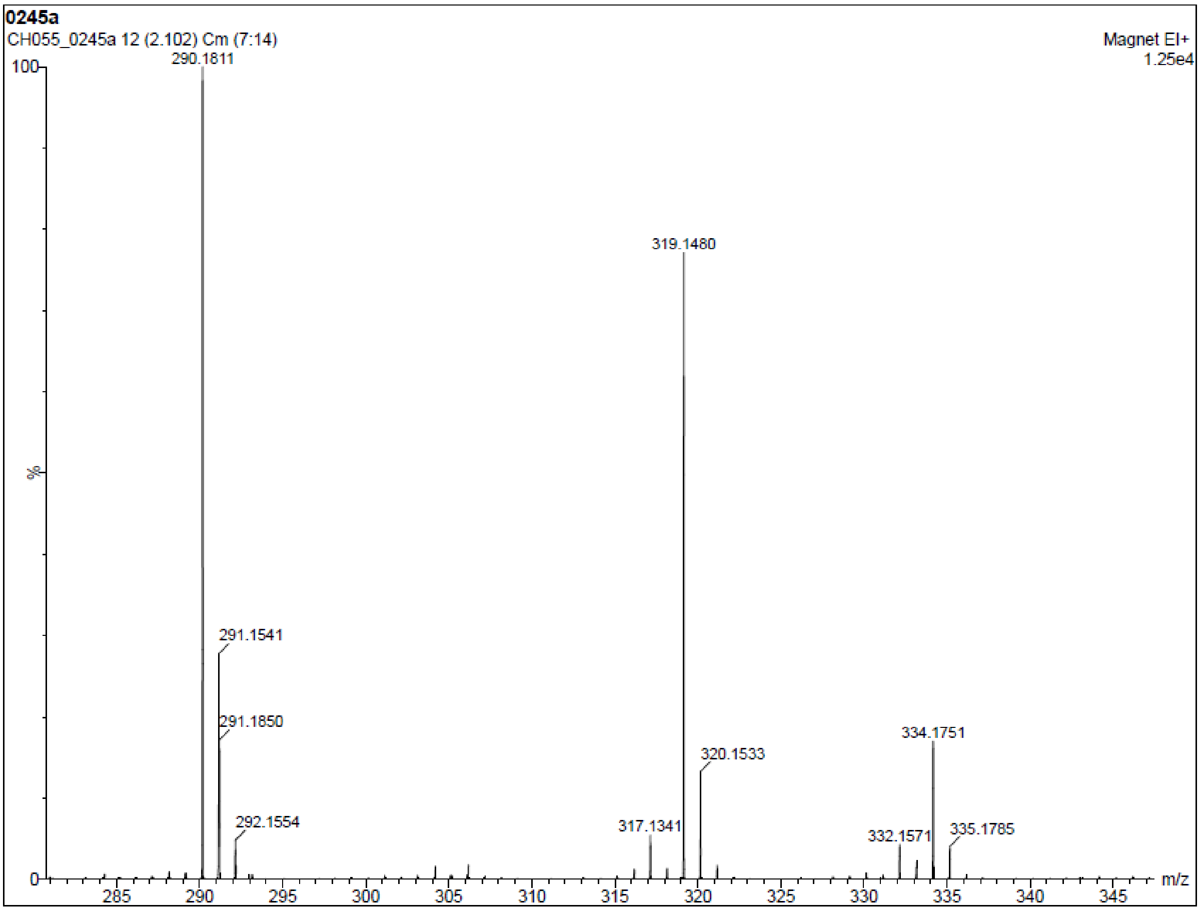
0245a
CH055_0245a 12 (2.102) Cm (7:14)
Magnet EI+
1.25e4
100
%
0
290.1811
319.1480
291.1541
291.1850
292.1554
317.1341
320.1533
334.1751
332.1571
335.1785
285
290
295
300
305
310
315
320
325
330
335
340
345
m/z

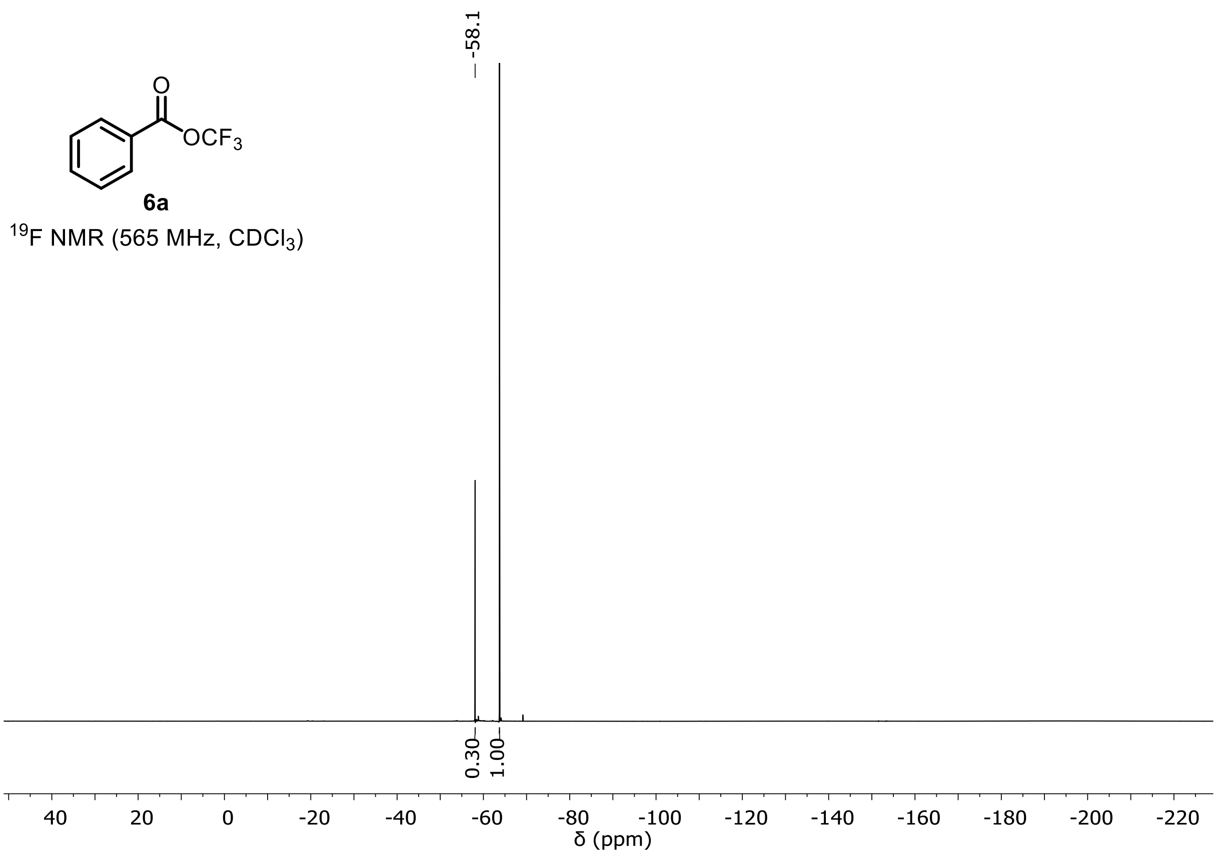

O
OCF3
6a
19F NMR (565 MHz, CDCl3)
-58.1
0.30
1.00
40
20
0
-20
-40
-60
-80
-100
-120
-140
-160
-180
-200
-220
δ (ppm)

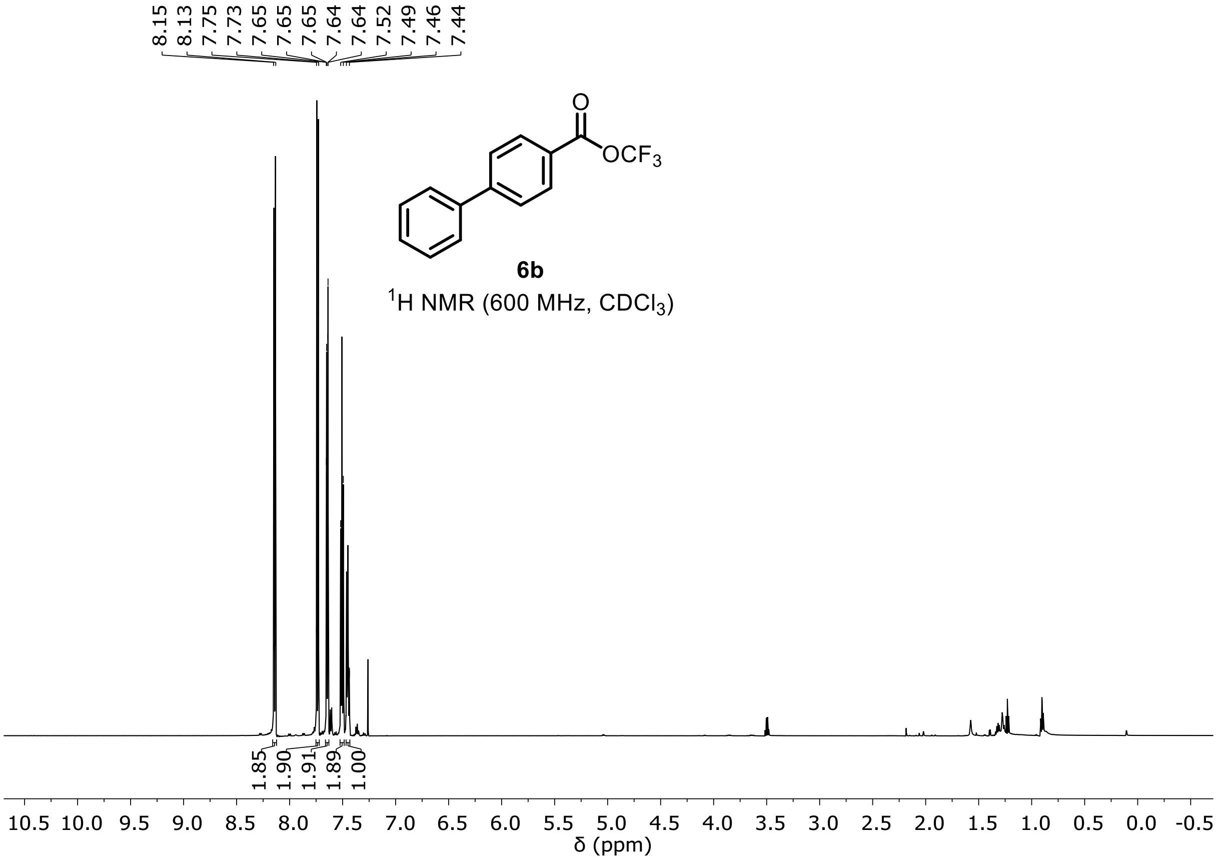
8.15
8.13
7.75
7.73
7.65
7.65
7.65
7.64
7.64
7.52
7.49
7.46
7.44
OCF3
O
6b
1H NMR (600 MHz, CDCl3)
1.85
1.90
1.91
1.89
1.00
10.5 10.0 9.5 9.0 8.5 8.0 7.5 7.0 6.5 6.0 5.5 5.0 4.5 4.0 3.5 3.0 2.5 2.0 1.5 1.0 0.5 0.0 -0.5
δ (ppm)

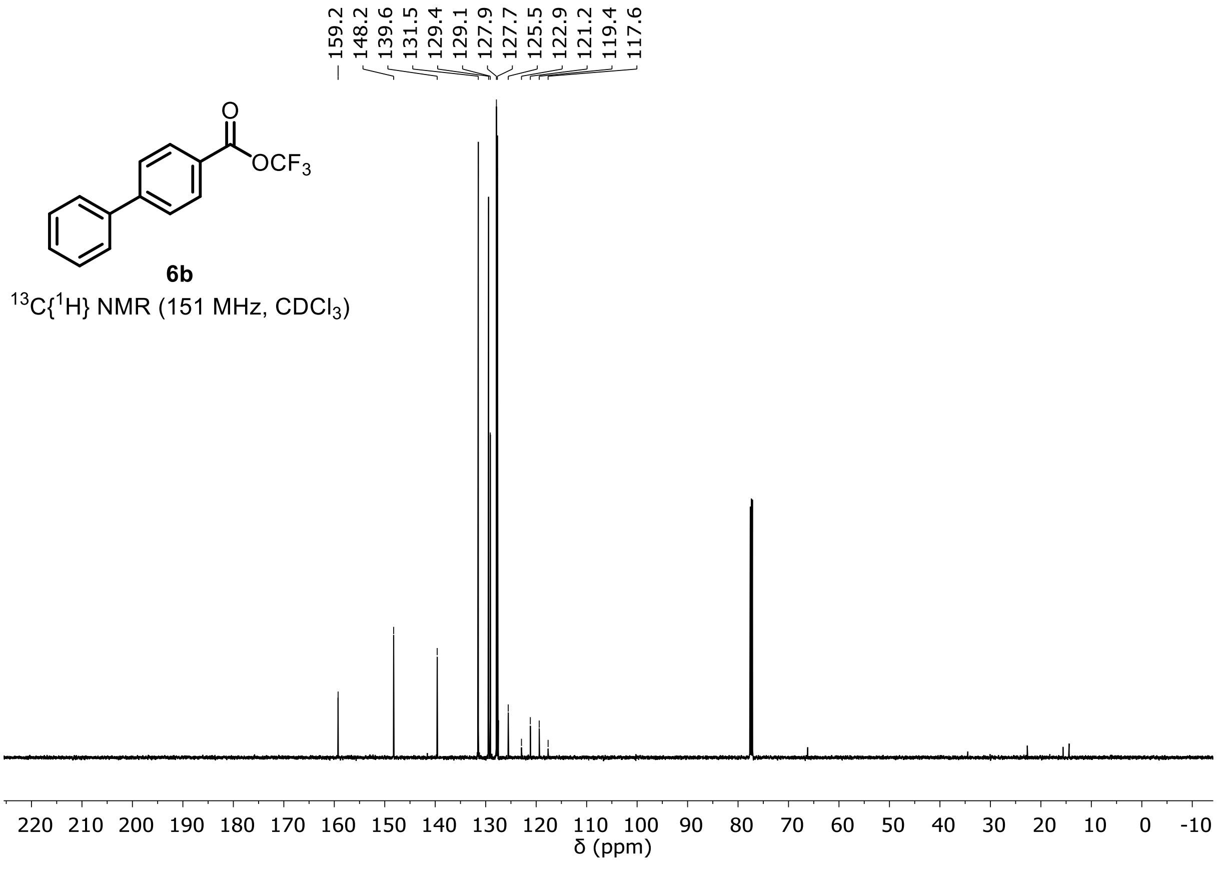
159.2
148.2
139.6
131.5
129.4
129.1
127.9
127.7
125.5
122.9
121.2
119.4
117.6
O
OCF3
6b
13C{1H} NMR (151 MHz, CDCl3)
220 210 200 190 180 170 160 150 140 130 120 110 100 90 80 70 60 50 40 30 20 10 0 -10
δ (ppm)

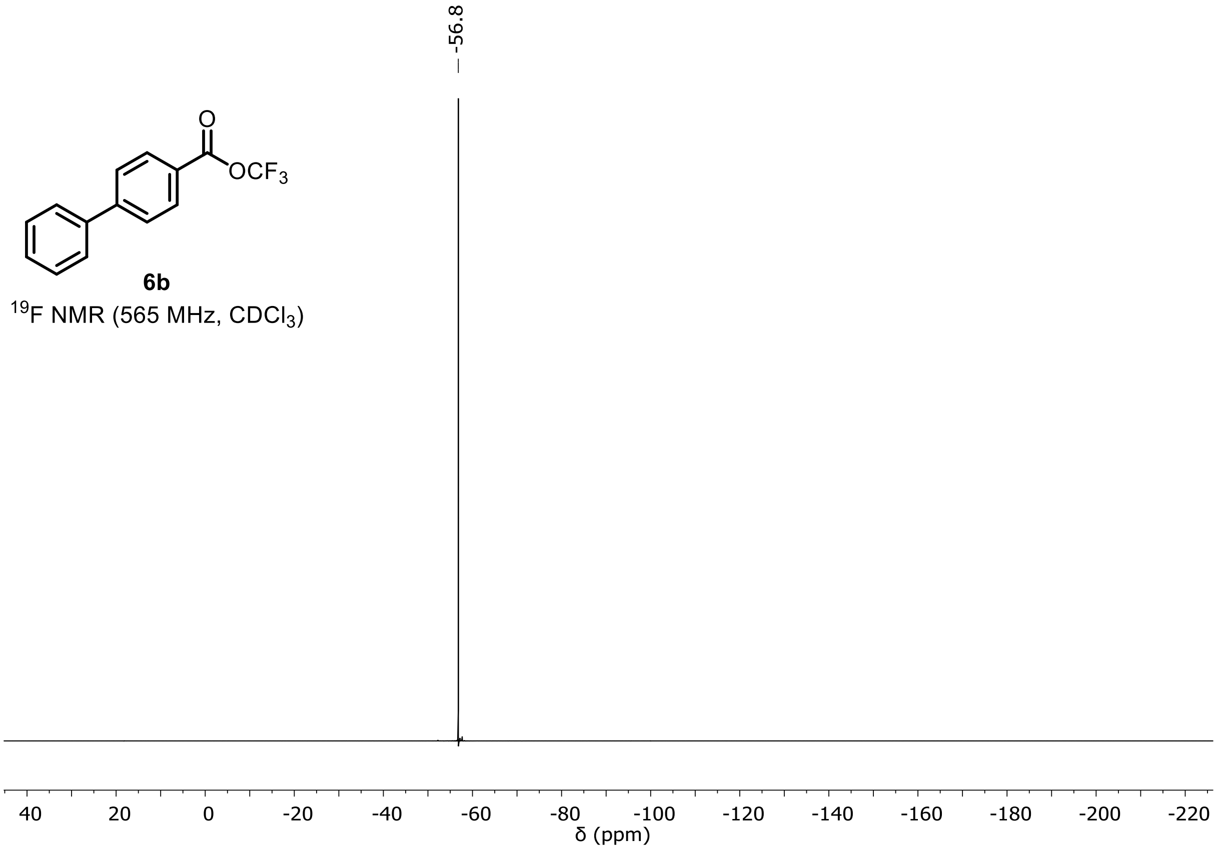
-56.8
O
OCF3
6b
19F NMR (565 MHz, CDCl3)
40 20 0 -20 -40 -60 -80 -100 -120 -140 -160 -180 -200 -220
δ (ppm)

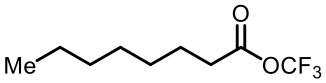

6c

^{19}F NMR (376 MHz, $CDCl_3$)

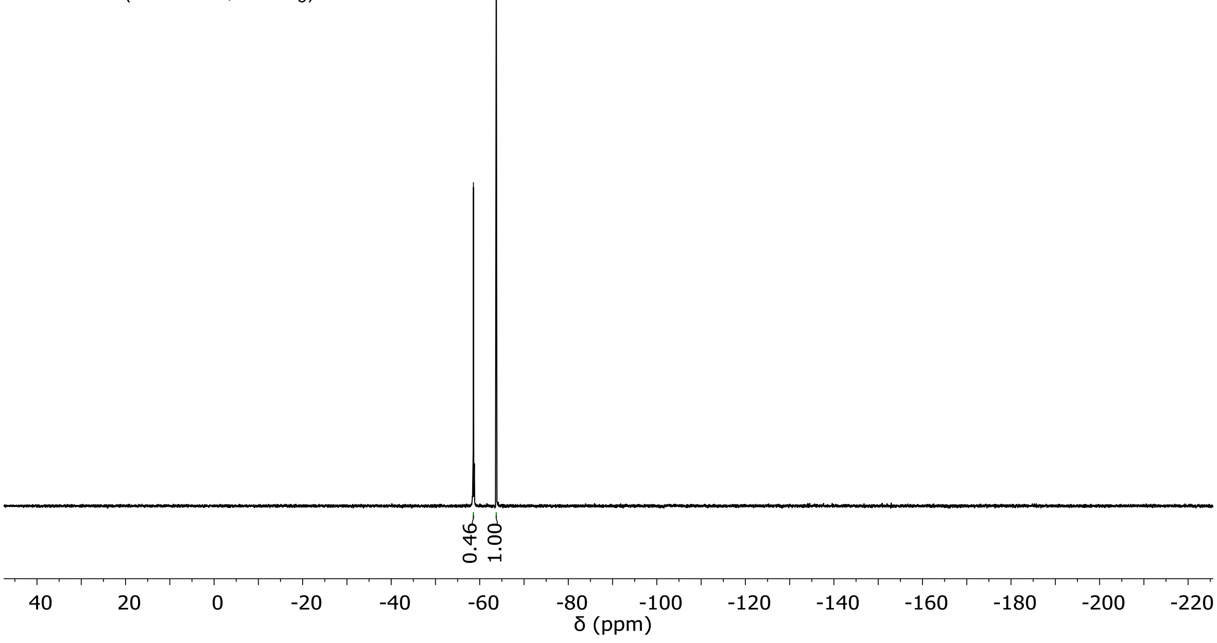

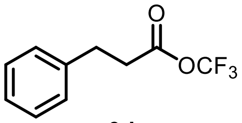

6d

^{19}F NMR (565 MHz, $CDCl_3$)

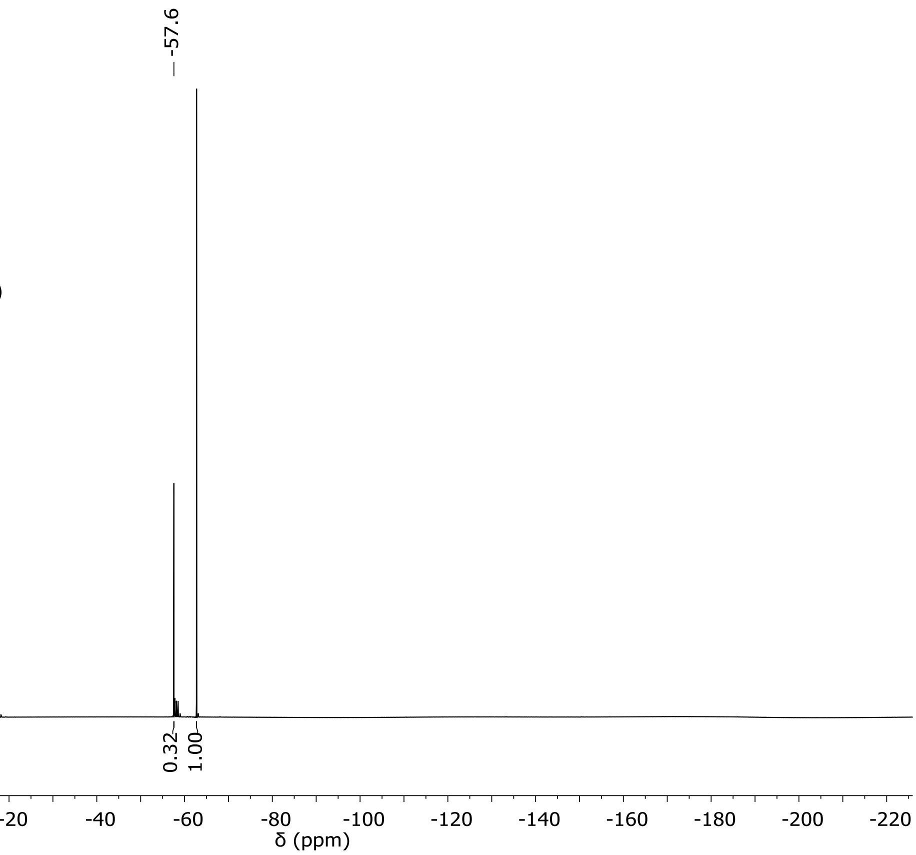

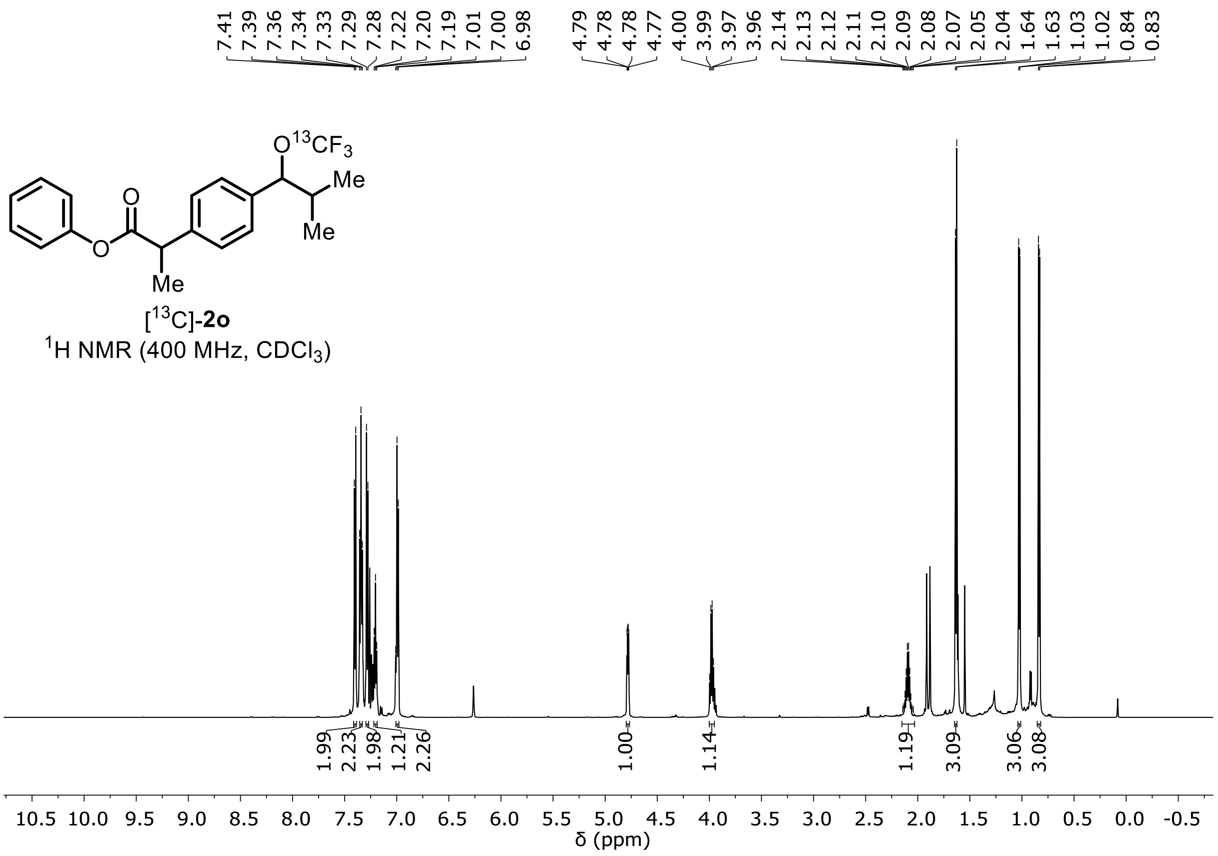

[^{13}C]-**2o**
^{1}H NMR (400 MHz, $CDCl_3$)

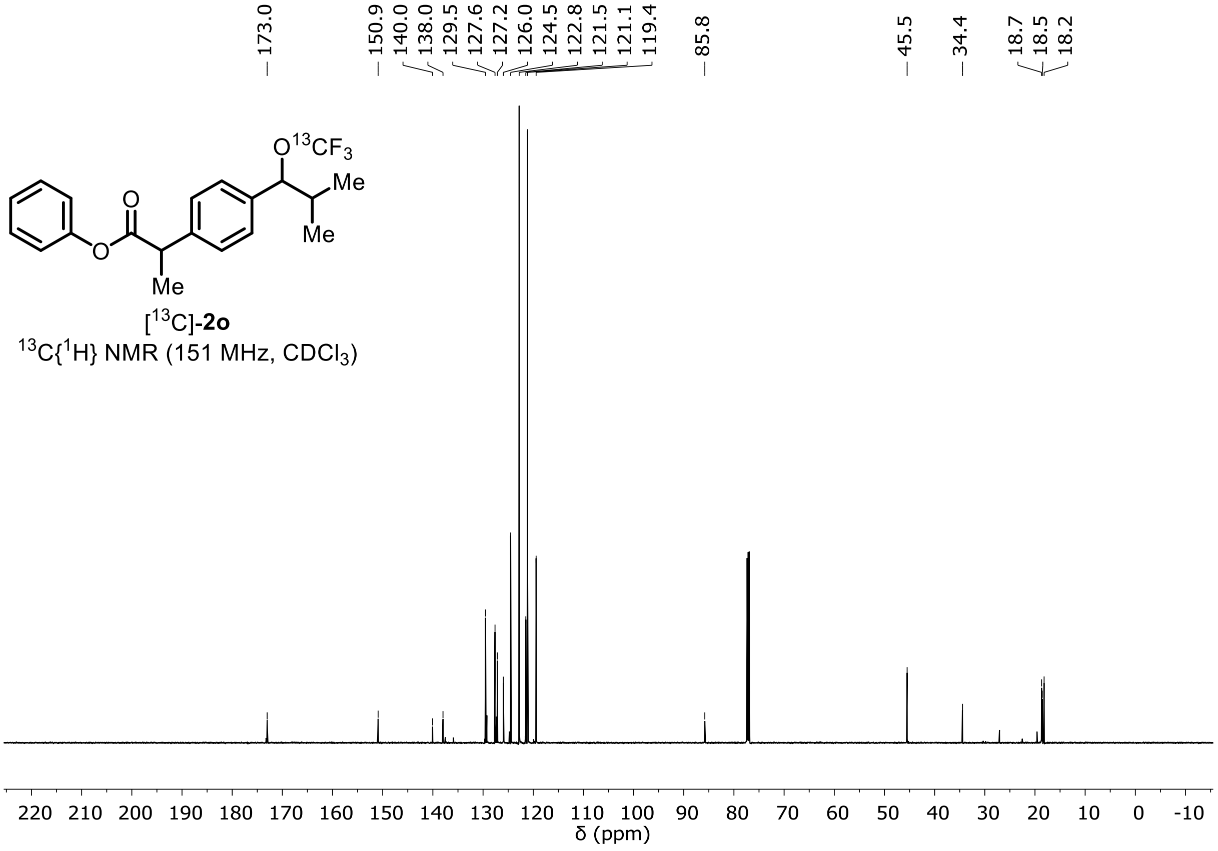

[^{13}C]-**2o**
^{13}C{^{1}H} NMR (151 MHz, $CDCl_3$)

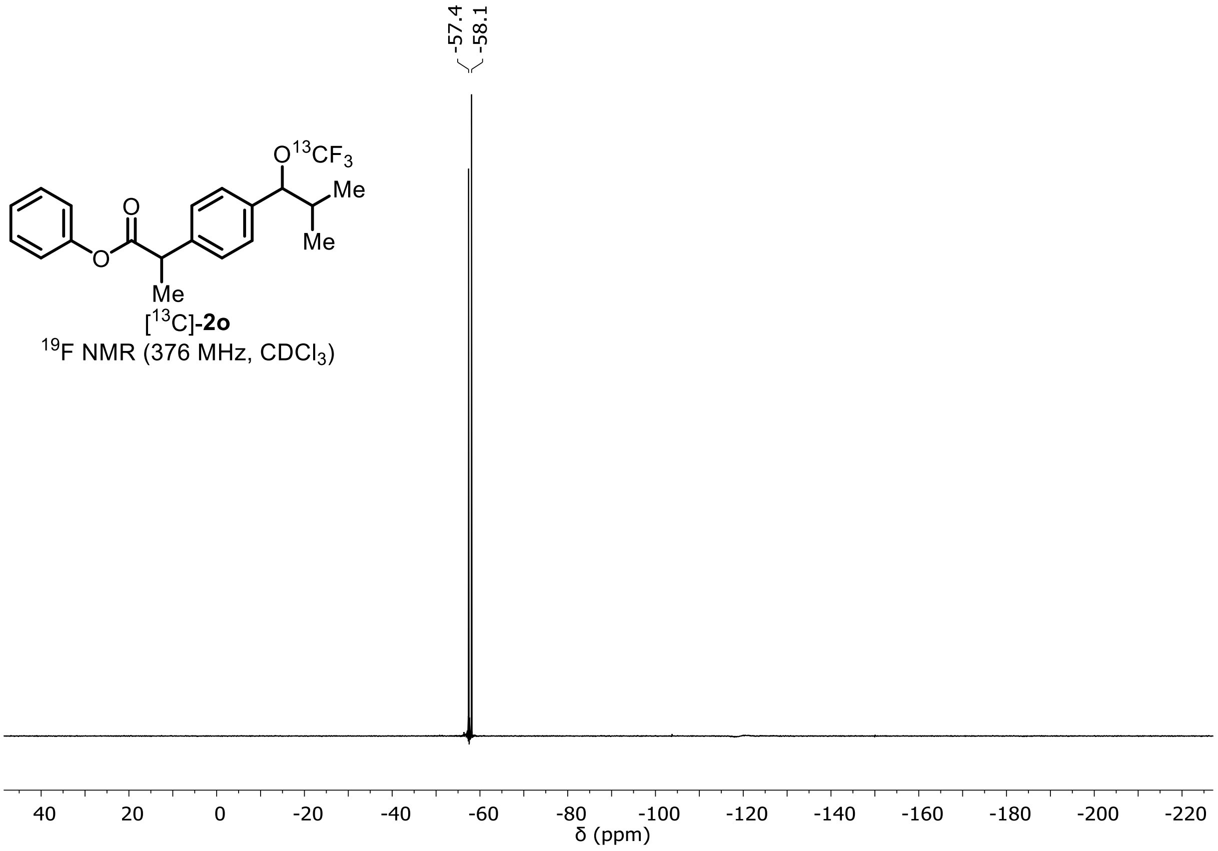

[^{13}C]-**2o**

^{19}F NMR (376 MHz, $CDCl_3$)

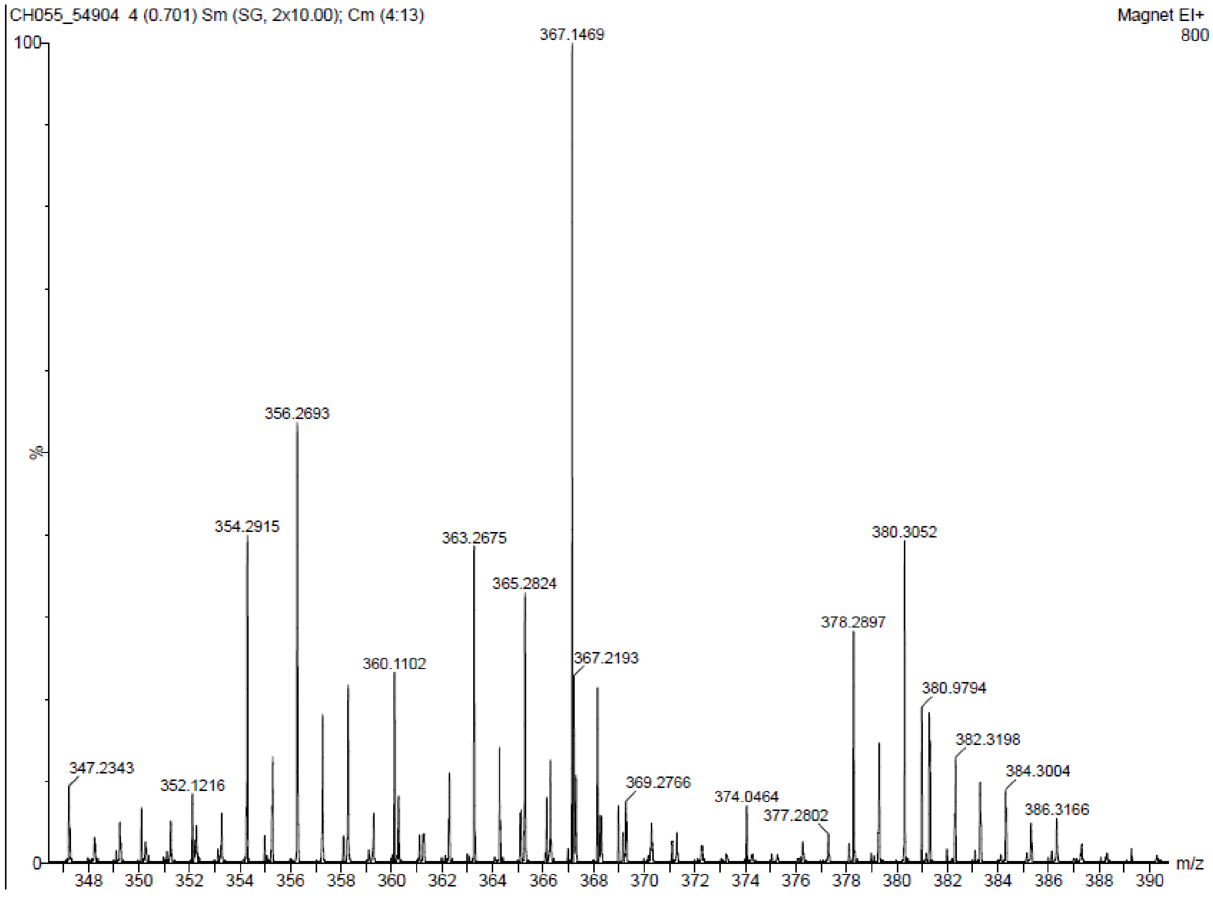

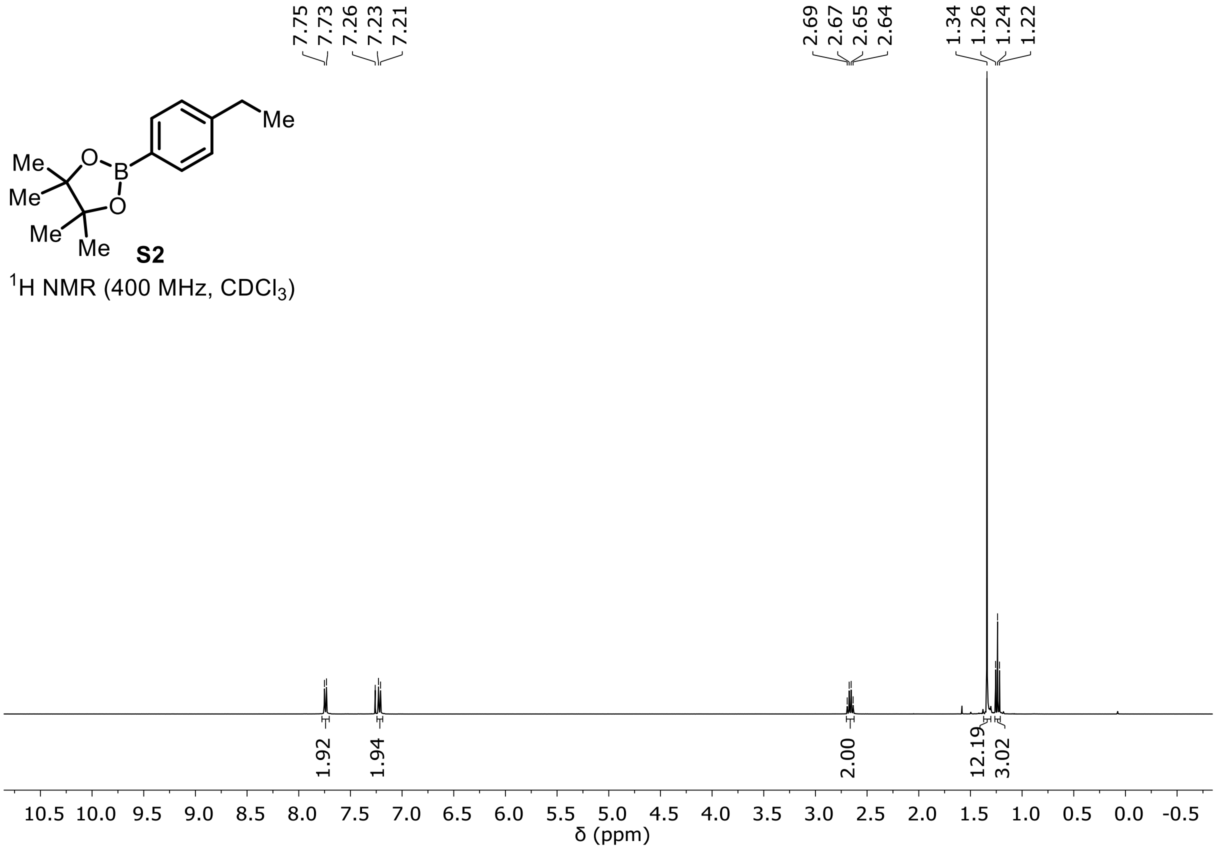
7.75
7.73
7.26
7.23
7.21
2.69
2.67
2.65
2.64
1.34
1.26
1.24
1.22
Me
Me
Me
Me
Me
O
B
O
S2
^{1}H NMR (400 MHz, CDCl$_3$)
1.92
1.94
2.00
12.19
3.02
10.5 10.0 9.5 9.0 8.5 8.0 7.5 7.0 6.5 6.0 5.5 5.0 4.5 4.0 3.5 3.0 2.5 2.0 1.5 1.0 0.5 0.0 -0.5
δ (ppm)

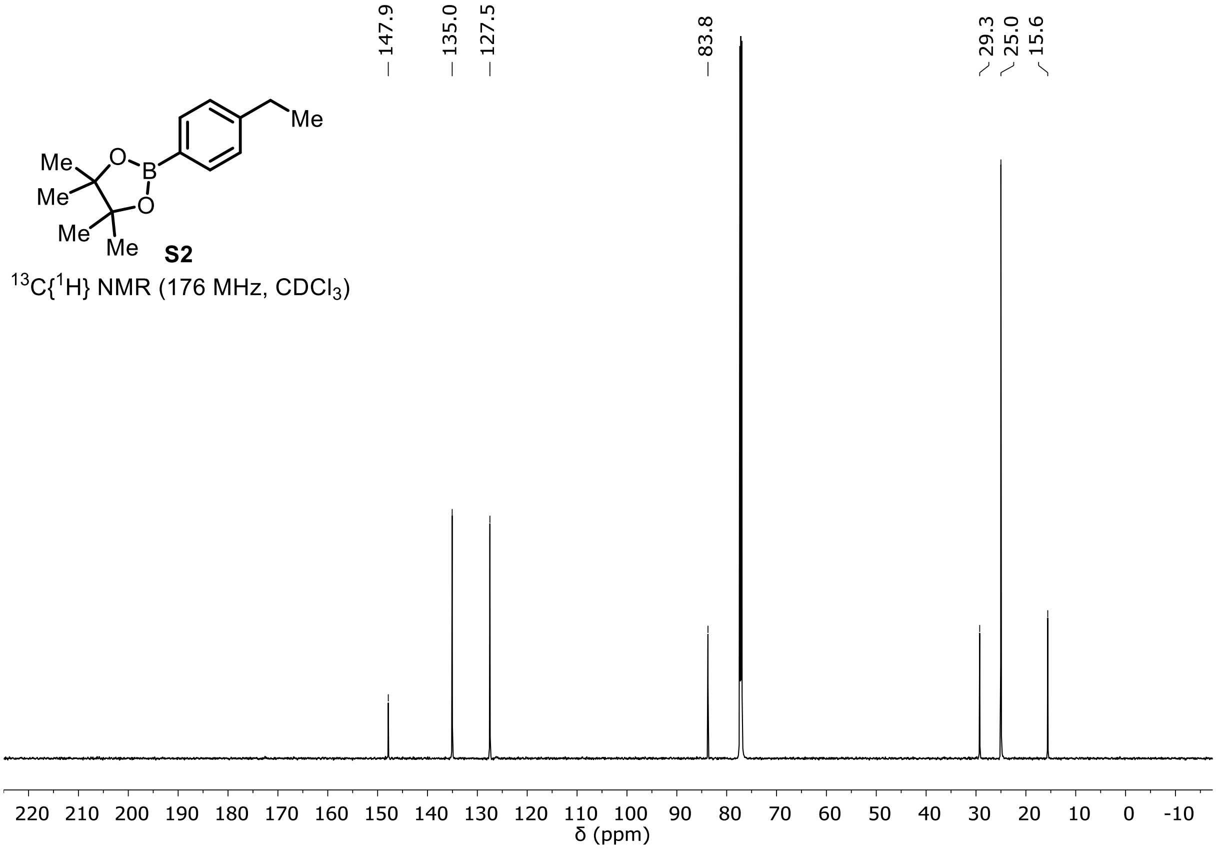
147.9
135.0
127.5
83.8
29.3
25.0
15.6
Me
Me
Me
Me
Me
O
B
O
S2
^{13}C{^{1}H} NMR (176 MHz, CDCl$_3$)
220 210 200 190 180 170 160 150 140 130 120 110 100 90 80 70 60 50 40 30 20 10 0 -10
δ (ppm)

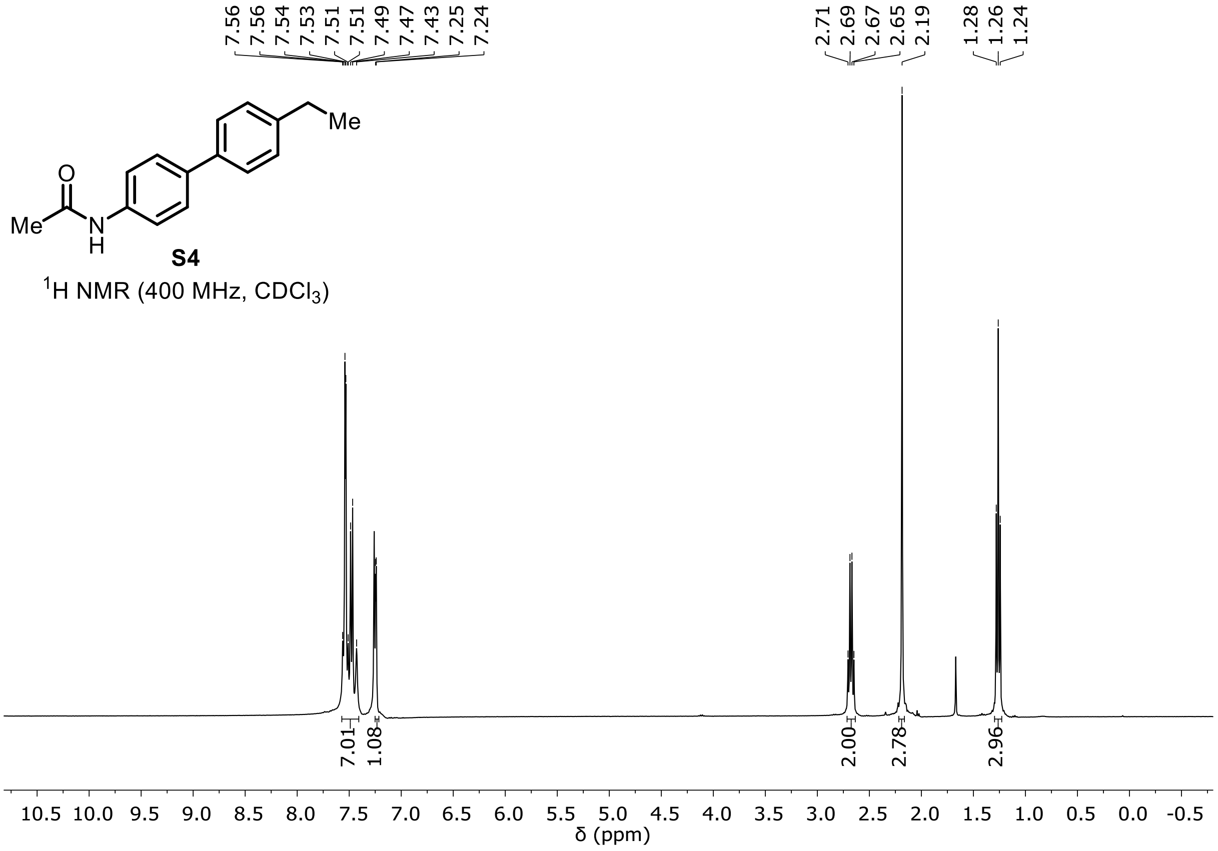
7.56
7.56
7.54
7.53
7.51
7.51
7.49
7.47
7.43
7.25
7.24
2.71
2.69
2.67
2.65
2.19
1.28
1.26
1.24
Me
O
Me
N
H
S4
¹H NMR (400 MHz, CDCl₃)
7.01
1.08
2.00
2.78
2.96
10.5 10.0 9.5 9.0 8.5 8.0 7.5 7.0 6.5 6.0 5.5 5.0 4.5 4.0 3.5 3.0 2.5 2.0 1.5 1.0 0.5 0.0 -0.5
δ (ppm)

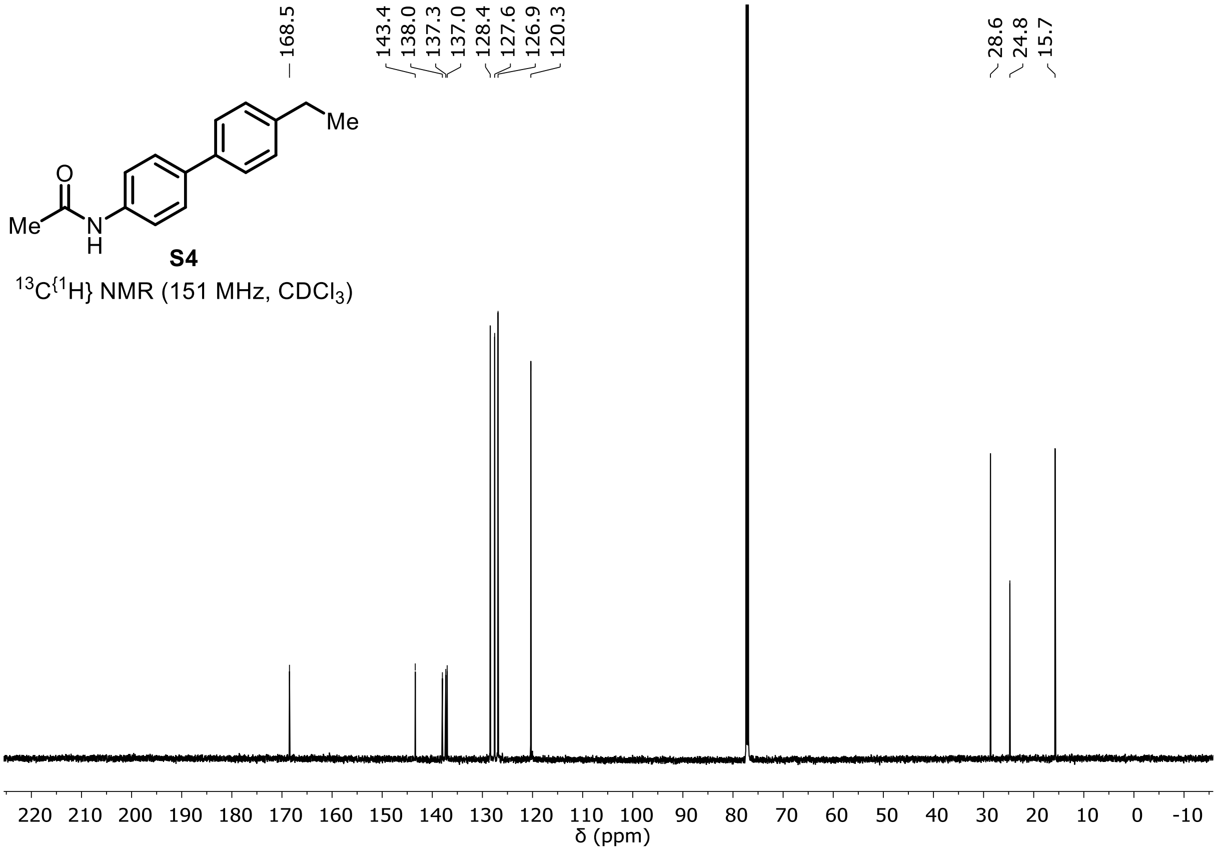
168.5
143.4
138.0
137.3
137.0
128.4
127.6
126.9
120.3
28.6
24.8
15.7
Me
O
Me
N
H
S4
¹³C{¹H} NMR (151 MHz, CDCl₃)
220 210 200 190 180 170 160 150 140 130 120 110 100 90 80 70 60 50 40 30 20 10 0 -10
δ (ppm)

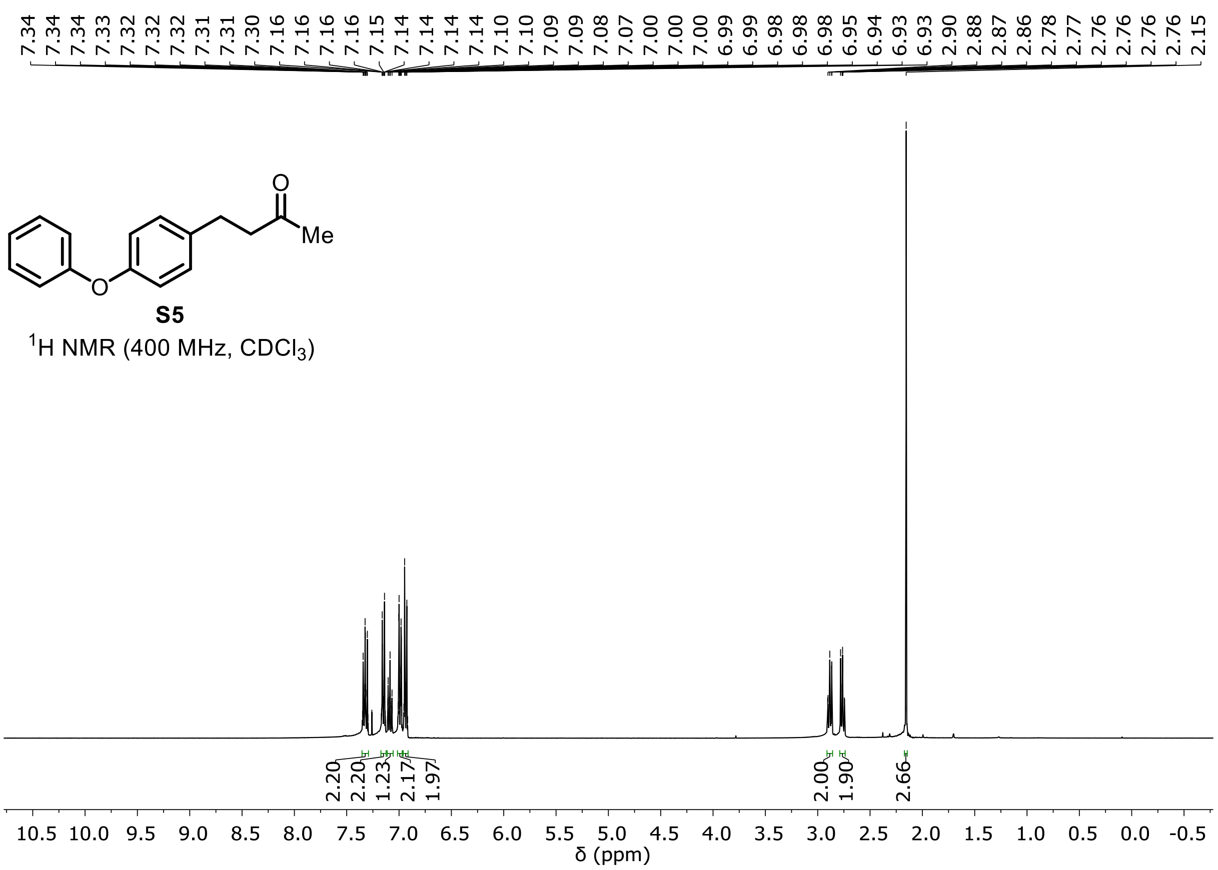
7.34 7.34 7.34 7.33 7.32 7.32 7.32 7.31 7.31 7.30 7.16 7.16 7.16 7.16 7.15 7.14 7.14 7.14 7.14 7.10 7.10 7.09 7.09 7.08 7.07 7.00 7.00 7.00 6.99 6.99 6.98 6.98 6.98 6.95 6.94 6.93 6.93 2.90 2.88 2.87 2.86 2.78 2.77 2.76 2.76 2.76 2.15
O
Me
O
S5
^{1}H NMR (400 MHz, $CDCl_3$)
2.20 2.20 1.23 2.17 1.97
2.00 1.90
2.66
10.5 10.0 9.5 9.0 8.5 8.0 7.5 7.0 6.5 6.0 5.5 5.0 4.5 4.0 3.5 3.0 2.5 2.0 1.5 1.0 0.5 0.0 -0.5
δ (ppm)

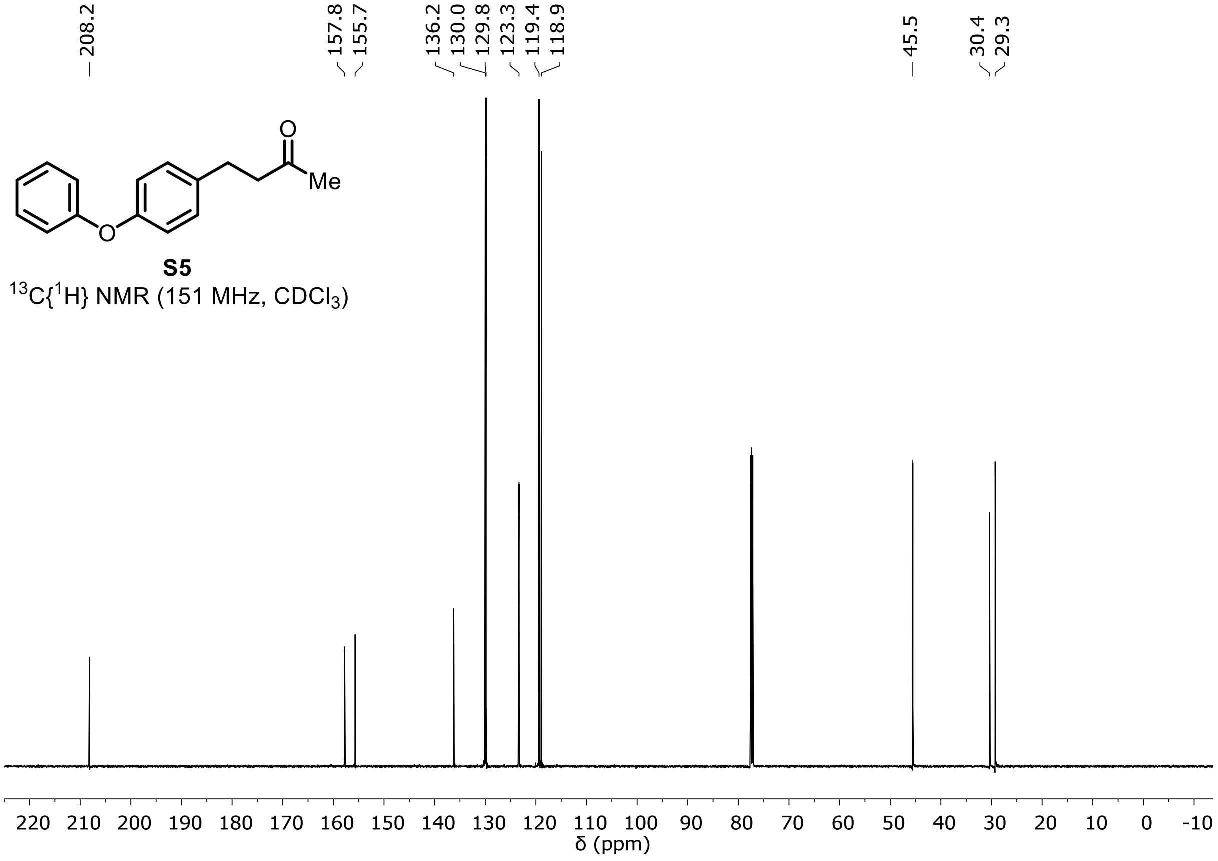
208.2
157.8 155.7
136.2 130.0 129.8 123.3 119.4 118.9
45.5
30.4 29.3
O
Me
O
S5
^{13}C{^{1}H} NMR (151 MHz, $CDCl_3$)
220 210 200 190 180 170 160 150 140 130 120 110 100 90 80 70 60 50 40 30 20 10 0 -10
δ (ppm)

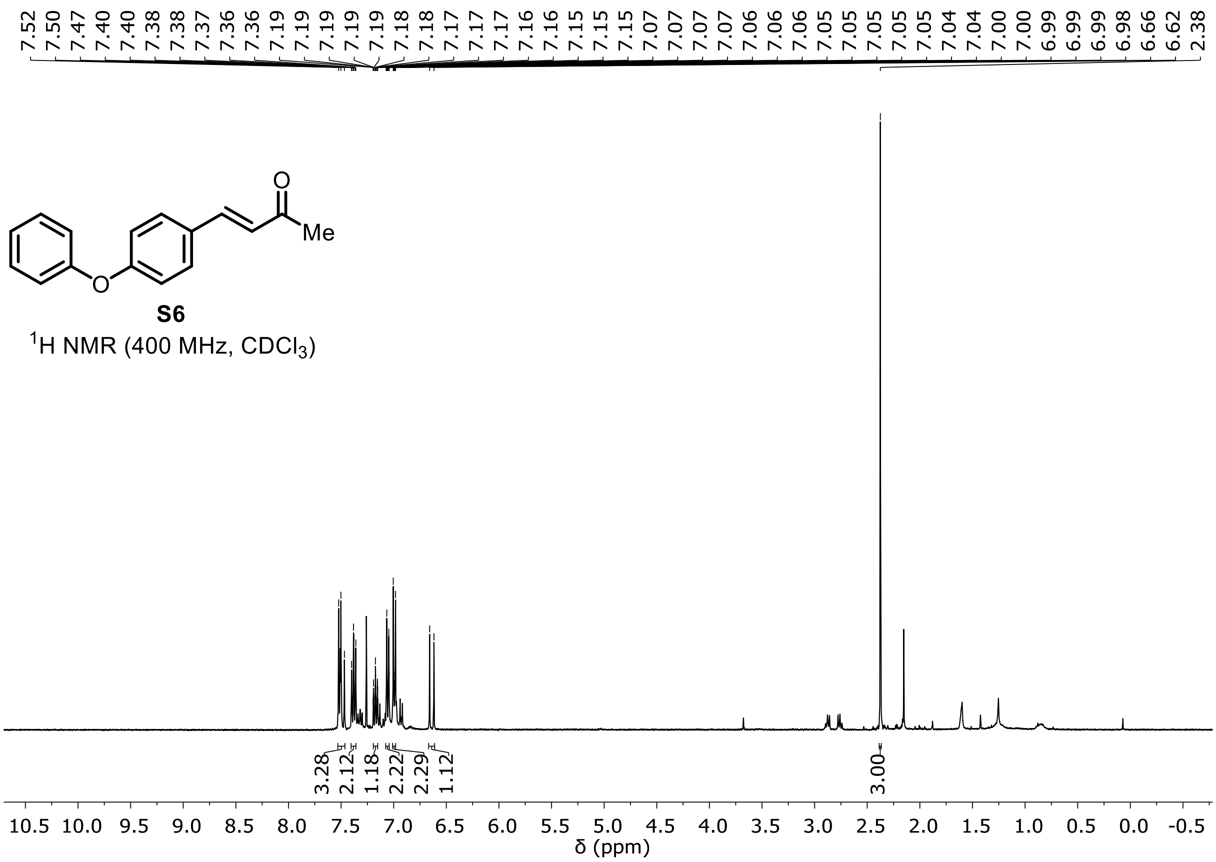
7.52 7.50 7.47 7.40 7.40 7.38 7.38 7.37 7.36 7.36 7.19 7.19 7.19 7.19 7.19 7.18 7.18 7.17 7.17 7.17 7.16 7.16 7.15 7.15 7.15 7.07 7.07 7.07 7.07 7.06 7.06 7.06 7.05 7.05 7.05 7.05 7.05 7.04 7.04 7.00 7.00 6.99 6.99 6.98 6.66 6.62 2.38
O
Me
O
S6
^{1}H NMR (400 MHz, CDCl$_3$)
3.28 2.12 1.18 2.22 2.29 1.12
3.00
10.5 10.0 9.5 9.0 8.5 8.0 7.5 7.0 6.5 6.0 5.5 5.0 4.5 4.0 3.5 3.0 2.5 2.0 1.5 1.0 0.5 0.0 -0.5
δ (ppm)

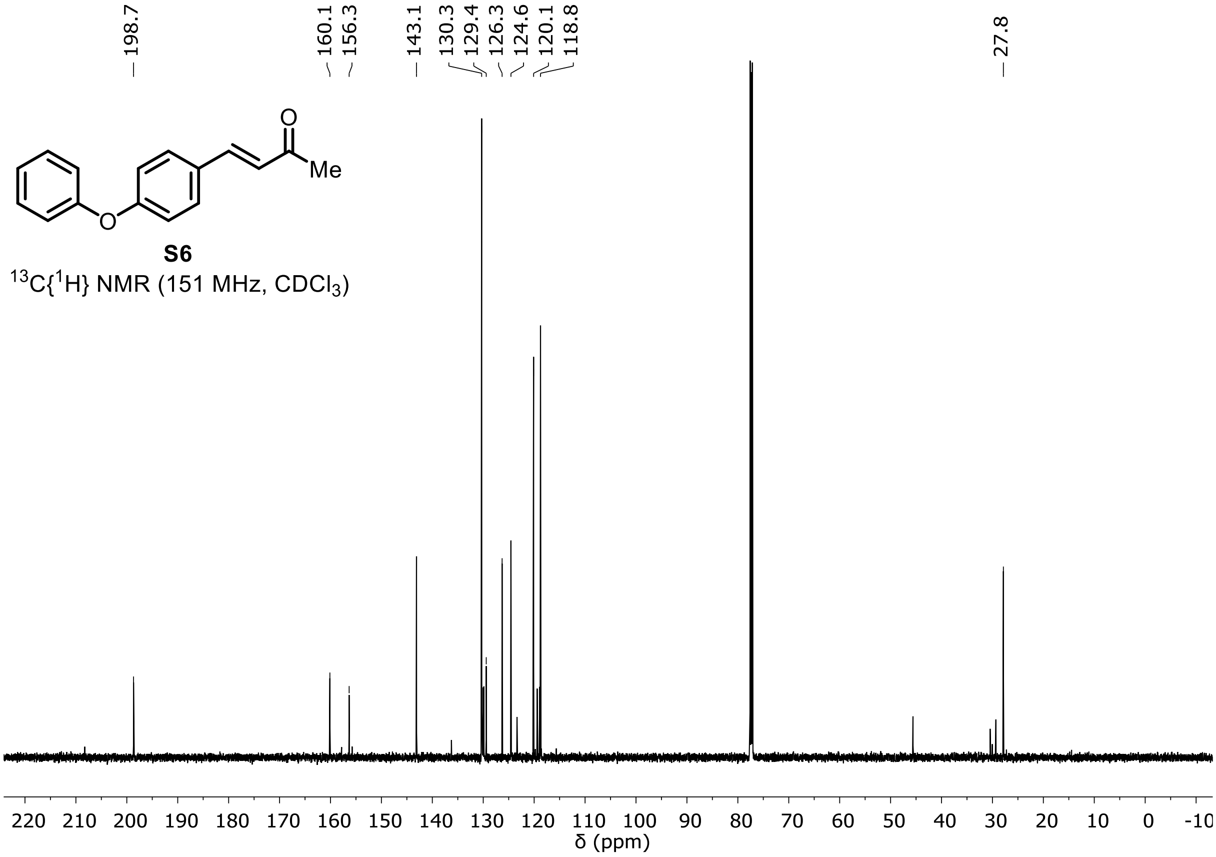
198.7
160.1 156.3
143.1
130.3 129.4 126.3 124.6 120.1 118.8
27.8
O
Me
O
S6
^{13}C{^{1}H} NMR (151 MHz, CDCl$_3$)
220 210 200 190 180 170 160 150 140 130 120 110 100 90 80 70 60 50 40 30 20 10 0 -10
δ (ppm)

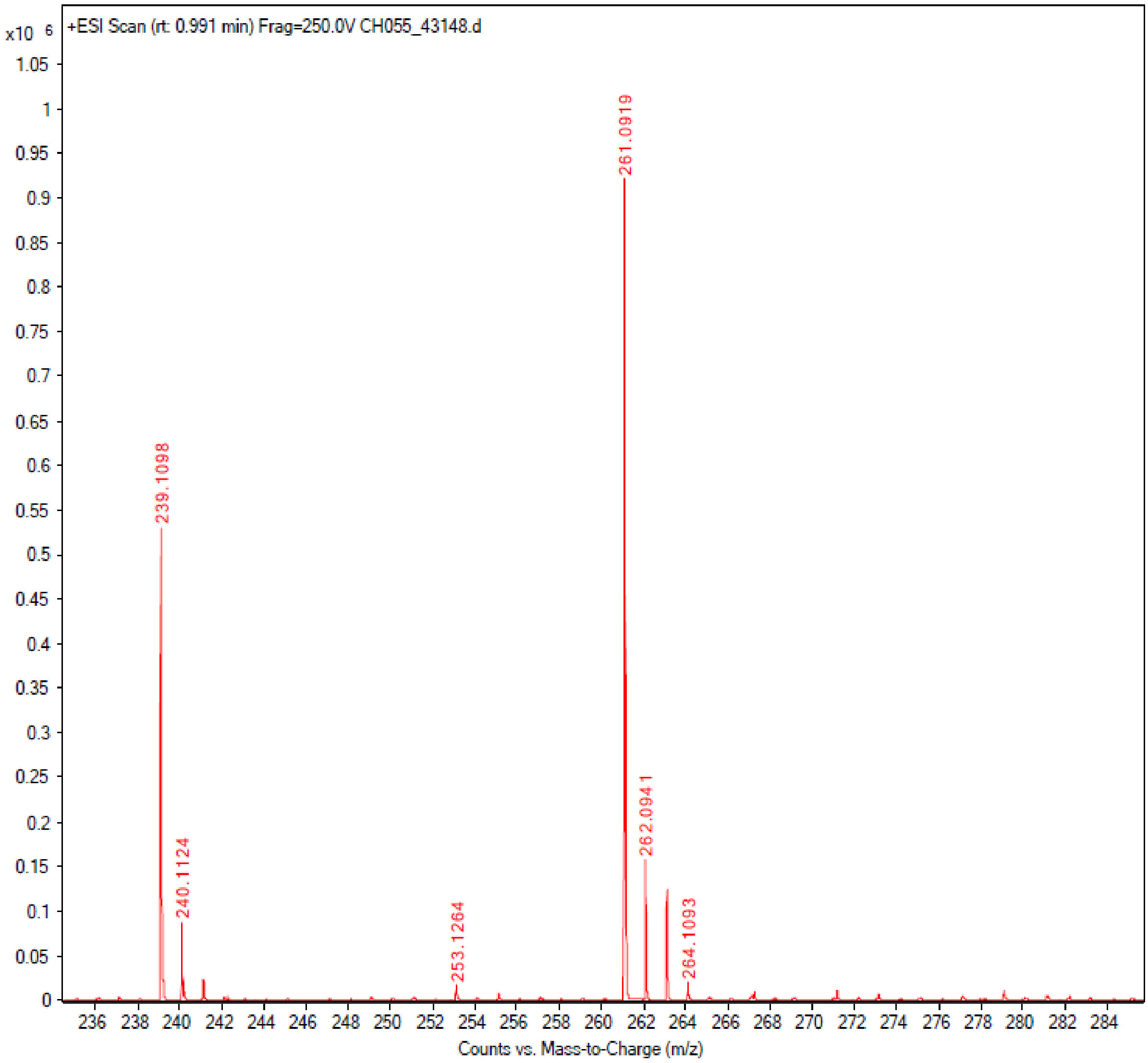

x10 6
+ESI Scan (rt: 0.991 min) Frag=250.0V CH055_43148.d
1.05
1
0.95
0.9
0.85
0.8
0.75
0.7
0.65
0.6
0.55
0.5
0.45
0.4
0.35
0.3
0.25
0.2
0.15
0.1
0.05
0
261.0919
239.1098
262.0941
240.1124
264.1093
253.1264
236 238 240 242 244 246 248 250 252 254 256 258 260 262 264 266 268 270 272 274 276 278 280 282 284
Counts vs. Mass-to-Charge (m/z)

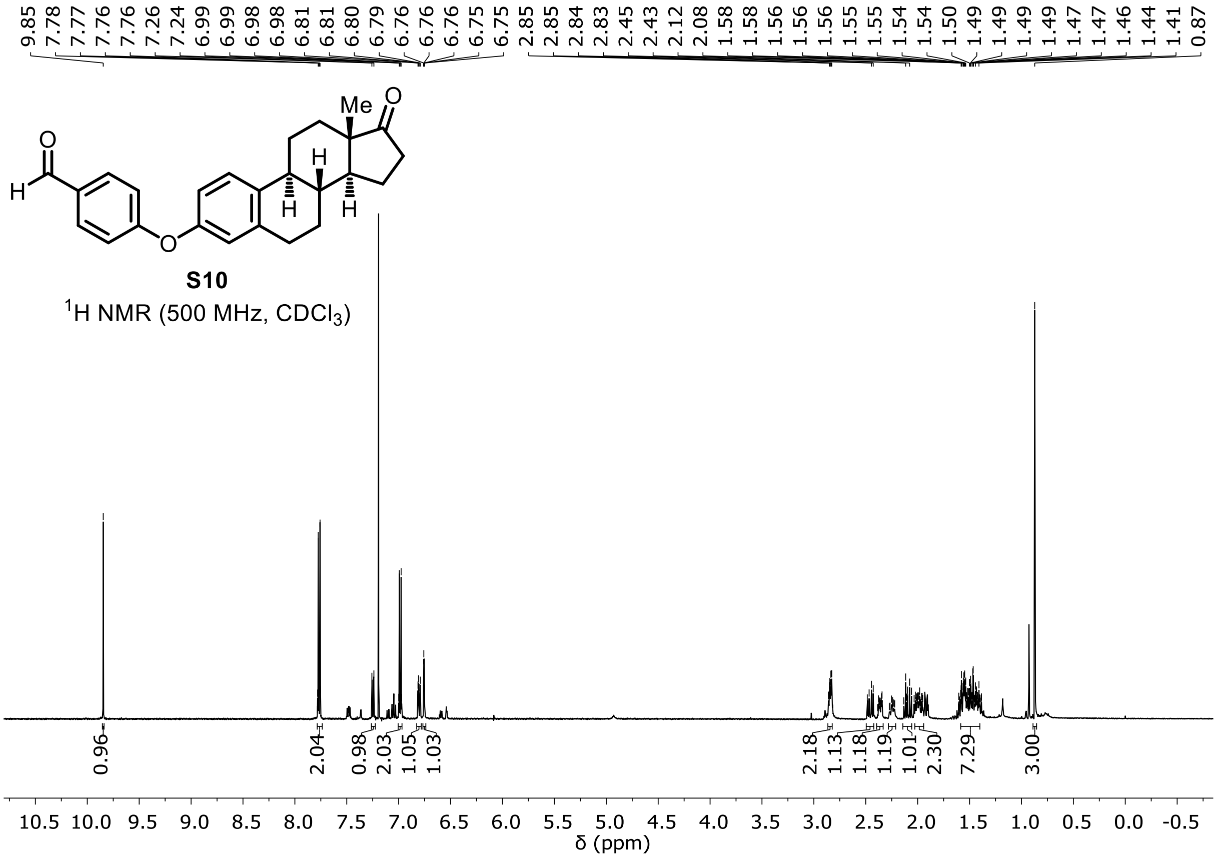

S10

^{1}H NMR (500 MHz, CDCl$_3$)

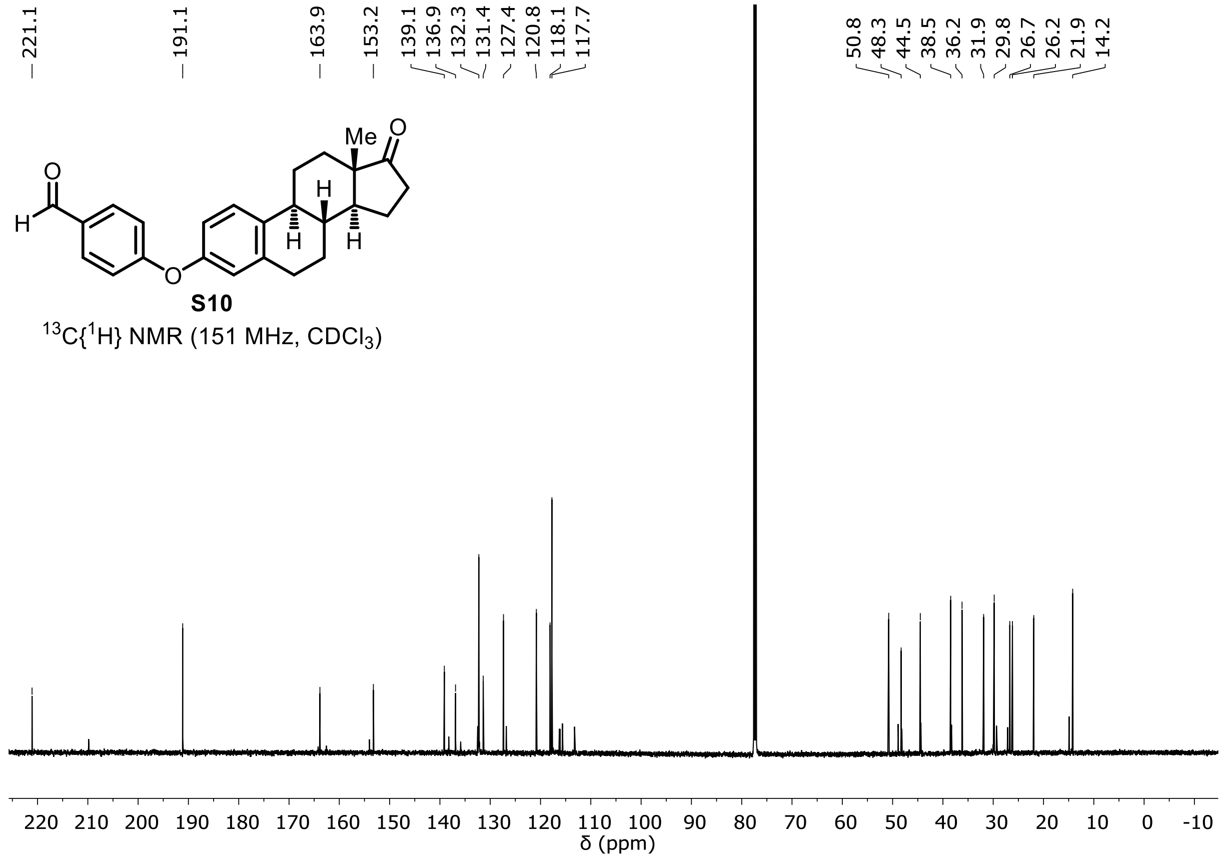

S10

^{13}C{^{1}H} NMR (151 MHz, CDCl$_3$)

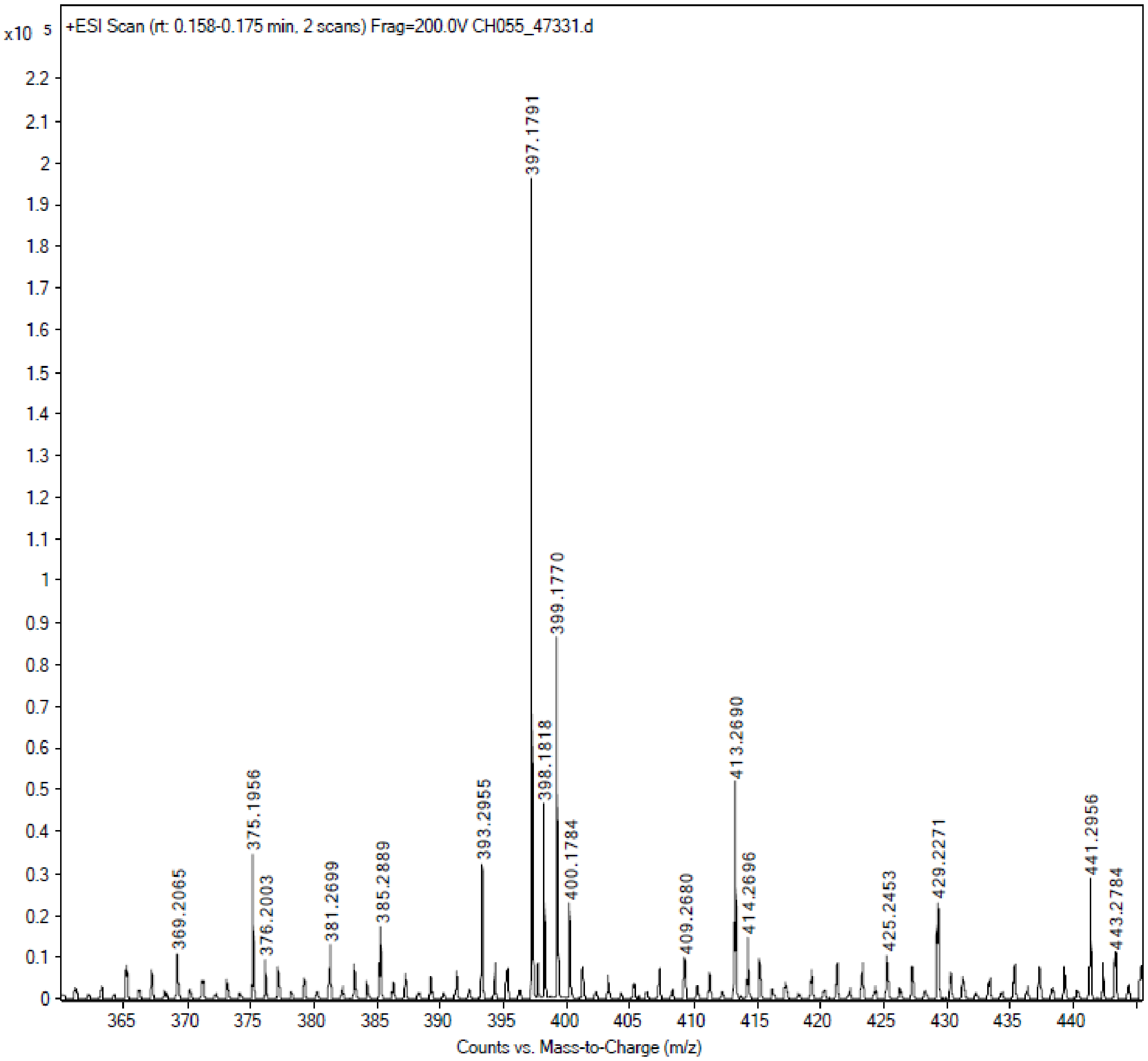
x10 5
+ESI Scan (rt: 0.158-0.175 min, 2 scans) Frag=200.0V CH055_47331.d
397.1791
399.1770
413.2690
398.1818
375.1956
393.2955
400.1784
441.2956
429.2271
414.2696
385.2889
381.2699
369.2065
409.2680
425.2453
443.2784
376.2003
Counts vs. Mass-to-Charge (m/z)

16. References

[1] Protti, S.; Ravelli, D.; Fagnoni, M.; Albini, A. Solar light-driven photocatalyzed alkylations. Chemistry on the window ledge *Chem. Commun.* **2009**, *47*, 7351–7353.

[2] Sarver, P. J.; Bacauanu, V.; Schultz, D. M.; DiRocco, D. A.; Lam, Y.; Sherer, E. C.; MacMillan, D. W. C. The merger of decatungstate and copper catalysis to enable aliphatic C(sp^3)–H trifluoromethylation *Nat. Chem.* **2020**, *12*, 459–467.

[3] Ruff, O.; Miltschitzky, G. Das Kohlenoxyfluorid COF_2 *Z. Anorg. Allg. Chem.* **1934**, *221*, 154–158.

[4] Dix, S.; Golz, P.; Schmid, J. R.; Riedel, S.; Hopkinson, M. N. Radical C−H Trifluoromethoxylation of (Hetero)arenes with Bis(trifluoromethyl)peroxide *Chem. Eur. J.* **2021**, *27*, 11554–11558.

[5] Yang, H.; Wang, F.; Jiang, X.; Zhou, Y.; Xu, X; Tang, P. Silver-Promoted Oxidative Benzylic C−H Trifluoromethoxylation *Angew. Chem. Int. Ed.* **2018**, *57*, 13266–13270.

[6] Cismesia, M. A.; Yoon, T. P. Characterizing chain processes in visible light photoredox catalysis *Chem. Sci.* **2015**, *6*, 5426–5434.

[7] Liu, N.; Wang, Z.-X. Kumada coupling of aryl, heteroaryl, and vinyl chlorides catalyzed by amido pincer nickel complexes *J. Org. Chem.* **2011**, *76*, 10031–10038.

[8] Espinal-Viguri, M.; Neale, S.E.; Coles, N. T.; Macgregor, S. A.; Webster, R. L. Room Temperature Iron-Catalyzed Transfer Hydrogenation and Regioselective Deuteration of Carbon–Carbon Double Bonds *J. Am. Chem. Soc.* **2019**, *141*, 572–582.

[9] Zhao, J.; Shen, T.; Sun, Z.; Wang, N.; Yang, L.; Wu, J.; You, H.; Liu, Z.-Q. Site-Specific Oxidation of (sp^3)C–C(sp^3)/H Bonds by $NaNO_2$/HCl *Org. Lett.* **2021**, *23*, 4057–4061.

[10] Iglesias, M. J.; Prieto, A.; Nicasio, M. C. Kumada–Tamao–Corriu Coupling of Heteroaromatic Chlorides and Aryl Ethers Catalyzed by (IPr)Ni(allyl)Cl *Org. Lett.* **2012**, *14*, 4318–4321.

[11] Day, C. S.; Fawcett, A.; Chatterjee, R.; Hartwig, J. F. *J. Am. Chem. Soc.* **2021**, *143*, 16184–16196.

[12] Liu, S.; Achou, R.; Boulanger, C.; Pawar, G.; Kumar, N.; Lusseau, J.; Robert, F.; Landais, Y. Copper-catalyzed oxidative benzylic C(sp^3)–H amination: direct synthesis of benzylic carbamates *Chem. Commun.* **2020**, *56*, 13013–13016.

[13] Akrami, Z.; Hosseini-Sarvari, M. Ni/g-C_3N_4 Photocatalysis: Aerobic Oxidative Coupling Reaction Leading to Amidation of Aldehydes with Amines and C−N, C−O, and C−C Cross-Coupling Reaction *Eur. J. Org. Chem.* **2022**, e202200429.

[14] Li, X.; Teng, Y.; Feng, F.; Hu, Q.; Yuan, Z. Aqueous Suzuki-Miyaura Reaction with 0.6 Equiv of Base: Green and Efficient Access to Biaryls and Unsymmetrical Terphenyls *ChemistrySelect* **2018**, *3*, 6022–6027.

[15] Barber, T.; Argent, S. P.; Ball, L. T. Expanding Ligand Space: Preparation, Characterization, and Synthetic Applications of Air-Stable, Odorless Di-tert-alkylphosphine Surrogates *ACS Catal.* **2020**, *10*, 5454–5461.

[16] Ren, W.; Chang, W.; Wang, Y.; Li, J.; Shi, Y. Pd-Catalyzed Regiodivergent Hydroesterification of Aryl Olefins with Phenyl Formate *Org. Lett.* **2015**, *17*, 3544–3547.

[17] Aman, H.; Chen, Y.-C.; Tu, J.-W.; Chang, C.-C.; Chuang, G. J. Catalyst/Additive Free Oxidation of Benzyl Bromides to Benzaldehydes *ChemistrySelect* **2020**, *5*, 15015–15019.

[18] Golz, P.; Shakeri, K.; Maas, L.; Balizs, M.; Pérez-Bitrián, A.; Kemmler, H. D.; Kleoff, M.; Voßnacker, P.; Christmann, M.; Riedel, S. Silver(I) Perfluoroalcoholates: Synthesis, Structure, and their Use as Transfer Reagents *Chem. Eur. J.* **2024**, *30*, e202400861.

[19] Koller, R.; Stanek, K.; Stolz, D.; Aardoom, R.; Niedermann, K.; Togni, A. Zinc-Mediated Formation of Trifluoromethyl Ethers from Alcohols and Hypervalent Iodine Trifluoromethylation Reagents *Angew. Chem. Int. Ed.* **2009**, *48*, 4332–4336.

[20] Kalim, J.; Duhail, T.; Pietrasiak, E.; Anselmi, E.; Magnier, E.; Togni, A. Direct Trifluoromethylation of Alcohols Using a Hypervalent Iodosulfoximine Reagent *Chem. Eur. J.* **2021**, *27*, 2638–2642.

[21] Zhou, M.; Ni, C.; Zeng, Y.; Hu, J. Trifluoromethyl Benzoate: A Versatile Trifluoromethoxylation Reagent *J. Am. Chem. Soc.* **2018,** *140*, 6801–6805.

[22] Zhu, H.; Gao, C.; Yu, T.; Xu, C.; Wang, M. O-Trifluoromethylation of Carboxylic Acids via the Formation and Activation of Acyloxy(phenyl)trifluoromethyl-λ^3-Iodanes *Angew. Chem. Int. Ed.* **2024**, *63*, e202400449.

[23] Wang, X.; Cui, P.; Xia, C.; Wu, L. Catalytic Boration of Alkyl Halides with Borane without Hydrodehalogenation Enabled by Titanium Catalyst *Angew. Chem. Int. Ed.* **2021**, *60*, 12298–12303.

[24] Nie, Q.; Yi, F.; Huang, B.; Cai, M. Efficient Heterogeneous Gold(I)-Catalyzed Direct C(sp^2)–C(sp) Bond Functionalization of Arylalkynes through a Nitrogenation Process to Amides *Adv. Synth. Catal.* **2017**, *359*, 3968–3976.

[25] Kuhlmann, J. H.; Dickoff, J. H.; Mancheño, O. G. Visible Light Thiyl Radical-Mediated Desilylation of Arylsilanes *Chem. Eur. J.* **2023**, *29*, e202203347.
